# High temperature Nb-Si alloys using data science: optimization of fracture toughness and high-temperature strength

**DOI:** 10.1038/s41467-026-75353-6

**Published:** 2026-07-10

**Authors:** Chao Xu, Dezhi Chen, Xiaofu Zhang, Qi Wang, Jingyue Yu, Shu Wang, Jingjie Guo, Hengzhi Fu, Ruirun Chen, Turab Lookman

**Affiliations:** 1 National Key Laboratory for Precision Hot Processing of Metals, Harbin Institute of Technology, Harbin, PR China; 2 AiMaterials Research LLC, Santa Fe, NM USA

**Keywords:** Metals and alloys, Mechanical properties

## Abstract

High temperature Nb-Si based alloys face a critical challenge: achieving adequate room-temperature fracture toughness ( > 18 MPa·m^1/2^) for processing while maintaining high-temperature strength, properties that typically compete with each other. Here, we overcome this inherent trade-off through machine learning-guided alloy design, employing a three-step feature screening strategy to identify 6 key descriptors from 200 initial features. SHAP analysis reveals how melting enthalpy and atomic radius mismatch control property outcomes, enabling targeted multi-objective optimization via NSGA-II algorithm. The optimized Nb-12.26Si-21.35Ti-1.98Al-1.96Cr-0.51Hf-4.34Zr-4.35 V alloy achieves an as-cast fracture toughness of 18.92 MPa·m^1/2^ while maintaining 322 MPa strength at 1250 °C, surpassing all reported as-cast Nb-Si alloys. Microstructural analysis shows that the superior properties originate from the dispersed distribution of nanoscale γ′-Nb_5_Si_3_ phase and crack deflection at phase boundaries with 67.6% lattice mismatch. Our results demonstrate that combining machine learning techniques with mechanistic understanding can accelerate the discovery of high temperature materials.

## Abstract (web summary)

Nb-Si alloys are candidates for high-temperature use but rarely combine fracture toughness with high-temperature strength. Here, the authors use machine learning to design an alloy surpassing all reported as-cast Nb-Si alloys in both properties.

## Introduction

With the rapid development of aerospace, energy, and advanced manufacturing sectors, the performance requirements for high temperature structural materials have increased significantly^1–3^. Among the candidates for next-generation high-temperature structural materials, Nb-Si-based alloys have emerged as particularly promising due to their excellent high-temperature strength, good oxidation resistance, and relatively low density^4,5^. However, high-temperature Nb-Si-based alloys face a critical challenge: achieving adequate room-temperature fracture toughness (> 18 MPa·m^1/2^) for processing while maintaining high-temperature strength. This trade-off has fundamental origins in the alloy microstructure. High-temperature strength primarily relies on the γ-Nb_5_Si_3_ silicide phase, which provides thermal stability through its high melting point and ordered D8m crystal structure. However, this phase has limited slip systems at room temperature, making it inherently brittle. Increasing the silicide volume fraction improves creep resistance at elevated temperatures through load-bearing and dislocation pinning mechanisms, but simultaneously increases crack propagation susceptibility at room temperature because cracks preferentially propagate through or along these brittle intermetallic phases. This creates an inherent design constraint where improvements in one property typically degrade the other^6^.

Traditional trial-and-error approaches struggle to systematically explore the complex composition-property relationships in Nb-Si alloys^7,8^. Machine learning (ML) offers a systematic framework to accelerate this exploration^9,10^, with data-driven approaches capable of extracting hidden patterns from experimental datasets and establishing quantitative relationships between composition and properties^11,12^. ML-based multi-objective optimization, particularly when combined with interpretability analysis, offers specific advantages for resolving property trade-offs. This integrated strategy has been achieved in the adaptive design of NiTi-based shape memory alloys^13–15^ and the optimization of hardness and strength-ductility balance in high entropy alloys^16,17^. For niobium-based alloys, Mohanty et al^18^. successfully navigated sparse data challenges through physics-guided feature engineering. However, ML applications for Nb-Si-based alloys remain limited^19,20^. While feature engineering, model interpretability, multi-objective optimization, and mechanistic integration are well-established in materials informatics, their systematic integration for Nb-Si alloys has been constrained by: (1) fragmented application of feature selection strategies; (2) limited use of interpretability tools to guide element selection; (3) isolated optimization of single properties; and (4) insufficient connection between data-driven predictions and microstructural mechanisms. Existing Nb-Si alloy studies have typically employed one or two of these approaches in isolation, rather than combining them to address the inherent trade-off between fracture toughness and high-temperature strength.

To address these challenges, we adopt a systematic data-driven strategy integrating ML with experimental validation. Our approach includes: (1) a 3-step progressive feature screening strategy that efficiently identifies six key factors from 200 initial features; (2) SHAP (SHapley Additive exPlanations) analysis to reveal quantitative feature-property relationships and guide element selection; (3) multi-objective optimization using Non-dominated Sorting Genetic Algorithm II (NSGA-II) to balance fracture toughness and high-temperature strength; (4) comprehensive experimental validation with multi-scale microstructural characterization to establish composition-structure-property relationships.

Our approach leads to the development of an 8-element as-cast alloy (Nb-12.26Si-21.35Ti-1.98Al-1.96Cr-0.51Hf-4.34Zr-4.35 V) with a fracture toughness (*K*_Q_) of 18.92 MPa·m^1/2^, while simultaneously maintaining excellent high-temperature compressive strength (HTC) of 322 MPa at 1250 °C. This achievement not only meets the dual requirements of processability and high-temperature performance but also validates the power of ML in accelerating high-temperature alloy development from years to months.

## Results and discussion

### Data collection and feature engineering

**Fig. 1:**
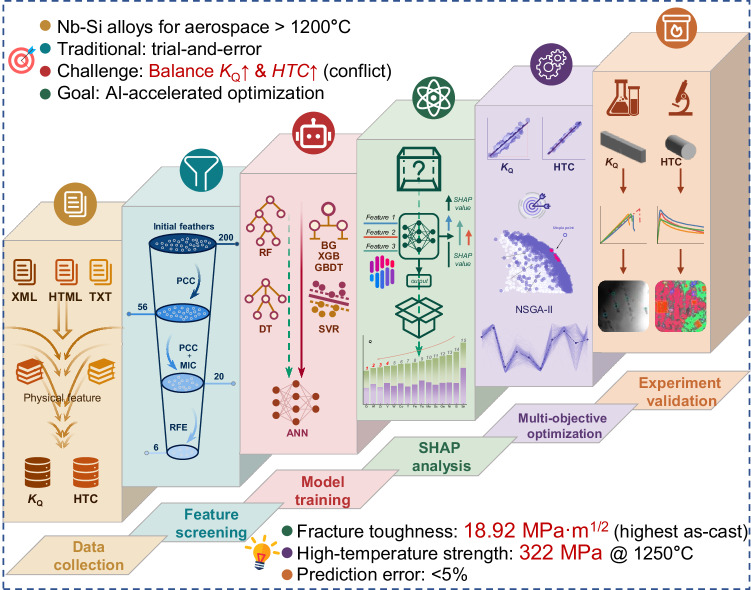
ML-based design workflow for Nb-Si alloys. The workflow comprises data collection (*n* = 203 samples for *K*_Q_; *n* = 34 for HTC), feature engineering (200 initial descriptors), three-step feature screening (PCC redundancy removal, PCC-MIC joint screening, RFE with ElasticNet), reducing to 6 key features, model training across eight algorithms with ANN selected, SHAP analysis, and NSGA-II multi-objective optimization followed by experimental validation. The optimized alloy achieves *K*_Q_ = 18.92 MPa·m^1/2^ and HTC = 322 MPa at 1250 °C, with prediction errors below 5%.

We established an ML-assisted Nb-Si alloy development system, as shown in Fig. 1. The entire process is divided into six key steps: data collection, feature screening, model training, SHAP analysis, multi-objective optimization, and experimental validation.

Our systematic literature survey collected comprehensive data on Nb-Si alloys from multiple processing routes. Analysis of element co-occurrence patterns across the complete dataset (Supplementary Fig. 1) reveals the general compositional design principles for Nb-Si alloys. The network analysis (Supplementary Fig. 1a) shows Nb-Si (100%), Nb-Ti (87%), and Nb-Cr (65%) as the dominant element pairs, with Nb as the central matrix element. Element occurrence frequency (Supplementary Fig. 1b) confirms that Nb (100%), Si (100%), Ti (87%), Cr (65%), Al (58%), Hf (42%), and Zr (38%) play central roles in alloy design. Content distribution analysis (Supplementary Fig. 1c, d) shows Si typically ranges 10-20 at.% (avg. ~14.5 at.%), while Ti exhibits a broader distribution (0-40 at.%) with averages of 18.3 at.% (*K*_Q_) and 21.5 at.% (HTC), reflecting their distinct roles in property tuning.

To ensure data consistency and eliminate processing-induced variations, we focused specifically on arc-melted, as-cast Nb-Si alloys, yielding 203 samples for *K*_Q_ prediction and 34 samples for HTC prediction. Examination of this refined dataset (Supplementary Fig. 2) establishes the baseline property distributions and optimization targets. The database is dominated by 6–7 element alloys (Supplementary Fig. 2a, b), with properties concentrated in medium performance ranges. *K*_Q_ values distribute across 7–20 MPa·m^1/2^, with 52.9% of samples falling in the 7–12 MPa·m^1/2^ range (28.5% at 7–10 MPa·m^1/2^, 24.4% at 10–12 MPa·m^1/2^), indicating relatively low fracture toughness levels. HTC values span 100–500 MPa, with 60% concentrated in the 200–400 MPa range (39% at 200–300 MPa, 21% at 300–400 MPa) (Supplementary Fig. 2c, d), representing medium-level high-temperature strength. This distribution pattern establishes clear targets for optimization through ML-guided design. Processing parameter analysis documented in Supplementary Tables 1–2 confirms controlled manufacturing conditions across the dataset (Supplementary Note 1).

### Feature screening and model construction

**Fig. 2:**
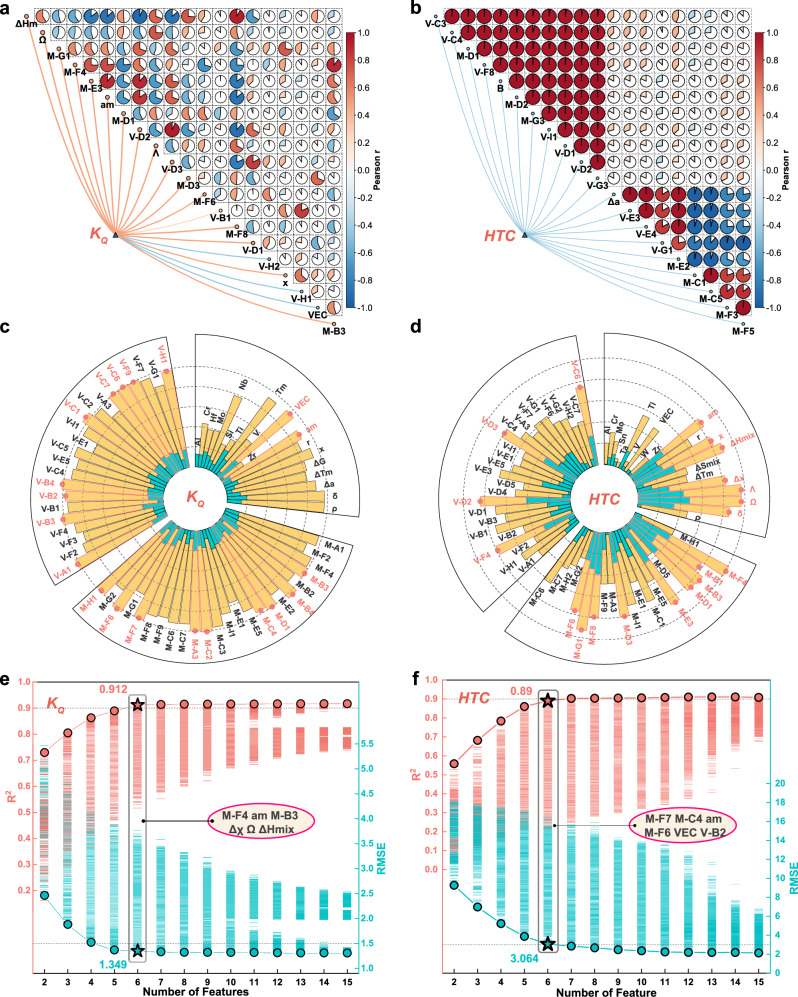
Feature selection process. **a**, **b** PCC heatmaps for the top 20 features correlated with **a** *K*_Q_ and **b** HTC. Color scale: Pearson r from −1.0 (blue) to +1.0 (red). **c**, **d** PCC-MIC joint screening for **c** *K*_Q_ and **d** HTC; bars show combined PCC and MIC scores for each feature; feature labels in red indicate retained features. **e**, **f** RFE results showing R² (red circles) and RMSE (cyan bars) versus feature number for **e** *K*_Q_ and **f** HTC; both plateau at six features (*K*_Q_: *R*^*2*^ = 0.912; HTC: *R*^*2*^ = 0.89). Feature statistics are derived from *n* = 203 independent arc-melted as-cast alloy samples (*K*_Q_) and *n* = 34 independent samples tested at 1250 °C (HTC). No technical replicates were used.

Feature screening is a key step in establishing accurate predictive models. We adopted a three-step progressive feature screening strategy to identify key factors from 200 initial features (103 for *K*_Q_ and 97 for HTC, Fig. 2), progressing from Pearson correlation coefficient (PCC)-based redundancy removal, through PCC-maximum information coefficient (MIC)^21^ joint screening, to recursive feature elimination (RFE)-based optimal subset selection.

After PCC-based redundancy removal (Fig. 2a, b), features were reduced from 103–67 (*K*_Q_, 35% reduction) and from 97–65 (HTC, 33% reduction). Joint PCC-MIC screening (Fig. 2c, d) retained the top 20 features for both models. RFE with ElasticNet identified the optimal six-feature subset, at which point both *K*_Q_ (*R*^2^ = 0.912) and HTC (*R*^*2*^ = 0.89) models reached a performance plateau; further increasing the number of features beyond six provides only marginal improvement in model performance. After the three-step screening, six key features were finally identified for both *K*_Q_ and HTC models (Supplementary Table 3). Feature selection robustness was validated through multiple independent methods documented in Supplementary Figs. 3–9, including permutation importance analysis, performance comparison with and without RFE, systematic ablation studies, and threshold optimization via grid search (Supplementary Note 2, 3).

**Fig. 3:**
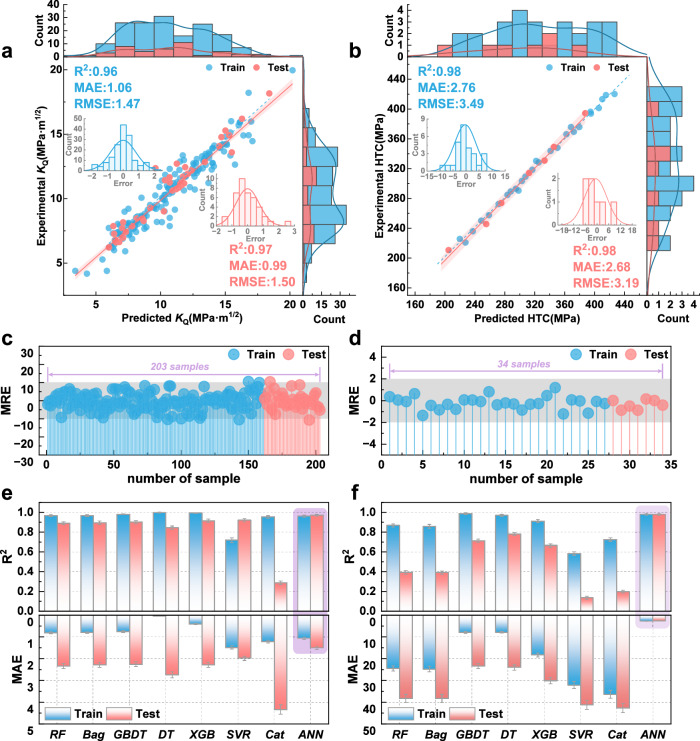
Model performance comparison. **a**, **b** Predicted versus experimental values for **a** *K*_Q_ and **b** HTC using the ANN model; blue: training set (*K*_Q_ *n* = 162; HTC *n* = 27); red: test set (*K*_Q_ *n* = 41; HTC *n* = 7); all individual data points shown. Insets: residual histograms with normal distribution curves overlaid. **c**, **d** Mean relative error (MRE, %) per sample for **c** *K*_Q_ (*n* = 203; gray band: ±10%) and **d** HTC (*n* = 34; gray band: ±2%). **e**, **f** R² and MAE for eight algorithms on **e** *K*_Q_ and **f** HTC; blue: training; red: test; dots show individual values from *n* = 5 independent runs; error bars: SD of *n* = 5 runs. All samples are independent arc-melted as-cast alloys; no technical replicates were used. Bag, bagging; DT, decision tree; XGB, extreme gradient boosting; Cat, CatBoost.

After feature screening, we built models for *K*_Q_ and HTC and compared the prediction performance of eight ML algorithms. Figure 3 shows the performance of different models on training and test sets. *K*_Q_ prediction results (Fig. 3a–c) indicate significant differences in model performance. Traditional ensemble learning methods, such as Random Forest (RF) (*R*^2^ training = 0.96, *R*^2^-test = 0.88) and Gradient Boosting Decision Tree (GBDT) (*R*^2^ training = 0.98, *R*^2^-test = 0.89) performed stably but with limited accuracy. Support Vector Regression (SVR) showed limited performance (*R*^2^ test = 0.92 for *K*_Q_, *R*^2^ test = 0.14 for HTC). The artificial neural networks (ANN) model achieved the best performance for both properties, with *R*^2^ test = 0.97 (*K*_Q_) and *R*^2^-test = 0.98 (HTC), demonstrating excellent generalization capability.

The distribution comparison between training and testing sets across the six finally selected features for the *K*_Q_ model reveals that the training and testing data show highly consistent distributions across all features (Supplementary Fig. 10). The test samples are well-covered within the main distribution regions of the training data, without shifts, outliers, or isolated regions, indicating reasonable data partitioning with good representation. Similar patterns were observed for the HTC model features (Supplementary Fig. 11). Comprehensive residual analysis for the HTC model (Supplementary Fig. 12) confirms normally distributed residuals with no systematic bias, validating fundamental statistical assumptions.

### SHAP analysis reveals feature-property relationships

**Fig. 4:**
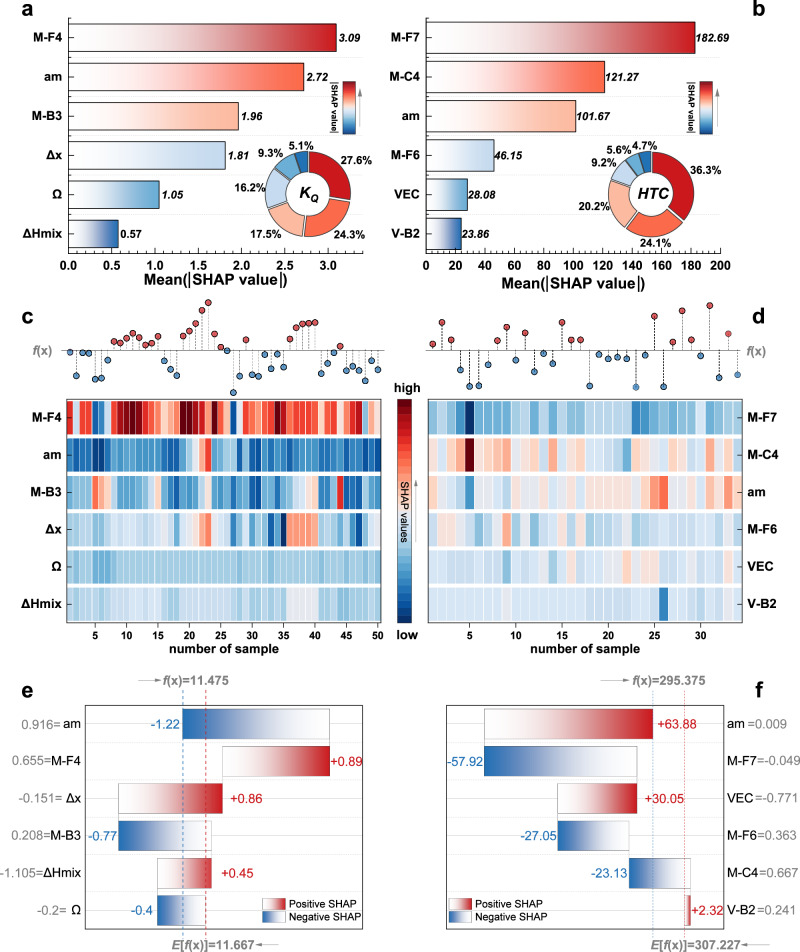
SHAP feature importance analysis. **a**, **b** Mean |SHAP| values for six key features in the **a** *K*_Q_ and **b** HTC models, calculated from all samples (*K*_Q_: *n* = 203; HTC: *n* = 34). red bars: features with positive mean SHAP; blue bars: features with negative mean SHAP; color depth indicates magnitude. Pie charts: percentage contribution of each feature to the sum of all mean |SHAP| values, representing each feature’s relative share of prediction variance and distinct from the absolute bar values. **c**, **d** SHAP heatmaps calculated from all samples for **c** *K*_Q_ (*n* = 203, first 50 samples displayed) and **d** HTC (*n* = 34, all samples displayed); red: positive SHAP values; blue: negative SHAP values; scatter above shows model output f(*x*). **e**, **f** Waterfall plots for one representative sample showing each feature’s contribution to the deviation from E[f(*x*)] for **e** *K*_Q_ and **f** HTC; red bars: positive contributions; blue bars: negative contributions.

Based on the feature screening results, {melting enthalpy (M-F4), atomic radius mismatch (am), electrophilicity index (M-B3), electronegativity difference (Δχ), thermodynamic parameter (Ω), ΔHmix} and {specific heat capacity (M-F7), covalent radius (M-C4), am, thermal expansion coefficient (M-F6), valence electron concentration (VEC), electron affinity (V-B2)} were defined as the key alloy factors influencing *K*_Q_ and HTC, respectively. To understand the influence of alloy composition on properties, SHAP analysis was applied to the trained ANN model. SHAP was selected over LIME and integrated gradients because it satisfies local accuracy, consistency, symmetry, and the dummy axioms, ensuring theoretically unbiased feature attributions. This is particularly relevant for small datasets where other methods can produce unstable attributions due to limited sample diversity. Figure 4a, b show the contribution of key features to *K*_Q_ and HTC predictions. For the *K*_Q_ prediction model (Fig. 4a), the M-F4 feature showed the most significant influence (average |SHAP| value of 3.09), followed by am (2.72), M-B3 (1.96), Δχ (1.81), Ω (1.05), and ΔHmix (0.57). Pie chart analysis shows that M-F4 and am features together contributed 55.3% of the prediction influence, reflecting the importance of melting enthalpy and atomic radius mismatch in fracture toughness prediction.

Multi-sample visualization results from the SHAP analysis revealed complex interactions between features. Heat map analysis (Fig. 4c, d) shows feature contribution distributions in *K*_Q_ and HTC models. SHAP force plots (Fig. 4e, f) precisely quantified the contribution of each feature to individual sample predictions. In the *K*_Q_ sample, the negative contribution of am (−1.22) was partially offset by positive contributions from M-F4 (+0.89) and Δχ (+0.86); in the HTC sample, the strong negative contribution of M-F7 (−57.92) was substantially offset by the strong positive contribution of am (+63.88), revealing complex interactions between features.

Dependency analysis (Supplementary Fig. 13) revealed nonlinear influence patterns of key features. The M-F4 feature had a positive contribution to toughness when below 28.5, corresponding to lower melting enthalpy, favoring the formation of ductile phases; the am feature had a significant positive contribution to toughness only when exceeding 350, indicating that moderate atomic size differences favor solid solution strengthening without damaging toughness. For high-temperature strength, am in the 352–360 range provided maximum contribution, suggesting an optimal balance between solid solution strengthening and phase stability.

Subsequently, by linearly fitting the relationship between normalized feature values and SHAP values (Supplementary Fig. 14), the influence coefficients ${k}_{{ni}}$ for key feature quantities were obtained (Supplementary Table 4). The positive or negative values of ${k}_{{ni}}$ determine the monotonic relationship between alloy factors and target properties, with the absolute value indicating the degree of influence. Based on each alloy factor, the importance of 15 elements for alloy properties was ranked (Supplementary Fig. 15). Using the weight coefficients ${Z}_{{ni}}$, weighted calculations were performed for the six key features in both *K*_Q_ and HTC models. From Supplementary Fig. 15q, the elements influencing both *K*_Q_ and HTC are ranked in descending order: Cr (12.08), Hf (13.16), Zr (13.39), V (13.83), W (14.06), and Mo (14.45). Therefore, the top four elements of Cr, Hf, Zr, and V were selected as candidate elements to enhance the properties of Nb-Si alloys.

### Multi-objective optimization and experimental validation

**Fig. 5:**
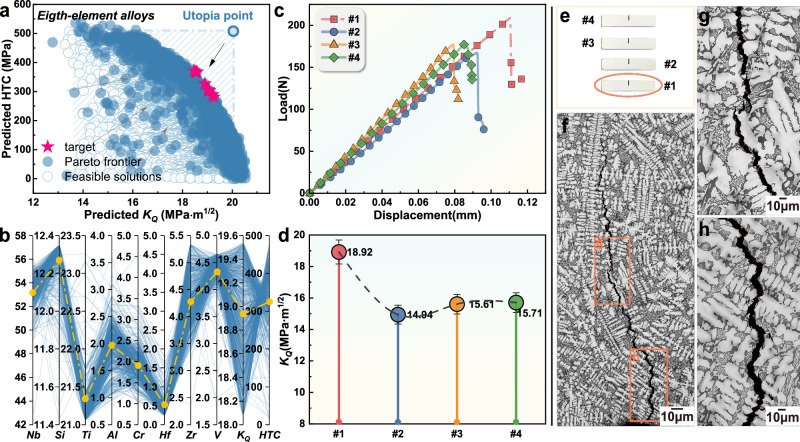
Multi-objective optimization and experimental validation. **a** Pareto frontier for the 8-element system; gray circles: feasible solutions (*n* = 10,000); pink circles: Pareto-optimal; pink stars: selected compositions; open blue circle: utopia point. **b** Parallel coordinate plot of Pareto-optimal alloys; yellow lines: validated compositions #1. Elemental compositions are in at.%; *K*_Q_ in MPa·m^1/2^; HTC in MPa. **c** Load-displacement curves from three-point bending tests (ASTM E399, 25 °C). **d** Measured *K*_Q_ for alloys #1–#4; error bars: SD of *n* = 3 independent specimens per alloy. **e** Macrographs of fractured specimens for alloys #1–#4. **f** SEM image (BSE, 20 kV) of the crack propagation path in alloy #1; orange boxes indicate the regions enlarged in (**g**, **h**), respectively; a secondary crack is visible in the lower region. **g** Enlarged view of the upper region marked in (**f**), showing crack bridging. **h** An enlarged view of the lower region marked in (**f**), showing crack deflection. No technical replicates were used. BSE, backscattered electron.

Based on the above, four alloy systems with different element combinations were constructed. For each alloy system, we formed 10,000 different alloy composition combinations within the search space shown in Supplementary Table 5, using Latin hypercube sampling to ensure uniform coverage. The Pareto fronts of multi-element alloys (Fig. 5a, b, Supplementary Fig. 16) showed a trade-off between *K*_Q_ and HTC. The 8-element and 7-element alloy systems presented broader front ranges with *K*_Q_ reaching 18–19 MPa·m^1/2^ and HTC reaching 320–360 MPa; while the 5-element and 6-element alloys had relatively narrower front regions, with *K*_Q_ upper limit of about 16 MPa·m^1/2^ and HTC upper limit of about 280 MPa. To select a single recommended alloy from the Pareto frontier, a weighted scalarization approach was used in which *K*_Q_ and HTC are combined into a single weighted sum. Both objectives were first normalized to [0, 1] across all feasible solutions to remove the effect of differences in absolute magnitude. The 1:1 weighting between *K*_Q_ and HTC reflects the requirement that both must be met simultaneously, with *K*_Q_ above 18 MPa·m^1/2^ for room-temperature processing and HTC competitive with existing Nb-Si alloys at 1250 °C. Sensitivity analysis with three schemes (*K*_Q_:HTC = 0.8:0.2, 1:1, 0.2:0.8) shows that shifting weights moves the recommended alloy along the Pareto frontier but leaves the frontier shape and compositional trends unchanged, and all recommended alloys satisfy both service thresholds under all three schemes (Supplementary Fig. 17, Supplementary Table 6). The weighting can be adjusted for specific applications, with a higher *K*_Q_ weight of 60% suited for turbine blades under cyclic loading and a higher HTC weight of 70% suited for static high-temperature structural components. To assess the stability of the Pareto frontier, we conducted 10 additional independent optimizations for the 8-element system using different random seeds. The analysis (Supplementary Table 7) shows that approximately 85% of optimal compositions identified in any single run also appeared in at least 8 out of 10 runs. Property values within each composition cluster varied by less than 3% across runs.

One composition was selected from each alloy system for experimental validation, numbered #1, #2, #3, and #4, with specific compositions shown in Supplementary Table 8. Supplementary Table 9 shows the comparison of predicted and experimental values for optimally designed alloys. Prediction errors for all alloys were within 5%. This accuracy level reaches the advanced level of material performance prediction, validating the reliability of the ML method. Supplementary Fig. 18 shows that alloys #2 and #4 fall outside the training data distribution, primarily due to their Ti concentrations (26.16 at.% and 35 at.%, respectively) exceeding the typical training range of 0–24 at.%. Additionally, alloy #4 contains only 5 elements, fewer than the dominant 6–7 element alloys in the training set. The successful prediction of these compositions with high accuracy demonstrates the extrapolation capability of the model.

Figure 5c–h shows the fracture toughness test results of the four alloys. From the load-displacement curves (Fig. 5c), it can be seen that sample #1 reached a maximum load value of approximately 200 N, significantly higher than the other samples. The measured *K*_Q_ values of the four alloys (Fig. 5d) were: #1 (18.92 MPa·m^1/2^), #2 (14.94 MPa·m^1/2^), #3 (15.61 MPa·m^1/2^), and #4 (15.71 MPa·m^1/2^).

Microstructural morphology analysis revealed two toughening mechanisms. Crack bridging (Fig. 5g) refers to the formation of bridge structures by the ductile Nbss phase spanning both sides of the crack during crack propagation. Crack deflection (Fig. 5h) manifests as the crack path deviating from its original direction^22^. These two mechanisms were particularly significant in sample #1, effectively improving the material’s fracture toughness.

Microstructural analysis was performed on the best-performing sample #1 (Supplementary Fig. 19). X-ray diffractometer (XRD) analysis confirmed that the sample mainly consisted of Nbss phase and γ-Nb_5_Si_3_ phase. Scanning electron microscope (SEM) images revealed a typical dendritic microstructure in the alloy. Energy dispersive spectrometer (EDS) analysis demonstrated the segregation patterns of various elements: Si element was mainly enriched in the gray γ-Nb_5_Si_3_ phase, while Nb element content was higher in the Nbss phase.

**Fig. 6:**
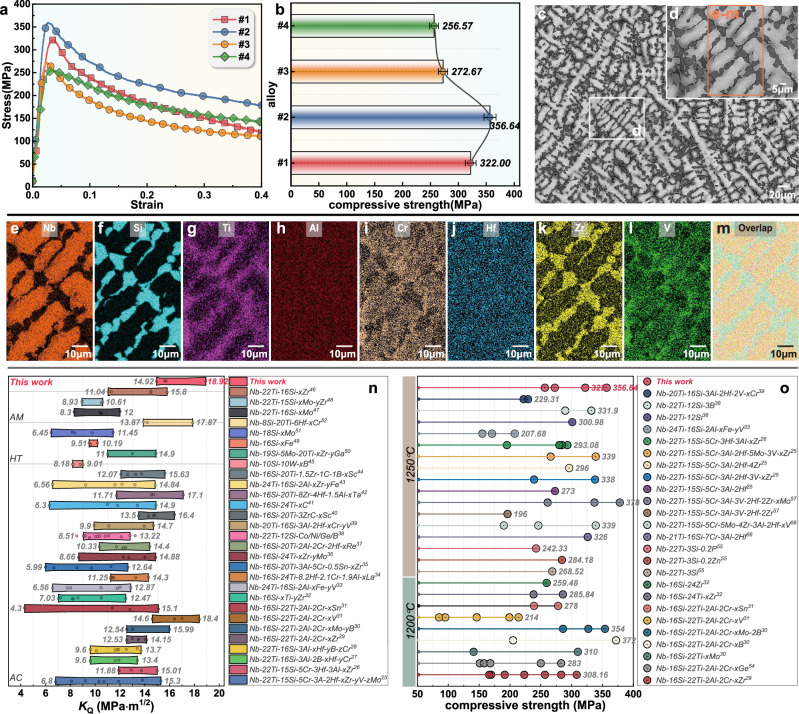
High-temperature compression testing and microstructural characterization. **a** Compressive stress-strain curves at 1250 °C for alloys #1-#4 (Gleeble 1500D, strain rate 0.005 s^-1^, vacuum). **b** Compressive strength at 1250 °C for alloys #1-#4; error bars: SD of *n* = 3 independent specimens per alloy. **c** SEM image (BSE, 20 kV) of alloy #1 after compression showing the two-phase microstructure. **d** Enlarged view of the region marked in (**c**) with EDS line scan. **e–m** EDS elemental maps of alloy #1 after compression: **e** Nb, **f** Si, **g** Ti, **h** Al, **i** Cr, **j** Hf, **k** Zr, **l** V, **m** overlap. **n**, **o** Literature comparison of **n** *K*_Q_ and **o** high-temperature compressive strength between this work and reported as-cast Nb-Si alloys; each symbol: one independently reported alloy. AM: arc-melted; HT: heat-treated; AC: as-cast.

Figure 6a, b shows the high-temperature compression performance test results of various samples at 1250 °C. The stress-strain curves (Fig. 6a) exhibited typical dynamic softening phenomena^23,24^. From Fig. 6b, it can be seen that the optimally designed alloys showed excellent high-temperature strength, with sample #2 reaching the highest value of 356.64 MPa, followed by sample #1 at 322 MPa. From microstructural analysis (Fig. 6c–m), it can be seen that the structure after high-temperature compression still consisted of the Nbss phase and γ-Nb_5_Si_3_ phase, but the eutectic regions disappeared, and dynamic recrystallization occurred.

To evaluate the performance level of our Nb-Si alloy, systematic comparisons were made with various types of Nb-Si-based alloys reported in the literature (Fig. 6n, o). Comparative analysis shows that our developed alloys outperform most existing Nb-Si alloys in both *K*_Q_^25–53^ and HTC^25,26,29–31,33,38,39,54–57^. In particular, the 18.92 MPa·m^1/2^ of sample #1 is the highest as-cast fracture toughness value reported for Nb-Si based alloys in the literature.

### Fracture toughness enhancement mechanisms

**Fig. 7:**
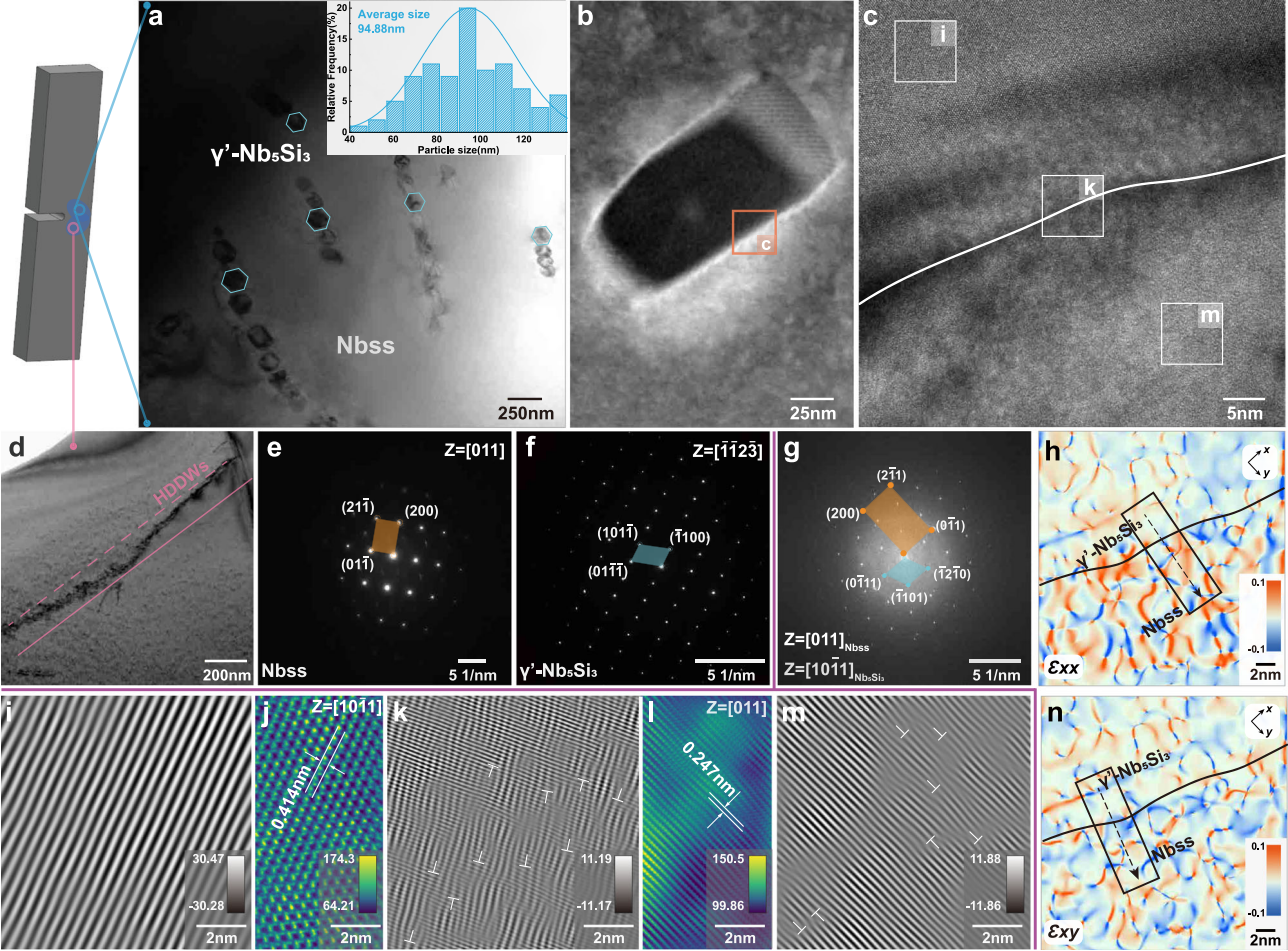
TEM characterization of alloy #1. **a** Bright-field TEM image showing dispersed γ′-Nb_5_Si_3_ nanoparticles (cyan hexagons) in the Nbss matrix; inset: particle size distribution (*n* = 32 particles); mean 94.88 nm. **b** HRTEM image of one γ′-Nb_5_Si_3_ particle; orange box: region enlarged in (**c**). **c** TEM image of the Nbss/γ′-Nb_5_Si_3_ phase interface; white boxes mark regions (**i,** **k**, and **m**) selected for analysis. **d** Low-magnification TEM image showing HDDWs near a crack in the Nbss matrix. **e** SAED pattern acquired from the Nbss phase in (**a**) (Z = [011]), confirming BCC structure; spots (211), (200), (011) indexed. **f** SAED pattern acquired from the γ′-Nb_5_Si_3_ phase in (**a**) (Z = $\left[\bar{1}\bar{1}2\bar{3}\right]$), confirming hexagonal D8m structure. **g** Composite SAED from the interface region in (**c**); superimposed spots of both phases confirm 67.6% lattice mismatch. **h**, **n** GPA maps of the interface in (**c**) showing **h** *ε*_xx_ and **n** *ε*_xy_ strain components; color scale: −0.1 (blue, compressive) to +0.1 (red, tensile); arrows: x/y reference d**i**rections. **i**, **j** IFFT images of the γ′-Nb_5_S**i**_3_ region: **i** lattice fringe image and **j** atomic column arrangement. **k** IFFT image of the interface transition zone. **l**, **m** IFFT images of the Nbss region: **l** atomic column arrangement and **m** lattice fringe image. Color scales in (**i–m**) represent IFFT image intensity (arb. units). *n* = 1 specimen; regions selected from multiple areas. Abbreviations: HDDW, high-density dislocation wall; SAED, selected-area electron diffraction.

The bright field transmission electron microscope (TEM) image (Fig. 7a, b) shows that fine and uniformly distributed hexagonal γ′-Nb_5_Si_3_ phase precipitated dispersedly within the Nbss matrix in alloy #1, with an average size of approximately 94.88 nm. SAED patterns confirm that Nbss exhibits a body-centered cubic structure (Z = $\left[011\right]$, Fig. 7e) and γ′-Nb_5_Si_3_ exhibits a hexagonal D8m structure (Z =$\left[\bar{1}\bar{1}2\bar{3}\right]$, Fig. 7f). EDS elemental maps (Supplementary Fig. 20) confirm that Si is enriched within these particles relative to the surrounding Nbss matrix, consistent with the γ′-Nb_5_Si_3_ phase composition. This nanoscale γ′ phase distribution not only avoided local stress concentration but also caused cracks to interact with Nbss/γ′ phase interfaces during propagation, forming crack bridging structures^58^.

High-resolution TEM analysis (Fig. 7c) revealed the crystal structures of three regions. The γ′ phase region showed an interplanar spacing of 0.414 nm (Fig. 7i, j), and layered atomic arrangement. The Nbss phase region showed an interplanar spacing of 0.247 nm (Fig. 7l, m) and a more compact atomic arrangement.

The diffraction pattern of the interface region (Fig. 7g) contained a superposition of diffraction spots from both phases. Inverse fast Fourier transform (IFFT) analysis (Fig. 7k) showed strain transition zones at interfaces with discontinuous and disordered atomic arrangements. The lattice mismatch between the two phases reached 67.6%, forming typical incoherent interface structures.

Geometric phase analysis (GPA) results (Fig. 7h, n) showed significant strain gradients at interfaces: the γ′ phase underwent compressive strain, while the Nbss matrix underwent tensile strain^59,60^, with dramatic strain changes at interfaces and differences of 6–8% on both sides (Supplementary Fig. 21a, b). The 67.6% lattice mismatch between Nbss and γ′-Nb_5_Si_3_ phases creates highly incoherent interfaces with substantial interfacial energy. These high-energy interfaces contribute to fracture toughness enhancement through crack deflection mechanisms rather than serving as crack initiation sites. When a propagating crack encounters the large lattice mismatch at Nbss/γ′ interfaces, the interfacial energy discontinuity causes the crack to deflect along or around the interface rather than continuing straight through^61^. Fracture surface observations (Fig. 5h) directly confirmed this deflection behavior, which increased the effective crack path length.

The critical factor enabling this beneficial deflection is the nanoscale dispersion of γ′-Nb_5_Si_3_ particles. At this length scale, cracks cannot form continuous paths through connected brittle phases, forcing repeated deflection events that dissipate fracture energy^62,63^. The schematic diagrams (Supplementary Fig. 21c, d) show the dispersed distribution of nanoscale γ′-Nb_5_Si_3_ phase in the Nbss phase and incoherent interface structures. In summary, fracture toughness enhancement originates from the synergistic combination of crack bridging by the ductile Nbss matrix (Fig. 5g) and crack deflection at high-energy Nbss/γ′ interfaces (Fig. 5h).

Quantitative crack path analysis (Supplementary Fig. 22) reveals that alloy #1 exhibits a deflection parameter D = 1.65–1.74 compared to D = 1.01–1.03 for alloy #2. This 63% increase in crack tortuosity directly confirms that the nanoscale γ′-Nb_5_Si_3_ dispersion and high-energy incoherent interfaces effectively promote crack deflection, thereby enhancing energy dissipation during fracture propagation.

### High-temperature strengthening mechanisms

**Fig. 8:**
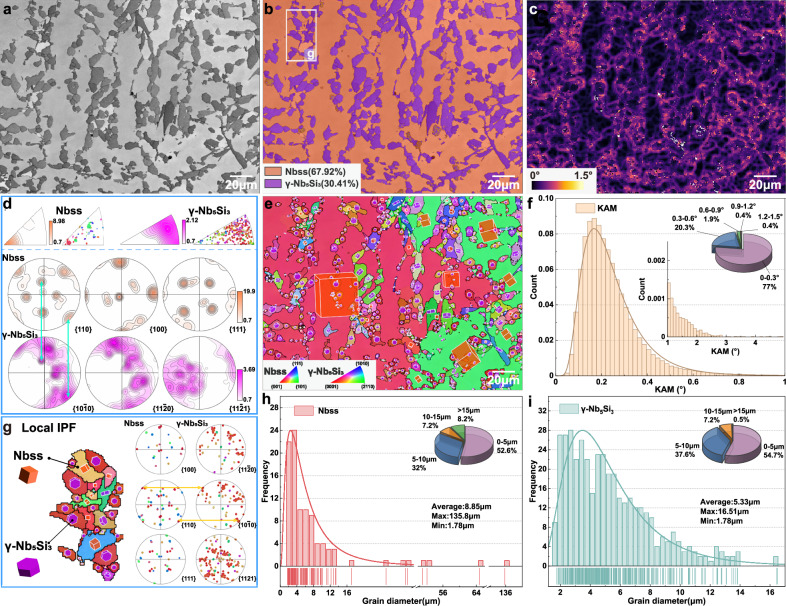
EBSD analysis of alloy #1 after compression at 1250 °C. **a** SEM (BSE, 15 kV). **b** Phase map; orange: Nbss (67.92%); purple: γ-Nb_5_Si_3_ (30.41%); ~2%: low-confidence index points (CI < 0.1) or minor silicide phases; step size 0.5 μm. **c** KAM map; **c**olor scale 0–1.5°; first nearest-neighbor kernels. **d** Pole figures for Nbss {110}/{100}/{111} and γ-Nb_5_Si_3_ $\left\{10\bar{1}0\right\}$/$\left\{11\bar{2}0\right\}$/$\left\{11\bar{2}1\right\}$; MUD color scale. **e** IPF map overlaid with unit cell orientations for Nbss and γ-Nb_5_Si_3_, with phase boundaries marked in black. **f** KAM histogram; inset pie: 0–0.3° (77%), 0.3–0.6° (20.3%), 0.6–0.9° (1.9%), 0.9–1.2° (0.4%), 1.2–1.5° (0.4%). **g** Local IPF map with IPF color keys for Nbss and γ-Nb_5_Si_3_. **h**, **i** Grain size distributions for (**h**) Nbss (*n* = 97 grains) and **i** γ- Nb_5_Si_3_ (*n* = 404 grains); curves: log-normal fits; insets: fractions in 0–5, 5–10, 10–15, >15 μm ranges; grains defined by >10° boundaries; only grains ≥10 pixels included. Abbreviations: IPF, inverse pole figure; MUD, multiples of uniform distribution; CI, confidence index.

Electron backscatter diffraction (EBSD) analysis in Fig. 8 revealed the microstructural evolution and strengthening mechanism of sample #1 after high-temperature compression at 1250 °C. Phase distribution and proportion (Fig. 8a, b) showed that after high-temperature compression, the alloy formed a stable two-phase structure dominated by the Nbss matrix (68%) and γ-Nb_5_Si_3_ phase (30%), with a small amount (approximately 2%) of other silicide phases.

Kernel average misorientation (KAM) analysis (Fig. 8c, f) revealed different deformation behavior of the two phases: the γ phase had higher KAM values than the Nbss phase, indicating that the hard and brittle γ phase accumulated higher strain gradients and lattice distortions in local regions. In contrast, the Nbss phase with its BCC structure’s multiple slip systems could effectively release stress through dislocation slip and dynamic recovery^64^.

From pole figure analysis in Fig. 8d, g, specific orientation relationships existed between the Nbss phase and γ phase: $\left\{110\right\}$Nbss//$\left\{10\bar{1}0\right\}$γ-Nb_5_Si_3_ and $\left\{100\right\}$Nbss//$\left\{11\bar{2}0\right\}$γ-Nb_5_Si_3_. These specific orientation relationships enable geometric compatibility between the slip systems of Nbss and γ phase, facilitating deformation coordination.

Grain size analysis (Fig. 8e, h, i) showed that the average grain size of the γ phase (5.33 μm) was significantly smaller than that of the Nbss phase (8.85 μm). The fine γ phase grains provided effective second phase strengthening^65^; the larger Nbss grains ensured sufficient mean free path for dislocations, favoring dislocation slip and dynamic recovery.

### Connecting ML-identified features to microstructural mechanisms

To establish the physical connection between ML-identified key features and the observed strengthening mechanisms, we systematically analyzed how each feature relates to microstructural characteristics. Quantitative regression analysis of all six ML-selected descriptors against microstructural parameters (Supplementary Tables 10, 11, Supplementary Note 4) enables data-supported validation of mechanistic interpretations.

For *K*_Q_, regression analysis against microstructural parameters reveals statistically significant correlations for three descriptors (Supplementary Table 11, Supplementary Fig. 23). ΔHmix shows the strongest correlation with Nbss vol.% (*r* = −0.386, *p* < 0.001, *n* = 78) and Nbss phase size (*r* = 0.702, *p* < 0.001, *n* = 24), while Ω shows the strongest correlation with silicide vol.% (*r* = −0.477, *p* < 0.001, *n* = 78). TEM characterization confirms that lower ΔHmix reduces the thermodynamic driving force for silicide formation, promoting nanoscale γ′-Nb_5_Si_3_ dispersion (average 94.88 nm, Fig. 7a) that forces crack bridging and deflection (Fig. 5g, h). Lower Ω simultaneously constrains silicide volume fraction, preventing continuous brittle network formation. For interface structure, M-B3 exhibits the strongest correlation with lattice mismatch *δ* (*r* = 0.814, *p* < 0.001, *n* = 17), with GPA quantifying 6–8% strain gradients at Nbss/γ′ interfaces (Fig. 7h, n), confirming that the interfacial bonding heterogeneity captured by M-B3 directly governs the high-energy incoherent interface structure that promotes crack deflection along phase boundaries.

For HTC, regression analysis identifies specific heat capacity (M-F7) as the strongest correlator with silicide vol.% (*r* = −0.271, *p* < 0.05, *n* = 78) and lattice mismatch *δ* (*r* = −0.684, *p* < 0.01, *n* = 17), indicating its dual role in governing both phase stability and interface characteristics. EBSD analysis after compression at 1250 °C (Fig. 8c, f) confirms that this manifests as differential strain accumulation, with the γ phase exhibiting higher KAM values than the Nbss phase, maintaining structural integrity and load-bearing capacity while the Nbss matrix undergoes dynamic recovery. VEC exhibits the strongest correlation with Nbss phase size (*r* = 0.780, *p* < 0.001, *n* = 24), quantitatively linking grain refinement to the crystallographic orientation relationships {110}Nbss//$\left\{10\bar{1}0\right\}$γ and {100}Nbss//$\left\{11\bar{2}0\right\}$γ (Fig. 7d, g) that enable geometric slip system matching and coordinated deformation (Supplementary Table 11).

Having established the feature-mechanism connections, we quantitatively benchmarked ML performance against traditional approaches. First, for HTC prediction, we compared ML with Labusch-Nabarro solid solution strengthening theory (Supplementary Table 12). The physics-based model showed prediction deviations of 10–15% from experiments, while ML predictions matched experimental values within 5% error, demonstrating the model’s ability to implicitly learn multi-mechanism interactions. Second, for *K*_Q_ prediction, we implemented three traditional physical models (Hall-Petch, Griffith, constrained mixture) on validated alloys, with detailed parametrization documented in Supplementary Figs. 24-27, Supplementary Tables 13–16 and Supplementary Note 5. Across both properties, the ML model achieves three-fold to 30-fold improvement in prediction accuracy compared to single-mechanism traditional models. Partial dependence analysis (Supplementary Figs. 28–31) reveals interpretable nonlinear feature interactions consistent with measured microstructural trends. Extrapolation assessment using multiple distance metrics (Supplementary Figs. 32, 33) demonstrates controlled predictive capability beyond training data ranges (Supplementary Note 6).

In summary, we developed an ML-guided strategy overcoming the trade-off between fracture toughness and high-temperature strength in Nb-Si alloys. A three-step feature screening identified 6 critical factors from 200 features, enabling an ANN model with *R*^*2*^ > 0.96. Quantitative validation through regression analysis, baseline model comparison, and extrapolation assessment established data-supported correlations between ML descriptors and microstructural parameters. SHAP analysis guided element selection (Cr, Hf, Zr, V) for NSGA-II optimization, yielding an 8-element alloy achieving 18.92 MPa·m^1/2^ toughness and 322 MPa strength at 1250 °C. The superior properties originate from nanoscale γ′-Nb_5_Si_3_ dispersion and high-energy interface-mediated crack deflection. This strategy demonstrates that combining established ML techniques with mechanistic understanding can accelerate the discovery of high-temperature materials, with broader applicability to other complex alloy systems facing similar property trade-offs.

## Methods

### Experimental design

This study aimed to develop Nb-Si-based alloys with balanced room-temperature fracture toughness and high-temperature strength using ML-guided design. The experimental workflow is described in Results and detailed in the subsequent sections.

### Data collection and subset construction

To construct a reliable training dataset for ML models, we conducted a comprehensive literature survey across major databases, including Web of Science and Materials Project, integrating Nb-Si alloy-related information on composition and properties. Special attention was paid to two key performance indicators: *K*_Q_ and HTC, establishing specialized subsets with 203 and 34 data points, respectively.

A critical challenge in materials database construction is handling the impact of different processing methods on alloy performance. Our comprehensive analysis of published Nb-Si alloy research revealed that processing methods are highly diverse: approximately 70% of published data used arc melting to produce as-cast samples, while the remaining 30% employed various alternative methods, including directional solidification, laser additive manufacturing, powder metallurgy, and post-casting heat treatments. However, incorporating samples from different processing routes would introduce significant modeling complications, as each processing method requires specific parameter features that would create sparse feature matrices where most parameters would be zero for most samples.

To ensure training data consistency and enable robust statistical modeling within a well-defined processing domain, we systematically selected only arc-melted, as-cast samples. Arc melting with controlled remelting, typically 7 or more cycles, produces reproducible microstructures characterized by equiaxed dendritic solidification and relatively uniform composition with limited microsegregation. This processing homogeneity is essential because it isolates the effect of chemical composition on properties, which is precisely what our ML models are designed to capture. By controlling for processing route, we eliminate confounding variables that would otherwise obscure composition-property relationships.

We further restricted our dataset to compositions containing commonly studied elements: Nb, Si, Ti, Al, Cr, Hf, Zr, V, Mo, W, Ta, and Sn. This filtering excluded samples with unconventional alloying elements such as La, In, Co, Ni, and Sc that appear in only a few compositions. Including these rare elements would exacerbate feature sparsity issues, as many elemental features would be undefined or zero for most samples in the database. This systematic filtering process resulted in our final database comprising 203 samples for *K*_Q_ prediction and 34 samples for HTC prediction.

All samples in the *K*_Q_ dataset were arc-melted, as-cast, and tested at room temperature. All samples in the HTC dataset were arc-melted, as-cast, and tested at 1250 °C under identical conditions: strain rate of 0.005 s⁻^1^ and 40% compression deformation. Processing parameter variance decomposition (Supplementary Fig. 34, Supplementary Table 17) confirms that compositional factors account for ~96% of total *K*_Q_ variance, with processing variations and measurement uncertainties combined contributing only ~4%, justifying the use of composition-based descriptors as model inputs.

### Construction and screening of key alloy factors

This study systematically extracted the intrinsic physicochemical features of various elements for *K*_Q_ and HTC models, including (1) atomic properties; (2) electronic properties; (3) thermodynamic properties; (4) physical transport properties; (5) mechanical properties; (6) structural characteristics, as detailed in Supplementary Tables 18–22. To expand from element-level features to alloy-level descriptors, alloy feature factors were calculated through weighted average and weighted variance:

1 $${f}_{{mi}}=\sum ({f}_{{ij}}\times {\alpha }_{j})/\sum {\alpha }_{j}$$

2 $${f}_{{vi}}=\sum \left[{\left({f}_{{ij}}-{f}_{{mi}}\right)}^{2}\times {\alpha }_{j}\right]/\sum {\alpha }_{j}$$

where ${f}_{{ij}}$ is the i-th feature value of element j; ${\alpha }_{j}$ is the atomic percentage of element j; ${f}_{{mi}}$ and ${f}_{{vi}}$ are the feature mean and variance factors of the alloy, respectively.

### Feature screening methodology

Feature screening is essential for establishing accurate predictive models while avoiding overfitting in small dataset scenarios. We adopted a three-step progressive strategy to systematically identify key factors from the initial 200 features. This approach addresses specific challenges in materials informatics: high dimensionality relative to sample size (103 features for 203 *K*_Q_ samples and 97 features for 34 HTC samples, 200 in total), strong collinearity among element-derived features due to periodic trends, and potential nonlinear composition-property relationships. Single-step methods such as Lasso or random forest importance ranking were not used because no single method simultaneously handles collinearity removal, nonlinear association detection, and regularized subset selection under a low sample-to-feature ratio.

The first step evaluated linear correlation between features by calculating the PCC. When the correlation coefficient between two features exceeded |*r*| > 0.95, indicating redundant information, the feature with lower correlation to the target property was removed.

The second step employed joint screening using both PCC and MIC to capture linear and nonlinear feature-target relationships. MIC detects associations with values ranging from 0 (no relationship) to 1 (perfect predictability), complementing PCC’s linear correlation analysis. By requiring features to pass both thresholds simultaneously, we ensured retention of features demonstrating relevance through either linear or nonlinear metrics, thereby reducing computational cost and noise while avoiding premature exclusion of potentially relevant features. The *K*_Q_ model applied thresholds of PCC > 0.2 and MIC > 0.2, while the HTC model used adjusted thresholds of PCC > 0.09 and MIC > 0.08 to accommodate weaker correlations in the smaller dataset.

The third step applied RFE with ElasticNet regression as the base estimator to select the optimal feature subset from the pre-screened features. ElasticNet combines L1 and L2 regularization (alpha = 0.01, l1_ratio = 0.5), making it particularly suitable for small datasets as it performs automatic feature selection while preventing overfitting. The RFE algorithm iteratively removes the least important features based on ElasticNet coefficients and evaluates subsets using cross-validation. Feature importance was ranked by coefficient magnitude, and the optimal number of features was determined by identifying the plateau in cross-validated R² performance.

### Model construction and evaluation

After screening key features, multiple ML models were constructed for *K*_Q_ and HTC prediction. Eight algorithms were compared: RF, Extreme Gradient Boosting, GBDT, DT, Bagging, CatBoost, and ANN, with detailed hyperparameter settings provided in Supplementary Table 23.

For the ANN model, in the data preprocessing stage, all features were standardized to eliminate dimensional effects. Complete specifications of the network architecture, optimization protocol, regularization strategies, training parameters, feature selection methodology, and NSGA-II optimization settings are provided in Supplementary Tables 24, 25. To address statistical robustness for the HTC model with limited samples (*n* = 34), the architecture was simplified with lightweight Dropout regularization (Supplementary Table 25). Uncertainty quantification through bootstrap ensemble and Monte Carlo dropout methods (Supplementary Figs. 35, 36) provides calibrated confidence intervals, with learning curve analysis (Supplementary Fig. 37) confirming model convergence (Supplementary Note 7). Model performance was evaluated using:

3 $${R}^{2}=1-\frac{{\sum }_{i=1}^{n}\,{({Ex}{p}_{i}-\Pr {e}_{i})}^{2}}{{\sum }_{i=1}^{n}\,{({Ex}{p}_{i}-\bar{{Exp}})}^{2}}$$

4 $${RMSE}=\sqrt{\frac{1}{n} {\sum }_{i=1}^{n}\,{({Ex}{p}_{i}-\Pr {e}_{i})}^{2}}$$

5 $${MAE}=\frac{1}{n}{\sum }_{i=1}^{n}\,{|Ex}{p}_{i}-\Pr {e}_{i}|$$

where ${Ex}{p}_{i}$ and $\Pr {e}_{i}$ are experimental and predicted values, respectively, $\bar{{Exp}}$ is the mean of experimental values, and n is the number of samples.

To rigorously evaluate model robustness and validate generalization performance in small dataset scenarios, we implemented rigorous five-fold cross-validation for both models. For the *K*_Q_ model (203 samples), cross-validation yields consistent performance across all folds with a mean R² of 0.94 ± 0.01, RMSE of 0.97 ± 0.07 MPa·m^1/2^ and MAE of 0.72 ± 0.04 MPa·m^1/2^. For the HTC model (34 samples), despite the limited data, we obtain a mean R² of 0.89 ± 0.01, RMSE of 4.4 ± 0.3 MPa, and MAE of 3.4 ± 0.2 MPa. The small standard deviations across folds demonstrate that our models achieve stable performance across different data partitions, indicating they capture genuine composition-property relationships rather than overfitting to specific training subsets (Supplementary Tables 26, 27). This k-fold validation complements our existing validation approach, which includes dynamic train-test splitting with different random seeds across multiple iterations, rolling averages over 5-iteration windows. The representativeness of the 34-sample HTC dataset was assessed by PCA of the 11-dimensional composition space. Training and test subsets occupy overlapping regions (Supplementary Fig. 38a, b); Kolmogorov-Smirnov tests on PC1 and PC2 scores give *p* = 0.663 and *p* = 0.427, confirming no distributional bias between the two groups (Supplementary Fig. 38c, d). The 34 samples cover Si from 12–18 at.%, Ti from 0–35 at.%, and alloy complexity from 5–7 elements, spanning the main compositional space of cast Nb-Si alloys. The limited sample size restricts coverage in multi-element regions; predictions within the validated composition range are reliable, and extrapolation outside the training distribution should be treated with caution.

### SHAP analysis-based alloy element selection

To enhance model interpretability, the SHAP analysis was applied to the Nb-Si alloy system. It was developed in game theory and, when applied to ML models, reflects the marginal contribution of features to model predictions^66,67^. Compared to traditional feature importance analysis, SHAP satisfies theoretical properties such as local accuracy, consistency, symmetry, and dummy, ensuring reliability and fairness of analysis results.

Based on SHAP analysis results, we developed a quantitative element ranking method. First, we obtained influence coefficients (${k}_{{ni}}$) by linear regression between normalized feature values and SHAP values, where positive ${k}_{{ni}}$ indicates feature-property positive correlation. We next calculated weight coefficients ${Z}_{{ni}}=\frac{|{k}_{{ni}}|}{{\sum }_{i=1}^{m}\,|{k}_{{ni}}|}$ to reflect each feature’s relative importance. For each element, the rank position ${q}_{i}$ within each feature (determined by whether higher or lower feature values are beneficial) was weighted by ${Z}_{{ni}}$ to yield property-specific scores: ${S}_{{K}_{Q}}={\sum }_{i=1}^{6}\,{Z}_{{ni}}^{{K}_{Q}}\times {q}_{i}^{{K}_{Q}}$ and ${S}_{{HTC}}={\sum }_{i=1}^{6}\,{Z}_{{ni}}^{{HTC}}\times {q}_{i}^{{HTC}}$. Finally, elements were ranked using weighted scores: ${S}_{{total}}={S}_{{K}_{Q}}+{S}_{{HTC}}$. Lower total scores indicate stronger beneficial effects. This approach identified Cr, Hf, Zr, and V as the top four elements for enhancing both *K*_Q_ and HTC.

### Multi-objective optimization and experimental validation

To achieve balanced optimization of *K*_Q_ and HTC, NSGA-II was used for multi-objective optimization based on the trained models^68^. NSGA-II was selected because it generates a complete Pareto front in a single optimization run without requiring separate optimizations at each weight setting, and has been widely validated for continuous alloy composition design. The population size was set to 100, the number of iterations was 100 generations, the crossover probability was 0.9, and the mutation probability was 0.3. To verify the accuracy of the model, four alloy systems of different complexity were designed based on element rankings: 8-element (Nb-Si-Ti-Al-Cr-Hf-Zr-V), 7-element (Nb-Si-Ti-Al-Cr-Hf-Zr), 6-element (Nb-Si-Ti-Al-Cr-Hf), and 5-element (Nb-Si-Ti-Al-Cr) alloys.

Samples were synthesized by arc melting using high-purity metal raw materials (purity >99.9%) supplied by Beijing Dream Material Technology Co., Ltd. (Beijing, China). Melting was conducted under an inert argon atmosphere (purity > 99.999%, pressure 0.05 MPa), and each sample was remelted at least 7 times to ensure compositional homogeneity with cooling on a water-cooled copper hearth. No post-casting heat treatment was applied. The obtained ingots were sectioned into standard specimens by wire electrical discharge machining (EDM) for mechanical testing and microstructural characterization.

Fracture toughness was measured by three-point bending at room temperature (25 °C) following ASTM E399. Specimen dimensions were 2 × 4 × 20 mm with a notch pre-crack of approximately 2 mm introduced by low-speed diamond wire cutting, with a span of 16 mm and a crosshead speed of 0.05 mm/min. High-temperature compression testing was performed on a Gleeble 1500D thermal simulation machine (Dynamic Systems Inc., USA) using cylindrical specimens (⌀6 × 9 mm). Specimens were resistance-heated to 1250 °C at 10 °C/s, held for 3 min, then compressed at a strain rate of 0.005 s⁻^1^ to 40% deformation under vacuum. Temperature was monitored by a K-type thermocouple spot-welded at the specimen mid-height, with temperature fluctuation controlled to ±2 °C throughout testing.

Phase composition was analyzed by X-ray diffraction (XRD, Empyrean, PANalytical, Netherlands) with Cu Kα radiation, operating at 45 kV and 40 mA over a 2θ range of 20°–100° at a scan rate of 8°/min. Microstructure was characterized by scanning electron microscopy (SEM, Merlin Compact, Zeiss, Germany) under backscattered electron (BSE) mode at 20 kV. Elemental distribution was analyzed using the integrated energy-dispersive X-ray spectroscopy (EDS, X-Max 80 mm^2^ SDD, Oxford Instruments). EBSD was performed on a Zeiss Gemini 560 field-emission SEM (Zeiss, Germany) equipped with an Oxford Instruments Symmetry S2 detector at 20 kV with a step size of 0.5 μm. Cross-sectional surfaces for EBSD were prepared by cross-section ion polishing. EBSD data were indexed and analyzed using AZtec software (Oxford Instruments) and ATEX software.

TEM was performed on a FEI Talos F200X instrument (FEI, USA) operated at 200 kV, equipped with a Super-X EDS detector system (four windowless silicon drift detectors). TEM foils were prepared by mechanical thinning to less than 50 μm, followed by ion thinning to electron transparency using a precision ion polishing system (GATAN 695). HRTEM images, SAED patterns, and GPA were used to characterize interface structures, crystal orientations, and local strain distribution near phase boundaries.

### Statistics & reproducibility

No statistical method was used to predetermine sample size. The dataset size (203 samples for *K*_Q_ and 34 samples for HTC) was determined by the availability of published experimental data. No data were excluded from the analyses. The experiments were not randomized. The Investigators were not blinded to allocation during experiments and outcome assessment. Machine learning model performance was evaluated using five-fold cross-validation. Error bars in figures represent standard deviation (SD) unless otherwise stated. Statistical significance was assessed at *p* < 0.05.

## Supplementary information

Supplementary Information

Transparent Peer Review file

## Source data

Source data

## Data Availability

The data generated in this study are provided in the Supplementary Information and Source Data files. The raw data relevant to the study are available at the GitHub repository (https://github.com/chaoxu-HIT/report-code) and have been archived on Zenodo 10.5281/zenodo.20302962^69^. Source data are provided with this paper.

## Code availability

All codes are available at the GitHub repository (https://github.com/chaoxu-HIT/report-code) and have been archived on Zenodo 10.5281/zenodo.20302962.
