# Supplementary material for: High temperature Nb-Si alloys using data science: optimization of fracture toughness and high-temperature strength
Source: Nat Commun. 2026 Jul 10;17:8503. doi: 10.1038/s41467-026-75353-6 (PMC13483041; doi:10.1038/s41467-026-75353-6)
Supplement: Supplementary file 1 [file 41467_2026_75353_MOESM1_ESM.pdf]

# Supplementary Information

# High temperature Nb-Si alloys using data science: optimization of fracture toughness and high-temperature strength

Chao Xu[1], Dezhi Chen[1*], Xiaofu Zhang[1], Qi Wang[1], Jingyue Yu[1], Shu Wang[1], Jingjie Guo[1], Hengzhi Fu[1], Ruirun Chen[1*], Turab Lookman[2*].

[1] National Key Laboratory for Precision Hot Processing of Metals, Harbin Institute of Technology, Harbin, 150001, PR China.

[2] AiMaterials Research LLC, Santa Fe, NM, 87501, USA.

*Corresponding author: Dezhi Chen, chendezhi383@163.com; Runrui Chen, ruirunchen@hit.edu.cn; Turab Lookman, turablookman@gmail.com

This PDF file includes:
Supplementary Figs. 1 to 38
Supplementary Tables 1 to 27
Supplementary Notes. 1 to 7
Supplementary References

# Table of Contents

**List of Figures**


| | | |
|---|---|---|
| Supplementary Fig. 1 | Dataset composition and feature distribution. | 5 |
| Supplementary Fig. 2 | Data distribution in the $K_Q$ and HTC datasets. | 6 |
| Supplementary Fig. 3 | Independent validation of feature selection. | 7 |
| Supplementary Fig. 4 | Model performance comparison with and without RFE for the $K_Q$ model. | 8 |
| Supplementary Fig. 5 | Model performance comparison with and without RFE for the HTC model. | 9 |
| Supplementary Fig. 6 | Impact of feature selection on model performance. | 10 |
| Supplementary Fig. 7 | NSGA-II versus random search for Pareto optimization. | 11 |
| Supplementary Fig. 8 | Grid search results across the PCC–MIC threshold space. | 12 |
| Supplementary Fig. 9 | Performance comparison of the top-5 threshold combinations. | 13 |
| Supplementary Fig. 10 | Feature distribution comparison for the $K_Q$ model. | 14 |
| Supplementary Fig. 11 | Feature distribution comparison for the HTC model. | 15 |
| Supplementary Fig. 12 | HTC model residual diagnostics. | 16 |
| Supplementary Fig. 13 | SHAP dependence plots for key features. | 17 |
| Supplementary Fig. 14 | Normalized alloy factors and corresponding SHAP values. | 18 |
| Supplementary Fig. 15 | SHAP-guided element importance ranking. | 19 |
| Supplementary Fig. 16 | Multi-objective optimization results for 7-, 6-, and 5-element alloy systems. | 20 |
| Supplementary Fig 17 | Sensitivity analysis of Pareto optimization to weighting schemes. | 21 |
| Supplementary Fig. 18 | t-SNE extrapolation analysis of optimized compositions. | 22 |
| Supplementary Fig. 19 | Microstructure of alloy #1. | 23 |
| Supplementary Fig. 20 | EDS elemental maps of the γ′-$Nb_5Si_3$ nanoparticle in Fig. 7c. | 24 |
| Supplementary Fig. 21 | Interfacial strain profiles and toughening mechanism schematics. | 25 |
| Supplementary Fig. 22 | Quantitative crack deflection analysis. | 26 |
| Supplementary Fig. 23 | Correlation between ML descriptors and microstructural parameters. | 27 |
| Supplementary Fig. 24 | EBSD grain size maps for the four validation alloys. | 28 |
| Supplementary Fig. 25 | EBSD phase distribution maps for the four validation alloys. | 29 |
| Supplementary Fig. 26 | Phase-specific nanoindentation results for the four validation alloys. | 30 |
| Supplementary Fig. 27 | Prediction accuracy comparison between ML and physics-based models. | 31 |
| Supplementary Fig. 28 | One-dimensional partial dependence plots for the $K_Q$ model. | 32 |
| Supplementary Fig. 29 | Partial dependence gradient analysis for the $K_Q$ model. | 33 |
| Supplementary Fig. 30 | Two-dimensional partial dependence interaction analysis for the $K_Q$ model. | 34 |
| Supplementary Fig. 31 | Shapley Interaction Index analysis for the $K_Q$ model. | 35 |
| Supplementary Fig. 32 | Distance metric analysis for $K_Q$ predictions. | 36 |

| | | |
|---|---|---|
| Supplementary Fig. 33 | Distance metric analysis for HTC predictions. | 37 |
| Supplementary Fig. 34 | Correlation between processing parameters and $K_Q$. | 38 |
| Supplementary Fig. 35 | Bootstrap ensemble uncertainty quantification for the HTC model. | 39 |
| Supplementary Fig. 36 | Monte Carlo dropout uncertainty quantification for the HTC model. | 40 |
| Supplementary Fig. 37 | Learning curve analysis for the HTC model. | 41 |
| Supplementary Fig. 38 | PCA representativeness analysis of the HTC dataset. | 42 |

**List of Tables**

| | | |
|---|---|---|
| Supplementary Table 1 | Processing parameter database for the 203 $K_Q$ dataset alloys. | 43 |
| Supplementary Table 2 | Summary statistics for processing parameters across the dataset. | 48 |
| Supplementary Table 3 | Key features identified for $K_Q$ and HTC prediction. | 49 |
| Supplementary Table 4 | Influence and weight coefficients from SHAP analysis. | 50 |
| Supplementary Table 5 | Composition search space for multi-objective optimization. | 51 |
| Supplementary Table 6 | Predicted $K_Q$ and HTC of recommended alloys under three weighting schemes. | 52 |
| Supplementary Table 7 | Pareto frontier stability assessment for the 8-element system. | 53 |
| Supplementary Table 8 | Compositions of the four experimental validation alloys (at.%). | 54 |
| Supplementary Table 9 | Predicted and experimental $K_Q$ and HTC for the four validation alloys. | 55 |
| Supplementary Table 10 | Microstructural parameters for experimentally characterized Nb-Si alloys. | 56 |
| Supplementary Table 11 | Regression results for ML descriptors against microstructural parameters. | 58 |
| Supplementary Table 12 | Comparison of ML and physics-based HTC predictions. | 59 |
| Supplementary Table 13 | Hall–Petch-type $K_Q$ predictions for the four validation alloys. | 61 |
| Supplementary Table 14 | Phase-based model parameters for Griffith and constrained mixture models. | 62 |
| Supplementary Table 15 | Griffith model $K_{IC}$ calculations for the four validation alloys. | 63 |
| Supplementary Table 16 | Constrained mixture model $K_{IC}$ calculations for the four validation alloys. | 64 |
| Supplementary Table 17 | Variance decomposition for the $K_Q$ dataset. | 65 |
| Supplementary Table 18 | Elemental features for the $K_Q$ model. | 66 |
| Supplementary Table 19 | Elemental feature values for the $K_Q$ model. | 67 |
| Supplementary Table 20 | Elemental features for the HTC model. | 69 |

Supplementary Table 21 Elemental feature values for the HTC model. 70
Supplementary Table 22 Alloy-level descriptor definitions for Nb-Si alloys. 72
Supplementary Table 23 Hyperparameter configurations for eight ML algorithms. 74
Supplementary Table 24 ANN model configuration for $K_Q$ prediction. 75
Supplementary Table 25 ANN model configuration for HTC prediction. 77
Supplementary Table 26 Five-fold cross-validation results for the $K_Q$ model. 79
Supplementary Table 27 Five-fold cross-validation results for the HTC model. 80

**List of Supplementary Notes**

Supplementary Note 1 Dataset Construction and Feature Engineering. 81
Supplementary Note 2 Feature Selection Validation and Robustness. 85
Supplementary Note 3 Ablation Study. 91
Supplementary Note 4 Quantitative Descriptor–Microstructure Relationships. 94
Supplementary Note 5 Baseline Model Comparisons for Toughness Prediction. 98
Supplementary Note 6 Model Extrapolation. 101
Supplementary Note 7 Statistical Robustness of HTC Model. 103
Supplementary Reference 107

## Supplementary Figures

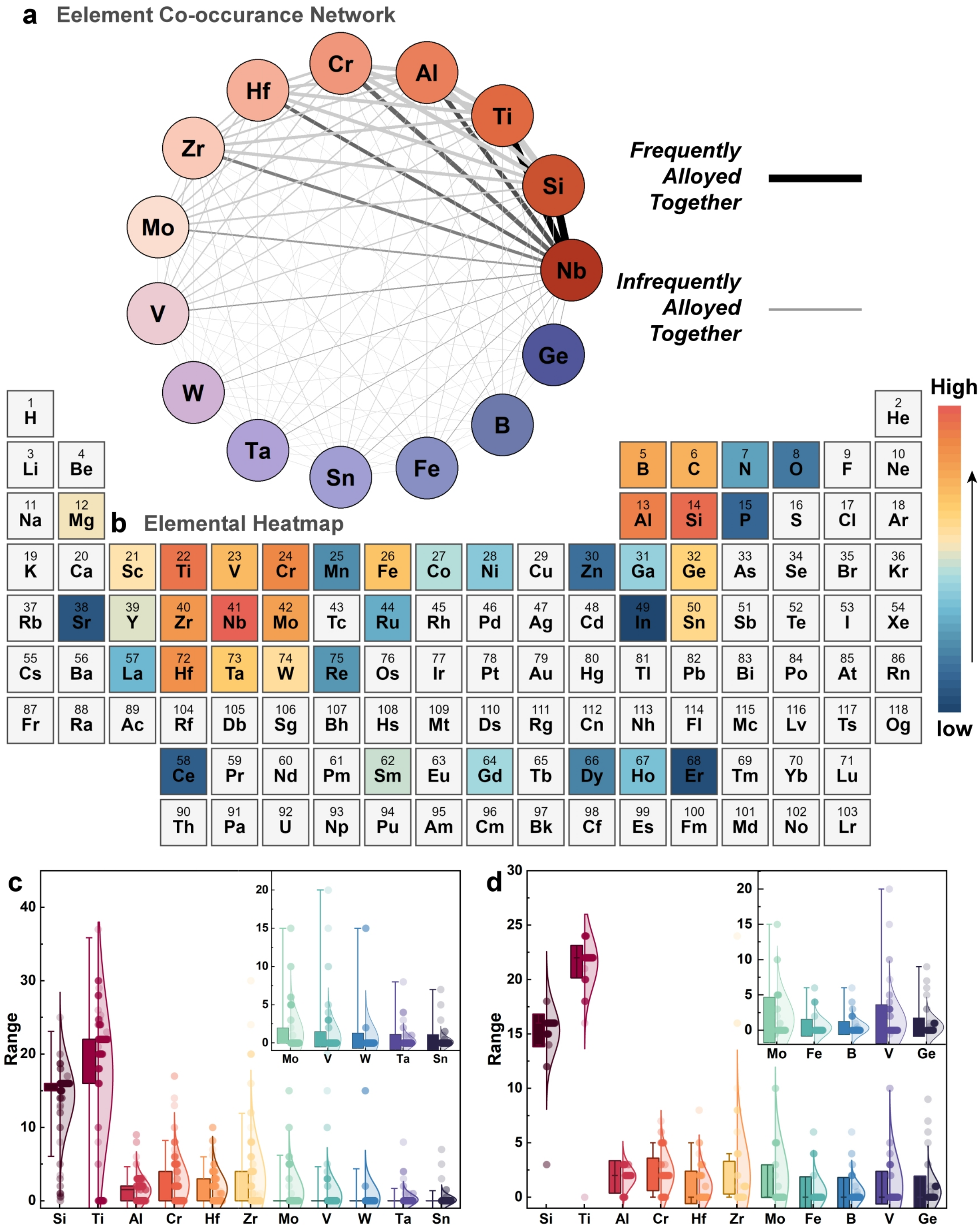


**Supplementary Fig. 1 Dataset feature visualization. a**, Element co-occurrence network with node size representing frequency and line thickness indicating co-occurrence strength. **b**, Element periodic table distribution heat map showing occurrence frequency across all alloys. **c**, **d**, Element content distribution ranges in (**c**) $K_Q$ (n=203) and (**d**) HTC (n=34) datasets with box plots showing median, quartiles, and outliers.

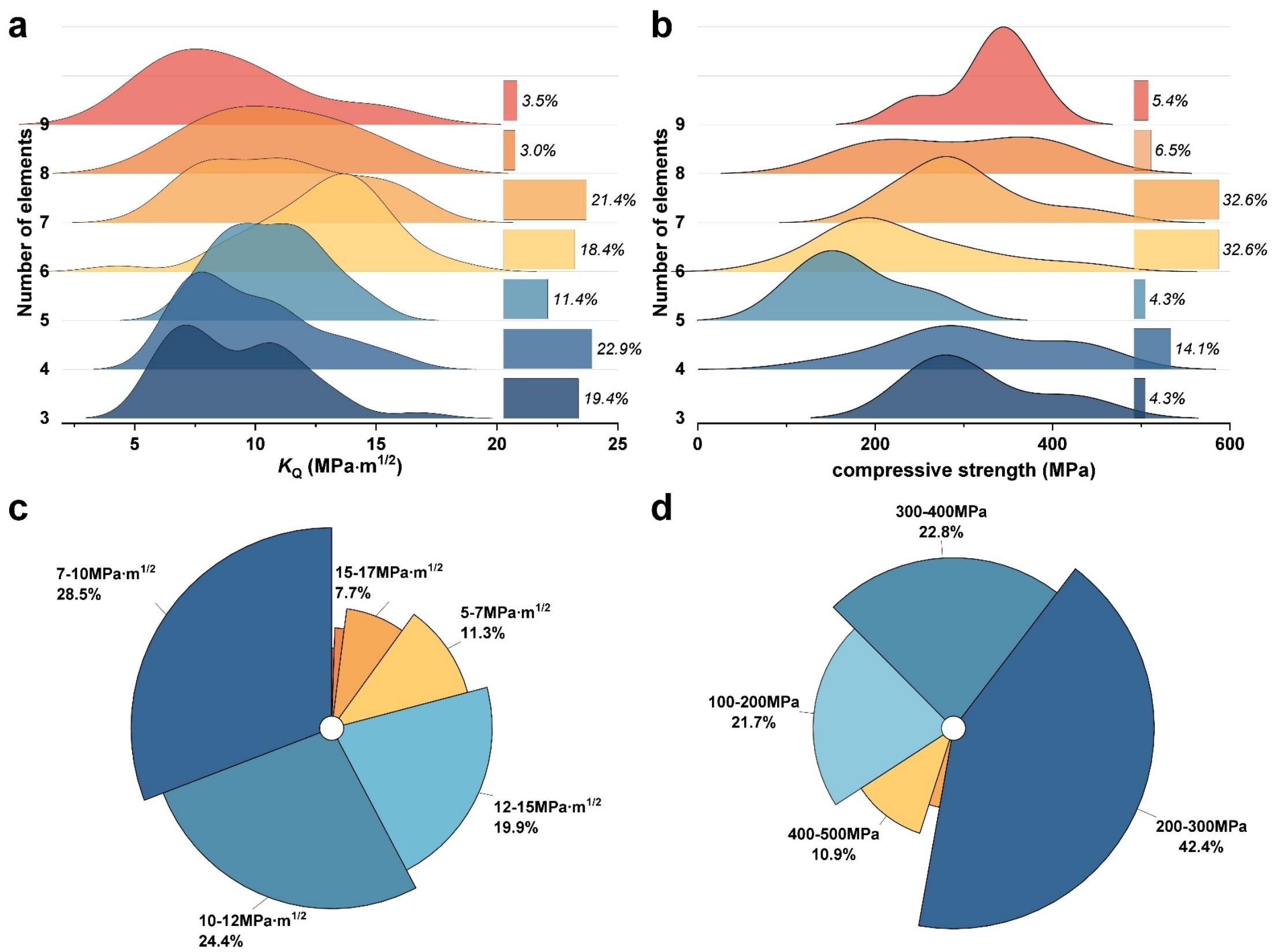


**Supplementary Fig. 2 Data distribution in $K_Q$ and HTC datasets. a**, **b**, Ridgeline plots showing the property distributions of alloys grouped by element number (3–9) for (**a**) $K_Q$ (n = 203) and (**b**) HTC (n = 34) datasets; bar plots on the right indicate the proportion of alloys in each element-number group. **c**, **d**, Pie charts showing the fraction of alloys in each performance interval for (**c**) $K_Q$ ($MPa \cdot m^{1/2}$) and (**d**) HTC (MPa), indicating concentration in medium performance ranges.

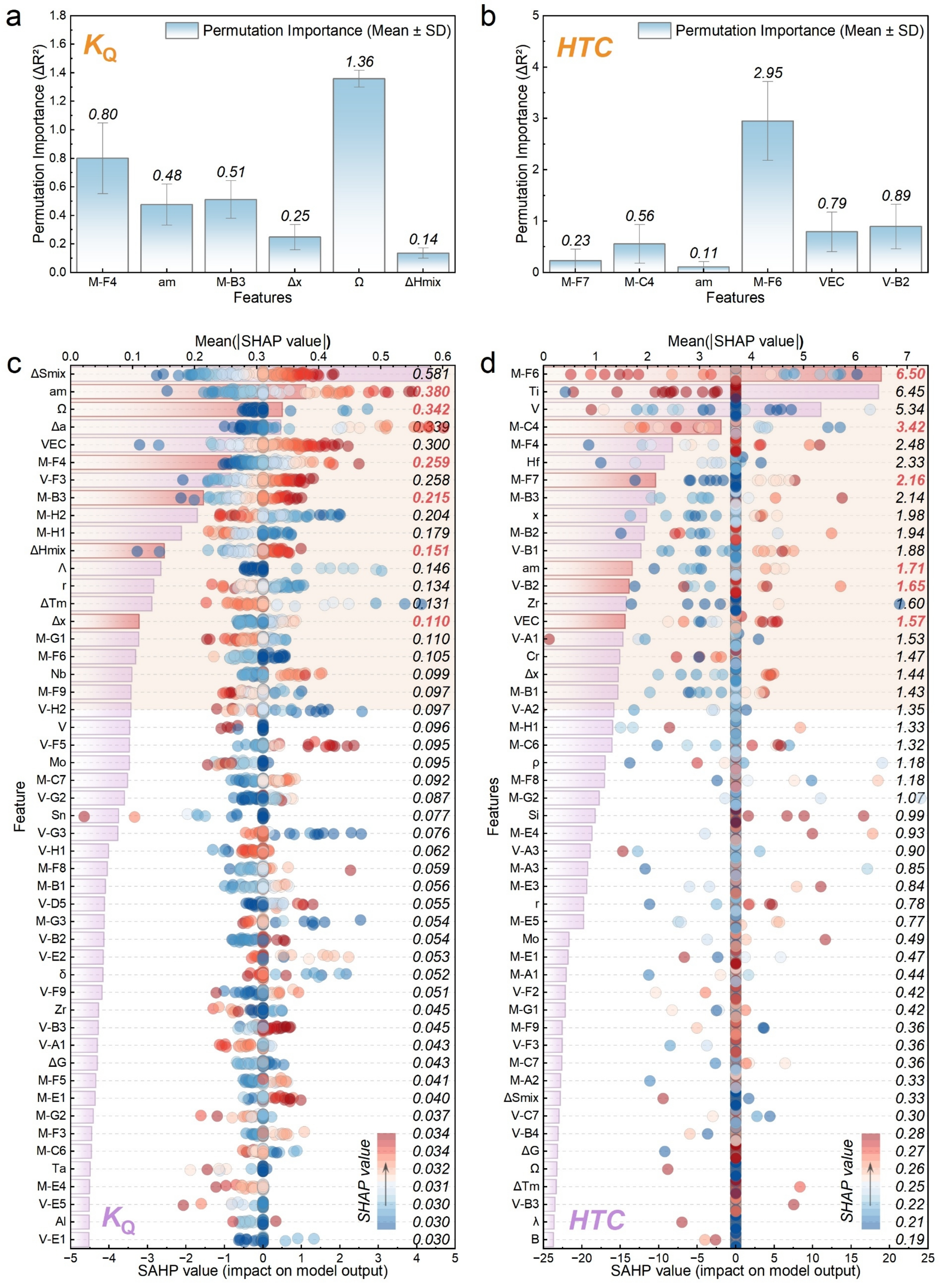


**Supplementary Fig. 3 Independent validation of feature selection**. **a**, **b**, Permutation importance for (**a**) $K_Q$ (n = 203) and (**b**) HTC (n = 34) models; each feature was randomly shuffled 100 times across all samples; bars show mean importance; error bars show SD across 100 permutations; all six selected features show positive importance ($p < 0.001$, t-test). **c**, **d**, SHAP-based feature rankings across the full feature space for (**c**) $K_Q$ (top 50 of 103 features shown) and (**d**) HTC (top 50 of 97 features shown).

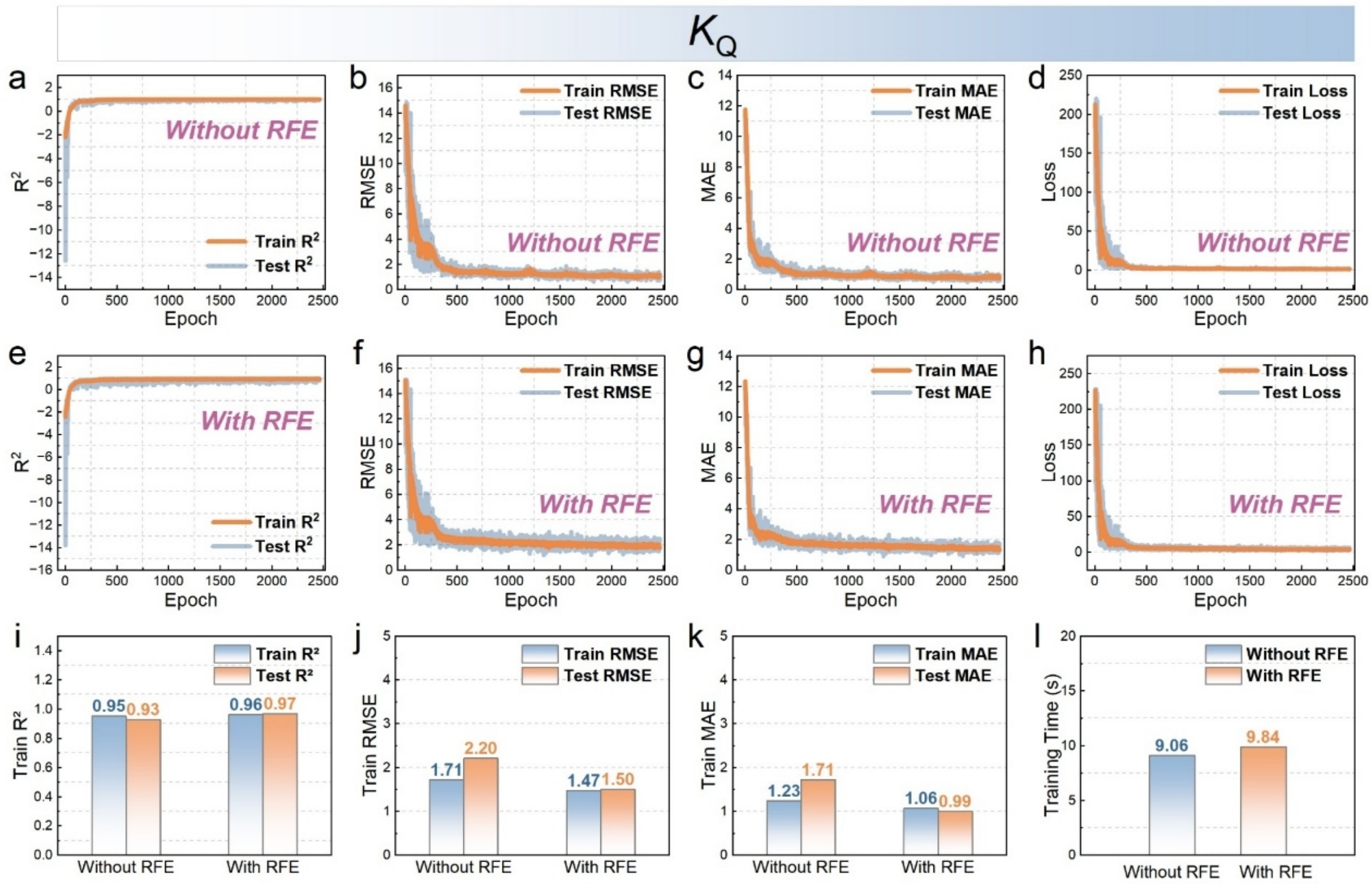


**Supplementary Fig. 4 Model performance comparison with and without RFE for the $K_Q$ model. a-d**, Training and test curves without RFE for (**a**) $R^2$, (**b**) RMSE, (**c**) MAE, and (**d**) loss. **e-h**, Training and test curves with RFE for (**e**) $R^2$, (**f**) RMSE, (**g**) MAE, and (**h**) loss. **i-l**, Final performance metric comparison across models for (**i**) $R^2$, (**j**) RMSE, (**k**) MAE, and (**l**) loss. All evaluations are based on n = 203 independent arc-melted as-cast alloy samples.

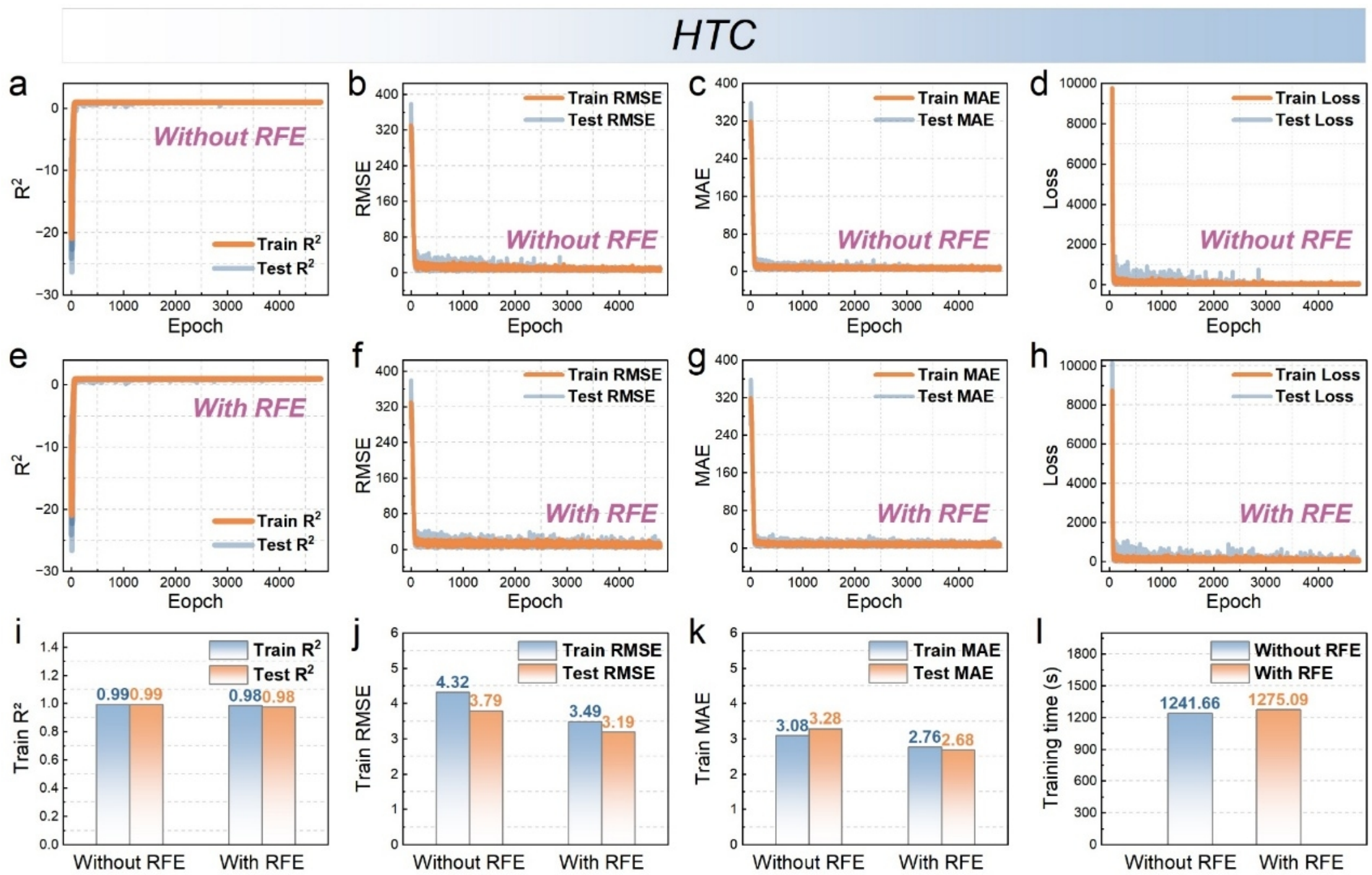


**Supplementary Fig. 5 Model performance comparison with and without RFE for the HTC model. a-d**, Training and test curves without RFE for (**a**) $R^2$, (**b**) RMSE, (**c**) MAE, and (**d**) loss. **e-h**, Training and test curves with RFE for (**e**) $R^2$, (**f**) RMSE, (**g**) MAE, and (**h**) loss. **i-l**, Final performance metric comparison across models for (**i**) $R^2$, (**j**) RMSE, (**k**) MAE, and (**l**) loss. All evaluations are based on n = 34 independent as-cast alloy samples tested at 1250°C.

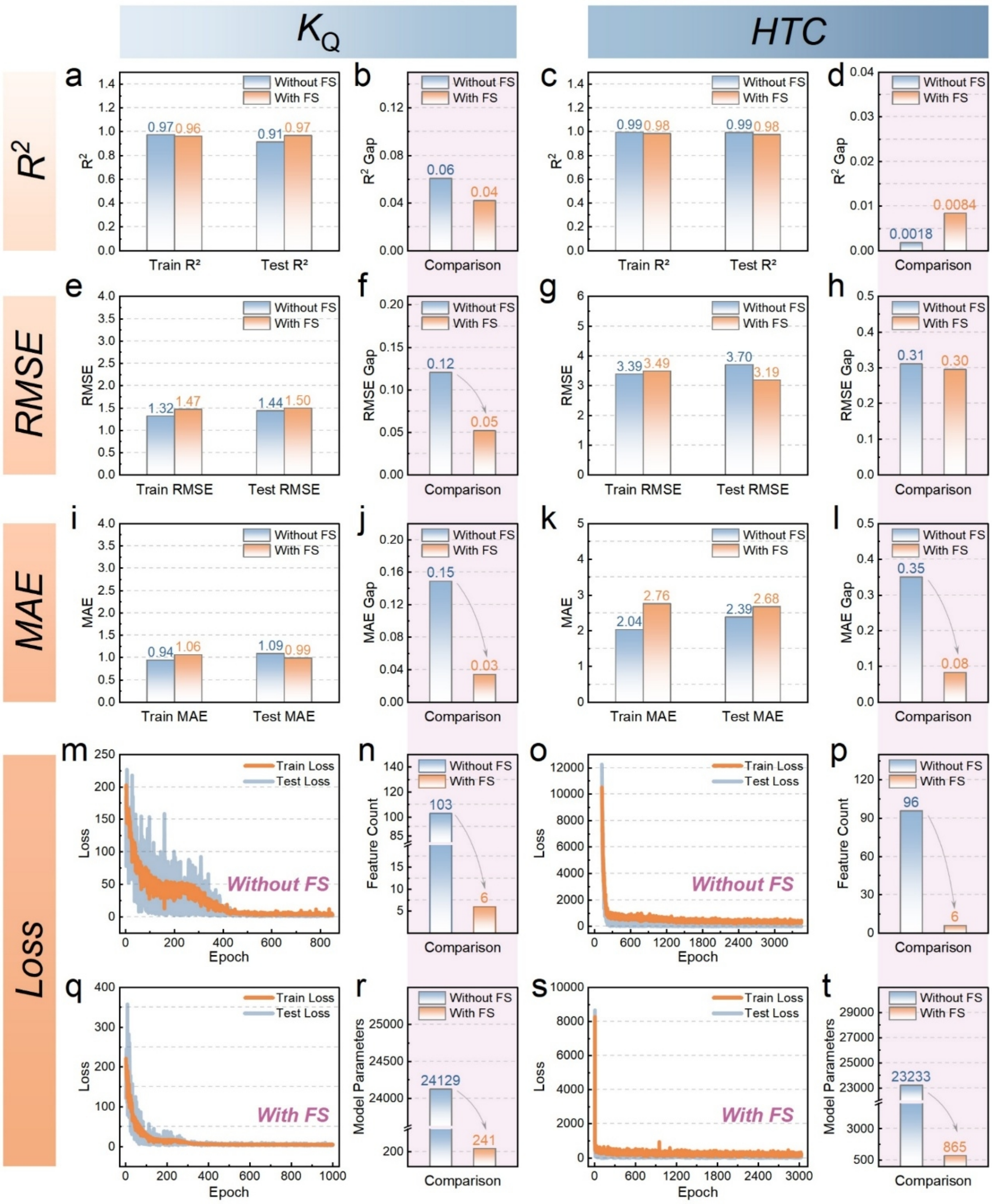


**Supplementary Fig. 6 Impact of feature selection on model performance.** Performance comparison for $K_Q$ (**a**, **b**, **e**, **f**, **i**, **j**, **m**, **n**, **q**, **r**) and HTC (**c**, **d**, **g**, **h**, **k**, **l**, **o**, **p**, **s**, **t**) models with and without feature selection: (**a**, **c**) training/test $R^2$; (**b**, **d**) $R^2$ gap between training and test; (**e**, **g**) training/test RMSE; (**f**, **h**) RMSE gap; (**i**, **k**) training/test MAE; (**j**, **l**) MAE gap; (**m**, **o**, **q**, **s**) loss curves; (**n**, **p**) feature count used; (**r**, **t**) total model parameters. $K_Q$ model: n = 203; HTC model: n = 34.

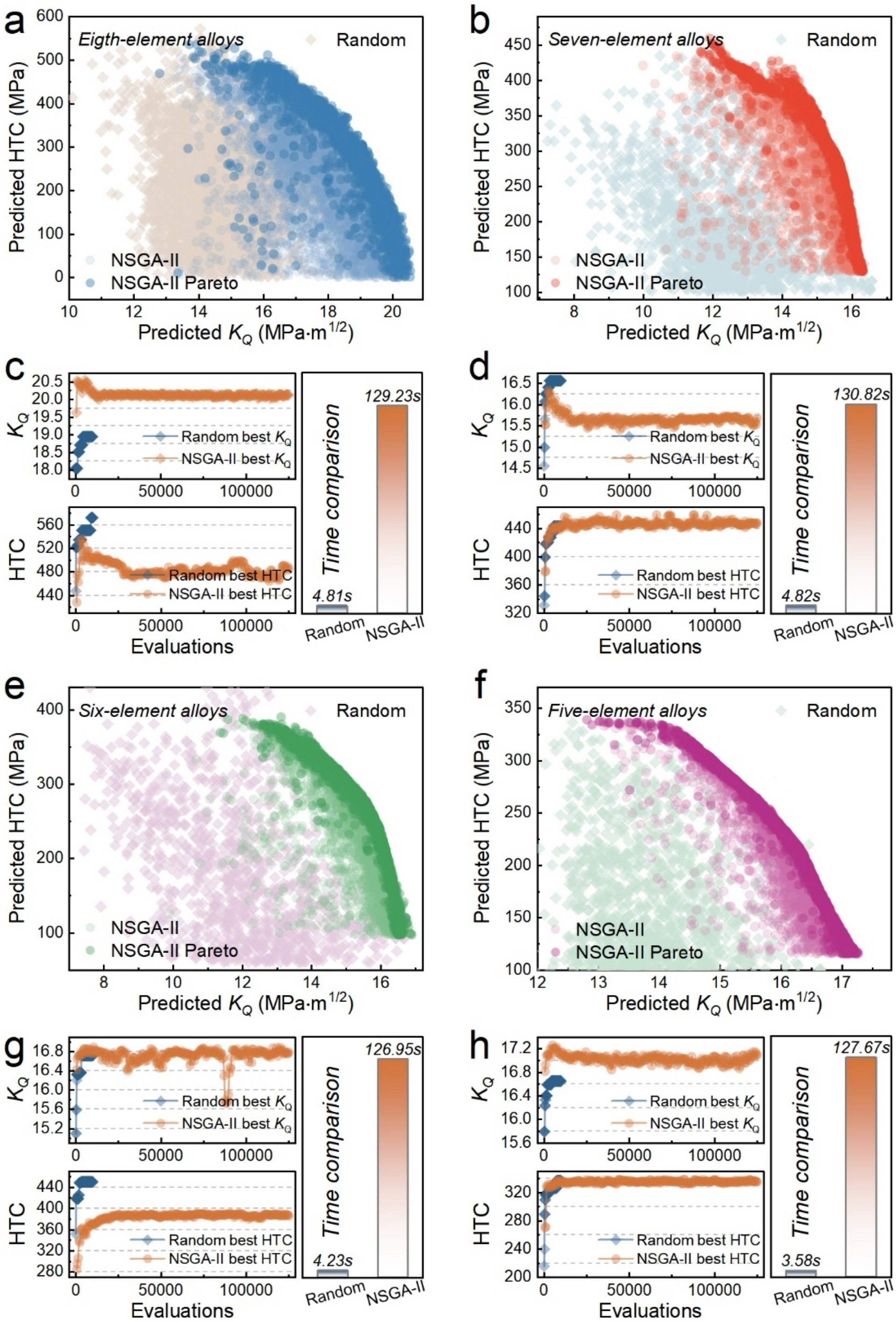


**Supplementary Fig. 7 NSGA-II versus random search for multi-objective optimization. a**, **b**, **e**, **f**, Predicted $K_Q$ versus HTC scatter plots for (**a**) 8-element, (**b**) 7-element, (**e**) 6-element, and (**f**) 5-element alloy systems, comparing random search evaluations, NSGA-II evaluations, and identified Pareto fronts. **c**, **d**, **g**, **h**, Convergence curves of best $K_Q$ and HTC values and computational time per generation for (**c**) 8-element, (**d**) 7-element, (**g**) 6-element, and (**h**) 5-element systems.

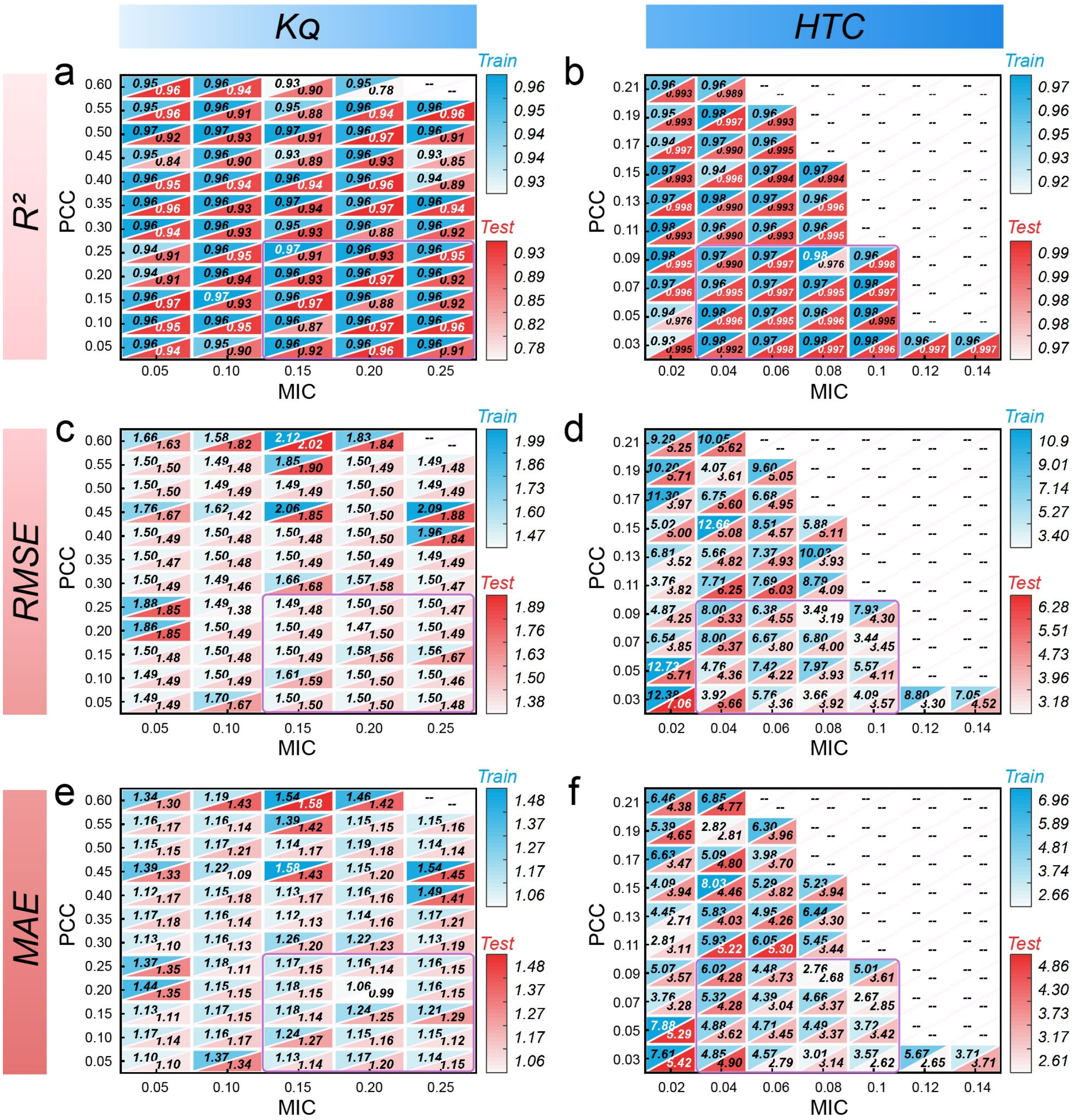


**Supplementary Fig. 8 Grid search results across the PCC-MIC threshold space. a**, **c**, **e**, $K_Q$ model results: (**a**) test $R^2$, (**c**) test RMSE, and (**e**) test MAE across all threshold combinations. **b**, **d**, f, HTC model results: (**b**) test $R^2$, (**d**) test RMSE, and (**f**) test MAE. White regions indicate threshold combinations where insufficient features remained after filtering; combinations beyond the displayed ranges yielded no valid models.

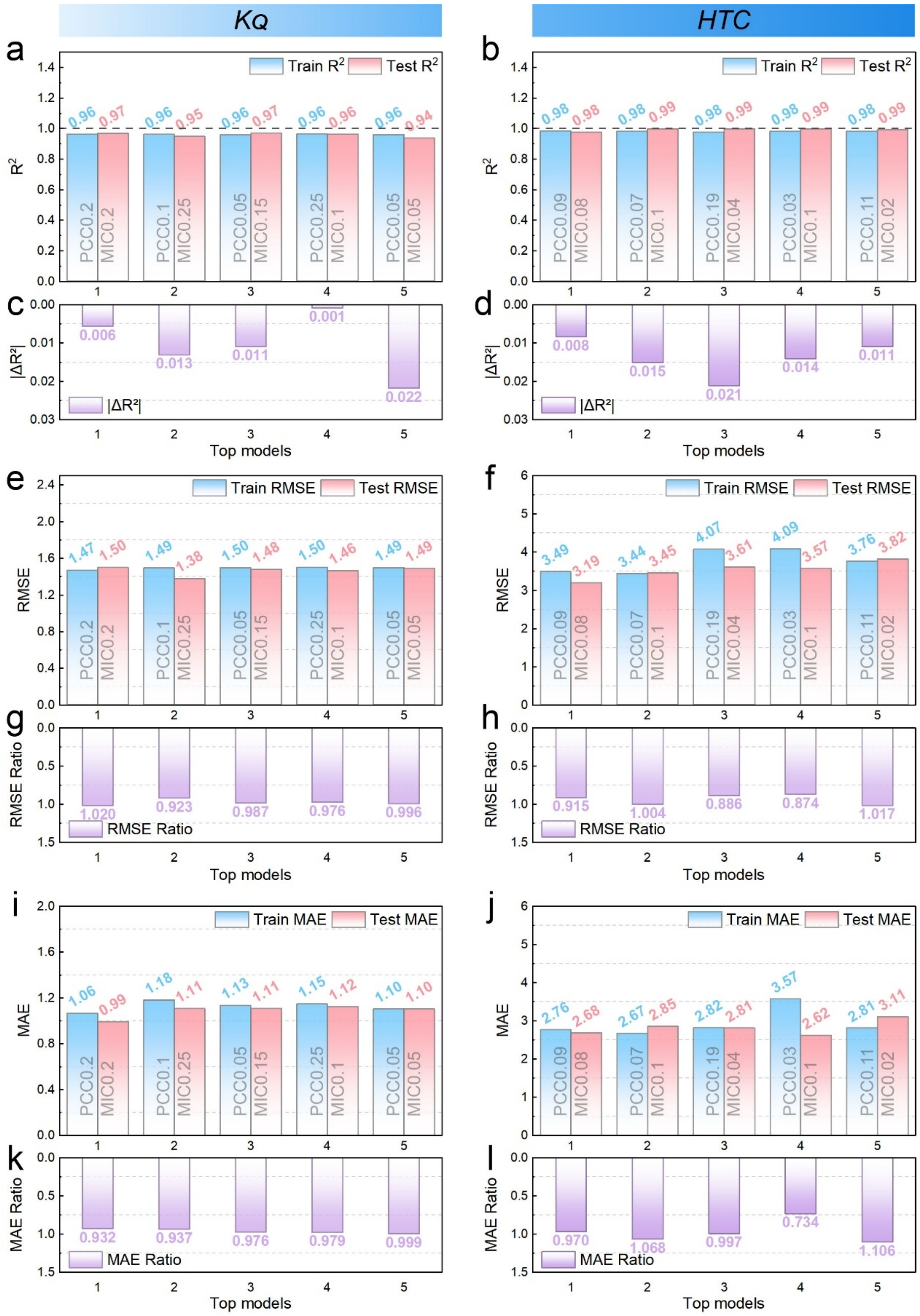


**Supplementary Fig. 9 Comprehensive performance comparison of top 5 threshold combinations.** **a**, **b**, Training and test $R^2$ values. **c**, **d**, Training and test RMSE values. **e**, **f**, Training and test MAE values. **g**, **h**, Absolute difference between training and test $R^2$. **i**, **j**, Ratio of test RMSE to training RMSE. **k**, **l**, Ratio of test MAE to training MAE. Left column (**a**, **c**, **e**, **g**, **i**, **k**): $K_Q$ model (n = 203); right column (**b**, **d**, **f**, **h**, **j**, **l**): HTC model (n = 34).

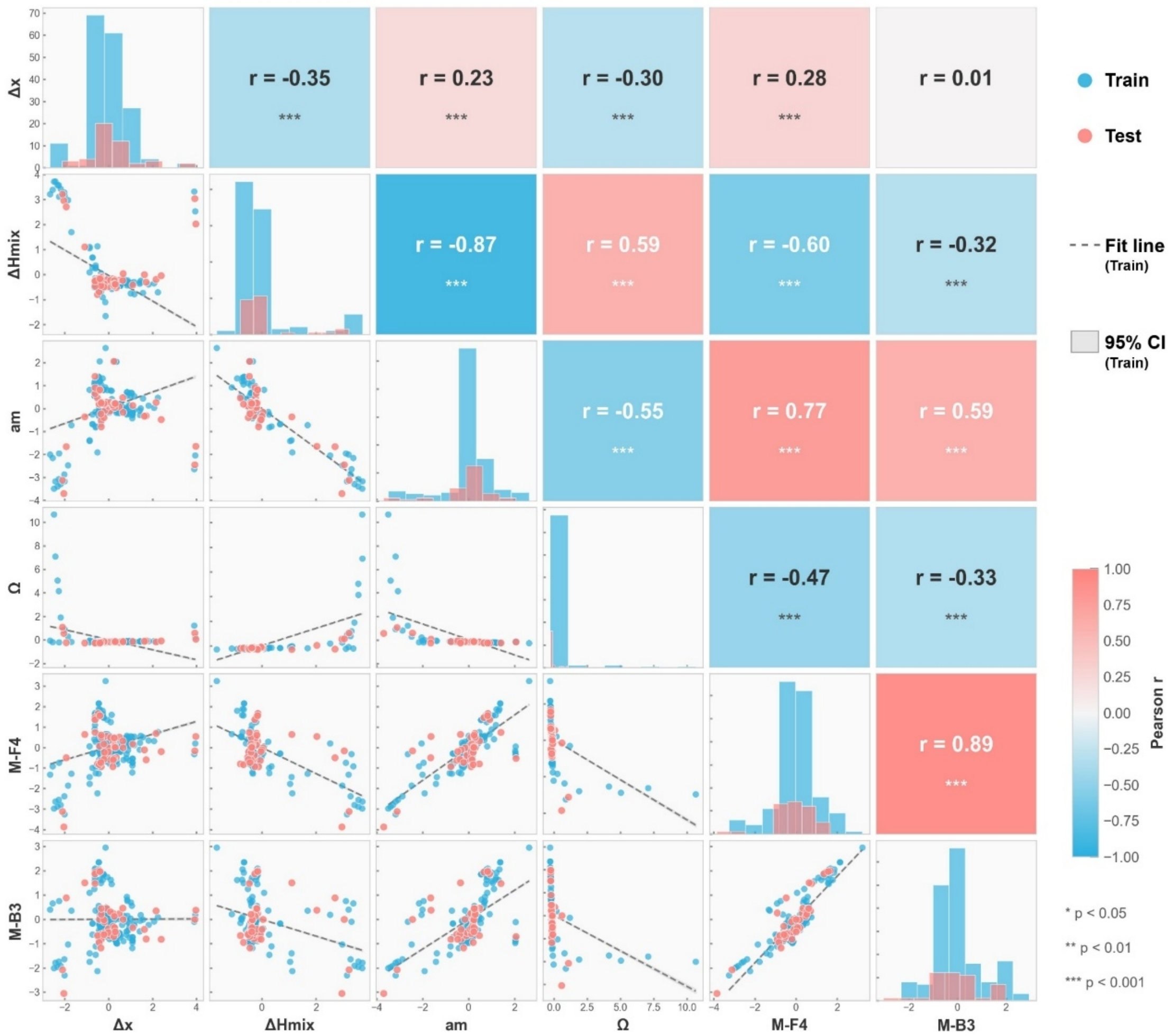


**Supplementary Fig. 10 Feature distribution comparison for the $K_Q$ model.** Histograms comparing the distributions of six selected features between the training set (n = 162) and the test set (n = 41) for M-F4, am, M-B3, Δχ, Ω, and ΔHmix. The consistent overlap across all features confirms good representativeness of the data split without distribution shift.

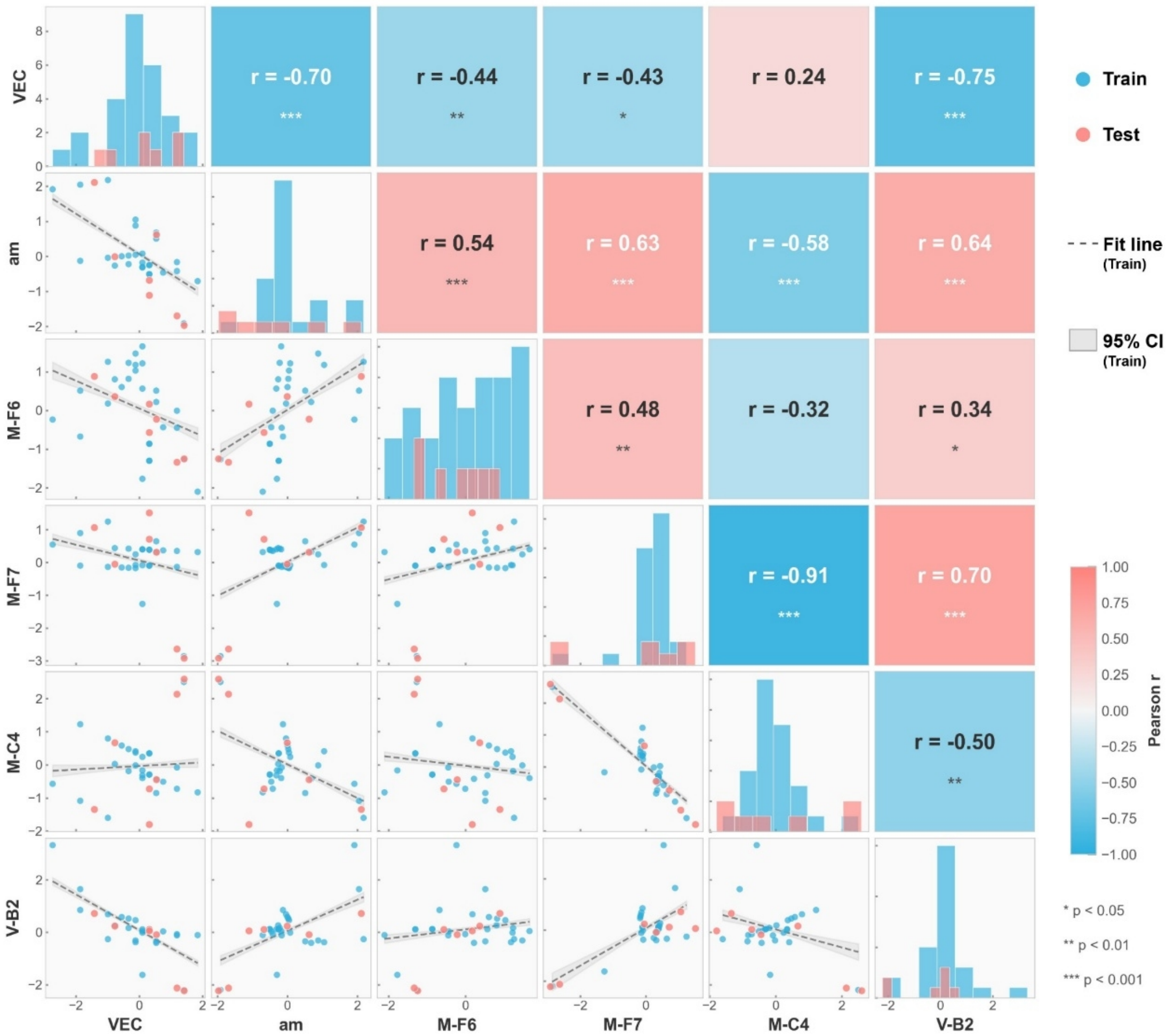


**Supplementary Fig. 11 Feature distribution comparison for the HTC model.** Histograms comparing the distributions of six selected features between the training set (n = 27) and the test set (n = 7) for M-F7, M-C4, am, M-F6, VEC, and V-B2. Consistent coverage across datasets confirms adequate representativeness despite the limited sample size.

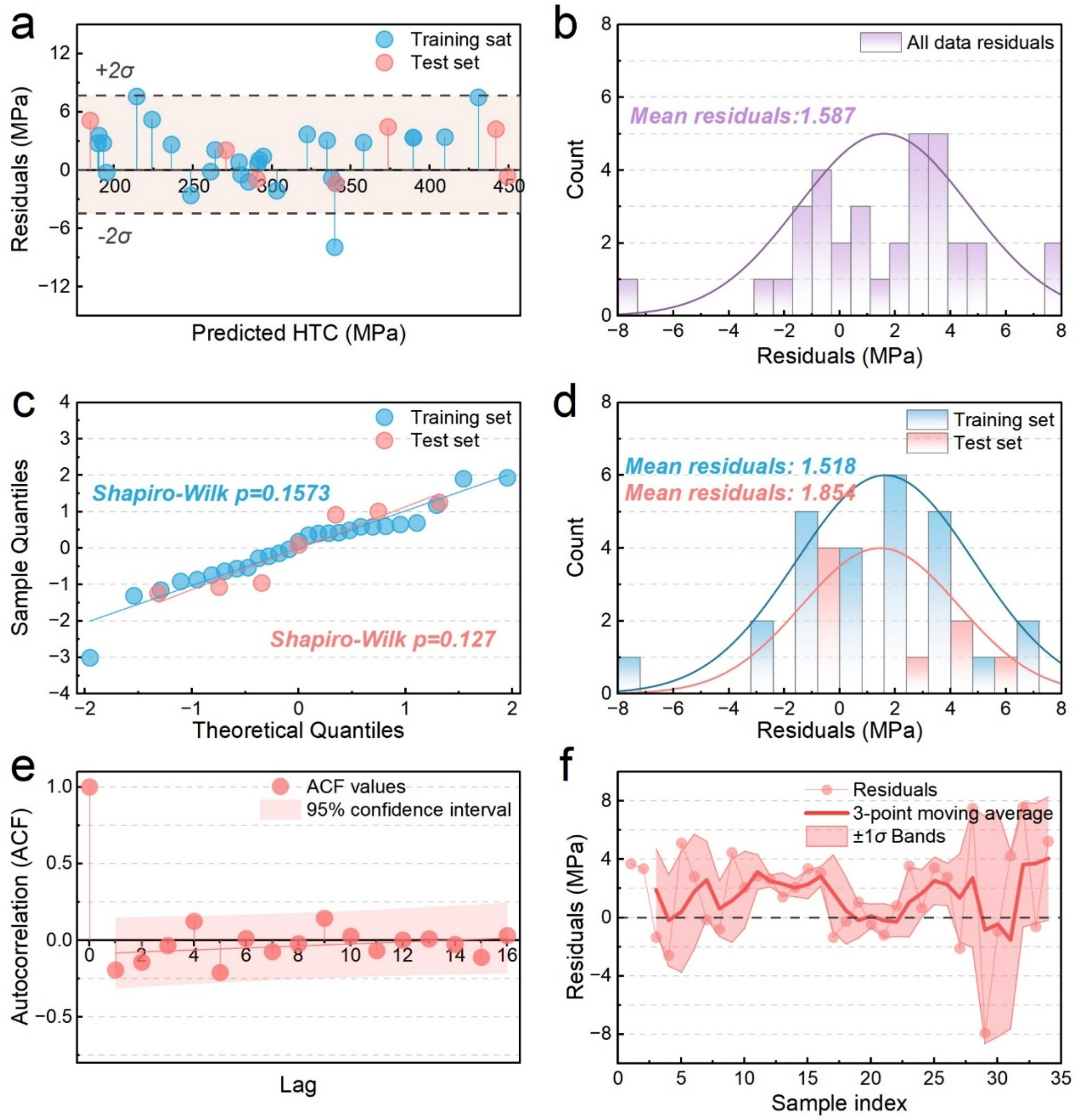


**Supplementary Fig. 12 HTC model residual diagnostics.** **a**, Residuals versus predicted HTC values with ±2$\sigma$ bounds (n = 34). **b**, Overall residual histogram with normal distribution fit. **c**, Q–Q plot of standardized residuals against theoretical normal quantiles. **d**, Training (n = 27) and test (n = 7) residual histograms shown separately. **e**, Autocorrelation function of residuals with 95% confidence interval; no significant autocorrelation detected. **f**, Residuals versus sample index with 3-point moving average and ±1$\sigma$ bands confirming absence of systematic bias.

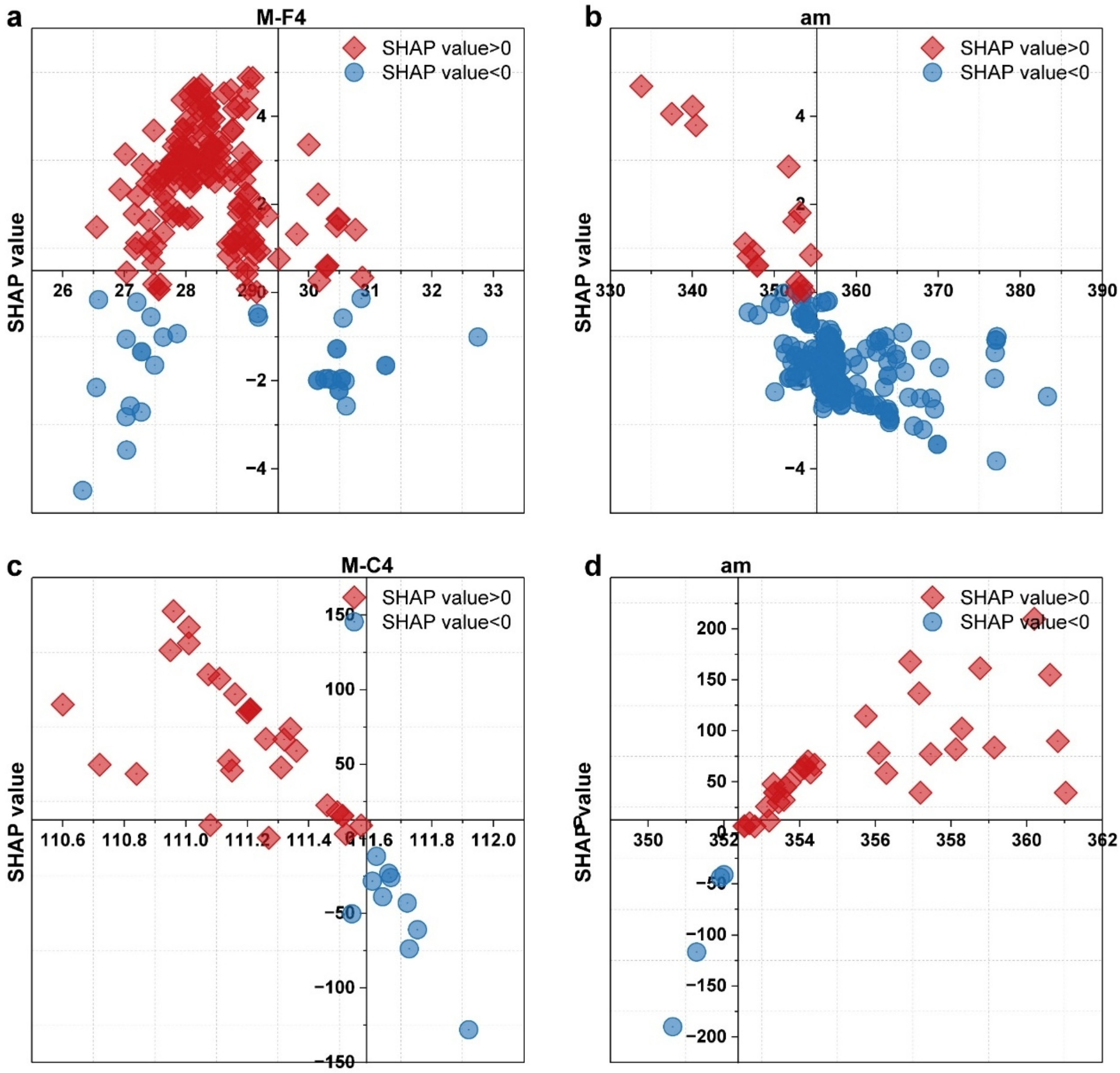


**Supplementary Fig. 13 SHAP dependence plots for key features. a**, **b**, SHAP value versus feature value for the $K_Q$ model: (**a**) M-F4; (**b**) am. **c**, **d**, SHAP value versus feature value for the HTC model: (**c**) M-C4 and (**d**) am. Each point represents one alloy sample; $K_Q$ model: n = 203; HTC model: n = 34.

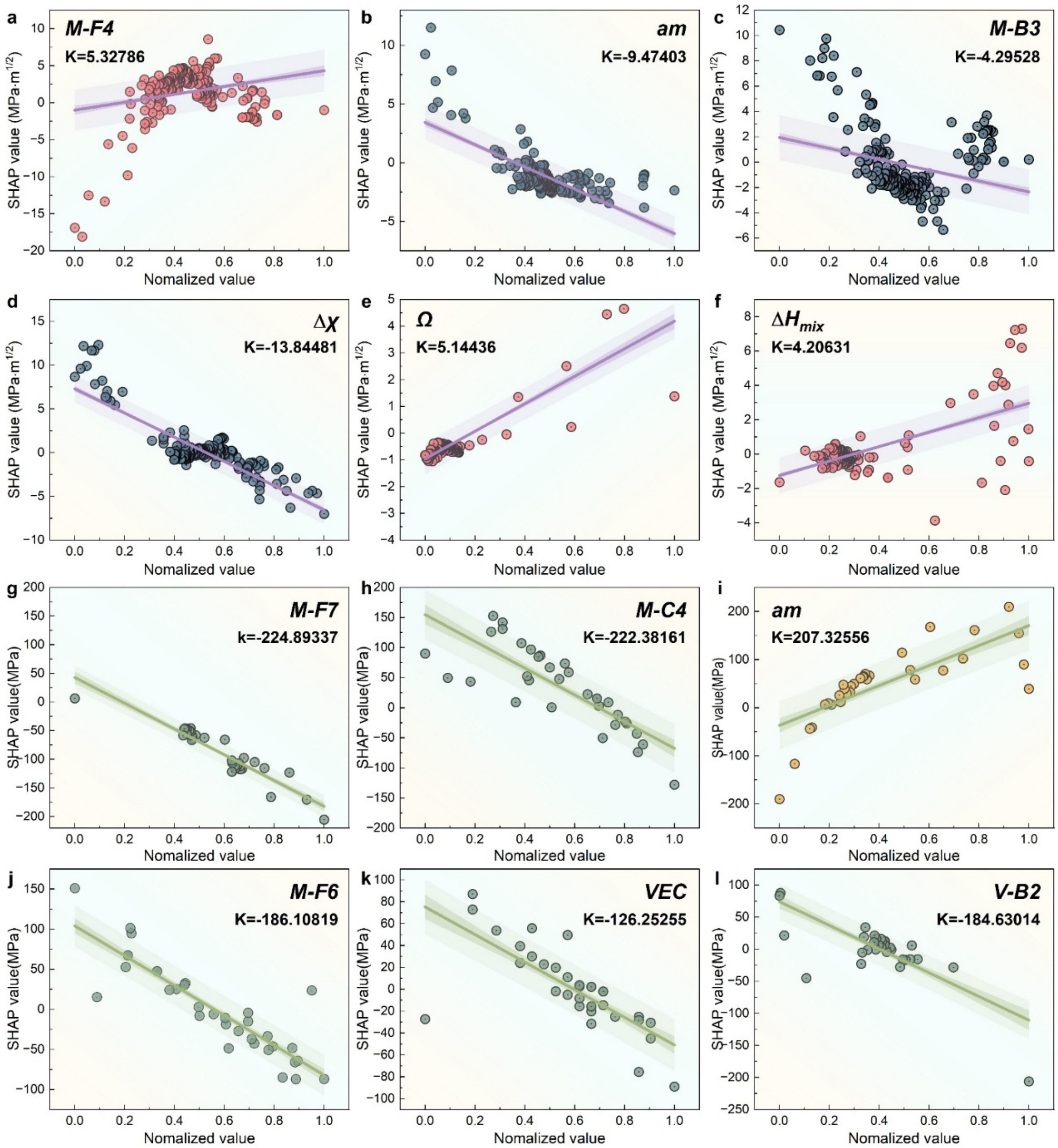


**Supplementary Fig. 14 Normalized alloy factors and corresponding SHAP values. a-f**, $K_Q$-related features: (**a**) M-F4, (**b**) am, (**c**) M-B3, (**d**) Δχ, (**e**) Ω, and (**f**) ΔHmix; scatter plots of normalized feature values versus SHAP values with linear regression lines. **g-l**, HTC-related features: (**g**) M-F7, (**h**) M-C4, (**i**) am, (**j**) M-F6, (**k**) VEC, and (**l**) V-B2. Slope of each regression line gives the influence coefficient $k_{ni}$ ; positive slope indicates feature–property positive correlation. $K_Q$ model: n = 203; HTC model: n = 34.

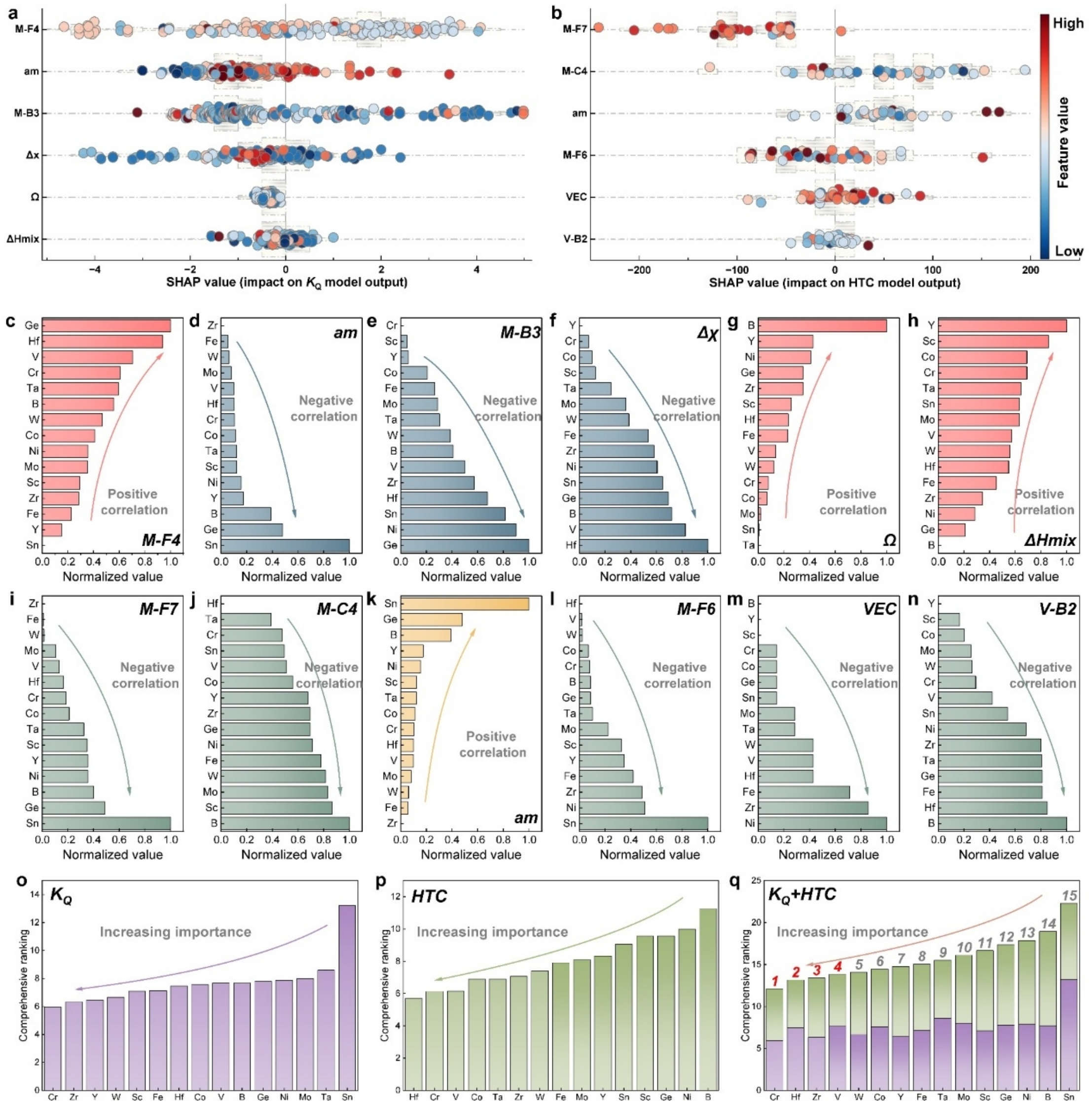

**Supplementary Fig. 15 SHAP analysis and element rank. a**, **b**, SHAP beeswarm plots showing the distribution of SHAP values for six key alloy factors in (**a**) $K_Q$ (n = 203) and (**b**) HTC (n = 34) models. **c-h**, Element importance rankings for $K_Q$ based on individual features: (**c**) M-F4, (**d**) am, (**e**) M-B3, (**f**) Δχ, (**g**) Ω, and (**h**) ΔHmix. **i-n**, Element importance rankings for HTC based on individual features: (**i**) M-F7, (**j**) M-C4, (**k**) am, (**l**) M-F6, (**m**) VEC, and (**n**) V-B2. **o-q**, Weighted element rankings for (**o**) $K_Q$, (**p**) HTC, and (**q**) combined $K_Q$ +HTC optimization.

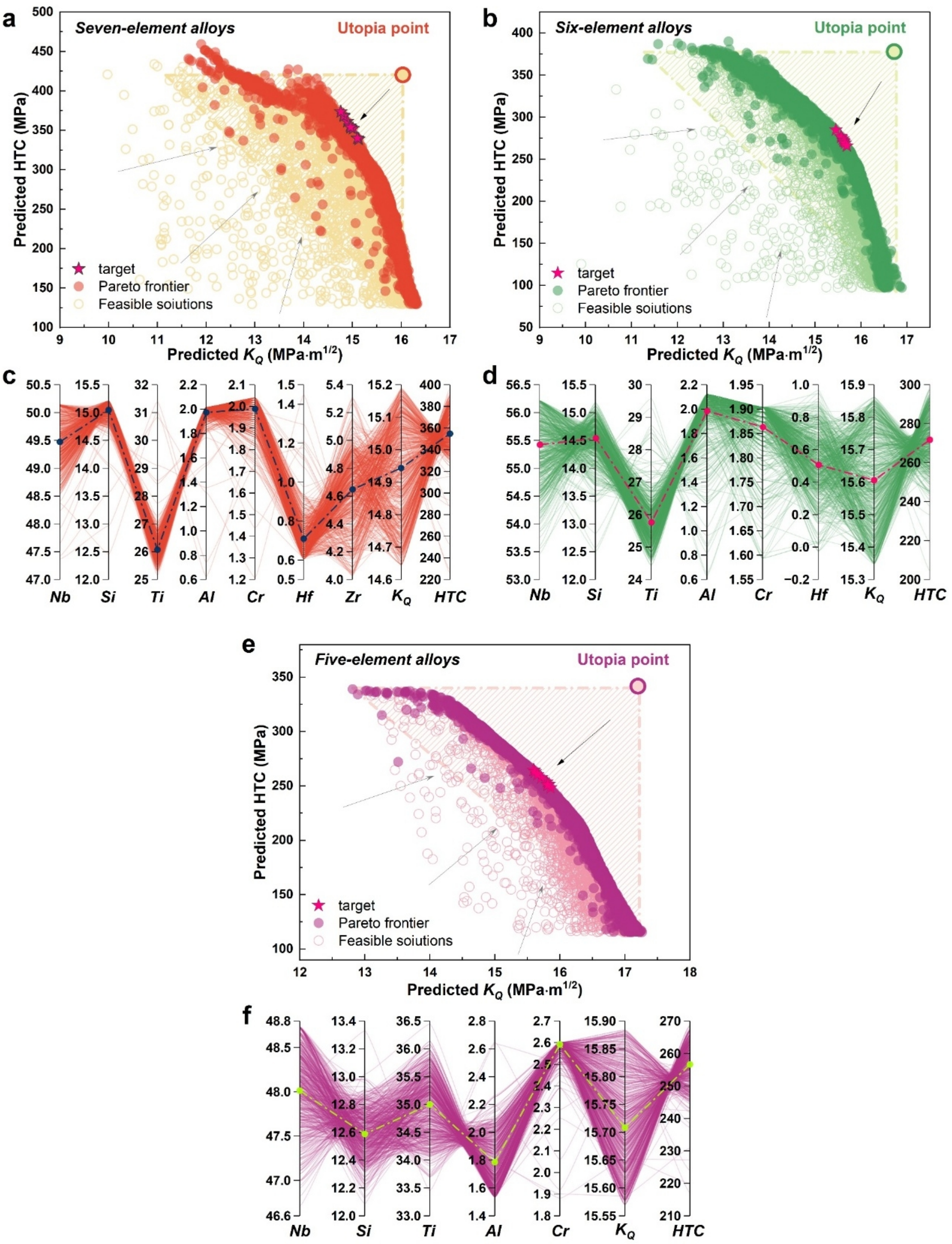


**Supplementary Fig. 16 Multi-objective optimization results for 7-, 6-, and 5-element alloy systems. a**, **b**, **e**, Pareto front diagrams for (**a**) 7-element, (**b**) 6-element, and (**e**) 5-element alloy systems. **c**, **d**, **f**, Corresponding parallel coordinate plots of Pareto-optimal alloy compositions showing composition variation along Pareto fronts for (**c**) 7-element, (**d**) 6-element, and (**f**) 5-element systems.

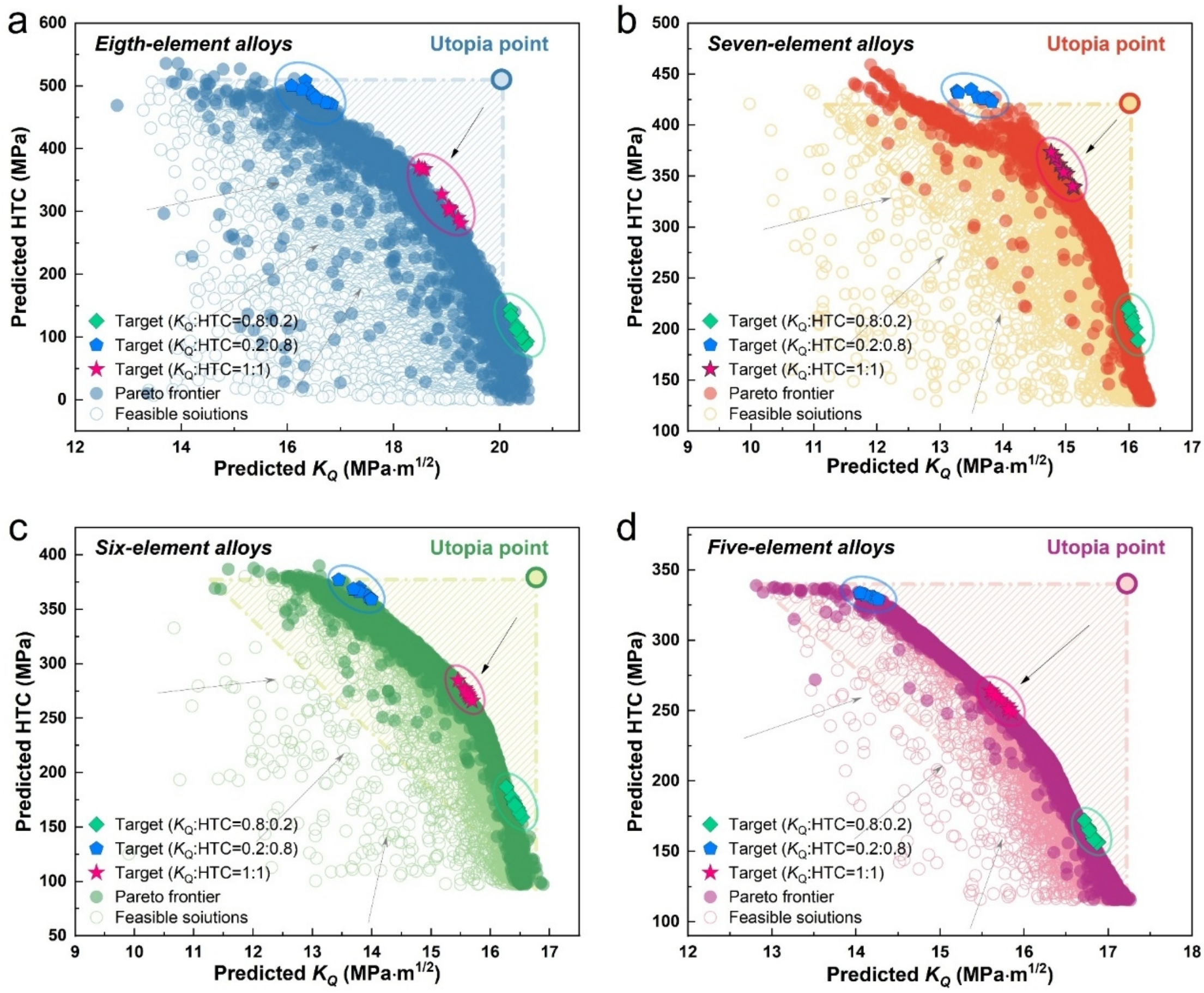


**Supplementary Fig. 17. Sensitivity analysis of Pareto optimization to weighting schemes. a-d**, Pareto frontier scatter plots for (**a**) 8-element, (**b**) 7-element, (**c**) 6-element, and (**d**) 5-element alloy systems under three $K_Q$:HTC weighting schemes; green diamond: recommended alloy at $K_Q$:HTC = 0.8:0.2; blue circle: recommended alloy at $K_Q$:HTC = 0.2:0.8; pink star: recommended alloy at $K_Q$:HTC = 1:1; open circle: utopia point. Arrows show the direction from the utopia point to each recommended alloy.

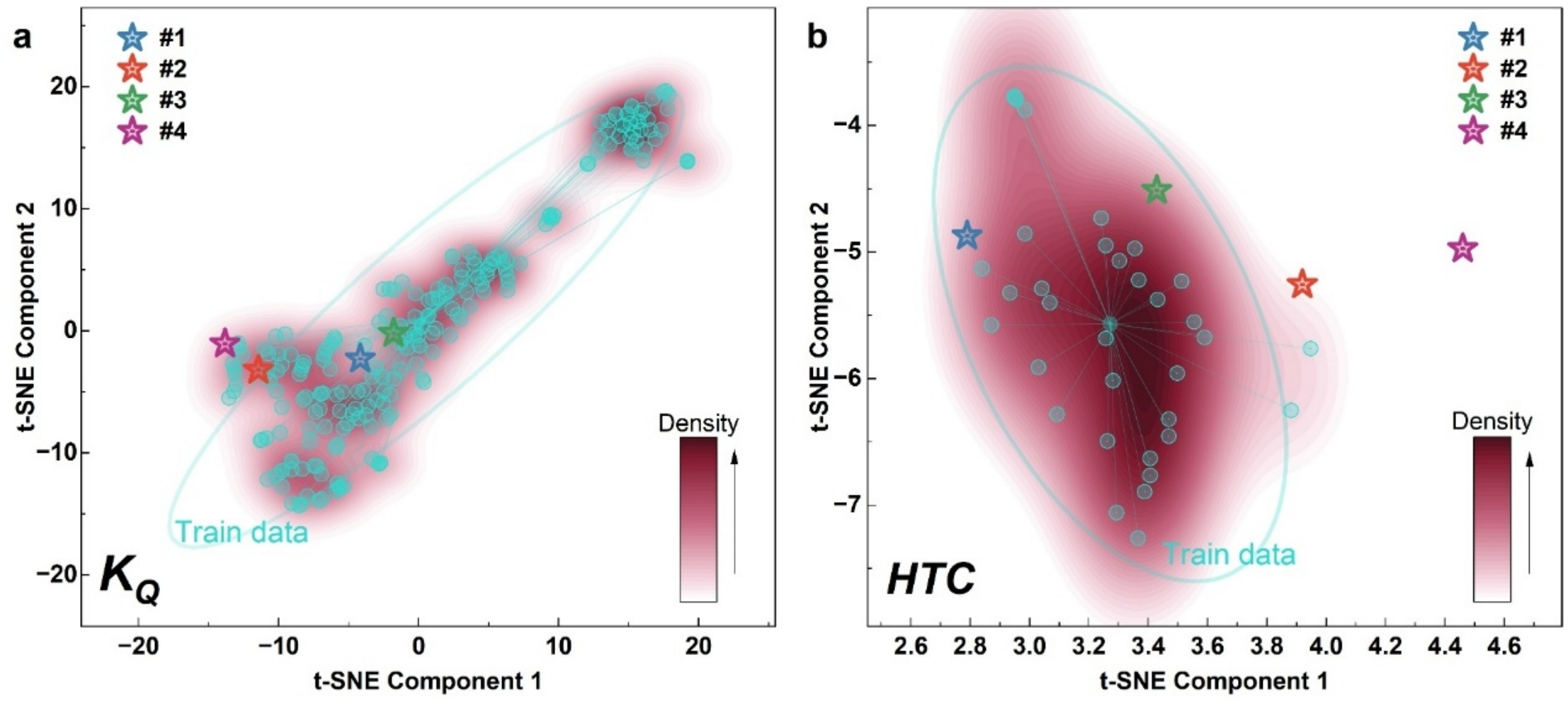


**Supplementary Fig. 18 t-SNE extrapolation analysis of optimized compositions. a**, **b**, t-SNE projections of the composition space for (**a**) $K_Q$ (training data: n = 203) and (**b**) HTC (training data: n = 34) datasets.

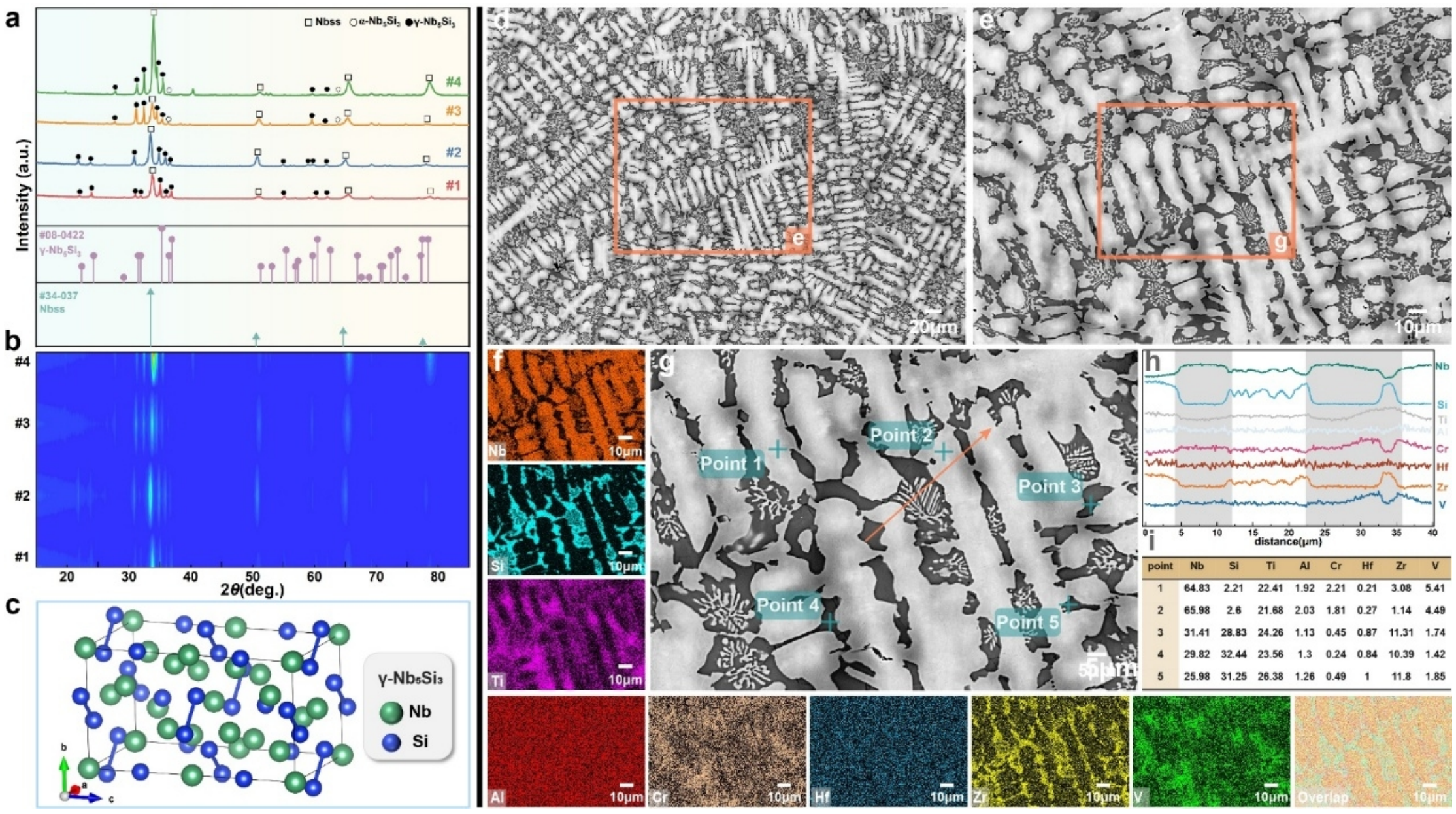


**Supplementary Fig. 19 Microstructure of alloy #1. a**, **b**, XRD patterns. **c**, Crystal structure model of γ-$Nb_5Si_3$ (hexagonal D8m). **d**, **e**, **g**, SEM images (BSE, 20 kV) showing the typical dendritic microstructure at different magnifications. **f**, **h**, **i**, EDS elemental maps and line scans revealing Si enrichment in the gray γ-$Nb_5Si_3$ phase and Nb enrichment in the Nbss matrix.

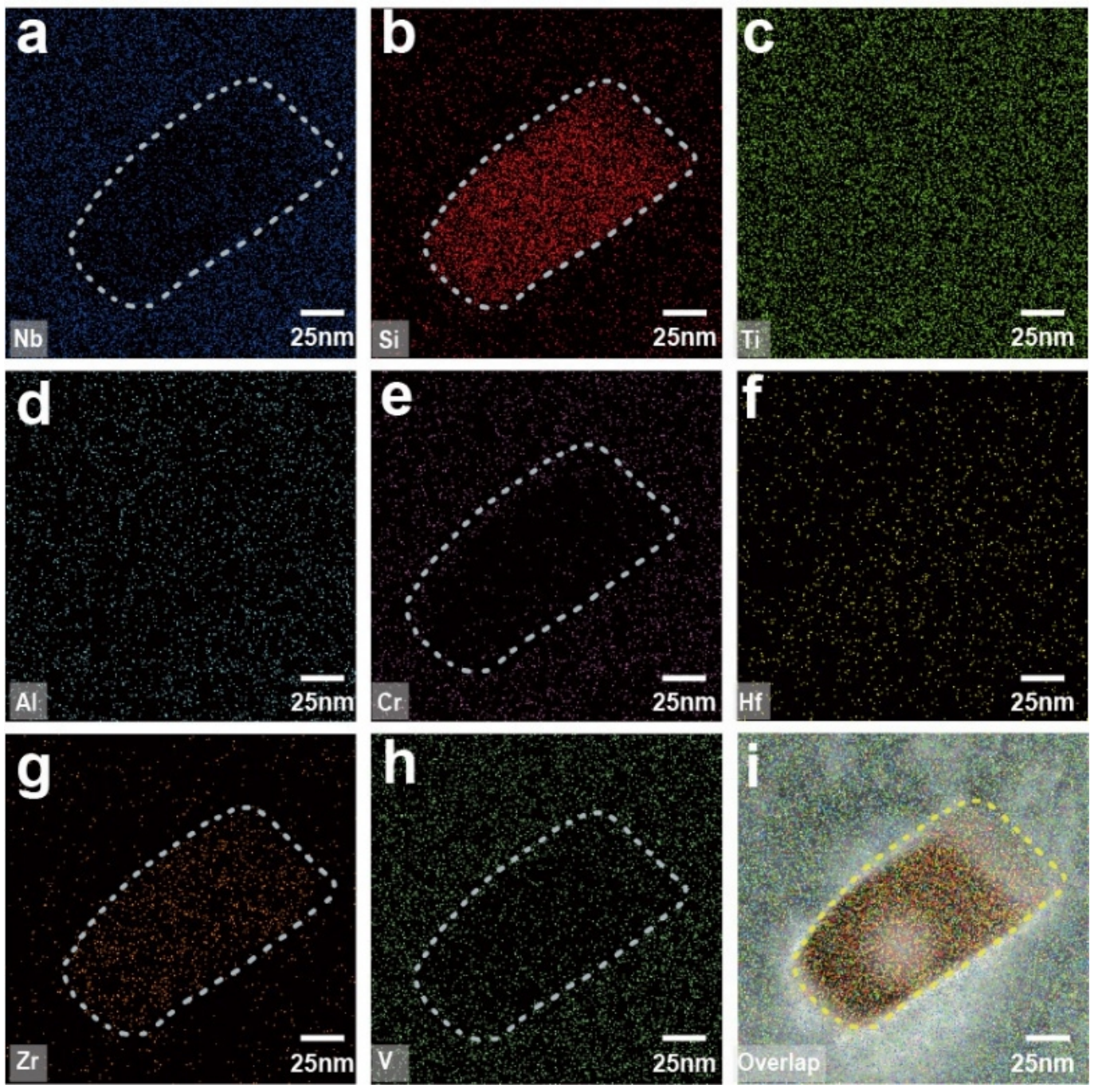


**Supplementary Fig. 20 EDS elemental maps of the γ′-$Nb_5Si_3$ nanoparticle in Fig. 7c**. **a-h**, Elemental distribution maps: (**a**) Nb, (**b**) Si, (**c**) Ti, (**d**) Al, (**e**) Cr, (**f**) Hf, (**g**) Zr, and (**h**) V. **i**, Overlay map of all elements.

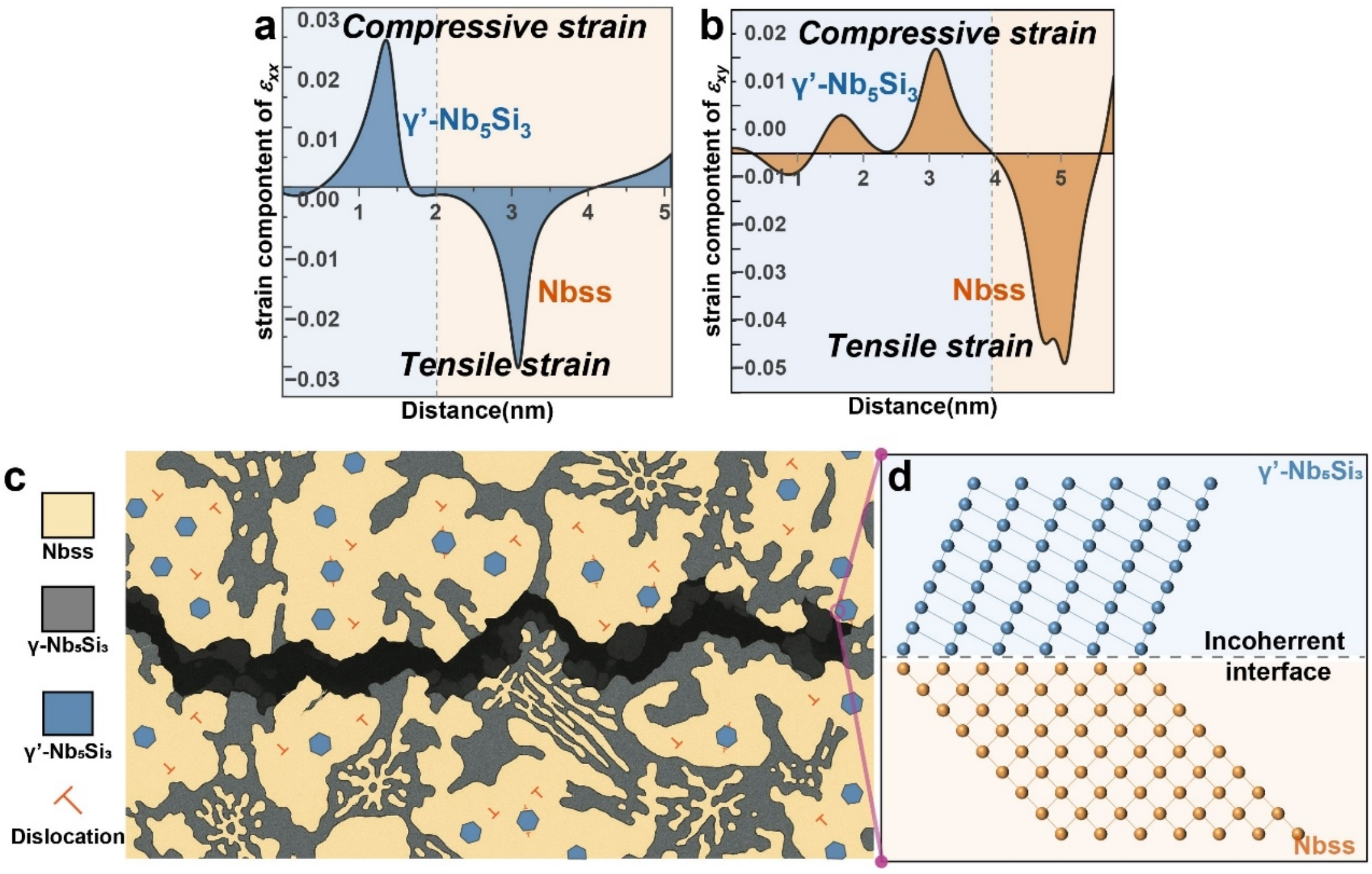


**Supplementary Fig. 21 Interfacial strain profiles and toughening mechanism schematics. a**, **b**, Quantitative strain profiles extracted from GPA analysis (Fig. 7**h** and 7**n**) across the Nbss/γ′-$Nb_5Si_3$ interface for (**a**) $\varepsilon_{xx}$ and (**b**) $\varepsilon_{xy}$ components, showing 6–8% strain gradients on either side of the interface. **c**, **d**, Schematic diagrams illustrating (**c**) the dispersed nanoscale γ′-$Nb_5Si_3$ distribution within the Nbss matrix and (**d**) the incoherent interface structure arising from 67.6% lattice mismatch.

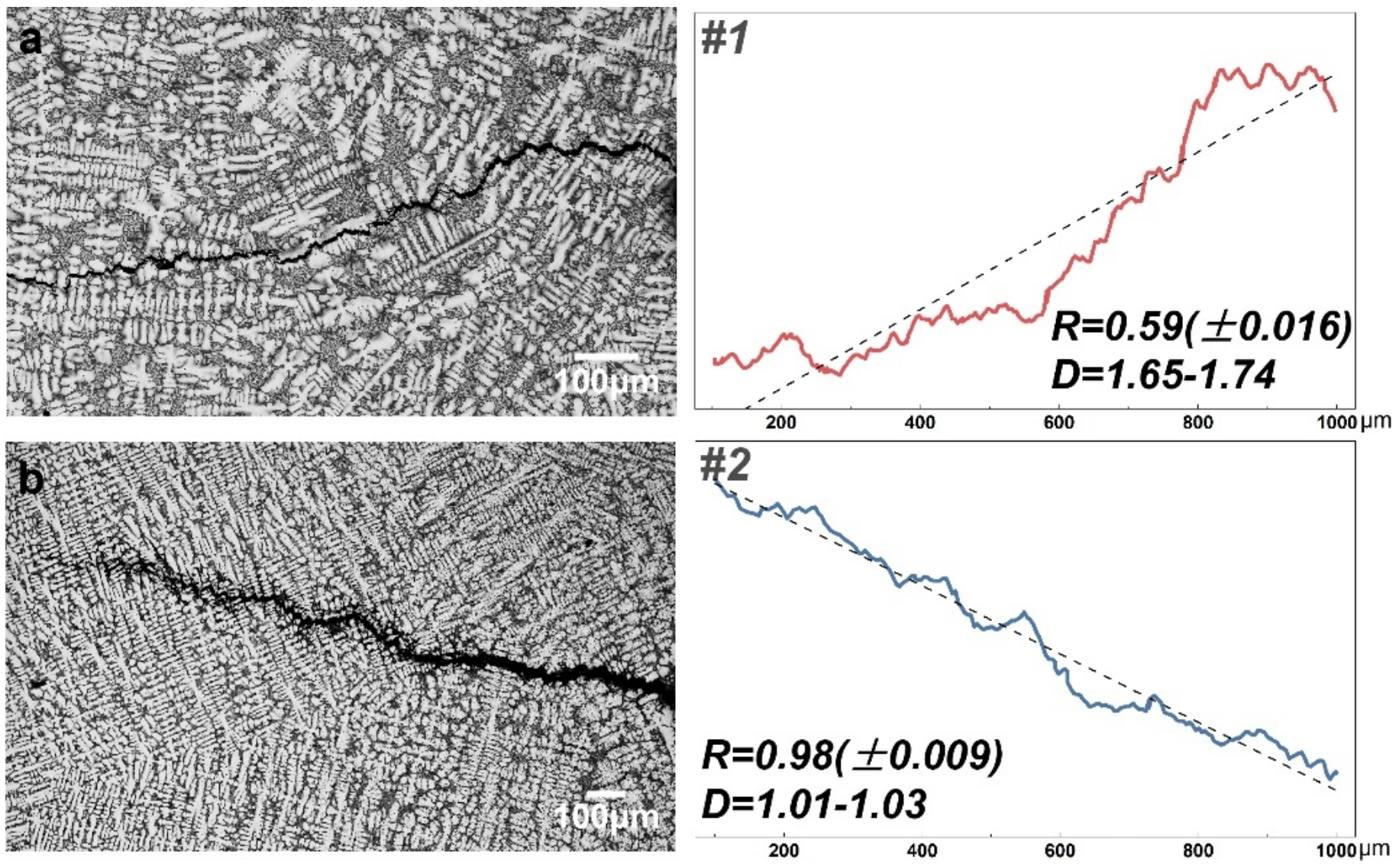


**Supplementary Fig. 22 Quantitative crack deflection analysis. a**, **b**, SEM images (BSE, 20 kV) showing crack propagation paths with digitized trajectories (solid lines) and linear reference fits (dashed lines) for (**a**) alloy #1 (deflection parameter D = 1.65-1.74) and (**b**) alloy #2 (D = 1.01-1.03).

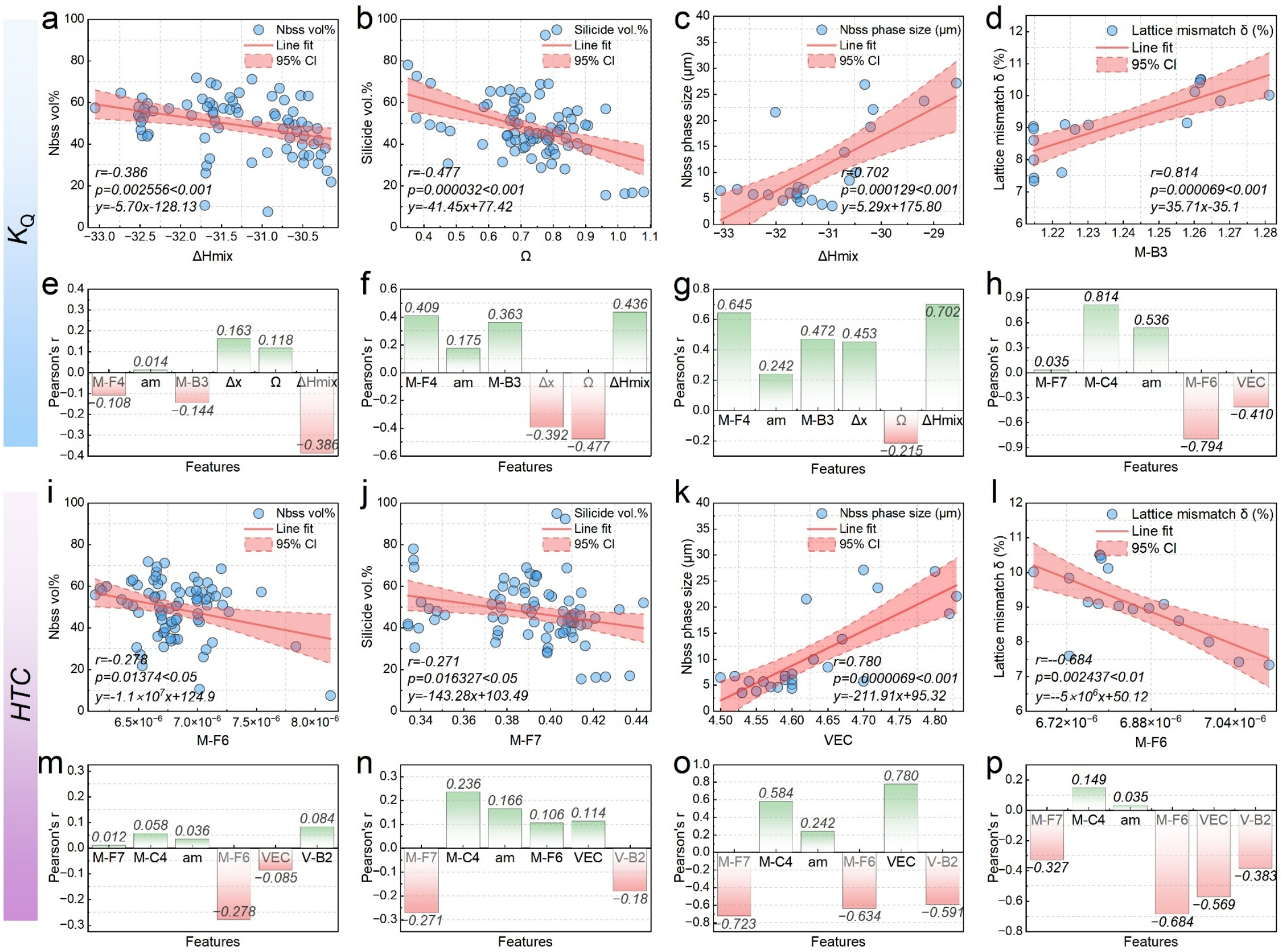


**Supplementary Fig. 23 Correlation between ML descriptors and microstructural parameters. a-d**, Scatter plots with linear fits and 95% confidence intervals for $K_Q$ model descriptors versus microstructural parameters: (**a**) Nbss vol.%, (**b**) silicide vol.%, (**c**) Nbss phase size, and (**d**) lattice mismatch $\delta$. **e-h**, Correlation bar charts (Pearson r) for $K_Q$ descriptors against (**e**) Nbss vol.%, (**f**) silicide vol.%, (**g**) Nbss phase size, and (**h**) lattice mismatch $\delta$; asterisks indicate statistical significance ($p < 0.05$). **i-l**, Scatter plots for HTC model descriptors versus the same microstructural parameters. **m-p**, Correlation bar charts for HTC descriptors. Sample sizes: Nbss vol.% and silicide vol.%, n = 78; Nbss phase size, n = 24; lattice mismatch $\delta$, n = 17.

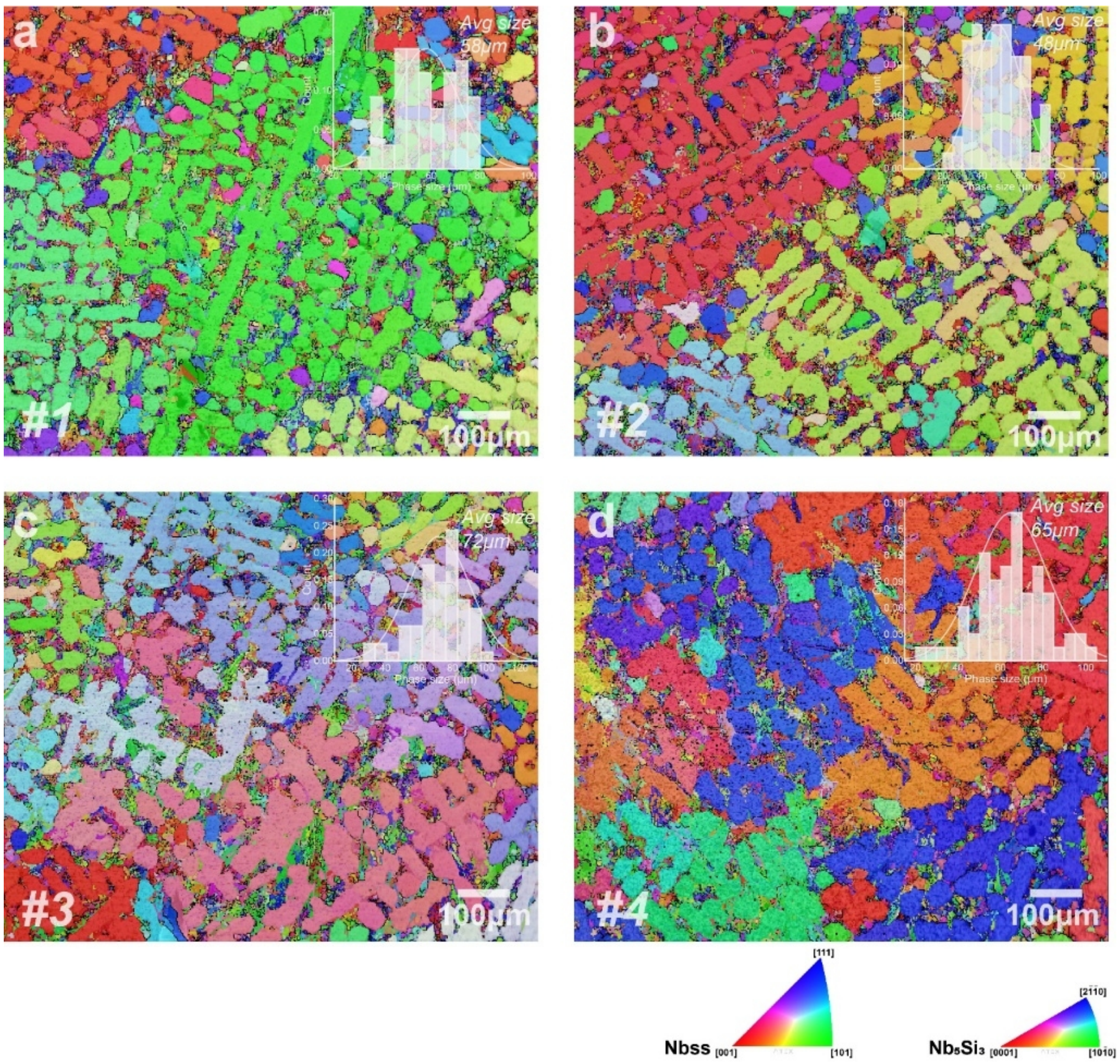


**Supplementary Fig. 24 EBSD grain size maps for the four validation alloys. a-d**, Grain size maps for (**a**) alloy #1, (**b**) alloy #2, (**c**) alloy #3, and (**d**) alloy #4.

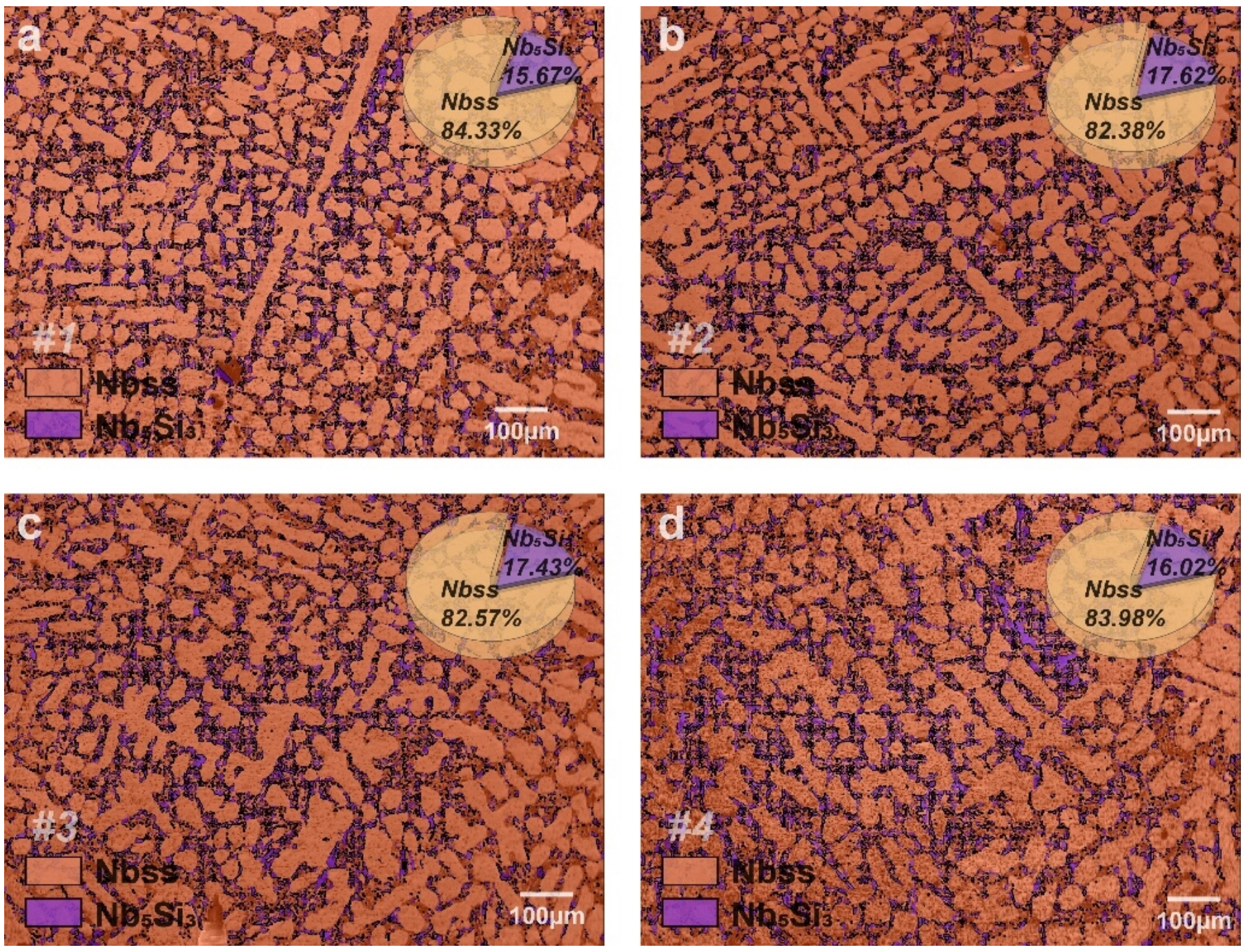


**Supplementary Fig. 25 EBSD phase distribution maps for the four validation alloys. a-d**, Phase maps for (**a**) alloy #1, (**b**) alloy #2, (**c**) alloy #3, and (**d**) alloy #4; orange: Nbss phase; purple: γ-$Nb_5Si_3$ phase.

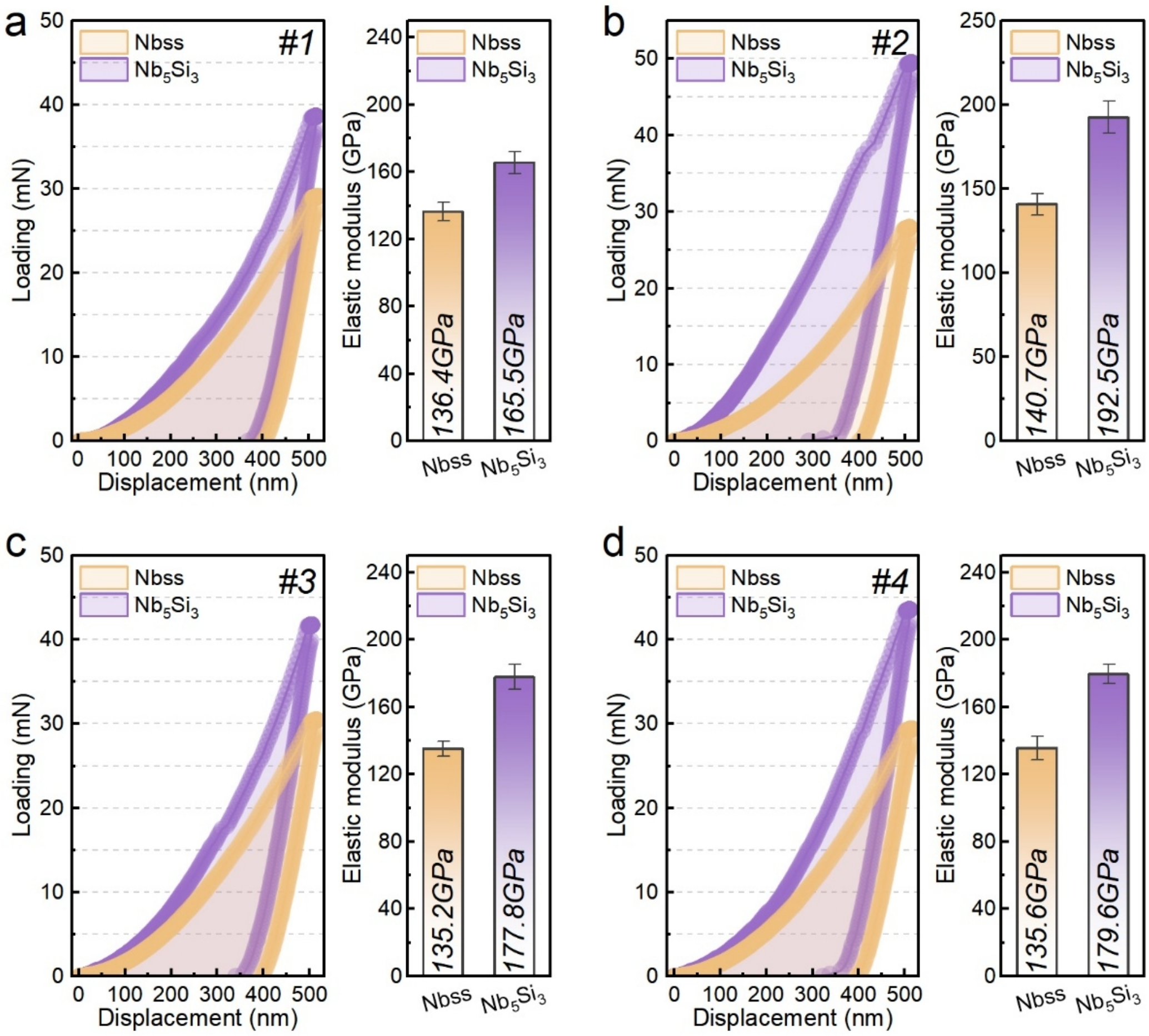


**Supplementary Fig. 26 Nanoindentation results for the four validation alloys. a-d**, Elastic modulus (*E*) and hardness (*H*) measured by nanoindentation for Nbss and γ-$Nb_5Si_3$ phases in (**a**) alloy #1, (**b**) alloy #2, (**c**) alloy #3, and (**d**) alloy #4; error bars: SD of n = 3 indentations per phase.

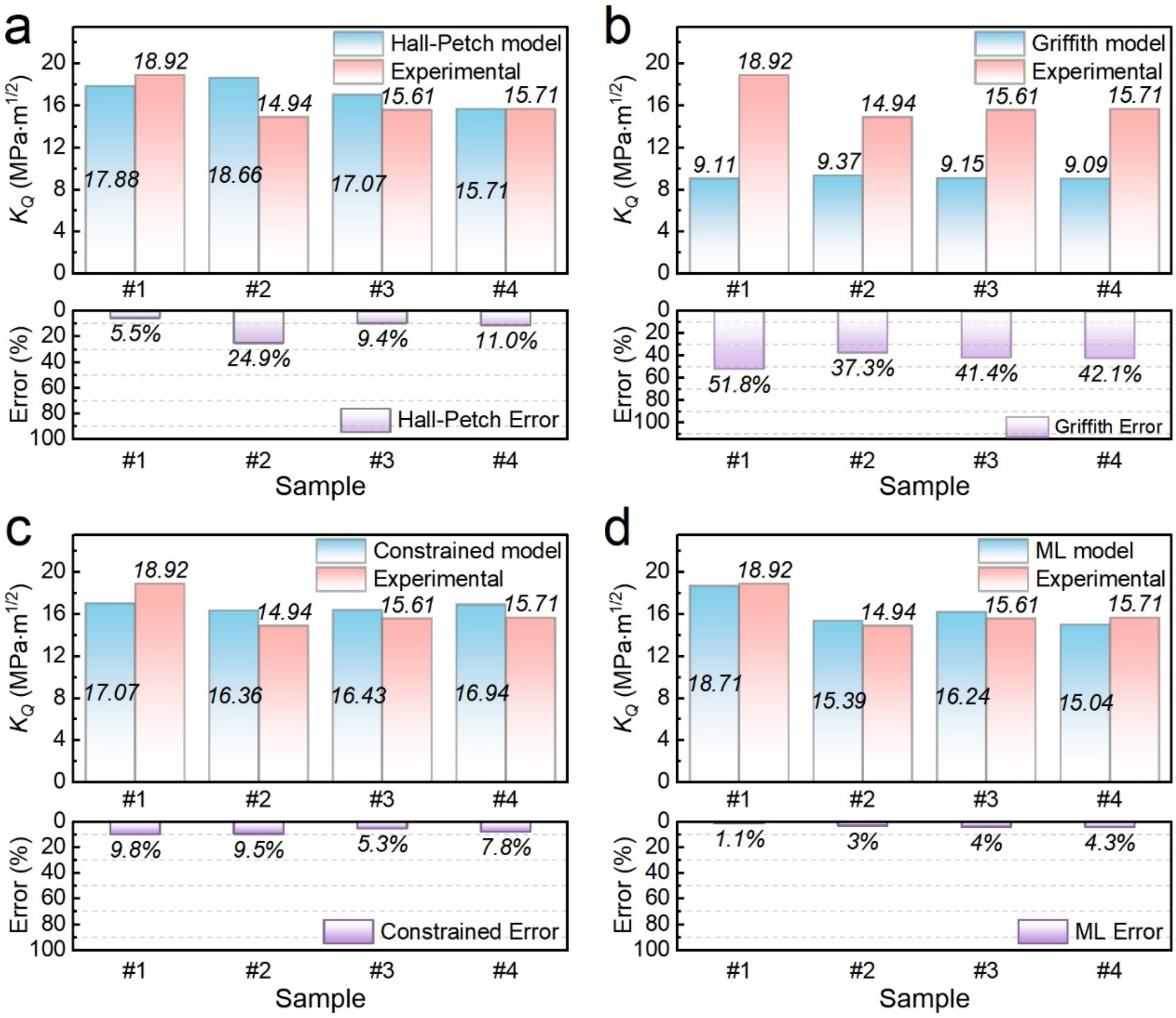


**Supplementary Fig. 27 Prediction accuracy comparison between ML and physics-based models. a-d**, Predicted versus experimental $K_Q$ for (**a**) Hall–Petch-type model, (**b**) Griffith model, (**c**) constrained mixture model, and (**d**) ML (ANN) model.

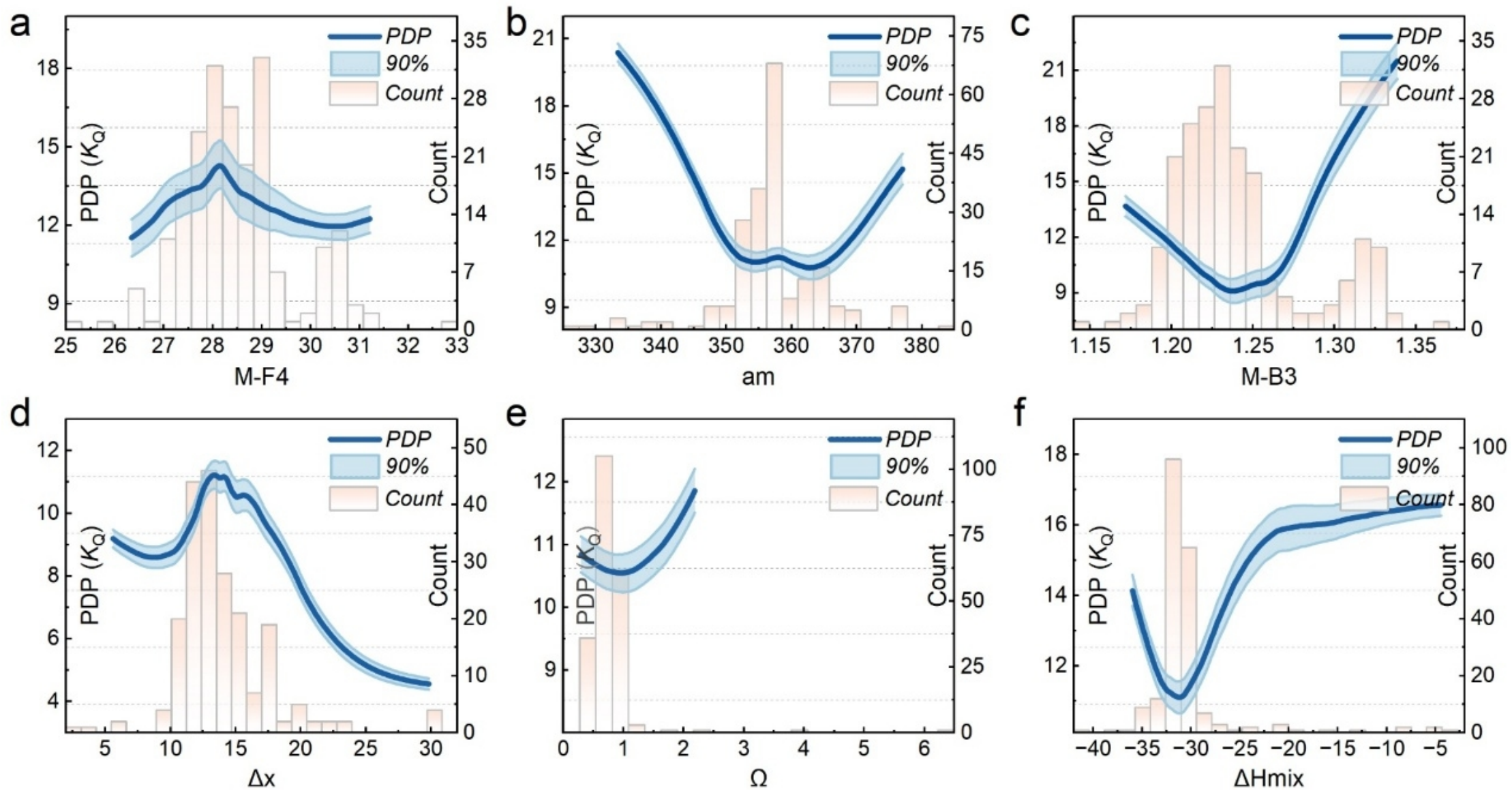


**Supplementary Fig. 28 One-dimensional partial dependence plots for the $K_Q$ model. a-f**, PDP curves showing the marginal effect of each feature on $K_Q$ prediction: (**a**) M-F4, (**b**) am, (**c**) M-B3, (**d**) Δχ, (**e**) Ω, and (**f**) ΔHmix; shaded regions: 95% confidence intervals; calculated from n = 203 samples.

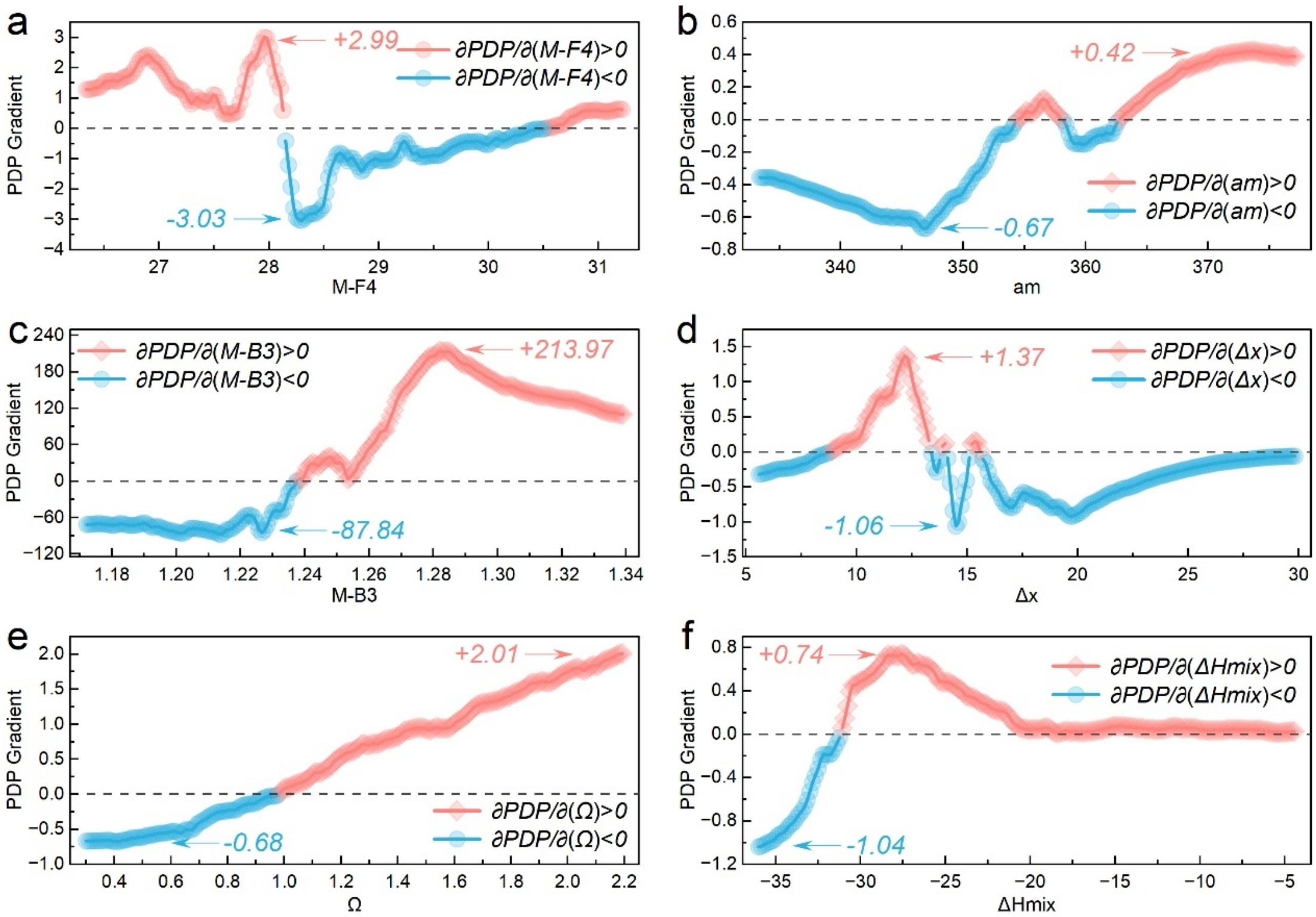


**Supplementary Fig. 29 Partial dependence gradient analysis for the $K_Q$ model. a-f**, Gradient plots showing regions where each feature exerts positive (red) versus negative (blue) effects on $K_Q$ prediction: (**a**) M-F4, (**b**) am, (**c**) M-B3, (**d**) Δχ, (**e**) Ω, and (**f**) ΔHmix; calculated from n = 203 samples.

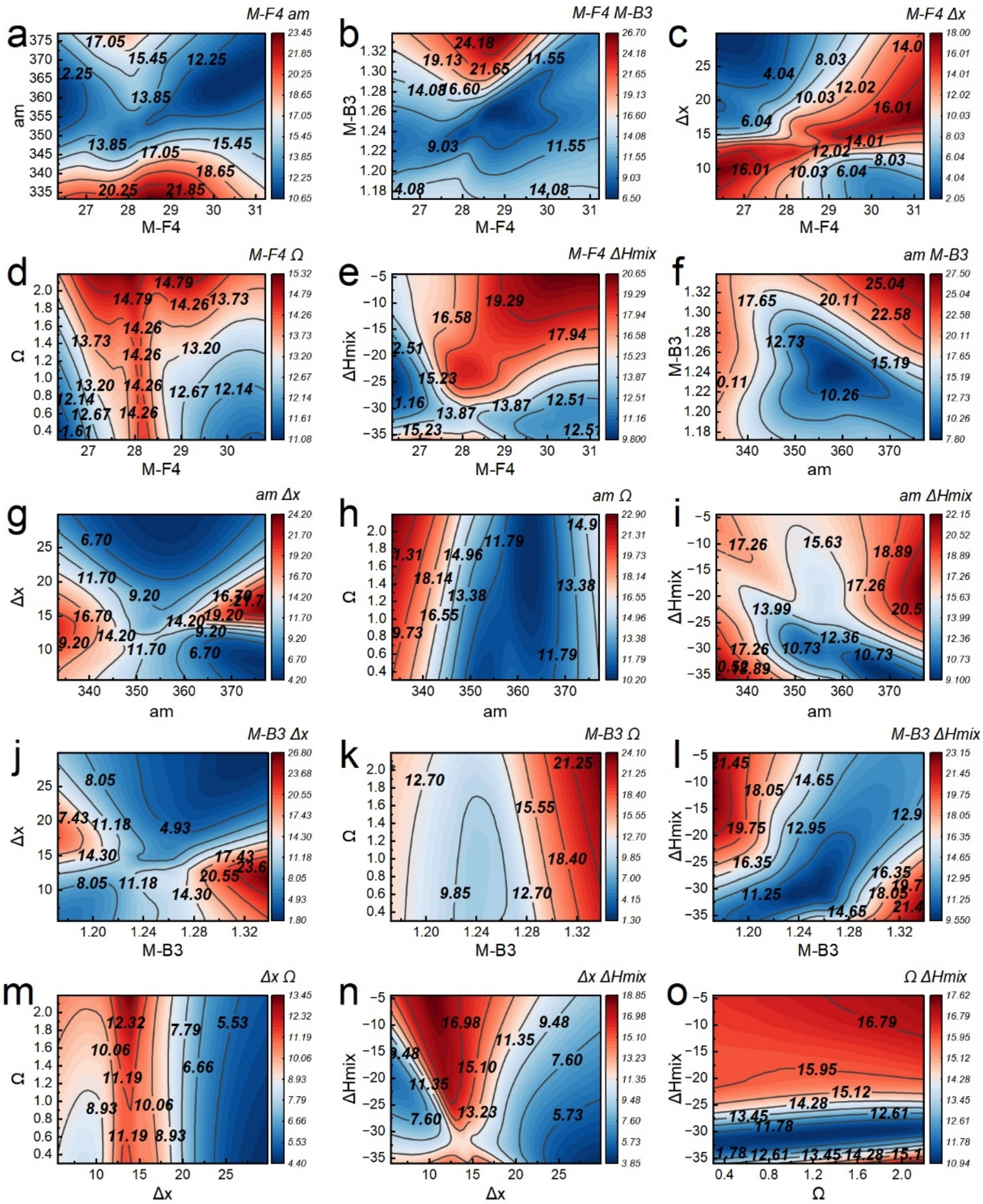


**Supplementary Fig. 30 Two-dimensional partial dependence interaction analysis for the $K_Q$ model. a-o**, 2D PDP heatmaps for all 15 pairwise feature combinations: (**a**) M-F4 - am; (**b**) M-F4 - M-B3; (**c**) M-F4 - Δχ; (**d**) M-F4 - Ω; (**e**) M-F4 - ΔHmix; (**f**) am - M-B3; (**g**) am - Δχ; (**h**) am - Ω; (**i**) am - ΔHmix; (**j**) M-B3 - Δχ; (**k**) M-B3 - Ω; (**l**) M-B3 - ΔHmix; (**m**) Δχ - Ω; (**n**) Δχ - ΔHmix; (**o**) Ω - ΔHmix.

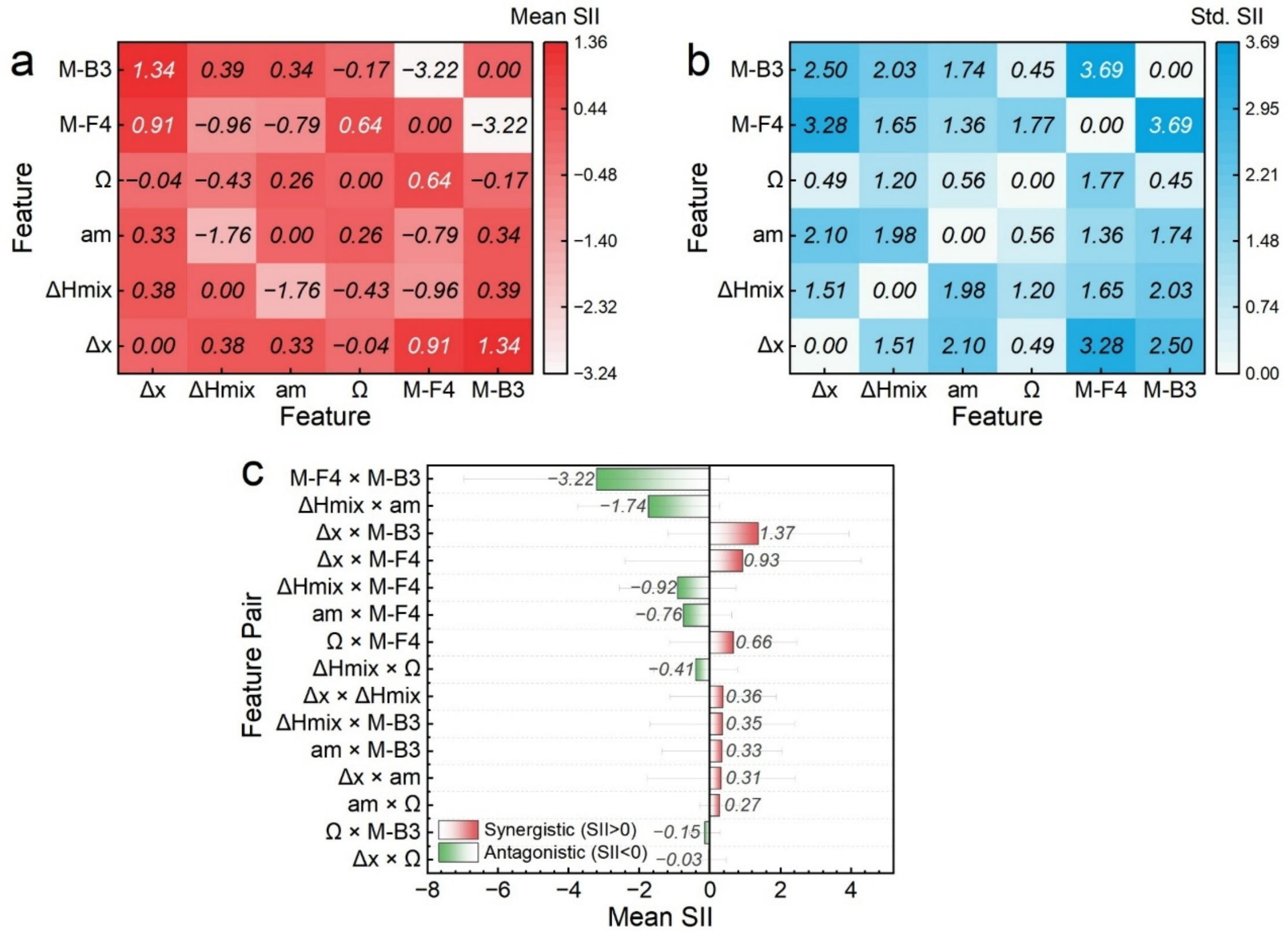


**Supplementary Fig. 31 Shapley Interaction Index analysis for the $K_Q$ model. a**, Mean SII matrix for all 15 feature pairs; color intensity reflects the magnitude of interaction strength; sign indicates synergistic (positive) or antagonistic (negative) interactions. **b**, Standard deviation of SII values across n = 203 samples. **c**, All 15 feature pairs ranked by absolute mean SII; error bars: SD across samples. SII, Shapley Interaction Index.

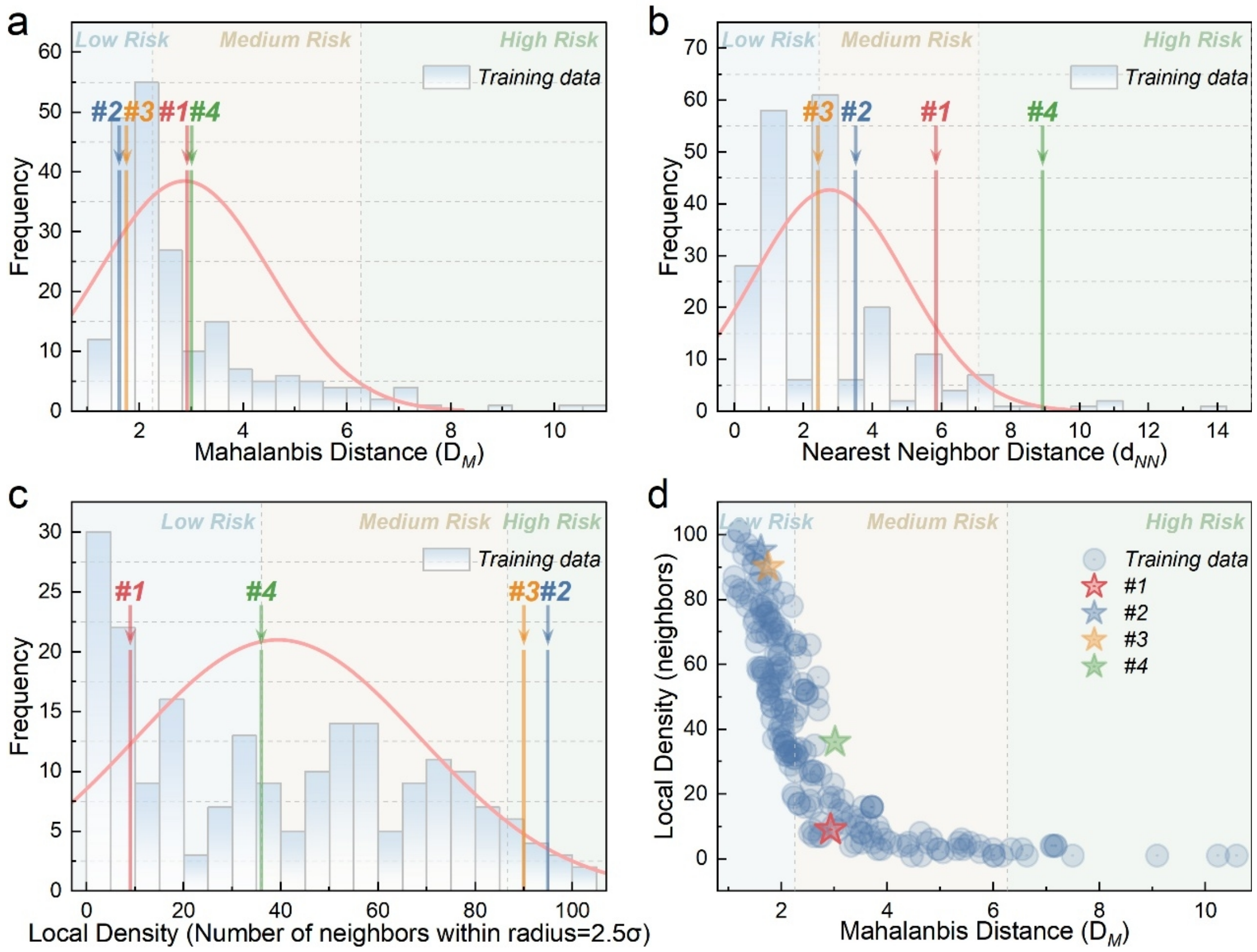


**Supplementary Fig. 32 Distance metric analysis for $K_Q$ predictions. a**, Mahalanobis distance distribution of training data (n = 203) with risk zone boundaries and positions of four optimized alloys. **b**, Nearest-neighbor distance distribution. **c**, Local density distribution using a 2.5$\sigma$ radius kernel. **d**, Scatter plot of Mahalanobis distance versus local density.

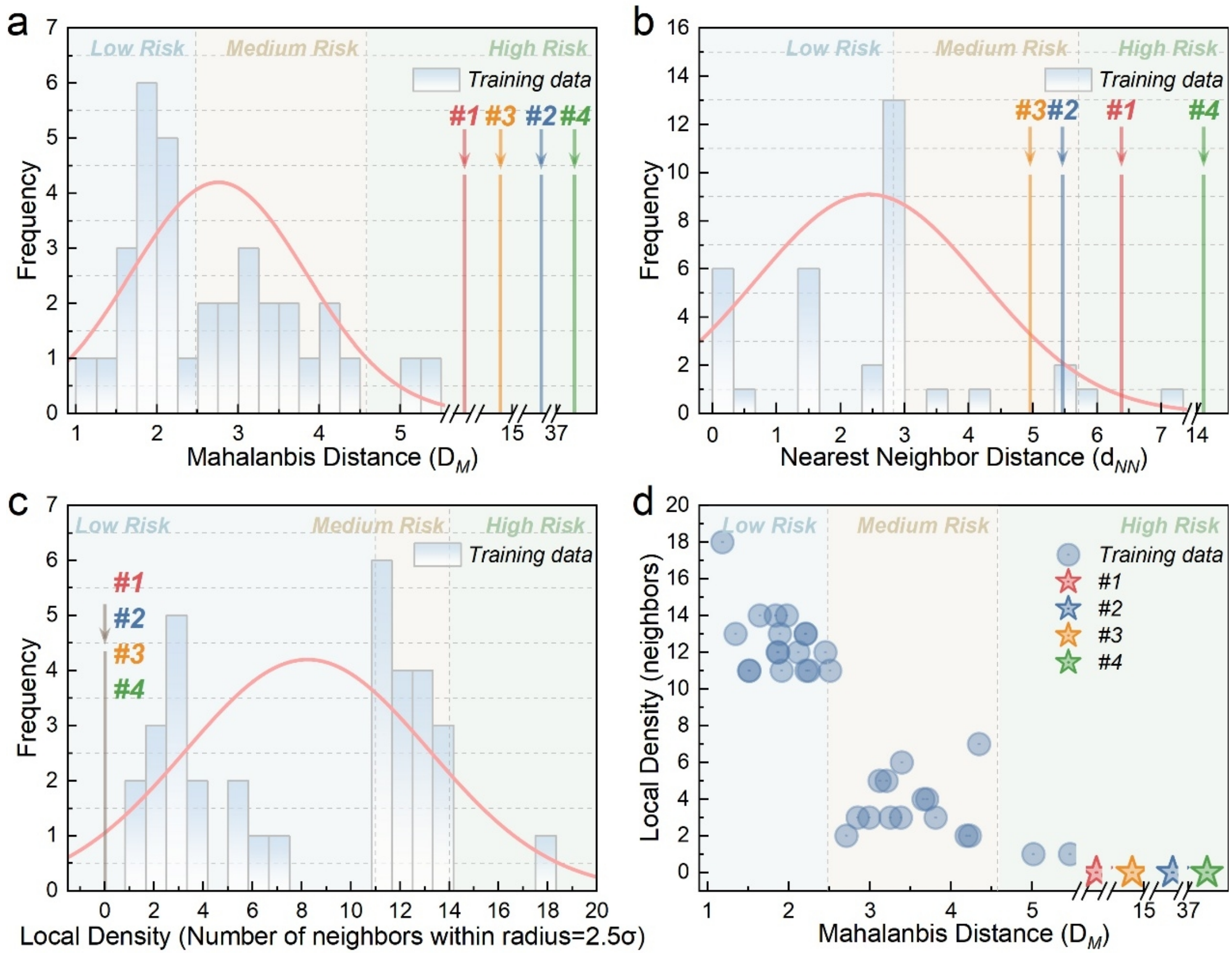


**Supplementary Fig. 33 Distance metric analysis for HTC predictions. a**, Mahalanobis distance distribution of training data (n = 34) with risk zone boundaries and positions of four optimized alloys. **b**, Nearest-neighbor distance distribution. **c**, Local density distribution using a 2.5$\sigma$ radius kernel. **d**, Scatter plot of Mahalanobis distance versus local density.

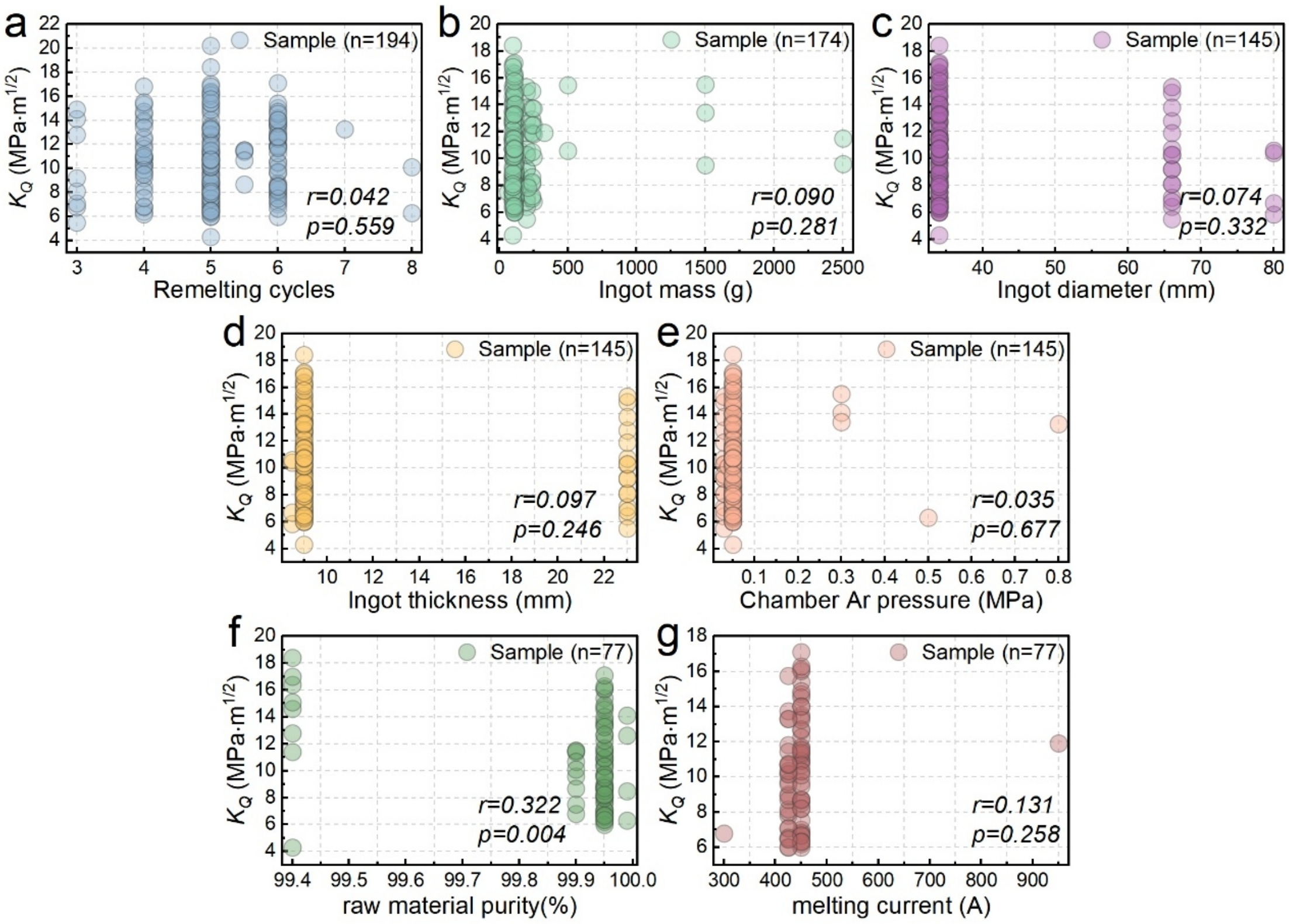


**Supplementary Fig. 34 Correlation between processing parameters and $K_Q$. a-g**, Scatter plots of $K_Q$ versus (**a**) remelting cycles (n = 194), (**b**) ingot mass (n = 174), (**c**) ingot diameter (n = 145), (**d**) ingot thickness (n = 145), (**e**) chamber argon pressure (n = 145), (**f**) raw material purity (n = 77), and (**g**) melting current (n = 77); Pearson r and p-values shown in each panel; only raw material purity shows statistically significant correlation ($p < 0.05$).

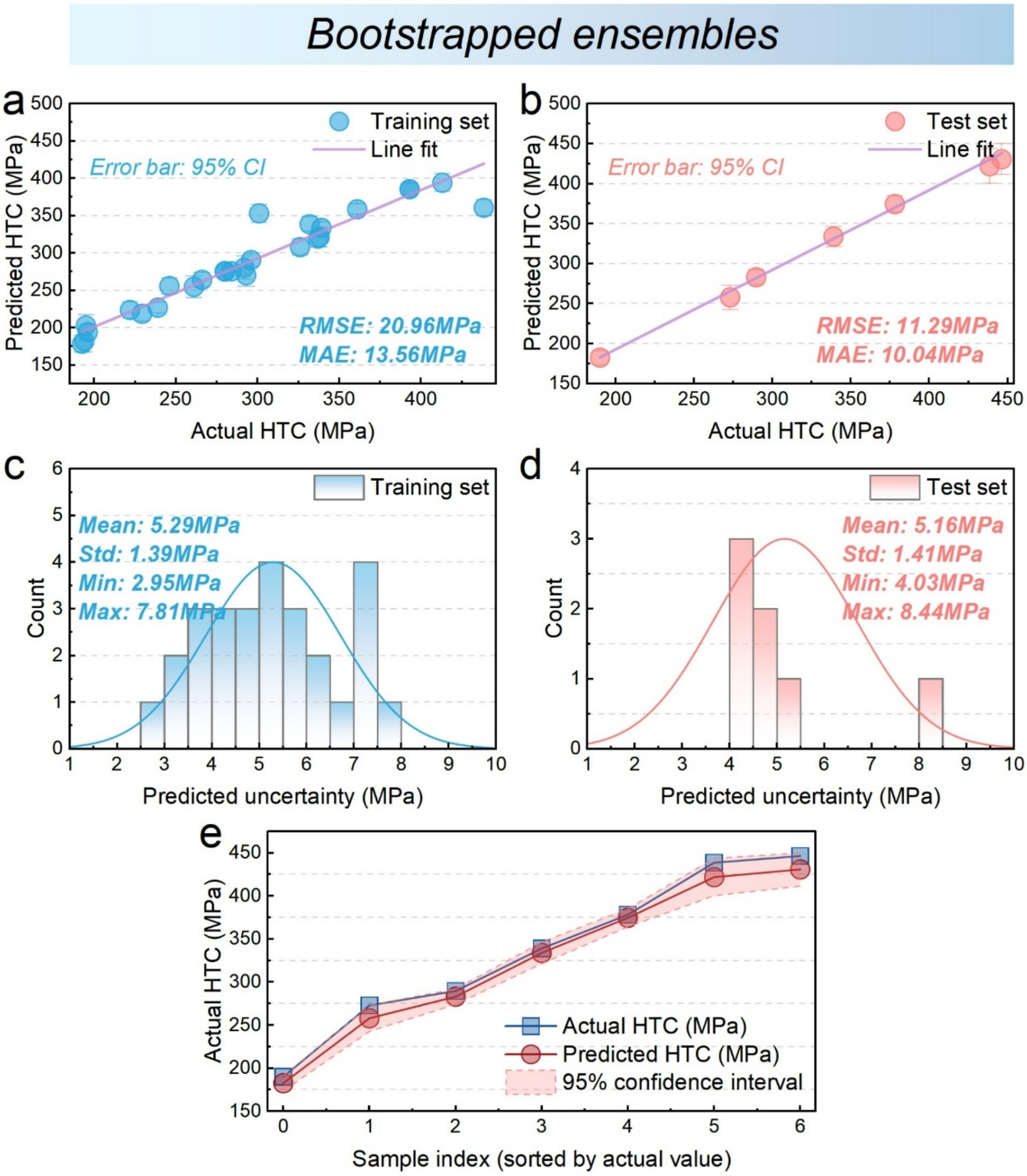


**Supplementary Fig. 35 Bootstrap ensemble uncertainty quantification for the HTC model. a**, **b**, Predicted versus actual HTC for (**a**) training (n = 27) and (**b**) test (n = 7) sets with 95% bootstrap confidence intervals. **c**, **d**, Distribution of prediction uncertainties for (**c**) training and (**d**) test sets. **e**, Test sample predictions with 95% confidence intervals; actual measurements shown as reference points.

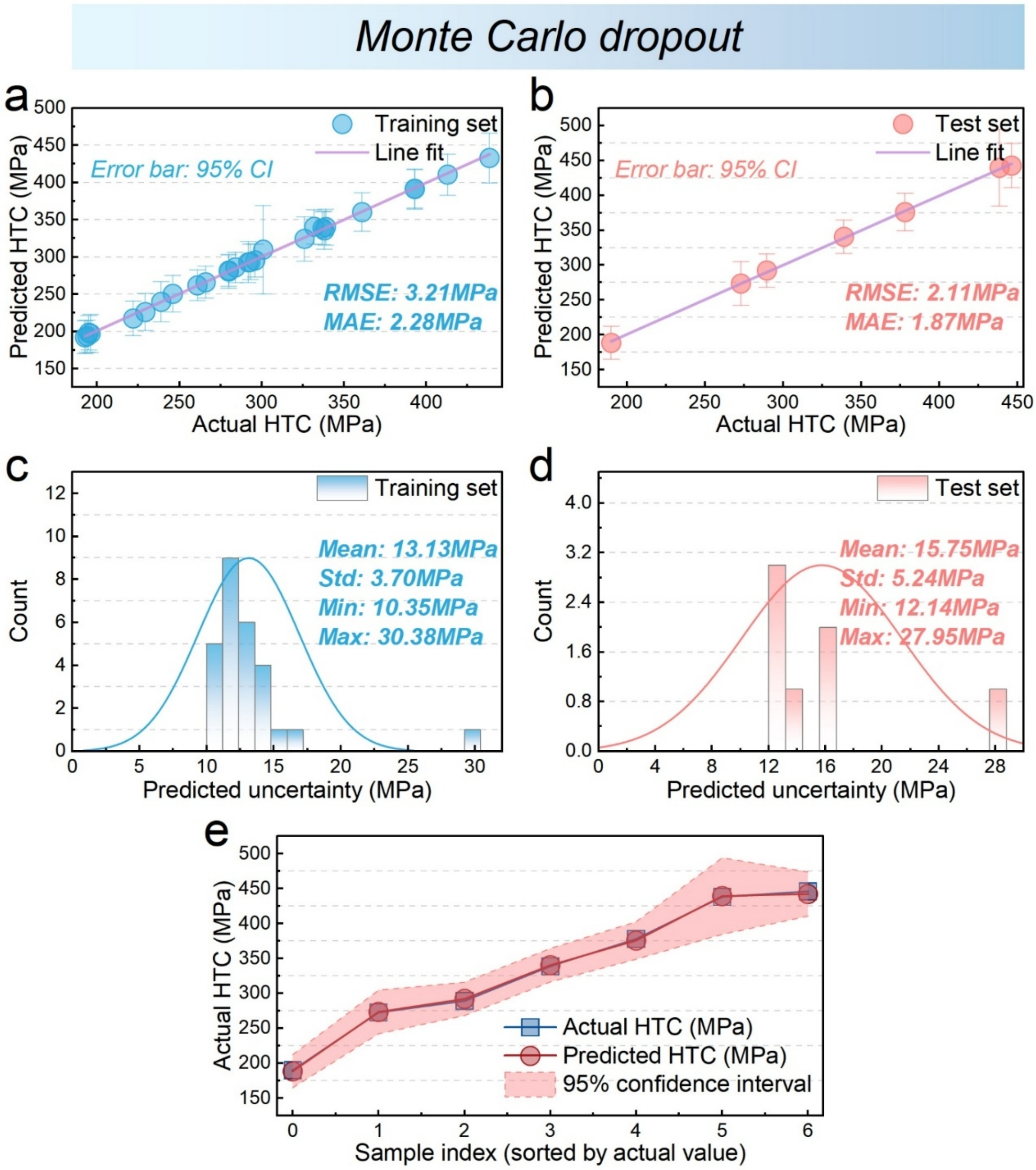


**Supplementary Fig. 36 Monte Carlo dropout uncertainty quantification for the HTC model.** **a**, **b**, Predicted versus actual HTC with MC dropout confidence intervals for (**a**) training (n = 27) and (**b**) test (n = 7) sets. **c**, **d**, Prediction uncertainty distributions for (**c**) training and (**d**) test sets. **e**, Test predictions with confidence intervals bracketing actual measurements.

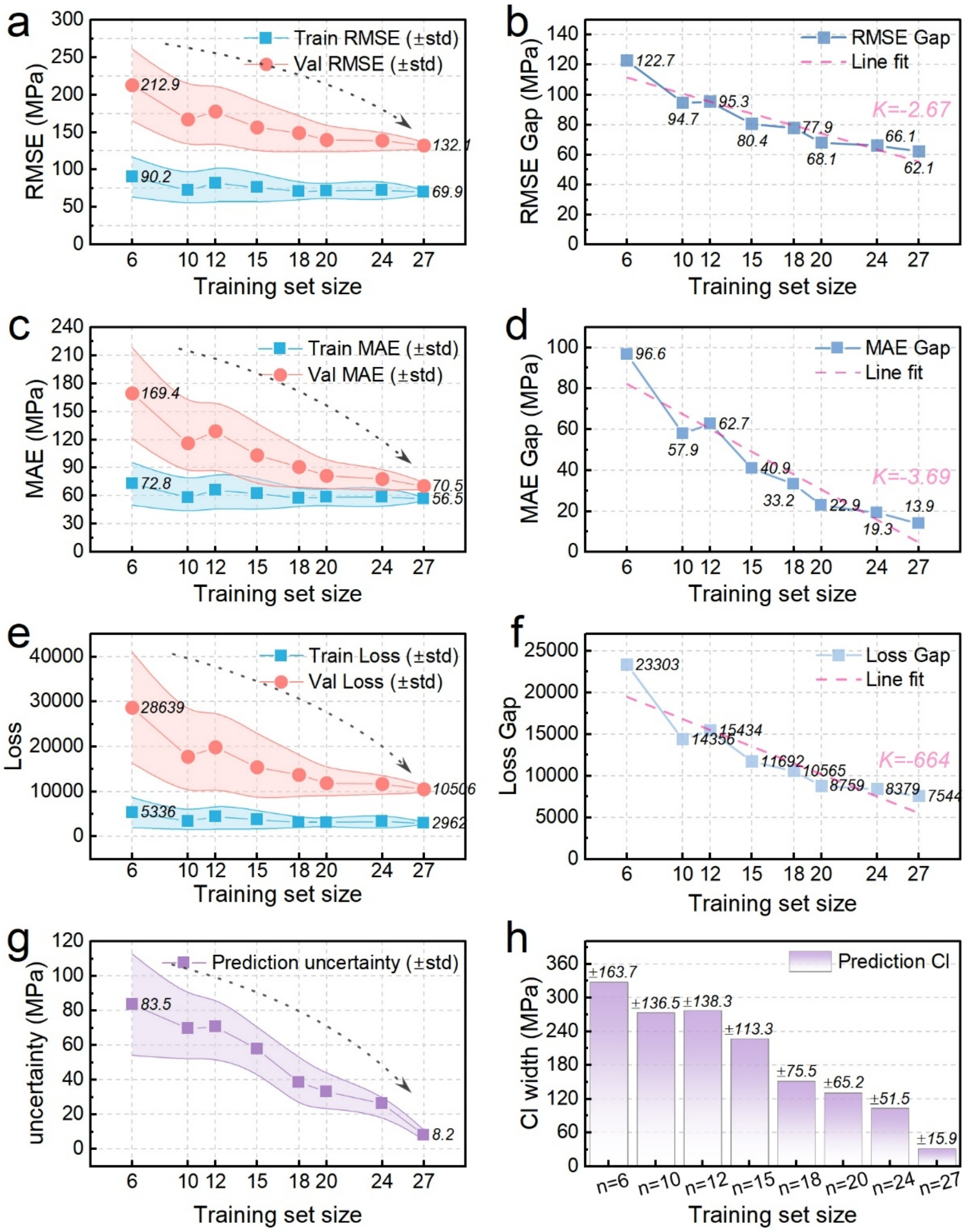


**Supplementary Fig. 37 Learning curve analysis for the HTC model. a**, Training and validation RMSE versus training set size. **b**, RMSE gap between training and validation with fitted convergence trend. **c**, MAE evolution with training set size. **d**, MAE gap reduction. **e**, Loss function trajectories for training and validation. **f**, Loss gap versus training set size. **g**, Prediction uncertainty reduction with increasing training data. **h**, Confidence interval widths as a function of training set size. All metrics evaluated on the HTC dataset (maximum n = 34).

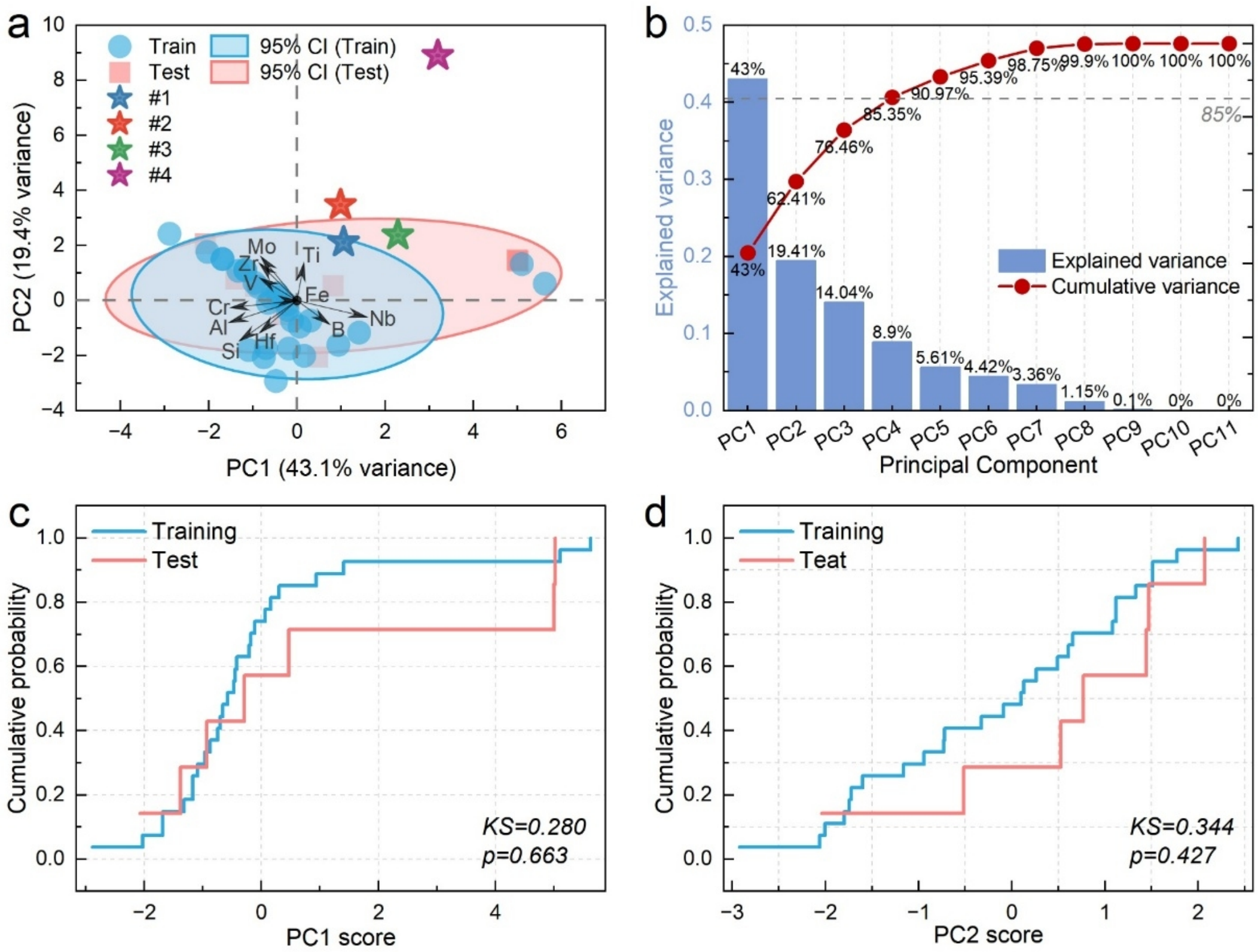


**Supplementary Fig. 38**. **PCA representativeness analysis of the HTC dataset. a**, PCA scatter plot (PC1 vs PC2); training set (n = 27); test set (n = 7); stars: four optimized alloys (#1–#4); ellipses: 95% confidence regions. **b**, Scree plot of explained variance per principal component (bars) and cumulative variance (red line); dashed line marks 85% cumulative threshold. **c**, **d**, Empirical CDFs of (**c**) PC1 and (**d**) PC2 scores for training (n = 27) and test (n = 7) sets; KS = 0.280 (p = 0.663) and KS = 0.344 (p = 0.427), both well above the significance level $\alpha$ = 0.05, confirming no statistically significant distributional difference between the two subsets.

## Supplementary Tables

**Supplementary Table 1 Processing parameter database for the 203 $K_Q$ dataset alloys.** Documentation of purity, atmosphere, argon pressure, melting current, remelting cycles, ingot mass, diameter, thickness, and cooling method for each alloy.

| alloy | Purity | Atmosphere | Ar Pressure | Current | Remelts | Mass | Diameter | Thickness | Cooling |
|---|---|---|---|---|---|---|---|---|---|
| alloy1 | 99.90% | Ar | - | - | 5-6× | 2500g | - | - | Cu crucible |
| alloy2 | 99.90% | Ar | - | - | 6× | 2500g | - | - | Cu crucible |
| alloy3 | 99.90% | Ar | - | 200A-400A | 3× | 250g | - | - | Cu crucible |
| alloy4 | 99.95% | Ar | - | - | 6× | 150g | - | - | Cu crucible |
| alloy5 | 99.95% | Ar | - | - | 6× | 150g | - | - | Cu crucible |
| alloy6 | 99.95% | Ar | - | - | 6× | 150g | - | - | Cu crucible |
| alloy7 | 99.95% | Ar | - | - | 6× | 150g | - | - | Cu crucible |
| alloy8 | - | Ar | - | - | 4× | - | - | - | Cu crucible |
| alloy9 | - | Ar | - | - | 4× | - | - | - | Cu crucible |
| alloy10 | - | Ar | - | - | 4× | - | - | - | Cu crucible |
| alloy11 | - | Ar | - | - | 4× | - | - | - | Cu crucible |
| alloy12 | - | Ar | - | - | 4× | - | - | - | Cu crucible |
| alloy13 | 99.99% | Ar | 0.3MPa | - | 3× | - | - | - | Cu crucible |
| alloy14 | - | Ar | 0.03MPa | - | 4× | 200g | 66mm | 23mm | Cu crucible |
| alloy15 | - | Ar | 0.03MPa | - | 4× | 200g | 66mm | 23mm | Cu crucible |
| alloy16 | - | Ar | 0.03MPa | - | 4× | 200g | 66mm | 23mm | Cu crucible |
| alloy17 | - | Ar | 0.03MPa | - | 4× | 200g | 66mm | 23mm | Cu crucible |
| alloy18 | - | Ar | 0.03MPa | - | ≥3× | 200g | 66mm | 23mm | Cu crucible |
| alloy19 | - | Ar | 0.03MPa | - | ≥3× | 200g | 66mm | 23mm | Cu crucible |
| alloy20 | - | Ar | 0.03MPa | - | ≥3× | 200g | 66mm | 23mm | Cu crucible |
| alloy21 | - | Ar | 0.03MPa | - | ≥3× | 200g | 66mm | 23mm | Cu crucible |
| alloy22 | - | Ar | 0.03MPa | - | ≥4× | 200g | 66mm | 23mm | Cu crucible |
| alloy23 | - | Ar | 0.03MPa | - | ≥4× | 200g | 66mm | 23mm | Cu crucible |
| alloy24 | - | Ar | 0.03MPa | - | ≥4× | 200g | 66mm | 23mm | Cu crucible |
| alloy25 | - | Ar | 0.03MPa | - | ≥4× | 200g | 66mm | 23mm | Cu crucible |
| alloy26 | - | Ar | 0.03MPa | - | ≥3× | 200g | 66mm | 23mm | Cu crucible |
| alloy27 | - | Ar | 0.03MPa | - | ≥3× | 200g | 66mm | 23mm | Cu crucible |
| alloy28 | - | Ar | 0.03MPa | - | 4× | 200g | 66mm | 23mm | Cu crucible |
| alloy29 | - | Ar | 0.03MPa | - | 4× | 200g | 66mm | 23mm | Cu crucible |
| alloy30 | - | Ar | - | - | 6× | 240g | | | Cu crucible |
| alloy31 | - | Ar | - | - | 6× | 240g | | | Cu crucible |
| alloy32 | - | Ar | - | - | 6× | 240g | | | Cu crucible |
| alloy33 | - | Ar | - | - | 6× | 240g | | | Cu crucible |
| alloy34 | - | Ar | - | - | 6× | 240g | | | Cu crucible |
| alloy35 | - | Ar | - | - | 6× | 250g | | | Cu crucible |
| alloy36 | - | Ar | - | - | 6× | 250g | | | Cu crucible |
| alloy37 | - | Ar | - | - | 6× | 250g | | | Cu crucible |
| alloy38 | - | Ar | - | - | 6× | 250g | | | Cu crucible |

| | | | | | | | | | |
|---|---|---|---|---|---|---|---|---|---|
| alloy39 | - | Ar | 0.05MPa | 300A-550A | 6× | 110g | 33-35mm | 8-10mm | Cu crucible |
| alloy40 | - | Ar | 0.05MPa | 300A-550A | 6× | 110g | 33-35mm | 8-10mm | Cu crucible |
| alloy41 | - | Ar | 0.05MPa | 300A-550A | 5× | 110g | 33-35mm | 8-10mm | Cu crucible |
| alloy42 | - | Ar | 0.05MPa | 300A-550A | 5× | 110g | 33-35mm | 8-10mm | Cu crucible |
| alloy43 | - | Ar | 0.05MPa | 300A-550A | 5× | 110g | 33-35mm | 8-10mm | Cu crucible |
| alloy44 | - | Ar | 0.05MPa | 300A-550A | 5× | 110g | 33-35mm | 8-10mm | Cu crucible |
| alloy45 | - | Ar | 0.05MPa | 300A-550A | 5× | 110g | 33-35mm | 8-10mm | Cu crucible |
| alloy46 | - | Ar | 0.05MPa | - | 6× | 100g | 33-35mm | 8-10mm | Cu crucible |
| alloy47 | - | Ar | 0.05MPa | - | 6× | 100g | 33-35mm | 8-10mm | Cu crucible |
| alloy48 | - | Ar | 0.05MPa | - | 6× | 100g | 33-35mm | 8-10mm | Cu crucible |
| alloy49 | - | Ar | 0.05MPa | - | 6× | 100g | 33-35mm | 8-10mm | Cu crucible |
| alloy50 | - | Ar | 0.05MPa | - | 6× | 100g | 33-35mm | 8-10mm | Cu crucible |
| alloy51 | - | Ar | 0.05MPa | - | 6× | 100g | 33-35mm | 8-10mm | Cu crucible |
| alloy52 | - | Ar | 0.05MPa | - | 6× | 100g | 33-35mm | 8-10mm | Cu crucible |
| alloy53 | - | Ar | 0.05MPa | - | ≥5× | 100g | 33-35mm | 8-10mm | Cu crucible |
| alloy54 | - | Ar | 0.05MPa | - | ≥5× | 100g | 33-35mm | 8-10mm | Cu crucible |
| alloy55 | - | Ar | 0.05MPa | - | ≥5× | 100g | 33-35mm | 8-10mm | Cu crucible |
| alloy56 | 99.40% | Ar | 0.05MPa | - | ≥5× | 100g | 33-35mm | 8-10mm | Cu crucible |
| alloy57 | 99.40% | Ar | 0.05MPa | - | ≥5× | 100g | 33-35mm | 8-10mm | Cu crucible |
| alloy58 | 99.40% | Ar | 0.05MPa | - | ≥5× | 100g | 33-35mm | 8-10mm | Cu crucible |
| alloy59 | 99.40% | Ar | 0.05MPa | - | ≥5× | 100g | 33-35mm | 8-10mm | Cu crucible |
| alloy60 | 99.40% | Ar | 0.05MPa | - | ≥5× | 100g | 33-35mm | 8-10mm | Cu crucible |
| alloy61 | 99.40% | Ar | 0.05MPa | - | ≥5× | 100g | 33-35mm | 8-10mm | Cu crucible |
| alloy62 | 99.40% | Ar | 0.05MPa | - | ≥5× | 100g | 33-35mm | 8-10mm | Cu crucible |
| alloy63 | 99.40% | Ar | 0.05MPa | - | ≥5× | 100g | 33-35mm | 8-10mm | Cu crucible |
| alloy64 | 99.95% | Ar | 0.05MPa | 300A-600A | 5× | 110g | 33-35mm | 8-10mm | Cu crucible |
| alloy65 | 99.95% | Ar | 0.05MPa | 300A-600A | 5× | 110g | 33-35mm | 8-10mm | Cu crucible |
| alloy66 | 99.95% | Ar | 0.05MPa | 300A-600A | 5× | 110g | 33-35mm | 8-10mm | Cu crucible |
| alloy67 | 99.95% | Ar | 0.05MPa | 300A-600A | 5× | 110g | 33-35mm | 8-10mm | Cu crucible |
| alloy68 | 99.95% | Ar | 0.05MPa | 300A-600A | 5× | 110g | 33-35mm | 8-10mm | Cu crucible |
| alloy69 | 99.95% | Ar | 0.05MPa | 300A-600A | 5× | 110g | 33-35mm | 8-10mm | Cu crucible |
| alloy70 | 99.95% | Ar | 0.05MPa | 300A-600A | 5× | 110g | 33-35mm | 8-10mm | Cu crucible |
| alloy71 | 99.95% | Ar | 0.05MPa | 300A-600A | 5× | 110g | 33-35mm | 8-10mm | Cu crucible |
| alloy72 | 99.95% | Ar | 0.05MPa | 300A-600A | 5× | 110g | 33-35mm | 8-10mm | Cu crucible |
| alloy73 | 99.95% | Ar | 0.05MPa | 300A-600A | 5× | 110g | 33-35mm | 8-10mm | Cu crucible |
| alloy74 | 99.95% | Ar | 0.05MPa | 300A-600A | 5× | 110g | 33-35mm | 8-10mm | Cu crucible |
| alloy75 | 99.95% | Ar | 0.05MPa | 300A-600A | 5× | 110g | 33-35mm | 8-10mm | Cu crucible |
| alloy76 | 99.95% | Ar | 0.05MPa | 300A-600A | 5× | 110g | 33-35mm | 8-10mm | Cu crucible |
| alloy77 | - | Ar | - | - | 6× | 240g | - | - | Cu crucible |
| alloy78 | - | Ar | - | - | 6× | 240g | - | - | Cu crucible |
| alloy79 | - | Ar | - | - | 6× | 240g | - | - | Cu crucible |
| alloy80 | - | Ar | - | - | 6× | 240g | - | - | Cu crucible |
| alloy81 | - | Ar | - | - | 6× | 240g | - | - | Cu crucible |
| alloy82 | - | Ar | - | - | 6× | 240g | - | - | Cu crucible |

| | | | | | | | | | |
|---|---|---|---|---|---|---|---|---|---|
| alloy83 | - | Ar | - | - | 6× | 240g | - | - | Cu crucible |
| alloy84 | 99.95% | Ar | 0.05MPa | 300A-600A | 4× | 110g | 33-35mm | 8-10mm | Cu crucible |
| alloy85 | 99.95% | Ar | 0.05MPa | 300A-600A | 4× | 110g | 33-35mm | 8-10mm | Cu crucible |
| alloy86 | 99.95% | Ar | 0.05MPa | 300A-600A | 4× | 110g | 33-35mm | 8-10mm | Cu crucible |
| alloy87 | 99.95% | Ar | 0.05MPa | 300A-600A | 4× | 110g | 33-35mm | 8-10mm | Cu crucible |
| alloy88 | 99.95% | Ar | 0.05MPa | 300A-600A | 4× | 110g | 33-35mm | 8-10mm | Cu crucible |
| alloy89 | 99.95% | Ar | 0.05MPa | 300A-600A | 4× | 110g | 33-35mm | 8-10mm | Cu crucible |
| alloy90 | 99.95% | Ar | 0.05MPa | 300A-600A | 4× | 110g | 33-35mm | 8-10mm | Cu crucible |
| alloy91 | 99.95% | Ar | 0.05MPa | 300A-550A | 5× | 120g | 33-35mm | 8-10mm | Cu crucible |
| alloy92 | 99.95% | Ar | 0.05MPa | 300A-550A | 5× | 120g | 33-35mm | 8-10mm | Cu crucible |
| alloy93 | 99.95% | Ar | 0.05MPa | 300A-550A | 5× | 120g | 33-35mm | 8-10mm | Cu crucible |
| alloy94 | 99.95% | Ar | 0.05MPa | 300A-550A | 5× | 120g | 33-35mm | 8-10mm | Cu crucible |
| alloy95 | 99.95% | Ar | 0.05MPa | 300A-550A | 5× | 120g | 33-35mm | 8-10mm | Cu crucible |
| alloy96 | 99.95% | Ar | 0.05MPa | 300A-550A | 5× | 120g | 33-35mm | 8-10mm | Cu crucible |
| alloy97 | 99.95% | Ar | 0.05MPa | 300A-550A | 5× | 120g | 33-35mm | 8-10mm | Cu crucible |
| alloy98 | 99.95% | Ar | 0.05MPa | 300A-550A | 5× | 120g | 33-35mm | 8-10mm | Cu crucible |
| alloy99 | 99.95% | Ar | 0.05MPa | 300A-550A | 5× | 120g | 33-35mm | 8-10mm | Cu crucible |
| alloy100 | 99.95% | Ar | 0.05MPa | 300A-550A | 5× | 110g | 33-35mm | 8-10mm | Cu crucible |
| alloy101 | 99.95% | Ar | 0.05MPa | 300A-550A | 6× | 120g | 33-35mm | 8-10mm | Cu crucible |
| alloy102 | - | Ar | $10^{-4}$ mbar | - | 5× | - | - | - | Cu crucible |
| alloy103 | 99.95% | Ar | 0.05MPa | 300A-600A | 6× | 110g | 33-35mm | 8-10mm | Cu crucible |
| alloy104 | 99.95% | Ar | 0.05MPa | 300A-600A | 6× | 110g | 33-35mm | 8-10mm | Cu crucible |
| alloy105 | 99.95% | Ar | 0.05MPa | 300A-600A | 6× | 110g | 33-35mm | 8-10mm | Cu crucible |
| alloy106 | 99.95% | Ar | 0.05MPa | 300A-600A | 6× | 110g | 33-35mm | 8-10mm | Cu crucible |
| alloy107 | 99.95% | Ar | 0.05MPa | 300A-600A | 6× | 110g | 33-35mm | 8-10mm | Cu crucible |
| alloy108 | 99.95% | Ar | 0.05MPa | 300A-600A | 6× | 110g | 33-35mm | 8-10mm | Cu crucible |
| alloy109 | 99.95% | Ar | 0.05MPa | 300A-600A | 6× | 110g | 33-35mm | 8-10mm | Cu crucible |
| alloy110 | 99.95% | Ar | 0.05MPa | 300A-600A | 6× | 110g | 33-35mm | 8-10mm | Cu crucible |
| alloy111 | 99.95% | Ar | 0.05MPa | 300A-600A | 6× | 110g | 33-35mm | 8-10mm | Cu crucible |
| alloy112 | 99.95% | Ar | 0.05MPa | 300A-600A | 6× | 110g | 33-35mm | 8-10mm | Cu crucible |
| alloy113 | 99.95% | Ar | 0.05MPa | 300A-600A | 5× | 110g | 33-35mm | 8-10mm | Cu crucible |
| alloy114 | 99.95% | Ar | 0.05MPa | 300A-600A | 5× | 110g | 33-35mm | 8-10mm | Cu crucible |
| alloy115 | 99.95% | Ar | 0.05MPa | 300A-600A | 5× | 110g | 33-35mm | 8-10mm | Cu crucible |
| alloy116 | 99.95% | Ar | 0.05MPa | 300A-600A | 5× | 110g | 33-35mm | 8-10mm | Cu crucible |
| alloy117 | 99.90% | Ar | 0.05MPa | 300A-600A | 5-6× | 100g | 33-35mm | 8-10mm | Cu crucible |
| alloy118 | 99.90% | Ar | 0.05MPa | 300A-600A | 5-6× | 100g | 33-35mm | 8-10mm | Cu crucible |
| alloy119 | 99.90% | Ar | 0.05MPa | 300A-600A | 5-6× | 100g | 33-35mm | 8-10mm | Cu crucible |
| alloy120 | 99.90% | Ar | 0.05MPa | 300A-600A | 5-6× | 100g | 33-35mm | 8-10mm | Cu crucible |
| alloy121 | 99.95% | Ar | 0.05MPa | 300A-600A | 5× | 110g | 33-35mm | 8-10mm | Cu crucible |
| alloy122 | 99.95% | Ar | 0.05MPa | 300A-600A | 5× | 110g | 33-35mm | 8-10mm | Cu crucible |
| alloy123 | 99.95% | Ar | 0.05MPa | 300A-600A | 5× | 110g | 33-35mm | 8-10mm | Cu crucible |
| alloy124 | 99.95% | Ar | 0.05MPa | 300A-600A | 5× | 110g | 33-35mm | 8-10mm | Cu crucible |
| alloy125 | 99.95% | Ar | 0.05MPa | 300A-600A | 5× | 110g | 33-35mm | 8-10mm | Cu crucible |
| alloy126 | - | Ar | 0.05MPa | 300A-600A | 6× | 120g | 33-35mm | 8-10mm | Cu crucible |

| | | | | | | | | | |
|---|---|---|---|---|---|---|---|---|---|
| alloy127 | - | Ar | - | - | 5× | - | - | - | Cu crucible |
| alloy128 | - | Ar | - | - | 5× | - | - | - | Cu crucible |
| alloy129 | - | Ar | - | - | 5× | - | - | - | Cu crucible |
| alloy130 | - | Ar | - | - | 5× | - | - | - | Cu crucible |
| alloy131 | - | Ar | - | - | 5× | 210g | - | - | Cu crucible |
| alloy132 | - | Ar | - | - | 4× | - | - | - | Cu crucible |
| alloy133 | - | Ar | - | - | 4× | - | - | - | Cu crucible |
| alloy134 | - | Ar | - | - | 4× | - | - | - | Cu crucible |
| alloy135 | - | Ar | - | - | 4× | - | - | - | Cu crucible |
| alloy136 | - | Ar | - | - | 5× | - | - | - | Cu crucible |
| alloy137 | - | Ar | - | - | 5× | - | - | - | Cu crucible |
| alloy138 | - | Ar | - | - | 5× | - | - | - | Cu crucible |
| alloy139 | - | - | - | - | - | - | - | - | Cu crucible |
| alloy140 | - | - | - | - | - | - | - | - | Cu crucible |
| alloy141 | - | - | - | - | - | - | - | - | Cu crucible |
| alloy142 | - | - | - | - | - | - | - | - | Cu crucible |
| alloy143 | - | - | - | - | - | - | - | - | Cu crucible |
| alloy144 | - | Ar | - | - | ≥5× | 500g | - | - | Cu crucible |
| alloy145 | - | Ar | - | - | ≥5× | 500g | - | - | Cu crucible |
| alloy146 | 99.90% | Ar | - | - | 6× | 65g | - | - | Cu crucible |
| alloy147 | - | Ar | - | - | - | - | 80mm | 8mm-9mm | Cu crucible |
| alloy148 | - | Ar | - | - | - | - | 80mm | 8mm-9mm | Cu crucible |
| alloy149 | - | Ar | - | - | - | - | 80mm | 8mm-9mm | Cu crucible |
| alloy150 | - | Ar | - | - | - | - | 80mm | 8mm-9mm | Cu crucible |
| alloy151 | 99.95% | Ar | 0.03MPa | 300A-600A | 5× | 110g | 33-35mm | 8-10mm | Cu crucible |
| alloy152 | 99.90% | Ar | 0.04MPa | 300A-600A | 8× | 100g | 33-35mm | 8-10mm | Cu crucible |
| alloy153 | 99.95% | Ar | <0.8MPa | 300A-600A | 6-8× | 110g | 33-35mm | 8-10mm | Cu crucible |
| alloy154 | 99.99% | Ar | 0.5MPa | 300A-600A | 8× | 110g | 33-35mm | 8-10mm | Cu crucible |
| alloy155 | 99.99% | Ar | - | - | 6× | 100g | - | - | Cu crucible |
| alloy156 | 99.99% | Ar | - | - | 6× | 100g | - | - | Cu crucible |
| alloy157 | - | Ar | - | - | ≥4× | - | - | - | Cu crucible |
| alloy158 | - | Ar | - | - | ≥4× | - | - | - | Cu crucible |
| alloy159 | - | Ar | 0.05MPa | - | 5× | 100g | 33-35mm | 8-10mm | Cu crucible |
| alloy160 | - | Ar | 0.05MPa | - | 5× | 100g | 33-35mm | 8-10mm | Cu crucible |
| alloy161 | - | Ar | 0.05MPa | - | 5× | 100g | 33-35mm | 8-10mm | Cu crucible |
| alloy162 | - | Ar | 0.05MPa | - | 5× | 100g | 33-35mm | 8-10mm | Cu crucible |
| alloy163 | - | Ar | 0.05MPa | - | 5× | 100g | 33-35mm | 8-10mm | Cu crucible |
| alloy164 | - | Ar | 0.05MPa | - | 5× | 100g | 33-35mm | 8-10mm | Cu crucible |
| alloy165 | - | Ar | 0.05MPa | - | 5× | 100g | 33-35mm | 8-10mm | Cu crucible |
| alloy166 | - | Ar | 0.05MPa | - | 5× | 100g | 33-35mm | 8-10mm | Cu crucible |
| alloy167 | - | Ar | 0.05MPa | - | 5× | 100g | 33-35mm | 8-10mm | Cu crucible |
| alloy168 | - | Ar | 0.05MPa | - | 5× | 100g | 33-35mm | 8-10mm | Cu crucible |
| alloy169 | - | Ar | 0.05MPa | - | 5× | 100g | 33-35mm | 8-10mm | Cu crucible |
| alloy170 | - | Ar | 0.05MPa | - | 5× | 100g | 33-35mm | 8-10mm | Cu crucible |

| | | | | | | | | | |
|---|---|---|---|---|---|---|---|---|---|
| alloy171 | - | Ar | 0.05MPa | - | 5× | 100g | 33-35mm | 8-10mm | Cu crucible |
| alloy172 | - | Ar | 0.05MPa | - | 5× | 100g | 33-35mm | 8-10mm | Cu crucible |
| alloy173 | - | Ar | 0.2-0.4MPa | - | ≥4× | 1500g | - | - | Cu crucible |
| alloy174 | - | Ar | 0.2-0.4MPa | - | ≥4× | 1500g | - | - | Cu crucible |
| alloy175 | - | Ar | 0.05MPa | - | 5× | 100g | 33-35mm | 8-10mm | Cu crucible |
| alloy176 | - | Ar | 0.05MPa | - | 5× | 100g | 33-35mm | 8-10mm | Cu crucible |
| alloy177 | - | Ar | 0.05MPa | - | 5× | 100g | 33-35mm | 8-10mm | Cu crucible |
| alloy178 | - | Ar | 0.05MPa | - | 5× | 100g | 33-35mm | 8-10mm | Cu crucible |
| alloy179 | - | Ar | 0.05MPa | - | 5× | 100g | 33-35mm | 8-10mm | Cu crucible |
| alloy180 | - | Ar | 0.05MPa | - | 5× | 100g | 33-35mm | 8-10mm | Cu crucible |
| alloy181 | - | Ar | 0.05MPa | - | 5× | 100g | 33-35mm | 8-10mm | Cu crucible |
| alloy182 | - | Ar | 0.05MPa | - | 5× | 100g | 33-35mm | 8-10mm | Cu crucible |
| alloy183 | - | Ar | 0.05MPa | - | 5× | 100g | 33-35mm | 8-10mm | Cu crucible |
| alloy184 | - | Ar | 0.05MPa | - | 5× | 100g | 33-35mm | 8-10mm | Cu crucible |
| alloy185 | - | Ar | 0.05MPa | - | 5× | 100g | 33-35mm | 8-10mm | Cu crucible |
| alloy186 | - | Ar | 0.05MPa | - | 5× | 100g | 33-35mm | 8-10mm | Cu crucible |
| alloy187 | - | Ar | 0.05MPa | - | 5× | 100g | 33-35mm | 8-10mm | Cu crucible |
| alloy188 | - | Ar | 0.05MPa | - | 5× | 100g | 33-35mm | 8-10mm | Cu crucible |
| alloy189 | - | Ar | 0.05MPa | - | 5× | 100g | 33-35mm | 8-10mm | Cu crucible |
| alloy190 | - | Ar | 0.05MPa | - | 5× | 100g | 33-35mm | 8-10mm | Cu crucible |
| alloy191 | - | Ar | 0.05MPa | - | 5× | 100g | 33-35mm | 8-10mm | Cu crucible |
| alloy192 | - | Ar | 0.05MPa | - | 5× | 100g | 33-35mm | 8-10mm | Cu crucible |
| alloy193 | - | Ar | 0.05MPa | 300A-550A | 5× | 110g | 33-35mm | 8-10mm | Cu crucible |
| alloy194 | - | Ar | 0.05MPa | 300A-550A | 5× | 110g | 33-35mm | 8-10mm | Cu crucible |
| alloy195 | - | Ar | 0.05MPa | 300A-550A | 5× | 110g | 33-35mm | 8-10mm | Cu crucible |
| alloy196 | - | Ar | 0.05MPa | 300A-550A | 5× | 110g | 33-35mm | 8-10mm | Cu crucible |
| alloy197 | - | Ar | 0.05MPa | 300A-550A | 5× | 110g | 33-35mm | 8-10mm | Cu crucible |
| alloy198 | - | Ar | 0.05MPa | 300A-550A | 5× | 110g | 33-35mm | 8-10mm | Cu crucible |
| alloy199 | - | Ar | 0.05MPa | 300A-550A | 5× | 110g | 33-35mm | 8-10mm | Cu crucible |
| alloy200 | - | Ar | 0.05MPa | 300A-550A | 5× | 110g | 33-35mm | 8-10mm | Cu crucible |
| alloy201 | - | Ar | 0.05MPa | 300A-550A | 5× | 110g | 33-35mm | 8-10mm | Cu crucible |
| alloy202 | - | Ar | 0.05MPa | - | 4× | 1500g | 10cm | - | Cu crucible |
| alloy203 | - | Ar | - | 900-1000A | 5× | 330g | - | - | Cu crucible |

Note: "-" indicates parameter not reported in original publication.

**Supplementary Table 2 Summary statistics for processing parameters across the dataset.** Documentation rate, range, mean ± SD, median, and most common value for remelting cycles, ingot mass, ingot diameter, ingot thickness, chamber argon pressure, protective atmosphere, cooling method, raw material purity, and melting current across all 203 $K_Q$ dataset alloys.

| Parameter | n | Documentation Rate | Range | Mean ± SD | Median | Most Common Value(s) |
|---|---|---|---|---|---|---|
| Remelting cycles | 194 | 95.6% | 3-8times | 5.0 ± 0.8 | 5 | 5 (55.2%) |
| Ingot mass | 174 | 85.7% | 65-2500 g | 187 ± 313 | 110 | 100-110 g (65.5%) |
| Ingot diameter | 146 | 71.9% | 33-100 mm | - | - | 33-35 mm (85.6%) |
| Ingot thickness | 145 | 71.4% | 8-23 mm | - | - | 8-10 mm (86.2%) |
| Chamber Ar pressure | 146 | 71.9% | 0.03-0.5 MPa | - | 0.05 MPa | 0.05 MPa (83.6%) |
| Protective atmosphere | 198 | 97.5% | Ar gas | - | - | Ar (100%) |
| Cooling method | 203 | 100% | Water-cooled Cu | - | - | Cu crucible (100%) |
| Raw material purity | 77 | 37.9% | 99.4-99.99% | - | 99.95% | 99.95% (72.7%) |
| Melting current | 77 | 37.9% | 200-1000 A | - | - | 300-600 A (97.4%) |

Note: "-" indicates not applicable or not calculated for categorical/non-normal distributions.

**Supplementary Table 3 Key features identified for $K_Q$ and HTC prediction.** Detailed descriptions of the six most influential factors for each property, including their physical significance and units.

| Factors Influencing $K_Q$ | | Factors Influencing HTC | |
|---|---|---|---|
| Factors | Description | Factors | Description |
| M-F4 | melting enthalpy | M-F7 | specific heat capacity |
| am | atomic radius mismatch | M-C4 | Covalent radius (Pyykko single bond) |
| M-B3 | electrophilicity index | am | atomic radius mismatch |
| Δχ | electronegativity difference | M-F6 | thermal expansion coefficient |
| Ω | thermodynamic parameter | VEC | valence electron concentration |
| ΔHmix | enthalpy of mixing | V-B2 | electron affinity |

**Supplementary Table 4 Influence and weight coefficients from SHAP analysis.** Complete influence coefficients ($k_{ni}$) and weight coefficients ($Z_{ni}$) derived from SHAP analysis for all features in both models.

| Properties | $K_Q$ | | | | | | HTC | | | | | |
|---|---|---|---|---|---|---|---|---|---|---|---|---|
| Key alloy factors | M-F4 | am | M-B3 | Δχ | Ω | ΔHmix | M-F7 | M-C4 | am | M-F6 | VEC | V-B2 |
| $k_{ni}$ | 5.328 | -9.474 | -4.295 | -13.845 | 5.144 | 4.206 | -224.893 | -222.382 | 207.326 | -186.108 | -126.253 | -184.630 |
| $Z_{ni}$ | 0.1226 | 0.224 | 0.102 | 0.327 | 0.122 | 0.099 | 0.195 | 0.193 | 0.18 | 0.162 | 0.110 | 0.160 |

**Supplementary Table 5 Composition search space for multi-objective optimization.** Lower and upper bounds (at.%) for each element in the four alloy systems (8-, 7-, 6-, and 5-element) used in NSGA-II optimization.

| Element/at.% | Nb | Si | Ti | Al | Cr | Hf | Zr | V |
|---|---|---|---|---|---|---|---|---|
| Lower Limit | 35 | 12 | 20 | 0 | 0 | 0 | 0 | 0 |
| Upper Limit | 55 | 16 | 35 | 2 | 2 | 2 | 4 | 5 |

**Supplementary Table 6. Predicted $K_Q$ and HTC of recommended alloys under three weighting schemes.** One representative alloy selected from the Pareto frontier for each of three $K_Q$:HTC weighting schemes (0.8:0.2, 1:1, 0.2:0.8) across four alloy systems (8-, 7-, 6-, and 5-element).

| System | Weighting scheme | Predicted $K_Q$ ($MPa \cdot m^{1/2}$) | Predicted HTC (MPa) |
|---|---|---|---|
| 8-element | $K_Q$:HTC = 0.8:0.2 | 20.33 | 116.84 |
| 8-element | $K_Q$:HTC = 1:1 | 18.71 | 325.67 |
| 8-element | $K_Q$:HTC = 0.2:0.8 | 16.07 | 470.55 |
| 7-element | $K_Q$:HTC = 0.8:0.2 | 16.14 | 221.60 |
| 7-element | $K_Q$:HTC = 1:1 | 15.39 | 361.12 |
| 7-element | $K_Q$:HTC = 0.2:0.8 | 13.26 | 422.87 |
| 6-element | $K_Q$:HTC = 0.8:0.2 | 16.41 | 186.91 |
| 6-element | $K_Q$:HTC = 1:1 | 16.24 | 278.33 |
| 6-element | $K_Q$:HTC = 0.2:0.8 | 13.44 | 366.33 |
| 5-element | $K_Q$:HTC = 0.8:0.2 | 16.71 | 166.82 |
| 5-element | KQ:HTC = 1:1 | 15.04 | 259.49 |
| 5-element | KQ:HTC = 0.2:0.8 | 14.05 | 329.16 |

**Supplementary Table 7 Pareto frontier stability assessment for the 8-element system.** Ten independent NSGA-II runs (different random seeds) for 8-element system showing high reproducibility.

| Property Combination | Representative Composition (at.%) | Appearance Frequency | Property Variation (CV%) |
|---|---|---|---|
| $K_Q$: 18.5-19.0, HTC: 320-325 | Nb-12.3Si-21.4Ti-2.0Al-2.0Cr-0.5Hf-4.3Zr-4.3V | 9/10 | $K_Q$: 2.1%, HTC: 2.8% |
| $K_Q$: 17.5-18.0, HTC: 330-335 | Nb-12.5Si-22.1Ti-1.9Al-1.9Cr-0.6Hf-4.5Zr-4.2V | 8/10 | $K_Q$: 2.5%, HTC: 2.3% |
| $K_Q$: 16.5-17.0, HTC: 340-345 | Nb-13.1Si-22.8Ti-1.8Al-1.8Cr-0.7Hf-4.2Zr-4.0V | 9/10 | $K_Q$: 2.7%, HTC: 1.9% |
| $K_Q$: 15.5-16.0, HTC: 350-355 | Nb-13.8Si-23.5Ti-1.7Al-1.7Cr-0.8Hf-3.9Zr-3.8V | 8/10 | $K_Q$: 2.9%, HTC: 2.1% |
| $K_Q$: 14.5-15.0, HTC: 355-360 | Nb-14.5Si-24.2Ti-1.6Al-1.6Cr-0.9Hf-3.6Zr-3.5V | 10/10 | $K_Q$: 1.8%, HTC: 1.6% |

Note: To assess the stability of the Pareto frontier, we performed 10 independent NSGA-II optimizations for the 8-element system (Nb-Si-Ti-Al-Cr-Hf-Zr-V) using different random seeds (12345, 23456, 34567, 45678, 56789, 67890, 78901, 89012, 90123, 10234). For each property interval, we grouped compositions with element concentrations differing by <2 at.% across all components and calculated appearance frequency and coefficient of variation (CV%). All five property intervals demonstrated ≥80% appearance frequency and CV% <3%, indicating that the Pareto frontier represents reproducible physical trade-offs rather than stochastic optimization artifacts. Approximately 85% of optimal compositions identified in any single run also appeared in at least 8 out of 10 runs, confirming robust convergence.

**Supplementary Table 8 Compositions of the four experimental validation alloys (at.%).** Exact elemental compositions of alloys #1 (8-element), #2 (7-element), #3 (6-element), and #4 (5-element) determined by NSGA-II multi-objective optimization.

| Alloy | Elements | Nb | Si | Ti | Al | Cr | Hf | Zr | V |
|---|---|---|---|---|---|---|---|---|---|
| #1 | 8-element | 44.01 | 12.26 | 21.35 | 1.98 | 1.96 | 0.51 | 4.34 | 4.35 |
| #2 | 7-element | 45.48 | 15.06 | 26.16 | 1.97 | 1.98 | 0.71 | 4.64 | - |
| #3 | 6-element | 54.40 | 14.51 | 25.72 | 1.98 | 1.87 | 0.52 | - | - |
| #4 | 5-element | 48.06 | 12.59 | 35.00 | 1.77 | 2.58 | - | - | - |

Note: Compositions were determined by NSGA-II multi-objective optimization. Alloys #2 and #4 contain Ti concentrations (26.16 at.% and 35 at.%, respectively) that exceed the typical training set range (0-24 at.%, see Supplementary Fig. 1c).

**Supplementary Table 9 Predicted and experimental $K_Q$ and HTC for the four validation alloys.** Predicted values, experimental measurements, and prediction errors for alloys #1–#4; all prediction errors are within 5%, validating the reliability of the ML models.

| Alloy | $K_Q$ | | HTC | | $K_Q$ Prediction Error | HTC Prediction Error |
|---|---|---|---|---|---|---|
| | Predicted | Experimental | Predicted | Experimental | | |
| #1 | 18.71 | 18.92 | 12.26 | 21.35 | -1.11% | +1.44% |
| #2 | 15.39 | 14.94 | 15.06 | 26.16 | +3.01% | +1.26% |
| #3 | 16.24 | 15.61 | 14.51 | 25.72 | +4.04% | +2.06% |
| #4 | 15.04 | 15.71 | 12.59 | 35.00 | -4.26% | +1.26% |

**Supplementary Table 10 Microstructural parameters for experimentally characterized Nb-Si alloys.** Nbss vol.%, silicide vol.%, Nbss phase size (μm), and silicide phase size (μm) for alloys with available microstructural data.

| alloy | Nbss vol.% | Silicide vol.% | Nbss phase size (μm) | Silicide phase size (μm) |
|---|---|---|---|---|
| alloy14 | 10.7 | 15.6 | - | - |
| alloy15 | 26.2 | 16.3 | - | - |
| alloy16 | 29.7 | 16.7 | - | - |
| alloy17 | 33.1 | 17.1 | - | - |
| alloy26 | 51 | 49 | - | - |
| alloy27 | 43 | 57 | - | - |
| alloy39 | 40.1 | 59.2 | - | - |
| alloy40 | 59.65 | 40.35 | - | - |
| alloy41 | 44.8 | 55.2 | - | - |
| alloy46 | 54.71 | 45.29 | 5.18 | 11.26 |
| alloy47 | 61.8 | 38.2 | 6.1 | 4 |
| alloy48 | 53.89 | 46.11 | 4.63 | 11.92 |
| alloy49 | 57.19 | 42.81 | 5.71 | 25.59 |
| alloy50 | 55.03 | 44.97 | 5.78 | 22.91 |
| alloy51 | 64.49 | 35.51 | 6.76 | 12.73 |
| alloy52 | 57.47 | 42.53 | 6.49 | 4.92 |
| alloy53 | 34.7 | 65.3 | 10 | 8.4 |
| alloy54 | 49.4 | 50.6 | 8.5 | 7.2 |
| alloy55 | 37.8 | 62.2 | 13.9 | 12.8 |
| alloy56 | 58.6 | 41.4 | 5.8 | 4.1 |
| alloy57 | 55.4 | 44.6 | 6.1 | 3.8 |
| alloy58 | 51.1 | 48.9 | 7.2 | 6.2 |
| alloy59 | 47.8 | 52.2 | 4.4 | 3.7 |
| alloy60 | 57.2 | 42.8 | 6.8 | 7.1 |
| alloy61 | 57.3 | 42.7 | 4.7 | 4.6 |
| alloy62 | 31.2 | 68.8 | 3.9 | 4.5 |
| alloy63 | 7.6 | 92.4 | 3.6 | 6.4 |
| alloy64 | 34.6 | 65.4 | - | - |
| alloy65 | 55.2 | 44.8 | - | - |
| alloy66 | 47.3 | 52.7 | - | - |
| alloy67 | 33.8 | 66.2 | - | - |
| alloy68 | 56.7 | 43.3 | - | - |
| alloy69 | 60.6 | 39.4 | - | - |
| alloy70 | 63.1 | 36.9 | - | - |
| alloy71 | 62.5 | 37.5 | - | - |
| alloy72 | 44 | 48 | - | - |
| alloy73 | 47 | 53 | - | - |
| alloy74 | 53 | 47 | - | - |
| alloy75 | 54 | 46 | - | - |
| alloy76 | 58 | 42 | - | - |

| | | | | |
|---|---|---|---|---|
| alloy84 | 42 | 58 | - | - |
| alloy85 | 38 | 62 | - | - |
| alloy86 | 40 | 60 | - | - |
| alloy87 | 36 | 64 | - | - |
| alloy88 | 35 | 65 | - | - |
| alloy89 | 43 | 57 | - | - |
| alloy90 | 31 | 69 | - | - |
| alloy91 | 48.62 | 51.38 | - | - |
| alloy92 | 44.06 | 55.94 | - | - |
| alloy93 | 63.36 | 36.64 | - | - |
| alloy94 | 46.11 | 53.89 | - | - |
| alloy95 | 55.59 | 44.41 | - | - |
| alloy96 | 50.12 | 49.88 | - | - |
| alloy97 | 61.2 | 38.8 | - | - |
| alloy98 | 66.9 | 33.1 | - | - |
| alloy99 | 54.69 | 45.31 | - | - |
| alloy101 | 62.26 | 37.74 | - | - |
| alloy110 | 57.1 | 42.9 | - | - |
| alloy111 | 52.4 | 47.6 | - | - |
| alloy112 | 50.6 | 49.4 | - | - |
| alloy113 | 71.2 | 28.8 | - | - |
| alloy114 | 64.8 | 35.2 | - | - |
| alloy115 | 69.4 | 30.6 | - | - |
| alloy116 | 71.9 | 28.1 | - | - |
| alloy117 | 60 | 40 | - | - |
| alloy118 | 56 | 44 | - | - |
| alloy119 | 59 | 41 | - | - |
| alloy120 | 58 | 42 | - | - |
| alloy121 | 47.5 | 52.5 | - | - |
| alloy122 | 50.6 | 49.4 | - | - |
| alloy123 | 51.8 | 48.2 | - | - |
| alloy124 | 53.7 | 46.3 | - | - |
| alloy153 | 68.5 | 31.5 | - | - |
| alloy161 | - | - | 27.1535 | - |
| alloy163 | - | - | 23.7278 | - |
| alloy166 | - | - | 21.5769 | - |
| alloy193 | 21.91 | 78.09 | 22.1 | - |
| alloy194 | 27.29 | 72.71 | 18.77 | - |
| alloy195 | 30.72 | 69.28 | 26.9 | - |
| alloy201 | 43.85 | 56.15 | - | - |
| alloy203 | 63.18 | 30.64 | - | - |

Note: "-" indicate unavailable measurements.

**Supplementary Table 11 Regression results for ML descriptors against microstructural parameters.** Sample size (n), number of statistically significant features ($p < 0.05$), strongest descriptor, Pearson r, p-value, and regression equation ($y = ax + b$) for $K_Q$ and HTC model descriptors regressed against Nbss vol.%, silicide vol.%, Nbss phase size, silicide phase size, and lattice mismatch $\delta$.

| Model | Target Variable | n | Sig. Features (p<0.05) | Strongest Feature | r | p-value | Regression Equation |
|---|---|---|---|---|---|---|---|
| $K_Q$ | Nbss vol.% | 78 | 1/6 | ΔHmix | -0.386 | <0.001 | $y = -5.70x - 128.13$ |
| $K_Q$ | Silicide vol.% | 78 | 5/6 | Ω | -0.477 | <0.001 | $y = -41.45x + 77.42$ |
| $K_Q$ | Nbss phase size | 24 | 4/6 | ΔHmix | 0.702 | <0.001 | $y = 5.29x + 175.80$ |
| $K_Q$ | Silicide phase size | 18 | 0/6 | - | - | ns | No significant relation |
| $K_Q$ | Lattice mismatch $\delta$ | 17 | 3/5 | electrophilicity index (M-B3) | 0.814 | <0.001 | $y = 35.71x - 35.10$ |
| HTC | Nbss vol.% | 78 | 1/6 | Thermal expansion (M-F6) | -0.278 | <0.05 | $y = -1.1\times10^{7}x + 124.90$ |
| HTC | Silicide vol.% | 78 | 2/6 | Specific heat (M-F7) | -0.271 | <0.05 | $y = -143.28x + 103.49$ |
| HTC | Nbss phase size | 24 | 5/6 | VEC | 0.780 | <0.001 | $y = -211.91x + 95.32$ |
| HTC | Silicide phase size | 18 | 0/6 | - | - | ns | No significant relation |
| HTC | Lattice mismatch $\delta$ | 17 | 5/6 | M-F7 | −0.684 | <0.01 | $y = -5\times10^{6}x + 50.12$ |

Note: For $K_Q$ model regression against lattice mismatch $\delta$, M-F4 (melting enthalpy) was excluded due to unavailable values for carbon (C)-containing alloys, leaving 5 descriptors for analysis.

**Supplementary Table 12 Comparison of ML and physics-based HTC predictions.** Theoretical HTC from the Labusch–Nabarro solid solution strengthening model, ML predictions, and experimental values for alloys #1-#4; model errors and ML errors shown as percentages. The physics-based model underestimates by 10–15% due to single-mechanism limitation, while the ML model achieves <5% error by capturing multi-mechanism synergies.

| Alloy | Solid Solution Model (MPa) | ML Prediction (MPa) | Experimental (MPa) | Model Error (%) | ML Error (%) |
|---|---|---|---|---|---|
| #1 | 287.93 | 325.67 | 322 | -10.63 | +1.44 |
| #2 | 312.65 | 361.12 | 356.64 | -12.34 | +1.26 |
| #3 | 298.77 | 278.33 | 272.67 | +9.63 | +2.06 |
| #4 | 292.77 | 259.49 | 256.57 | +14.12 | +1.26 |

Note: To provide a quantitative baseline for comparison with ML predictions, we calculated the theoretical high-temperature compressive strength using the Labusch-Nabarro solid solution strengthening model. The total strengthening contribution is expressed as:

$$\Delta\sigma_{ss}=\left(\sum(\Delta\sigma_i)^{3/2}\right)^{2/3} \tag{S1}$$

where the individual element contribution is:

$$\Delta\sigma_i=AGf_i^{4/3}c_i^{2/3} \tag{S2}$$

Here $A$ = 0.02 is the strengthening constant, $G$ is the shear modulus of element i, $c_i$ is its atomic fraction, and $f_i$ is the misfit parameter defined as:

$$f_i=\sqrt{\delta_{G,\,i}^2+\alpha^2\delta_{r,\,i}^2} \tag{S3}$$

where $\alpha$ = 9 is the dislocation parameter, and $\delta_{G,\,i}$ and $\delta_{r,\,i}$ are the shear modulus and atomic radius mismatch parameters calculated as:

$$\delta_{G,\,i}=\frac{9}{8}\sum c_j\delta_{G,ij} \tag{S4}$$

$$\delta_{r,\,i}=\frac{9}{8}\sum c_j\delta_{r,ij} \tag{S5}$$

where $G_i$, $G_j$ are the shear moduli and $r_i$, $r_j$ are the atomic radii for elements $i$ and $j$, respectively. The overall mismatch parameters for element $i$ are then obtained by weighted averaging over all alloying elements:

$$\delta_{G,ij}=\frac{2(G_i\text{-}G_j)}{(G_i+G_j)} \tag{S6}$$

$$\delta_{r,ij}=\frac{2(r_i\text{-}r_j)}{(r_i+r_j)} \tag{S7}$$

where cj is the atomic fraction of element $j$, and the factor 9/8 accounts for the anisotropic elastic effects in the solid solution.

Room-temperature elemental properties (shear modulus, atomic radius, yield strength) were obtained from standard databases (Supplementary Tables 18-21). To account for high-temperature effects at 1250°C (1523 K), we applied a unified reduction factor of 0.25 to all mechanical properties based on the reported modulus degradation behavior of refractory alloys. The intrinsic matrix strength was calculated as $\sigma_0 = \sum_i (c_i \cdot \sigma_{0.2i})$, where $\sigma_{0.2i}$ is the yield strength of element $i$. The theoretical yield strength was then determined as $\sigma_{y,theory} = \sigma_0 + \Delta\sigma_{ss}$. This physics-based calculation provides a single-mechanism baseline that does not account for phase transformation, grain boundary, or precipitate strengthening effects.

**Supplementary Table 13 Hall–Petch-type $K_Q$ predictions for the four validation alloys.** Grain size d (μm), predicted $K_{IC}$, experimental $K_{IC}$, and prediction error (%) for alloys #1-#4.

| Alloy | d (μm) | $k \cdot d^{-\frac{1}{2}}$ (MPa·m$^{1/2}$) | $K_{IC}$ pred (MPa·m$^{1/2}$) | $K_{IC}$ exp (MPa·m$^{1/2}$) | Error (%) |
|---|---|---|---|---|---|
| #1 | 58 | 7.88 | 17.88 | 18.92 | 5.5 |
| #2 | 48 | 8.66 | 18.66 | 14.94 | 24.9 |
| #3 | 72 | 7.07 | 17.07 | 15.61 | 9.4 |
| #4 | 65 | 7.44 | 17.44 | 15.71 | 11.0 |

Note: $K_0$ = 10 MPa·m$^{1/2}$ estimated from rule-of-mixtures[1]; k = 60 MPa·m$^{1/2}$·μm$^{0.5}$ estimated considering phase boundary dilution effects[2].

**Supplementary Table 14 Phase-based model parameters for Griffith and constrained mixture models.** Nbss volume fraction (Vd), $Nb_5Si_3$ volume fraction (Vb), elastic moduli of Nbss ($E_{Nbss}$) and $Nb_5Si_3$ ($E_{Nb5Si3}$), and effective modulus ($E_{eff}$) for alloys #1–#4, used as input parameters for Griffith and constrained mixture $K_{IC}$ predictions.

| Alloy | Vd (Nbss %) | Vb ($Nb_5Si_3$%) | $E_{Nbss}$ (GPa) | $E_{Nb5Si3}$(GPa) | $E_{eff}$ (GPa) |
|---|---|---|---|---|---|
| #1 | 84.33 | 15.67 | 136.4 | 165.5 | 141.0 |
| #2 | 82.38 | 17.62 | 140.7 | 192.5 | 149.8 |
| #3 | 82.57 | 17.43 | 135.2 | 177.8 | 142.6 |
| #4 | 83.98 | 16.02 | 134.6 | 178.1 | 141.5 |

Note: $E_{eff}$ calculated as $Vd \cdot E_{Nbss} + Vb \cdot E_{Nb5Si3}$.

**Supplementary Table 15 Griffith model $K_{IC}$ calculations for the four validation alloys.** Effective modulus $E_{eff}$ (GPa), effective surface energy $\gamma_{eff}$ ($J/m^2$), predicted $K_{IC}$ ($MPa \cdot m^{1/2}$), experimental $K_{IC}$, and prediction error (%) for alloys #1-#4.

| Alloy | $E_{eff}$ (GPa) | $\gamma_{eff}$ ($J/m^2$) | $K_{IC}$ pred ($MPa \cdot m^{1/2}$) | $K_{IC}$ exp ($MPa \cdot m^{1/2}$) | Error(%) |
|---|---|---|---|---|---|
| #1 | 141.0 | 2.91 | 9.11 | 18.92 | 51.8 |
| #2 | 149.8 | 2.93 | 9.37 | 14.94 | 37.3 |
| #3 | 142.6 | 2.93 | 9.15 | 15.61 | 41.4 |
| #4 | 141.5 | 2.91 | 9.09 | 15.71 | 42.1 |

Note: $\gamma_{Nb}$ = 2.7 $J/m^2$; $\gamma_{Nb5Si3}$ = 4.0 $J/m^2$ estimated from Miedema model with covalent bonding correction, analogous to $Mo_5Si_3$[3,4]; $\nu$ = 0.38.

**Supplementary Table 16 Constrained mixture model KIC calculations for the four validation alloys.** Brittle phase volume fraction (Vb), constraint factor Pc, toughness ratio $[Kd/Kb]^2$, predicted KIC (MPa·$m^{1/2}$), experimental $K_{IC}$, and prediction error (%) for alloys #1-#4; constraint parameter.

| Alloy | Vb | Pc | $\left[\frac{Kd}{Kb}\right]^2 Pc$ | $K_{IC}$ pred | $K_{IC}$ exp | Error (%) |
|---|---|---|---|---|---|---|
| #1 | 0.1567 | 0.6093 | 69.33 | 17.07 | 18.92 | 9.8 |
| #2 | 0.1762 | 0.5653 | 64.32 | 16.36 | 14.94 | 9.5 |
| #3 | 0.1743 | 0.5695 | 64.80 | 16.43 | 15.61 | 5.3 |
| #4 | 0.1602 | 0.6013 | 68.41 | 16.94 | 15.71 | 7.8 |

Note: Kb = 3.0 MPa·$m^{1/2}$, Kd = 32.0 MPa·$m^{1/2}$, q' = $1.0^2$.

**Supplementary Table 17 Variance decomposition for the $K_Q$ dataset.** Sources of variation, estimated variance $\sigma$ (MPa·m$^{1/2}$), and percentage contribution to total $K_Q$ variance for compositional factors.

| Source of Variation | Variance $\sigma$ (MPa·m$^{1/2}$) | Percentage (%) | Combined Magnitude | Estimation Method |
|---|---|---|---|---|
| Compositional factors | 3.03 | ~96% | - | Test $R^2$ = 0.96 |
| Processing variations | 0.4-0.5 | 3-4% | - | Residual analysis |
| Measurement uncertainties | ~0.2 | ~1% | - | Remainder from metrics |
| Processing + Measurement | 0.62 | ~4% | 0.5-0.7 | $\sigma_{unexplained}$ |
| Total (observed) | 3.09 | 100% | - | Standard deviation |

**Supplementary Table 18 Elemental features for the $K_Q$ model.** Complete list of 38 elemental features with feature codes, descriptions, and units, categorized by property type (atomic, electronic, thermodynamic, physical transport, mechanical, and structural characteristics), used as the basis for constructing alloy-level descriptors for $K_Q$ prediction.

| feature code | feature description | unit |
|---|---|---|
| A1 | Atomic number | - |
| A2 | Atomic weight | g/mol |
| A3 | Density | g/cm$^3$ |
| B1 | Electronegativity (Pauling) | - |
| B2 | Electron affinity | kJ/mol |
| B3 | Electrophilicity Index | eV |
| C1 | Radii covalent | pm |
| C2 | Covalent radius Pyykko(Single Bond) | pm |
| C3 | Covalent radius Pyykko(Double Bond) | pm |
| C4 | Covalent radius Pyykko(Triple Bond) | pm |
| C5 | Radii atomic (coordination number 12) | pm |
| C6 | Metallic radius | pm |
| C7 | Metallic radius 12 Neighbors | pm |
| D1 | Mass Magnetic Susceptibility | m$^3$/kg |
| D2 | Molar Magnetic Susceptibility | m$^3$/mol |
| D3 | Volume Magnetic Susceptibility | - |
| D4 | Neutron Cross Section | barns |
| D5 | Neutron Mass Absorption | m$^2$/kg |
| E1 | Space Group Number | - |
| E2 | Glawe Number | - |
| E3 | Lattice Constants a | Å |
| E4 | Lattice Constants b | Å |
| E5 | Lattice Constants c | Å |
| F1 | Temperature melting | K |
| F2 | Temperature boiling | K |
| F3 | Enthalpy vaporization | kJ/mol |
| F4 | Enthalpy melting | kJ/mol |
| F5 | Enthalpy atomization | kJ/mol |
| F6 | Thermal expansion coefficient | K$^{-1}$ |
| F7 | Specific heat capacity | J/(g·K) |
| F8 | Molar heat capacity | J/(mol·K) |
| F9 | Molar volume | cm$^3$/mol |
| G1 | Thermal conductivity | W/(m·K) |
| G2 | Electrical resistivity | MS/m |
| G3 | Resistivity | μΩ·m |
| H1 | Young’s Modulus | Gpa |
| H2 | Mohs hardness | - |
| I1 | Speed of Sound | m/s |

**Supplementary Table 19 Elemental feature values for the $K_Q$ model.** Complete dataset of all 38 feature values for the 12 elements (Nb, Si, Ti, Al, Cr, Hf, Zr, Mo, V, W, Ta, Sn) used in $K_Q$ prediction.

| | Nb | Si | Ti | Al | Cr | Hf | Zr | Mo | V | W | Ta | Sn |
|---|---|---|---|---|---|---|---|---|---|---|---|---|
| A1 | 41 | 14 | 22 | 13 | 24 | 72 | 40 | 42 | 23 | 74 | 73 | 50 |
| A2 | 92.90637 | 28.085 | 47.867 | 26.98153 | 51.9961 | 178.49 | 91.224 | 95.95 | 50.9415 | 183.84 | 180.94788 | 118.71 |
| A3 | 8.57 | 2.33 | 4.507 | 2.7 | 7.19 | 13.31 | 6.511 | 10.28 | 6.11 | 19.25 | 16.65 | 7.31 |
| B1 | 1.6 | 1.9 | 1.54 | 1.61 | 1.66 | 1.3 | 1.33 | 2.16 | 1.63 | 2.36 | 1.5 | 1.96 |
| B2 | 86.1 | 133.6 | 7.6 | 42.5 | 64.3 | 0 | 41.1 | 71.9 | 50.6 | 78.6 | 31 | 107.3 |
| B3 | 1.261 | 1.683 | 0.884 | 0.927 | 1.132 | 0.858 | 1.004 | 1.211 | 1.062 | 1.336 | 1.072 | 1.434 |
| C1 | 164 | 111 | 160 | 121 | 139 | 175 | 175 | 154 | 153 | 162 | 170 | 139 |
| C2 | 147 | 116 | 136 | 126 | 122 | 152 | 154 | 138 | 134 | 137 | 146 | 140 |
| C3 | 125 | 107 | 117 | 113 | 111 | 128 | 127 | 121 | 112 | 120 | 126 | 130 |
| C4 | 116 | 102 | 108 | 111 | 103 | 122 | 121 | 113 | 106 | 115 | 119 | 132 |
| C5 | 198 | 111 | 176 | 118 | 166 | 208 | 206 | 190 | 171 | 193 | 200 | 145 |
| C6 | 134 | 117 | 132 | 125 | 119 | 144 | 145 | 130 | 122 | 130 | 134 | 142 |
| C7 | 146 | 138 | 147 | 143 | 128 | 159 | 160 | 139 | 134 | 139 | 146 | 163 |
| D1 | 27.6 | -1.6 | 40.1 | 7.8 | 44.5 | 5.3 | 16.8 | 11.7 | 62.8 | 4.59 | 10.7 | -3.1 |
| D2 | 2.56 | -0.0449 | 1.919 | 0.21 | 2.314 | 0.946 | 1.53 | 1.122 | 3.199 | 0.844 | 1.936 | -0.368 |
| D3 | 0.000237 | -0.00000373 | 0.0001807 | 0.0000211 | 0.0003177 | 0.0000705 | 0.000109 | 0.0001203 | 0.0003837 | 0.0000884 | 0.0001782 | -0.0000227 |
| D4 | 1.15 | 0.166 | 6.1 | 0.233 | 3.1 | 104 | 0.184 | 2.6 | 5.06 | 18.4 | 20.5 | 0.62 |
| D5 | 0.0004 | 0.0002 | 0.0044 | 0.03 | 0.0021 | 0.02 | 0.00066 | 0.0009 | 0.0033 | 0.0036 | 0.0041 | 0.0002 |
| E1 | 229 | 227 | 194 | 225 | 229 | 194 | 194 | 229 | 229 | 229 | 229 | 141 |
| E2 | 53 | 85 | 51 | 78 | 55 | 50 | 49 | 56 | 54 | 57 | 52 | 83 |
| E3 | 330.04 | 543.09 | 295.08 | 404.95 | 291 | 319.64 | 323.2 | 314.7 | 303 | 316.52 | 330.13 | 905.4 |
| E4 | 330.04 | 543.09 | 295.08 | 404.95 | 291 | 319.64 | 323.2 | 314.7 | 303 | 316.52 | 330.13 | 908.3 |
| E5 | 330.04 | 543.09 | 468.55 | 404.95 | 291 | 505.11 | 514.7 | 314.7 | 303 | 316.52 | 330.13 | 1160.1 |
| F1 | 2750 | 1687 | 1941 | 933.47 | 2180 | 2506 | 2128 | 2896 | 2183 | 3695 | 3290 | 505.08 |
| F2 | 5017 | 3173 | 3560 | 2792 | 2944 | 4876 | 4682 | 4912 | 3650 | 5828 | 5731 | 2875 |
| F3 | 680 | 383 | 422.6 | 284.1 | 342 | 575 | 567 | 590 | 460 | 824 | 758 | 296 |
| F4 | 26.8 | 50.6 | 18.8 | 10.75 | 21 | 25.1 | 19.2 | 28 | 17.5 | 35 | 24.7 | 7.07 |
| F5 | 733 | 450 | 473 | 330.9 | 397.48 | 618.4 | 610 | 658.98 | 515.5 | 851 | 782 | 301.2 |
| F6 | 0.0000073 | 0.0000026 | 0.0000086 | 0.0000231 | 0.0000049 | 0.0000059 | 0.0000057 | 0.0000048 | 0.0000084 | 0.0000045 | 0.0000063 | 0.000022 |

| | | | | | | | | | | | | |
|---|---|---|---|---|---|---|---|---|---|---|---|---|
| F7 | 0.265 | 0.712 | 0.523 | 0.897 | 0.449 | 0.144 | 0.278 | 0.251 | 0.489 | 0.132 | 0.14 | 0.227 |
| F8 | 24.6 | 19.789 | 25.06 | 24.2 | 23.35 | 25.73 | 25.36 | 24.06 | 24.89 | 24.27 | 25.36 | 27.112 |
| F9 | 10.841 | 12.054 | 10.621 | 9.99 | 7.2317 | 13.4102 | 14.011 | 9.334 | 8.3374 | 9.5501 | 10.8677 | 16.239 |
| G1 | 53.7 | 149 | 21.9 | 237 | 93.9 | 23 | 22.7 | 139 | 30.7 | 173 | 57.5 | 66.8 |
| G2 | 6.7 | 0.001 | 2.5 | 38 | 7.9 | 3.3 | 2.4 | 20 | 5 | 20 | 7.7 | 9.1 |
| G3 | 0.0000015 | 0.001 | 0.0000004 | 0.00000026 | 0.0000013 | 0.0000003 | 0.00000042 | 5E-08 | 0.0000002 | 5E-08 | 0.0000013 | 0.0000011 |
| H1 | 105 | 47 | 116 | 70 | 279 | 78 | 68 | 329 | 128 | 411 | 186 | 50 |
| H2 | 6 | 6.5 | 6 | 2.75 | 8.5 | 5.5 | 5 | 5.5 | 7 | 7.5 | 6.5 | 1.5 |
| I1 | 3480 | 2200 | 4140 | 5100 | 5940 | 3010 | 3800 | 6190 | 4560 | 5174 | 3400 | 2500 |

**Supplementary Table 20 Elemental features for the HTC model.** Complete list of 35 elemental features with feature codes, descriptions, and units, specifically selected for high-temperature compressive strength prediction.

| feature code | feature description | unit |
|---|---|---|
| A1 | Atomic number | - |
| A2 | Atomic weight | g/mol |
| A3 | Density | $g/cm^3$ |
| B1 | Electronegativity (Pauling) | - |
| B2 | Energy ionization first | kJ/mol |
| B3 | Electron affinity | kJ/mol |
| B4 | Electrophilicity Index | eV |
| C1 | Covalent radius Cordero | pm |
| C2 | Covalent radius (Pyykko single bond) | pm |
| C3 | Covalent radius (Pyykko single bond) | pm |
| C4 | Covalent radius (Pyykko single bond) | pm |
| C5 | Radii atomic (coordination number 12) | pm |
| C6 | Metallic radius | pm |
| C7 | Metallic radius (12 Neighbors) | pm |
| D1 | Neutron Cross Section | barns |
| D2 | Neutron Mass Absorption | $m^2/kg$ |
| E1 | Space Group Number | - |
| E2 | Glawe Number | - |
| E3 | Lattice Constants a | Å |
| E4 | Lattice Constants b | Å |
| E5 | Lattice Constants c | Å |
| F1 | Temperature melting | K |
| F2 | Temperature boiling | K |
| F3 | Enthalpy vaporization | kJ/mol |
| F4 | Enthalpy melting | kJ/mol |
| F5 | Enthalpy atomization | kJ/mol |
| F6 | Thermal expansion coefficient | $K^{-1}$ |
| F7 | Specific heat capacity | J/(g·K) |
| F8 | Molar heat capacity | J/(mol·K) |
| F9 | Molar volume | $cm^3/mol$ |
| G1 | Thermal conductivity | W/(m·K) |
| G2 | Electrical conductivity | MS/m |
| G3 | Electrical resistivity | μΩ·m |
| H1 | Mohs hardness | GPa |
| I1 | Speed of Sound | m/s |

**Supplementary Table 21 Elemental feature values for the HTC model.** Complete dataset of all 35 feature values for the 12 elements (Nb, Si, Ti, Al, Cr, Hf, Zr, Mo, Fe, B, V, Ge) used in HTC prediction.

| | Nb | Si | Ti | Al | Cr | Hf | Zr | Mo | Fe | B | V | Ge |
|---|---|---|---|---|---|---|---|---|---|---|---|---|
| A1 | 41 | 14 | 22 | 13 | 24 | 72 | 40 | 42 | 26 | 5 | 23 | 32 |
| A2 | 92.90637 | 28.085 | 47.867 | 26.98154 | 51.9961 | 178.49 | 91.224 | 95.95 | 55.845 | 10.81 | 50.9415 | 72.63 |
| A3 | 8.57 | 2.33 | 4.507 | 2.7 | 7.19 | 13.31 | 6.511 | 10.28 | 7.874 | 2.46 | 6.11 | 5.323 |
| B1 | 1.6 | 1.9 | 1.54 | 1.61 | 1.66 | 1.3 | 1.33 | 2.16 | 1.83 | 2.04 | 1.63 | 2.01 |
| B2 | 652.1 | 786.5 | 658.8 | 577.5 | 652.9 | 658.5 | 640.1 | 684.3 | 762.5 | 800.6 | 650.9 | 762 |
| B3 | 86.1 | 133.6 | 7.6 | 42.5 | 64.3 | 0 | 41.1 | 71.9 | 15.7 | 26.7 | 50.6 | 119 |
| B4 | 1.261 | 1.683 | 0.884 | 0.927 | 1.132 | 0.858 | 1.004 | 1.211 | 1.046 | 1.147 | 1.062 | 1.564 |
| C1 | 164 | 111 | 160 | 121 | 139 | 175 | 175 | 154 | 142 | 84 | 153 | 120 |
| C2 | 147 | 116 | 136 | 126 | 122 | 152 | 154 | 138 | 116 | 85 | 134 | 121 |
| C3 | 125 | 107 | 117 | 113 | 111 | 128 | 127 | 121 | 109 | 78 | 112 | 111 |
| C4 | 116 | 102 | 108 | 111 | 103 | 122 | 121 | 113 | 102 | 73 | 106 | 114 |
| C5 | 198 | 111 | 176 | 118 | 166 | 208 | 206 | 190 | 156 | 87 | 171 | 125 |
| C6 | 134 | 117 | 132 | 125 | 119 | 144 | 145 | 130 | 117 | 80 | 122 | 124 |
| C7 | 146 | 138 | 147 | 143 | 128 | 159 | 160 | 139 | 126 | 98 | 134 | 144 |
| D1 | 1.15 | 0.166 | 6.1 | 0.233 | 3.1 | 104 | 0.184 | 2.6 | 2.56 | 760 | 5.06 | 2.2 |
| D2 | 0.0004 | 0.0002 | 0.0044 | 0.03 | 0.0021 | 0.02 | 0.00066 | 0.0009 | 0.0015 | 2.4 | 0.0033 | 0.0011 |
| E1 | 229 | 227 | 194 | 225 | 229 | 194 | 194 | 229 | 229 | 166 | 229 | 225 |
| E2 | 53 | 85 | 51 | 78 | 55 | 50 | 49 | 56 | 71 | 86 | 54 | 84 |
| E3 | 330.4 | 543.09 | 295.08 | 404.95 | 291 | 319.64 | 323.2 | 314.7 | 286.65 | 506 | 303 | 565.75 |
| E4 | 330.4 | 543.09 | 295.08 | 404.95 | 291 | 319.64 | 323.2 | 314.7 | 286.65 | 506 | 303 | 565.75 |
| E5 | 330.4 | 543.09 | 468.55 | 404.95 | 291 | 505.11 | 514.7 | 314.7 | 286.65 | 506 | 303 | 565.75 |
| F1 | 2750 | 1687 | 1941 | 933.47 | 2180 | 2506 | 2128 | 2896 | 1811 | 2348 | 2183 | 1211.4 |
| F2 | 5017 | 3173 | 3560 | 2792 | 2944 | 4876 | 4682 | 4912 | 3134 | 4273 | 3650 | 3093 |
| F3 | 680 | 383 | 422.6 | 284.1 | 342 | 575 | 567 | 590 | 340 | 504.5 | 460 | 328 |
| F4 | 26.8 | 50.6 | 18.8 | 10.75 | 21 | 25.1 | 19.2 | 28 | 13.8 | 23.6 | 17.5 | 36.8 |
| F5 | 733 | 450 | 473 | 330.9 | 397.48 | 618.4 | 610 | 658.98 | 415.5 | 565 | 515.5 | 372 |
| F6 | 7.3E-06 | 2.6E-06 | 8.6E-06 | 2.31E-05 | 4.9E-06 | 5.9E-06 | 5.7E-06 | 4.8E-06 | 1.18E-05 | 0.000006 | 8.4E-06 | 0.000006 |
| F7 | 0.265 | 0.712 | 0.523 | 0.897 | 0.449 | 0.144 | 0.278 | 0.251 | 0.449 | 1.026 | 0.489 | 0.32 |
| F8 | 24.6 | 19.789 | 25.06 | 24.2 | 23.35 | 25.73 | 25.36 | 24.06 | 25.1 | 11.087 | 24.89 | 23.222 |
| F9 | 10.841 | 12.054 | 10.621 | 9.99 | 7.2317 | 13.4102 | 14.011 | 9.334 | 7.0923 | 4.3943 | 8.3374 | 13.645 |

| | | | | | | | | | | | | |
|---|---|---|---|---|---|---|---|---|---|---|---|---|
| G1 | 53.7 | 149 | 21.9 | 237 | 93.9 | 23 | 22.7 | 139 | 80.4 | 27.4 | 30.7 | 60.2 |
| G2 | 6.7 | 0.001 | 2.5 | 38 | 7.9 | 3.3 | 2.4 | 20 | 10 | 1E-10 | 5 | 0.002 |
| G3 | 1.5E-07 | 0.001 | 4E-07 | 2.6E-08 | 1.3E-07 | 3E-07 | 4.2E-07 | 5E-08 | 9.7E-08 | 10000 | 2E-07 | 0.0005 |
| H1 | 6 | 6.5 | 6 | 2.75 | 8.5 | 5.5 | 5 | 5.5 | 4 | 9.3 | 7 | 6 |
| I1 | 3480 | 2200 | 4140 | 5100 | 5940 | 3010 | 3800 | 6190 | 4910 | 16200 | 4560 | 5400 |

**Supplementary Table 22. Alloy-level descriptor definitions for Nb-Si alloys.** Phase parameters, formulae, and descriptions for 15 alloy-level features serving as input descriptors for both $K_Q$ and HTC models, enabling systematic exploration of composition–property relationships.

| Phase Parameter | Formula | Description |
|---|---|---|
| VEC | $\sum_{i=1}^{n} C_i \cdot \mathrm{VEC}_i$ | Valence electron concentration |
| $\chi$ | $\sum_{i=1}^{n} C_i \cdot \chi_i$ | Electronegativity |
| $\Delta\chi$ | $\sqrt{\sum_{i=1}^{n} c_i(\chi_i - \overline{\chi})^2}$ | Difference of electronegativity |
| $\Delta H_{mix}$ | $\sum_{\mathrm{i=1,j>i}}^{\mathrm{n}} 4\mathrm{C_i C_j} \cdot \Delta H_{ij}^{\mathrm{mix}}$ | Mixing enthalpy |
| $\Delta S_{mix}$ | $-R\sum_{i=1}^{n} C_i \cdot \ln{(C_i)}$ | Configuration entropy |
| $\Delta G_{mix}$ | $\Delta H_{mix} - T \cdot \Delta S_{mix}$ | Gibbs free energy of mixing |
| $\delta$ | $\sqrt{\sum_{\mathrm{i=1}}^{n} c_{\mathrm{i}}\left(1 - \frac{r_{\mathrm{i}}}{\overline{r}}\right)^2} \times 100$ | Atomic size difference |
| $\Lambda$ | $\frac{\Delta S_{\mathrm{mix}}}{\delta^2}$ | $\Lambda$ parameter |
| $T_m$ | $\sum_{i=1}^{n} C_i \cdot (T_m)_i$ | Melting temperature |
| $\Delta T_m$ | $\sqrt{\sum_{i=1}^{n} C_i \cdot [(T_m)_i - \overline{T_m}]^2}$ | Melting temperature mismatch |
| $\rho$ | $\sum_{i=1}^{n} C_i \cdot \rho_i$ | Density |
| $r$ | $\sum_{i=1}^{n} C_i \cdot r_i$ | Atomic radius |
| am | $\sum_{i=1}^{n} C_i \cdot (a_m)_i$ | Lattice parameter |
| $\Delta a$ | $\sqrt{\sum_{i=1}^{n} C_i \cdot (1 - \frac{(a_m)_i}{\overline{a_m}})^2}$ | Lattice parameter mismatch |

| | | |
|---|---|---|
| $\Omega$ | $\frac{T_m \Delta S_{mix}}{\lvert \Delta H_{mix} \rvert}$ | $\Omega$ parameter |

Note: $C_i$, $r_i$, $\chi_i$, $\text{VEC}_i$, $(T_m)_i$, $\rho_i$, and $(a_m)_i$ represent mole ratio, atomic radius, Pauling electronegativity, valence electron concentration, melting temperature, density, and lattice parameter of *i*th element respectively. $\bar{\chi}$, $\overline{T_m}$, $\bar{r}$ and $\overline{a_m}$ represent the mean value of Pauling electronegativity, melting temperature, atomic radius, and lattice parameter of all elements in Nb-Si based alloys. T and R represent absolute temperature and gas constant (8.314 $J \cdot mol^{-1} \cdot K^{-1}$). $\Delta H_{ij}^{\text{mix}}$ is the mixing enthalpy between elements *i* and *j*.

**Supplementary Table 23 Hyperparameter configurations for eight ML models.** Optimized hyperparameter settings for RF, XGBoost, GBDT, CatBoost, Bagging, DT, SVR, and ANN; identical feature sets and data splits used across all algorithms for fair comparison; ANN architecture selected via grid search and 5-fold cross-validation.

| parameter | RF | XGBoost | GBDT | CatBoost | Bagging | DT | SVR | ANN |
|---|---|---|---|---|---|---|---|---|
| Number of Trees/Neurous | 100 | 100 | 100 | 1000 | 10 | 1 | - | 450 |
| Learning Rate | - | 0.3 | 0.1 | 0.03 | - | - | - | 0.01 |
| Max Depth | None | 6 | 3 | 6 | None | None | - | 2 layers |
| Subsample Rate | 100% | 100% | 100% | 100% | 100% | - | - | 100% |
| Feature Sampling | √n_features | 100% | 100% | - | 100% | 100% | - | 100% |
| L2 Regularization | - | 1 | - | 3.0 | - | - | C=1.0 | $1\times10^{-5}$ |
| L1 Regularization | - | 0 | - | - | - | - | ε=0.1 | - |
| Kernel | Yes | - | - | Bayesian | Yes | - | RBF | - |

**Supplementary Table 24 ANN model configuration for $K_Q$ prediction.** Network architecture, optimization protocol, regularization strategies, training parameters, feature selection methodology, and NSGA-II optimization settings for the $K_Q$ ANN model (203 samples, 6 input features).

| Parameter Category | Parameter | Value | Description |
|---|---|---|---|
| Network Architecture | Input Layer | 6 neurons ($K_Q$) | Feature input dimensions |
| | Hidden Layer | 24 neurons + LayerNorm | ReLU activation, layer normalization |
| | Output Layer | 1 neuron | Property prediction ($K_Q$) |
| Optimization | Optimizer | Adam | Adaptive learning rate optimization |
| | Initial Learning Rate | 0.01 | Starting learning rate |
| | Beta Parameters | (0.9, 0.999) | Adam momentum parameters |
| | Epsilon | $1\times10^{-8}$ | Numerical stability |
| | Scheduler | CosineAnnealingLR | T_max=200, eta_min=$1\times10^{-6}$ |
| Regularization | L2 Weight Decay | $1\times10^{-5}$ | Prevents overfitting |
| | Batch Normalization | Momentum=0.1 | Layer normalization |
| | Early Stopping | Dual criteria | Train $R^2$>0.95, Test $R^2$>0.91 |
| Training Protocol | Loss Function | MSE | Mean squared error |
| | Batch Size | Full batch | Uses entire training set |
| | Data Split | 80:20 | Training:Testing ratio |
| | Random Seeds | Varied each iteration | Multiple independent splits |
| | Max Iterations | 1,000,000 | Typical convergence: 50k-150k |
| | Evaluation Frequency | Every 50 iterations | Rolling average window: 5 |
| Feature Selection | Initial Features | 200 | Element-derived descriptors |
| | PCC Filtering | \|r\|>0.95 removed | Reduces to 67 ($K_Q$), 65 (HTC) |
| | PCC-MIC Thresholds ($K_Q$) | PCC>0.2, MIC>0.2 | Retains 20 features |
| | RFE Parameters | ElasticNet base | alpha=0.01, l1_ratio=0.5 |
| | Final Features | 6 features | Selected by RFE performance |
| Preprocessing | StandardScaler | mean=0, std=1 | fit on training only |
| NSGA-II Optimization | Population Size | 500 | Number of individuals |
| | Max Generations | 250 | Minimum 20 generations |
| | Crossover | SBX, eta=15, rate=90% | Simulated binary crossover |
| | Mutation | Polynomial, eta=20, rate=10% | Polynomial mutation |
| | Objective Weights | Adjustable | Default: $K_Q$ 50%, HTC 50% |
| SHAP Analysis | Explainer | KernelExplainer | Model-agnostic interpretation |

| | | |
|---|---|---|
| Background Samples | 100 | Training data subset |

Note: This table combined with detailed text in the Methods section provides complete information for reproducing our results. We have also added Code Availability statement indicating full training code will be made available through GitHub upon publication.

**Supplementary Table 25 ANN model configuration for HTC prediction.** Network architecture, optimization protocol, regularization strategies, training parameters, feature selection methodology, and NSGA-II optimization settings for the HTC ANN model (34 samples, 6 input features); simplified architecture with lightweight Dropout regularization to address statistical robustness for the limited dataset.

| Parameter Category | Parameter | Value | Description |
|---|---|---|---|
| Network Architecture | Input Layer | 6 neurons (HTC) | Feature input dimensions |
| | Hidden Layer | 32 neurons + BatchNorm1d + Dropout(0.05) | ReLU activation, batch normalization, dropout |
| | Hidden Layer 2 | 16 neurons + BatchNorm1d + Dropout(0.02) | ReLU activation, batch normalization, dropout |
| | Output Layer | 1 neuron | Property prediction (HTC) |
| Optimization | Optimizer | Adam | Adaptive learning rate optimization |
| | Initial Learning Rate | 0.01 | Starting learning rate |
| | Beta Parameters | (0.9, 0.999) | Adam momentum parameters |
| | Epsilon | $1\times10^{-8}$ | Numerical stability |
| | Scheduler | CosineAnnealingLR | T_max=200, eta_min=$1\times10^{-6}$ |
| Regularization | L2 Weight Decay | $1\times10^{-5}$ | Prevents overfitting |
| | Batch Normalization | Momentum=0.1 | Layer normalization |
| | Early Stopping | Dual criteria | Train $R^2$>0.95, Test $R^2$>0.91 |
| | Dropout | Layer 1: 0.05, Layer 2: 0.02 | Additional regularization |
| Training Protocol | Loss Function | MSE | Mean squared error |
| | Batch Size | Full batch | Uses entire training set |
| | Data Split | 80:20:00 | Training:Testing ratio |
| | Random Seeds | Varied each iteration | Multiple independent splits |
| | Max Iterations | 1,000,000 | Typical convergence: 50k-150k |
| | Evaluation Frequency | Every 50 iterations | Rolling average window: 5 |
| Feature Selection | Initial Features | 200 | Element-derived descriptors |
| | PCC Filtering | \|r\|>0.95 removed | Reduces to 65 (HTC) |
| | PCC-MIC Thresholds (HTC) | PCC>0.09, MIC>0.08 | Adjusted for weaker correlations |
| | RFE Parameters | ElasticNet base | alpha=0.01, l1_ratio=0.5 |
| | Final Features | 6 features | Selected by RFE performance |
| Preprocessing | StandardScaler | mean=0, std=1 | fit on training only |
| NSGA-II | Population Size | 500 | Number of individuals |

| | | | |
|---|---|---|---|
| Optimization | | | |
| | Max Generations | 250 | Minimum 20 generations |
| | Crossover | SBX, eta=15, rate=90% | Simulated binary crossover |
| | Mutation | Polynomial, eta=20, rate=10% | Polynomial mutation |
| | Objective Weights | Adjustable | Default: $K_Q$ 50%, HTC 50% |
| SHAP Analysis | Explainer | KernelExplainer | Model-agnostic interpretation |
| | Background Samples | 100 | Training data subset |

Note: This table combined with detailed text in the Methods section provides complete information for reproducing our results. We have also added Code Availability statement indicating full training code will be made available through GitHub upon publication.

**Supplementary Table 26. Five-fold cross-validation results for the $K_Q$ model.** Training and test $R^2$, RMSE (MPa·$m^{1/2}$), and MAE (MPa·$m^{1/2}$) for each of five folds and overall statistics; 203 samples divided into five folds of 40–41 samples each.

| Fold | Training $R^2$ | Test $R^2$ | Training RMSE (MPa·$m^{1/2}$) | Test RMSE (MPa·$m^{1/2}$) | Training MAE (MPa·$m^{1/2}$) | Test MAE (MPa·$m^{1/2}$) |
|---|---|---|---|---|---|---|
| 1 | 0.967 | 0.952 | 0.78 | 0.91 | 0.59 | 0.72 |
| 2 | 0.971 | 0.931 | 0.73 | 1.06 | 0.55 | 0.78 |
| 3 | 0.969 | 0.946 | 0.76 | 0.88 | 0.57 | 0.65 |
| 4 | 0.965 | 0.938 | 0.81 | 0.98 | 0.61 | 0.71 |
| 5 | 0.973 | 0.927 | 0.71 | 1.01 | 0.54 | 0.74 |
| Mean | 0.969 | 0.939 | 0.76 | 0.97 | 0.57 | 0.72 |
| Std | 0.003 | 0.010 | 0.04 | 0.07 | 0.03 | 0.04 |

Note: To demonstrate model robustness and address concerns about overfitting, we implemented rigorous 5-fold cross-validation for $K_Q$ model. The dataset was divided into 5 equal folds, and each fold served as the test set once while the remaining 4 folds were used for training.

**Supplementary Table 27. Five-fold cross-validation results for the HTC model.** Training and test $R^2$, RMSE (MPa), and MAE (MPa) for each of five folds and overall statistics; 34 samples divided into five folds (folds 1–4: 7 samples each; fold 5: 6 samples); results confirm adequate generalization despite the limited sample size.

| Fold | Training $R^2$ | Test $R^2$ | Training RMSE (MPa) | Test RMSE (MPa) | Training MAE (MPa) | Test MAE (MPa) |
|---|---|---|---|---|---|---|
| 1 | 0.924 | 0.883 | 3.8 | 4.6 | 2.8 | 3.5 |
| 2 | 0.918 | 0.901 | 4.0 | 4.2 | 3.0 | 3.2 |
| 3 | 0.931 | 0.876 | 3.6 | 4.8 | 2.7 | 3.7 |
| 4 | 0.915 | 0.908 | 4.1 | 4.0 | 3.1 | 3.0 |
| 5 | 0.927 | 0.885 | 3.7 | 4.5 | 2.8 | 3.4 |
| Mean | 0.923 | 0.891 | 3.8 | 4.4 | 2.9 | 3.4 |
| Std | 0.007 | 0.013 | 0.2 | 0.3 | 0.1 | 0.2 |

Note: To demonstrate model robustness and address concerns about overfitting, we implemented rigorous 5-fold cross-validation for HTC model. The dataset was divided into 5 equal folds, and each fold served as the test set once while the remaining 4 folds were used for training.

## Supplementary Notes

### Supplementary Note 1: Processing Parameter Analysis and Variance Decomposition

To quantify processing homogeneity in the arc-melted dataset, we systematically extracted processing parameters from all 203 publications and performed correlation analysis and variance decomposition.

**(I) Processing Parameter Database**

We extracted nine key processing parameters from all 203 publications: remelting cycles, ingot mass, ingot diameter, ingot thickness, chamber argon pressure, protective atmosphere, cooling method, raw material purity, and melting current. Table S1 contains the complete database showing all documented parameters for each sample, while Table S2 presents summary statistics including documentation rates, ranges, means, medians, and most common values for each parameter.

Remelting cycles were documented for 194 samples (96% coverage), ranging from 3 to 8 cycles with mean 5.0 ± 0.8 and median of 5. Most samples (55%) used exactly 5 remelts, while 95% exceeded 4 remelts. Literature establishes that 4 remelts achieve >95% compositional homogeneity in niobium-silicide systems. Ingot geometry showed 72% documentation for diameter (146 samples) and 71% for thickness (145 samples). The distribution is remarkably uniform: 86% measured 33-35 mm diameter by 8-10 mm thickness, 11% were 66 mm by 23 mm, and only 3% were large ingots of 80-100 mm. Arc-melting universally employs water-cooled copper crucibles, though only 10% of publications explicitly state this standard practice.

Ingot mass was documented for 174 samples (86% coverage), with nominal range 65-2500 g but clustering around 100-150 g (66% of values). Mean mass is 187 ± 313 g with median 110 g. Chamber argon pressure was recorded for 146 samples (72% coverage), ranging nominally from 0.03 to 0.5 MPa. However, 84% used 0.05 MPa and another 12% used 0.03 MPa, yielding median 0.05 MPa. All samples used argon protective atmosphere (198 samples explicitly stated, 98% documentation).

Raw material purity was documented for 77 samples (38% coverage), predominantly 99.95% (73% of documented values) with median 99.95%. Melting current was documented for 77 samples (38% coverage), mostly 300-600 A (97% of documented values). Cooling method showed universal consistency with all 203 samples using water-cooled copper crucibles. Oxygen contamination was not directly documented (0% coverage), but high-purity argon atmosphere combined with positive chamber pressure effectively excludes oxygen ingress, with levels estimated at 50-200 ppm based on similar arc-melting protocols.

**(II) Correlation Analysis**

We performed statistical correlation analysis and variance decomposition to quantify how processing differences contribute to property variation. Pearson correlation analysis between each documented processing parameter and fracture toughness reveals no statistically significant relationships except for raw material purity (r=−0.322, p=0.004), as detailed in Fig. S34. The remaining six parameters exhibited weak correlations: remelting cycles (Fig. S34a), ingot mass (Fig. S34b), ingot diameter (Fig. S34c), ingot thickness (Fig. S34d), argon pressure (Fig. S34e), and melting current (Fig. S34g) all showed $p > 0.05$.

We quantified the magnitude through variance decomposition. The total $K_Q$ variance is $\sigma_{total}$= 3.09 MPa·m$^{1/2}$. The machine learning model achieves test $R^2 = 0.96$ (train $R^2 = 0.98$) with cross-validation metrics of RMSE = 0.88 MPa·m$^{1/2}$ (train 0.89) and MAE = 0.61 MPa·m$^{1/2}$ (train 0.61). Using test $R^2$ for conservative estimation, the explained variance is: $\sigma_{comp}=\sqrt{R^2\times\sigma_{total}^2}=3.03\text{MPa}\cdot\text{m}^{1/2}$. The unexplained variance is $\sigma_{unexplained}=\sqrt{(1\text{-}R^2)\times\sigma_{total}^2}=0.62\text{MPa}\cdot\text{m}^{1/2}$ which encompasses processing variations and measurement uncertainties.

Within-group analysis confirms limited processing contribution. Among samples with ≥4 remelting cycles (n=185, 95.6% of dataset), $K_Q$ standard deviation is 3.05 MPa·m$^{1/2}$ compared to 3.09 MPa·m$^{1/2}$ for the full dataset, indicating remelting variations contribute <4%. Standard small ingots (n=145, 71.9%) show $K_Q$ standard deviation of 3.08 MPa·m$^{1/2}$. Given these consistent processing conditions, we estimate

processing variations contribute approximately 0.4-0.5 MPa·m$^{1/2}$ (3-4% of total variance), with measurement uncertainties accounting for ~0.2 MPa·m$^{1/2}$ (~1%), yielding combined processing and measurement contribution of 0.5-0.7 MPa·m$^{1/2}$ (approximately 4% of total variance). Table S17 documents this variance decomposition showing compositional factors dominate at 3.03 MPa·m$^{1/2}$ (96%) while processing and measurement combined contribute 0.5-0.7 MPa·m$^{1/2}$ (4%).

**(III) limitation statement**

The training dataset exhibits relatively consistent processing: 95% of samples received ≥4 remelting cycles (median 5), 86% used small ingot geometry (33-35 mm diameter, 8-10 mm thickness) yielding cooling rates of 1.2-1.6 K/s, 84% employed 0.05 MPa argon pressure, 100% used argon atmosphere and water-cooled copper crucible solidification, and documented samples showed predominantly 99.95% raw material purity (median value). These conditions correspond to established compositional homogeneity (>95% from 4+ remelts) and controlled oxygen contamination (estimated 50-200 ppm).

Model predictions are reliable when applied to alloys processed under conditions consistent with this training dataset. However, applicability becomes uncertain under deviations: samples with <4 remelting cycles may exhibit compositional inhomogeneity (>5% local variation) not captured by the composition-based model; cooling rates substantially faster (>3 K/s from rapid quenching) or slower (<0.3 K/s from large ingots or furnace cooling) may alter grain size, silicide morphology, and phase distribution in ways not reflected in bulk composition; oxygen contamination exceeding 300 ppm (from inadequate chamber sealing, low argon purity, or vacuum melting without proper gettering) can introduce brittle oxide phases and reduce toughness beyond training experience; alternative processing routes including powder metallurgy, directional solidification, or additive manufacturing produce fundamentally different microstructures not represented in the arc-melted training dataset.

For experimental validation, we recommend processing conditions matching the

training distribution: perform ≥4 remelting cycles (preferably 5-6) to ensure compositional homogeneity, use water-cooled copper crucible arc-melting with argon atmosphere at 0.04-0.06 MPa chamber pressure, employ ingot dimensions of 30-40 mm diameter and 8-12 mm thickness to achieve 1-2 K/s cooling rates, use raw materials with minimum 99.5% purity, and maintain oxygen contamination below 200 ppm through proper chamber preparation and high-purity argon (≥99.999%). Under these conditions, model predictions exhibit uncertainty of approximately ±1.0 $MPa\cdot m^{1/2}$ from natural compositional scatter and residual processing variations.

**Supplementary Note 2: Feature Selection Validation and Robustness**

To validate the feature selection pipeline and address concerns about potential bias from linear filtering, we implemented four independent validation approaches.

**(I) Permutation Importance Analysis and SHAP-based pruning**

Permutation importance validates that all six selected features genuinely contribute to the final trained model by measuring performance degradation when each feature is randomly shuffled. We performed 100 random permutations per feature on the test set for both $K_Q$ and HTC models.

For the $K_Q$ model, Fig. S3a shows all six features demonstrate positive importance with statistical significance ($p < 0.001$ for all features based on t-tests across 100 permutations). The importance values are Ω (1.36), M-F4 (0.80), M-B3 (0.51), am (0.48), Δχ (0.25), and ΔHmix (0.14). For the HTC model, Fig. S3b shows similar results with M-F6 (2.95), V-B2 (0.89), VEC (0.79), M-C4 (0.56), M-F7 (0.23), and am (0.11), all achieving $p < 0.001$. The consistent positive importance values across all features confirm that none are redundant and each makes a real contribution to prediction accuracy.

SHAP-based pruning provides independent validation by analyzing the complete feature space without any pre-filtering. We trained gradient boosting models using all features (103 for $K_Q$ and 97 for HTC) and computed SHAP values across all training samples. Features were ranked by mean absolute SHAP value without sequential elimination steps. For the $K_Q$ model, Fig. S3c shows all six RFE-selected features appear within the top 20 positions among 103 features when ranked by SHAP importance, with mean SHAP values ranging from 0.38 to 0.11. For the HTC model, Fig. S3d shows all six selected features similarly appear in the top 20 SHAP rankings among 97 features, with values ranging from 6.50 to 1.57.

**(II) Performance Comparison With/Without RFE**

We performed systematic performance comparison between models with RFE versus models without RFE to isolate the effect of this final feature selection step. Both configurations started with the same pre-filtered feature set after PCC and MIC

screening, then diverged only in whether RFE was applied. All models used identical neural network architectures, training procedures, and hyperparameters to ensure fair comparison.

For the $K_Q$ model, Fig. S4a-d, e-h show training curves for the models, both models follow similar training trajectories. Performance comparison in Fig. S4i-l reveals that the with-RFE model achieves test $R^2$ of 0.96 and test RMSE of 1.50 MPa·$m^{1/2}$ compared to test $R^2$ of 0.93 and test RMSE of 1.71 MPa·$m^{1/2}$ for the without-RFE model. The with-RFE model shows improvement of 0.03 in $R^2$ and 0.21 MPa·$m^{1/2}$ in RMSE. Training time is comparable between configurations (9.06 s with RFE versus 9.94 s without RFE).

For the HTC model, performance metrics in Fig. S5i-l demonstrate that the with-RFE model achieves test $R^2$ of 0.98 and test RMSE of 3.19 compared to test $R^2$ of 0.99 and test RMSE of 3.79 for the without-RFE model. Although the without-RFE model shows slightly higher $R^2$ (0.99 vs 0.98), the with-RFE model achieves substantially better RMSE with improvement of 0.60. Training time remains comparable (1241.66 s with RFE versus 1273.09 s without RFE). These comparisons demonstrate that applying RFE as the final feature selection step maintains or improves predictive performance, validating that RFE effectively identifies the most informative features from the PCC-MIC pre-filtered set without sacrificing model accuracy.

**(III) Partial Dependence Analysis**

We conducted comprehensive partial dependence analysis to verify that no important nonlinear interactions were excluded during feature selection. For the $K_Q$ model, one-dimensional partial dependence analysis in Fig. S28a-f shows strong nonlinear effects for all six selected features. Fig. S28a shows M-F4 exhibits a steep increase in the 28-29 range followed by gradual decline. Fig. S28b shows am displays a sharp peak around 350-360 pm. Fig. S28c shows M-B3 demonstrates continuous increase. Fig. S28d shows $\Delta\chi$ has an optimal range around 15-20 with reduced toughness at extreme values. Fig. S28e shows $\Omega$ displays steady increase. Fig. S28f

shows ΔHmix has optimal performance at intermediate negative values around −20 to −25 kJ/mol. These nonlinear patterns confirm the features capture complex composition-property relationships.

Gradient analysis in Fig. S29a-f identifies regions where each feature has positive versus negative effects, with M-F4 showing strong positive gradients above 28.5 (Fig. S29a), am showing positive effects in 350-365 pm range (Fig. S29b), M-B3 exhibiting predominantly positive gradients (Fig. S29c), Δχ showing positive effects in 10-20 range (Fig. S29d), Ω displaying consistent positive gradients (Fig. S29e), and ΔHmix showing positive gradients in −15 to −25 kJ/mol range (Fig. S29f).

Two-dimensional partial dependence analysis in Fig. S30a-o reveals pairwise feature interactions across all 15 possible combinations of the six selected features. Non-rectangular contour patterns indicate genuine multiplicative interactions rather than simple additive effects. Fig. S30b shows M-F4 versus M-B3 interaction with a diagonal red zone where high toughness (21-24 $MPa·m^{1/2}$) requires both M-F4 around 28-29 and M-B3 above 1.26, demonstrating neither feature alone determines toughness. Fig. S30f shows am versus M-B3 interaction revealing peak toughness (22-25 $MPa·m^{1/2}$) only when am is 350-365 pm combined with M-B3 of 1.28-1.32. Fig. S30k shows M-B3 versus Ω interaction with curved boundaries confirming multiplicative effects where maximum toughness (21 $MPa·m^{1/2}$) requires Ω of 1.24-1.32 with M-B3 above 1.6. Fig. S30e shows M-F4 versus ΔHmix interaction demonstrating that intermediate ΔHmix values of −15 to −20 kJ/mol combined with M-F4 around 29 produce high toughness (17-19 $MPa·m^{1/2}$), while extreme values yield low toughness. The smooth continuous boundaries throughout Fig. S30a-o indicate genuine physical interactions rather than random noise.

To further quantify these interaction patterns, we applied Shapley Interaction Index (SII) analysis using the shapiq framework. Fig. S31a shows the strongest antagonistic interaction is between M-F4 and M-B3 (SII = −3.22), consistent with the constrained high-toughness region in the corresponding 2D PDP contour, while Δχ and M-B3 exhibit the largest synergistic interaction (SII = +1.37). Fig. S30b shows

the composition-sensitivity of each interaction, with M-F4 and M-B3 showing the largest standard deviation (Std =3.74). Fig. S31c confirms that the interactions are concentrated in a physically interpretable subset of feature pairs. The 2D PDP and SII analyses across all 15 feature pairs together confirm that the RFE-selected features retain all pairwise nonlinear interactions present in the full feature space, with no relevant interactions inadvertently excluded.

**(IV) Threshold Grid Search Validation**

We performed systematic grid search to determine optimal PCC and MIC thresholds and explain the large threshold difference between the two models. For the $K_Q$ model with 203 samples, we tested 361 threshold combinations with PCC ranging from 0.05 to 0.95 and MIC ranging from 0.05 to 0.95 at step size 0.05. For the HTC model with 34 samples, we tested 380 combinations with PCC ranging from 0.03 to 0.40 and MIC ranging from 0.02 to 0.40 at step size 0.02. Each combination underwent complete modeling including filtering, RFE selection, and neural network training with identical architecture to ensure fair comparison.

Grid search revealed markedly different optimal threshold landscapes for the two models. For the $K_Q$ model, Fig. S8a, c, and e show that stable performance occurs in the region with PCC ranging from 0.05 to 0.25 and MIC ranging from 0.15 to 0.25, where train $R^2$ remains around 0.96-0.97 and test $R^2$ varies between 0.87 and 0.97. Beyond PCC=0.65 or MIC=0.25, white regions with "--" indicate insufficient features after filtering. For the HTC model, Fig. S8b, d, and f show performance concentrated in a much narrower low-threshold region with PCC ranging from 0.03 to 0.09 and MIC ranging from 0.04 to 0.1, where train $R^2$ reaches 0.96-0.98 and test $R^2$ ranges from 0.97 to 0.99. Beyond PCC=0.15 or MIC=0.12, extensive white regions indicate insufficient features for valid model training. The viable performance region is substantially narrower for HTC than $K_Q$, demonstrating that permissive thresholds are essential for small sample sizes.

We quantitatively compared the top 5 threshold combinations for each model across multiple evaluation dimensions including $R^2$, RMSE, MAE, train-test

differences, and generalization ratios. For the $K_Q$ model, Fig. S9a, c, e, g, i, k show that the top-ranked combinations maintain train $R^2$ consistently at 0.96, with test $R^2$ ranging from 0.87 to 0.94, minimal $|\Delta R^2|$ values between 0.001 and 0.022, and generalization ratios (test/train for RMSE and MAE) ranging from 0.923 to 1.012. For the HTC model, Fig. S9b, d, f, h, j, l show train $R^2$ at 0.98-0.99 and test $R^2$ at 0.98-0.99, $|\Delta R^2|$ values between 0.008 and 0.021, and generalization ratios ranging from 0.874 to 1.017. These top-ranked combinations exhibit nearly identical comprehensive performance across all metrics, indicating that multiple threshold selections within the stable regions yield statistically equivalent results. Our selected thresholds (PCC=0.20, MIC=0.20 for $K_Q$; PCC=0.09, MIC=0.08 for HTC) fall within these validated high-performance regions.

The large threshold difference between models directly results from drastically different sample sizes and statistical constraints. With 203 samples, the standard error of Pearson correlation follows $SE(r)=\frac{1}{\sqrt{n-3}}$, giving SE=0.071 for the $K_Q$ model. The threshold PCC=0.20 represents 2.8 standard errors, providing reasonable confidence for preliminary screening. With only 34 samples, the HTC model faces SE=0.180, making even moderate correlations highly uncertain. The threshold PCC=0.09 represents 0.5 standard errors, which is deliberately permissive to preserve potentially informative features for subsequent RFE evaluation rather than prematurely discarding them based on unreliable correlation estimates. Statistical power analysis supports this interpretation. The power to detect correlation coefficient r at significance level α with sample size n follows:

$$\text{Power}=\Phi(z_r-z_{1-\alpha/2}) \tag{S8}$$

where $z_r = \frac{1}{2}\ln\left(\frac{1+r}{1-r}\right)\sqrt{n-3}$ is the Fisher Z-transformation and Φ is the cumulative standard normal distribution function.

For the $K_Q$ model, detecting r=0.20 with n=203 at α=0.05 yields power=0.74, which is acceptable for preliminary screening. For the HTC model, detecting r=0.09 with n=34 gives power=0.11, which is very low but unavoidable given severe sample

limitation. This limited power necessitates permissive initial filtering to retain more candidate features for subsequent RFE evaluation. Through systematic grid search evaluating 361 and 380 threshold combinations for $K_Q$ and HTC models respectively, we identified stable performance regions where our selected thresholds (PCC=0.20, MIC=0.20 for $K_Q$; PCC=0.09, MIC=0.08 for HTC) fall within the optimal stable regions, validating the rationale for threshold selection. The large threshold difference between the two models exists because the sample size disparity (n=203 versus n=34) creates different statistical constraints. The $K_Q$ model with larger sample size can use higher thresholds (PCC=0.20) while maintaining acceptable statistical power (0.74), whereas the HTC model with limited samples must use lower thresholds (PCC=0.09) to avoid prematurely discarding potentially informative features due to insufficient statistical power (0.11).

**Supplementary Note 3: Ablation Study**

To validate that each workflow component including feature selection, SHAP analysis, and NSGA-II optimization contributes essential functionality rather than redundant processing, we conducted systematic ablation studies quantifying the impact of omitting each component. This establishes the necessity of the integrated workflow.

**(I) Impact of Omitting Feature Selection**

For feature selection, we compared models with complete PCC-MIC-RFE screening versus models without this screening using identical neural network architectures. For SHAP analysis, we performed optimization with and without SHAP-derived element selection and design constraints. For NSGA-II, we compared multi-objective genetic algorithm optimization against random search baseline.

Omitting feature selection degrades model performance. For the $K_Q$ model, Fig. S6a shows test $R^2$ drops from 0.96 (with feature selection) to 0.91 (without feature selection), while Fig. S6e shows test RMSE increases from 1.47 to 1.50 MPa·$m^{1/2}$. Fig. S6b reveals the generalization gap widens with $R^2$ gap increasing from 0.04 to 0.06, and Fig. S6f shows RMSE gap increasing from 0.05 to 0.12. Fig. S6j demonstrates MAE gap increases from 0.03 to 0.15. Fig. S6m and Fig. S6q show the training process becomes significantly more unstable without feature selection. Fig. S6n illustrates the stark difference in dimensionality with 103 features retained without complete screening versus only 6 features after PCC-MIC-RFE, while Fig. S6r shows model complexity explodes from 241 parameters to 24,129 parameters without feature selection. For the HTC model, Fig. S6c shows test $R^2$ remains similar (0.98 with feature selection versus 0.99 without), but Fig. S6d reveals $R^2$ gap increases from 0.0084 to 0.0018. More critically, Fig. S6h shows RMSE gap improves substantially with feature selection (0.30 versus 0.31 without), and Fig. S6l shows MAE gap decreases dramatically from 0.35 to 0.08 with feature selection. Fig. S6p shows feature count reduction from 96 to 6, while Fig. S6t reveals model parameters decrease from 23,233 to 863.

**(II) Impact of Omitting SHAP Analysis**

Omitting SHAP analysis removes critical guidance for optimization. Model prediction accuracy remains identical because SHAP is a post-hoc interpretation tool not affecting training. However, optimization loses two essential capabilities. Without SHAP-based element selection, optimization explores the full 15-element space (Nb, Si, Ti, Al, Cr, Hf, Zr, V, Mo, W, Ta, Re, Fe, Ge, Sn) rather than focusing on beneficial elements. SHAP analysis ranked elements by weighted contributions using Score = $\Sigma(\alpha_i \times \lambda_i)$, where influence coefficients α derived from SHAP value-feature linear regression and weight coefficients λ reflect relative feature importance. This ranking identified Cr (12.08), Hf (13.16), Zr (13.39), and V (13.83) as optimal additions, with lower scores indicating stronger beneficial effects on both $K_Q$ and HTC. Without this screening, optimization includes potentially detrimental elements, reducing efficiency. Without SHAP-derived descriptor constraints, optimization operates in unconstrained 6-dimensional space without understanding favorable microstructural ranges. SHAP revealed that melting enthalpy descriptor M-F4 contributes positively below 28.5 and atomic radius mismatch am above 350 pm, translating to constraints preventing excessive silicide fractions. Optimization without these mechanistic constraints may yield compositions with reasonable predicted properties but unfavorable microstructures such as continuous brittle silicide networks.

**(III) Impact of Replacing NSGA-II with Random Search**

Replacing NSGA-II with random search produces substantially inferior optimization performance. Fig. S7a shows the comparison for 8-element alloys where random search generates widely scattered points across the entire $K_Q$-HTC space without structure, while NSGA-II concentrates evaluations along a clear Pareto front. Similar patterns appear in Fig. S7b for 7-element alloys, Fig. S7e for 6-element alloys, and Fig. S7f for 5-element alloys, consistently demonstrating NSGA-II's ability to identify the optimal trade-off frontier between the two objectives. For 8-element alloys, Fig. S7c shows three key results: in $K_Q$ convergence, NSGA-II reaches 20.5 MPa·$m^{1/2}$ after 100,000 evaluations while random search plateaus at 19.0 MPa·$m^{1/2}$; in HTC convergence, both methods achieve similar values around 520 MPa; for

computational cost, NSGA-II requires 129.23 seconds versus 4.81 seconds for random search. Although NSGA-II demands substantially longer computation time, this cost remains acceptable given the superior solution quality and complete Pareto front identification. Fig. S7d for 7-element alloys, Fig. S7g for 6-element alloys, and Fig. S7h for 5-element alloys show consistent patterns with computation times of 130.82s, 126.95s, and 127.67s for NSGA-II versus 4.82s, 4.23s, and 3.58s for random search. The key advantage lies not merely in finding better solutions but in generating complete Pareto fronts with well-distributed compositions allowing selection based on different $K_Q$-HTC trade-off preferences. Random search cannot identify this frontier structure and produces randomly scattered solutions potentially missing optimal trade-off regions.

The three components each address distinct challenges in the workflow. PCC-MIC-RFE reduces dimensionality from 103 (for $K_Q$) and 97 (for HTC) to 6 features, enabling efficient training and reliable predictions. SHAP screens beneficial elements from 15 candidates and provides mechanistic constraints guiding optimization toward favorable microstructures. NSGA-II efficiently explores composition space while identifying complete Pareto fronts for multi-objective optimization. Ablation studies demonstrate that each component plays an essential role, with omission of any single component degrading overall system performance.

**Supplementary Note 4: Quantitative Descriptor-Microstructure Relationships**

To establish quantitative links between ML-selected descriptors and physical microstructure, we collected microstructural data from published literature and performed systematic regression analyses between all six descriptors and each measured parameter.

The dataset includes Nbss vol.% and Silicide vol.% for 78 alloys, Nbss phase size for 24 alloys, and Silicide phase size for 18 alloys compiled in Table S10. For γ′ particle size, only 2 data points were identified across all experimentally investigated alloys, which is statistically insufficient for robust regression analysis. We will prioritize collecting more γ′ particle size data in future work. For interface strain fields, direct quantitative measurements are rarely reported in the Nb-Si alloy literature. Since lattice mismatch between phases is a well-established indicator of interface strain field intensity, we used the volumetric lattice mismatch $\delta$ between γ-$Nb_5Si_3$ and Nbss as an indirect measure, and collected $\delta$ values for 17 alloys with confirmed γ-$Nb_5Si_3$ phase presence. Complete regression results with correlation coefficients, p-values, and predictive equations are summarized in Table S11.

**(I) Results for model Descriptors**

For phase volume fractions with 78 alloy measurements, statistically significant correlations are established. In the $K_Q$ model, ΔHmix shows correlation with Nbss vol.% at r=0.386 and p<0.001 shown in Fig. S23a with regression equation $y$=-5.70$x$-128.13. The Ω parameter correlates with Silicide vol.% at r=-0.477 and p<0.001 shown in Fig. S23b with equation $y$=-41.45$x$+77.42. Fig. S23e confirms ΔHmix exhibits the strongest correlation with Nbss vol.% amongst all six $K_Q$ descriptors, while Fig. S23f confirms Ω exhibits the strongest correlation with Silicide vol.%. In the HTC model, M-F6 (thermal expansion coefficient) correlates with Nbss vol.% at r=-0.278 and p<0.05 shown in Fig. S23i and S23m, while M-F7 correlates with Silicide vol.% at r=-0.271 and p<0.05 shown in Fig. S23j and S23n. These regression equations enable predictive calculation where descriptors computed from composition yield phase fractions before experimental characterization.

For Nbss phase size with 24 measurements, statistically significant correlations are established in both models. In the $K_Q$ model, ΔHmix shows strong correlation at r=0.702 and p<0.001 shown in Fig. S23c with regression equation $y$=5.29$x$+175.80. Fig. S23g confirms ΔHmix exhibits the strongest correlation with Nbss phase size amongst all six $K_Q$ descriptors at r=0.702. In the HTC model, VEC shows strong correlation at r=0.780 and p<0.001 shown in Fig. S23k with equation $y$=-211.91$x$+95.32. Fig. S23o confirms VEC exhibits the strongest correlation with Nbss phase size amongst all six HTC descriptors at r=0.780. Silicide phase size with 18 measurements shows no statistically significant correlation with any descriptor in either model.

For lattice mismatch $\delta$ with 17 alloy measurements, statistically significant correlations are established in both models. In the $K_Q$ model, M-B3(electrophilicity index) shows the strongest correlation with δ at r=0.814 and p<0.001 shown in Fig. S23d with regression equation $y$=35.71$x$−35.10, and Fig. S23h confirms M-B3 has the highest Pearson r among all $K_Q$ descriptors. In the HTC model, M-F7 shows the strongest correlation with $\delta$ at r=−0.684 and p<0.01 shown in Fig. S23l with regression equation $y=-5\times10^6x+50.12$, and Fig. S22p confirms M-F7 has the highest absolute Pearson r among all HTC descriptors. These results show that the ML-selected descriptors in both models are closely related to the interface strain field intensity.

In response to the request for quantitative descriptor-microstructure relationships, we established statistically significant regression correlations for phase fractions (78 alloys), grain sizes (24 alloys), and interface strain fields (17 alloys, via lattice mismatch $\delta$), with predictive equations documented.

**(II) Three-Level Validation**

To integrate mechanistic insights into the ML framework, we developed three-level validation approach connecting ML feature importance through SHAP analysis, experimental microstructural correlations through regression, and physical theory through established thermodynamic principles.

For the $K_Q$ model, SHAP analysis ranks features with M-F4 showing highest contribution at 3.09, followed by am (2.72), M-B3 (1.96), Δχ (1.81), Ω (1.05), and ΔHmix (0.57). Regression against microstructural data confirms ΔHmix shows strongest correlation with Nbss vol.% at $r=-0.386$ and $p<0.001$ shown in Fig. S23a, Ω shows strongest correlation with Silicide vol.% at $r=-0.477$ and $p<0.001$ shown in Fig. S23b, and ΔHmix shows strong correlation with Nbss phase size at $r=0.702$ and $p<0.001$ shown in Fig. S23c. Furthermore, M-B3 shows the strongest correlation with lattice mismatch δ at $r=0.814$ and $p<0.001$ shown in Fig. S23d, confirming that this SHAP-identified important descriptor is also closely linked to interface strain field intensity. Fig. S23e-h confirm ΔHmix and Ω exhibit the strongest correlations among all six $K_Q$ descriptors for their respective microstructural parameters. Classical solution thermodynamics provides mechanistic explanation that ΔHmix quantifies thermodynamic driving force for Nb dissolution where more negative values stabilize higher Nbss fractions and influence grain growth kinetics, while Ω reflects liquidus-to-solidus temperature range ratio governing solidification path that controls eutectic silicide formation. The strong correlations between these descriptors and experimentally measured microstructural parameters establish that ML predictions operate through physically meaningful composition-microstructure pathways.

For the HTC model, SHAP analysis identifies M-F7 as highest contributor at 182.69, followed by M-C4 (121.27), am (101.67), M-F6 (46.15), VEC (28.08), and V-B2 (23.86). Regression confirms M-F6 correlates with Nbss vol.% at $r=-0.278$ and $p<0.05$ shown in Fig. S23i, M-F7 correlates with Silicide vol.% at $r=-0.271$ and $p<0.05$ shown in Fig. S23j, and VEC shows strongest correlation with Nbss phase size at $r=0.780$ and $p<0.001$ shown in Fig. S23k. In addition, M-F7 shows the strongest correlation with lattice mismatch $\delta$ at $r=-0.684$ and $p<0.01$ shown in Fig. S22l, providing further evidence that this top SHAP-ranked descriptor is linked to interface strain field intensity. Fig. S23m-p confirm M-F6, VEC, and M-F7 exhibit the strongest correlations of all HTC descriptors for Nbss vol.%, Nbss phase size, and both Silicide vol.% and lattice mismatch $\delta$, respectively. Thermal mechanics of

two-phase systems provides explanation that thermal expansion mismatch generates interfacial stress concentrations at elevated temperatures while relative heat capacity affects transient thermal gradients, and VEC influences grain boundary mobility through electronic structure effects on atomic bonding. The convergence between SHAP rankings and regression-confirmed correlations validates that the ML framework captures genuine links between composition and microstructure-dependent properties.

The three-level validation framework documented in Tables S10-S11 and Fig. S23 integrates mechanistic insights by establishing that regression-confirmed descriptor-microstructure correlations for phase fractions and phase sizes connect to SHAP-identified feature importance through collective multi-descriptor effects, and these relationships are consistent with established physical mechanisms from thermodynamic and thermal theories, demonstrating ML framework captures genuine composition-microstructure-property relationships.

**Supplementary Note 5: Baseline Model Comparisons for Toughness Prediction**

To benchmark ML performance against established physical models, we implemented three baseline approaches: Hall-Petch grain size scaling, Griffith elastic fracture mechanics, and constrained mixture modeling.

**(I) Hall–Petch-type:**

For Hall–Petch-type scaling, we applied the relationship:

$$K_{IC} = K_0 + k \cdot d^{-\frac{1}{2}} \quad \text{(S9)}$$

where d represents average grain diameter, $K_0$ captures intrinsic matrix toughness independent of grain size effects, and k quantifies grain boundary resistance to crack propagation. We applied literature parameters established for niobium-based alloys with $K_0$ = 10 MPa·$m^{1/2}$ and k = 60 MPa·$m^{1/2}$·$mm^{0.5}$.

Fig. S24 presents EBSD grain size maps for the four validation alloys revealing grain sizes of 58 μm for Alloy #1 (Fig. S24a), 48 μm for Alloy #2 (Fig. S24b), 72 μm for Alloy #3 (Fig. S24c), and 65 μm for Alloy #4 (Fig. S24d). Table S13 presents complete calculations with measured grain sizes and corresponding predictions.

The Hall–Petch-type scaling yields predictions of 17.07-18.66 MPa·$m^{1/2}$ with mean absolute percentage error of 12.7%. The model captures the general magnitude but shows systematic deviations, particularly for Alloy #2 with smaller grain size yielding 24.9% error. This indicates that while grain size contributes to toughness, it is not the dominant factor in these compositionally complex multi-phase alloys where phase distribution, interface characteristics, and transformation mechanisms play significant roles.

**(II) Griffith-type:**

For Griffith-type scaling, we applied the elastic fracture mechanics relationship:

$$K_{IC} = \sqrt{\frac{2E \cdot \gamma_{eff}}{1-\nu^2}} \quad \text{(S10)}$$

where E represents Young's modulus, $\gamma_{eff}$ captures effective fracture energy, and ν denotes Poisson's ratio.

Fig. S25 displays EBSD phase distribution maps with ductile Nbss solid solution phase and brittle $Nb_5Si_3$ silicide phase. Nbss ranges from 82.4 to 84.3% with average

83.3% while $Nb_5Si_3$ ranges from 14.6 to 17.3% with average 15.7%.

Fig. S26 presents nanoindentation measurements quantifying phase-specific elastic properties essential for Griffith fracture mechanics. We performed nanoindentation tests using the G200 nanoindenter with indentation depth of 500 nm and spacing of 20 μm between adjacent indents. The Nbss ductile phase exhibited moduli ranging from 135 to 141 GPa across the four alloys while the $Nb_5Si_3$ brittle silicide phase showed consistently higher values from 166 to 193 GPa. Weighted averaging by measured phase volume fractions yields effective elastic moduli ranging from 141.0 to 149.8 GPa for the four alloys detailed in Table S14. Table S15 shows complete Griffith model calculations and predictions.

The Griffith-type scaling yields predictions of 9.09-9.37 MPa·$m^{1/2}$ with mean absolute percentage error of 43.2%, representing systematic underestimation by approximately two-fold. This severe underestimation is expected because Griffith theory assumes ideal brittle fracture with crack propagation governed solely by elastic energy release and surface creation. In our Nb-Si composites, the ductile Nbss matrix (~83 vol.%) undergoes substantial plastic deformation ahead of crack tips, and additional energy dissipation occurs through crack deflection at strained phase boundaries and crack bridging by Nbss ligaments. This quantifies the extensive multi-mechanism toughening that Griffith theory fundamentally cannot capture.

**(III) constrained mixture model:**

The constrained mixture model addresses fundamental limitations of simple phase averaging by incorporating how the interconnected brittle silicide network geometrically constrains the deformation capacity of the ductile Nbss matrix. The governing equations are:

$$\mathrm{Pc(Vb)} = \exp\left\{-\frac{8q'}{3}\left[\frac{\mathrm{Vb}}{1-\mathrm{Vb}}\right]\right\} \quad \text{(S11)}$$

$$\mathrm{K_{IC}} = \mathrm{Kb}\left[1 + \alpha\sqrt{1-\mathrm{Vb}}\left[\left[\frac{\mathrm{Kd}}{\mathrm{Kb}}\right]^2 \mathrm{Pc(Vb)} - 1\right]\right]^{1/2} \quad \text{(S12)}$$

where Vb represents the brittle silicide phase volume fraction, Pc quantifies the constraint factor imposed by the brittle network on ductile phase deformation, q' is an

empirical parameter controlling constraint intensity (set to 1.0), $\alpha$ is a geometric correction factor (set to 0.5), Kb denotes the intrinsic toughness of the brittle phase (3.0 $MPa \cdot m^{1/2}$), and Kd represents the intrinsic toughness of the ductile phase (32.0 $MPa \cdot m^{1/2}$).

The constrained mixture model yields predictions of 16.36-17.07 $MPa \cdot m^{1/2}$ with mean error of 8.1% (Table S16), representing substantial improvement over Hall–Petch-type (12.7%) and Griffith-type scaling (43.2%). The model demonstrates consistent performance across all four alloys with narrow error range (5.3-9.8%), indicating successful capture of geometric constraint effects. The remaining deviations suggest that composition-driven variations in interface characteristics and transformation mechanisms introduce complexity beyond simple geometric constraints.

Fig. S27 compares prediction accuracy across all four approaches. Traditional models achieve mean errors of 12.7% (Hall–Petch-type), 43.2% (Griffith-type), and 8.1% (constrained mixture). The machine learning model achieves mean error of 3.1%, outperforming all three traditional models by capturing nonlinear multi-mechanism interactions rather than single dominant factors.

## Supplementary Note 6: Model Extrapolation

To quantify how far optimized alloys lie from the training distribution in compositional space, we implemented three complementary distance metrics: Mahalanobis distance, nearest-neighbor distance, and local density analysis.

### (I) Distance Metrics

Mahalanobis distance accounts for both variance and correlations among compositional features. Nearest-neighbor distance identifies compositional proximity to existing training data. Local density quantifies how well-populated the compositional space is around each prediction.

For the $K_Q$ dataset, Fig. S32a shows the Mahalanobis distance distribution, with risk zones defined as low ($D_M$ < 2.26, median), medium (2.26-6.27, 95th percentile), and high (> 6.27, beyond 95th percentile). Our four optimized alloys show distinct positions: alloys #2 and #3 fall in the low-risk zone with $D_M$ = 1.89 and 2.01 respectively, while alloys #1 and #4 occupy the medium-risk zone with $D_M$ = 3.45 and 4.12. Fig. S32b displays nearest-neighbor distances, revealing that alloy #3 has exceptional compositional proximity to training data with nearest-neighbor distance of only 2.1 (differing by ~2 at.% in major elements), while alloys #1, #2, and #4 show distances of 3.8, 3.2, and 4.5 respectively. Fig. S32c presents local density analysis using a search radius of 2.5$\sigma$ (where $\sigma$ is the average standard deviation across compositional dimensions), showing alloys #2 and #3 surrounded by 95 and 90 training neighbors respectively, while alloys #1 and #4 have 36 and 42 neighbors. Fig. S32d visualizes the combined metrics through a scatter plot of Mahalanobis distance versus local density, clearly showing alloys #2 and #3 embedded within the main training cluster (low $D_M$, high density) and alloys #1 and #4 in moderately explored regions.

The HTC dataset with 33 samples across 11 dimensions shows different extrapolation behavior. Fig. S33a shows training sample Mahalanobis distances range from 1.18 to 5.46 with median 2.48 and 95th percentile 4.58. Our optimized alloys display $D_M$ of 11.53 (#1), 19.23 (#2), 14.86 (#3), and 37.23 (#4) exceeding the

training maximum by factors of 2.1 to 6.8. All predictions fall into the high-risk extrapolation zone. Fig. S33b shows nearest-neighbor distances of 4.96 to 14.11 placing alloys at or above the 88th percentile of training distribution. Fig. S33c shows all four alloys display zero local density confirming complete isolation from training samples. Fig. S33d shows all alloys clustered in the unsupported region ($D_M > 11$, density = 0), contrasting sharply with the training distribution.

**(II) Model extrapolation analysis**

For HTC the sample-to-dimension ratio of 3.1 falls below typical requirements for capturing high-dimensional relationships. Standard confidence intervals fail to account for compositional distance. A prediction with zero local density carries higher uncertainty than one surrounded by numerous training examples. Extrapolative compositions may encounter unforeseen failure modes including phase instabilities or brittle fracture mechanisms in unexplored regions.

Experimental validation demonstrated successful predictions despite these limitations. For $K_Q$ predictions in well-supported regions with Mahalanobis distance below 3 and local density above 36 as shown in Fig. S32d, experimental measurements matched predictions within 5% error. Alloys #2 and #3 with $D_M$ of 1.61 and 1.75 and local densities of 95 and 90 neighbors showed excellent agreement. Even alloys #1 and #4 in moderately sparse regions with $D_M$ of 2.93 and 3.01 showed reasonable agreement. For HTC, experimental validation proved critical given the severe extrapolation visible in Fig. S33. All four predictions despite Mahalanobis distances of 11.53, 19.23, 14.86, and 37.23 (exceeding training maximum by factors of 2.1 to 6.8) and zero local density yielded experimental results within physically reasonable ranges. While prediction errors were larger than for $K_Q$ as expected for extrapolation, the model avoided catastrophic failures.

**Supplementary Note 7: Statistical Robustness of HTC Model**

The HTC model with only 34 samples requires comprehensive statistical validation to establish reliability. We implemented four complementary validation approaches: bootstrap ensemble analysis, Monte Carlo dropout, learning curve evaluation, and residual diagnostics.

**(I) bootstrapped ensembles**

For bootstrapped ensemble analysis, we trained (N=500) independent models on resampled training sets and aggregated predictions to obtain uncertainty estimates. Fig. S35a shows training set predictions with RMSE of 20.96 MPa and MAE of 13.56 MPa, where 95% confidence intervals shown as error bars remain relatively tight across samples. Fig. S35b presents test set results with RMSE of 11.29 MPa and MAE of 10.04 MPa, where error bars demonstrate that uncertainty estimates appropriately capture prediction variability. Fig. S35c shows training set prediction uncertainty distribution with mean of 5.29 MPa, standard deviation of 1.39 MPa, minimum of 2.95 MPa, and maximum of 7.81 MPa. Fig. S35d presents test set uncertainty distribution with mean of 5.16 MPa, standard deviation of 1.41 MPa, minimum of 4.03 MPa, and maximum of 8.44 MPa. The similar mean values between training and test sets suggest reasonable generalization. Fig. S35e compares predicted HTC values with actual measurements across all seven test samples sorted by actual value, where predicted values with 95% confidence intervals closely track actual values, with all actual measurements falling within or very close to predicted confidence intervals.

**(II) Monte Carlo dropout**

We also applied Monte Carlo dropout (N=1000) for independent epistemic uncertainty assessment. Fig. S36a shows training set predictions achieving RMSE of 3.21 MPa and MAE of 2.28 MPa. Fig. S36b presents test set results with RMSE of 2.11 MPa and MAE of 1.87 MPa. Fig. S36c shows training set uncertainties with mean of 13.13 MPa, standard deviation of 3.70 MPa, minimum of 10.35 MPa, and maximum of 30.38 MPa. Fig. S36d presents test set uncertainties with mean of 15.75 MPa, standard deviation of 5.24 MPa, minimum of 12.14 MPa, and maximum of

27.95 MPa. These values are systematically higher than bootstrap uncertainties because MC dropout captures additional model-structural uncertainty. Fig. S36e shows MC dropout confidence intervals successfully capture all actual measurements. Two independent uncertainty quantification methods both produce well-calibrated confidence intervals that successfully bracket all experimental test measurements. Bootstrapped ensembles yield mean uncertainty approximately 5 MPa while Monte Carlo dropout yields 13-16 MPa, providing quantified prediction uncertainty that addresses the statistical fragility concern.

**(III)Learning Curve Analysis**

we generated learning curves systematically varying training set size from 6 to 27 samples at eight levels (6, 10, 12, 15, 18, 20, 24, 27 samples) while maintaining consistent test set for fair comparison across different data fractions.

Fig. S37a shows training RMSE decreases from 90.2 MPa at 6 samples to 69.9 MPa at 27 samples while validation RMSE drops from 212.9 MPa to 132 MPa. The steep improvement from 6 to 15 samples with validation RMSE dropping over 40% indicates each additional sample provides substantial information when data is severely limited. Fig. S37b shows RMSE gap narrows from 122.7 MPa at 6 samples to 62.1 MPa at 27 samples with fitted slope K=-2.67 demonstrating consistent reduction in generalization gap.

Fig. S37c shows training MAE decreases modestly from 72.8 MPa to 70.5 MPa while validation MAE improves substantially from 169.4 MPa to 70.5 MPa. Fig. S36d shows MAE gap reduces from 96.6 MPa to 13.9 MPa with steeper slope K=-3.69 than RMSE gap indicating typical errors converge faster than worst-case errors. Fig. S36e shows loss functions with training loss decreasing from 5336 to 2962 and validation loss dropping from 28639 to 10506. Fig. S36f shows loss gap narrowing from 23303 to 7544 with slope K=-664.

Fig. S37g shows prediction uncertainty decreases dramatically from 83.5 MPa at 6 samples to 8.2 MPa at 27 samples representing 90% reduction. Fig. S37h shows confidence interval width narrows from 163.7 MPa to 15.9 MPa across training sizes.

The consistent narrowing of training-validation gaps across all metrics confirms the optimized 865-parameter architecture achieves appropriate capacity for available data without severe overfitting.

**(IV)Residual Diagnostics**

we performed comprehensive residual analysis evaluating heteroscedasticity, normality, train-test consistency, and error independence.

Fig. S12a shows residuals against predicted HTC values for both training and test sets. All data points fall within the $\pm 2\sigma$ envelope with no monotonic trend or curvature across the predicted value range of approximately 200-450 MPa, ruling out heteroscedasticity and confirming that prediction error does not systematically vary with the magnitude of predicted property. Fig. S12b shows overall residual histogram following near-Gaussian shape with mean of 1.587 MPa corresponding to less than 0.7% of the full prediction range. Fig. S12c shows Q-Q plots where both training and test residuals align closely with theoretical normal reference line, supported by Shapiro-Wilk tests yielding $p=0.1573$ for training and $p=0.127$ for test, both exceeding 0.05 threshold so normality cannot be rejected. Fig. S12d shows separated histograms where training and test mean residuals are 1.518 MPa and 1.854 MPa respectively with difference of only 0.336 MPa, confirming the model does not introduce systematic bias when generalizing from training to test data.

Fig. S12e shows autocorrelation function with ACF values at all lags beyond zero remaining within 95% confidence interval confirming no serial correlation among prediction errors. Fig. S12f shows residual trend plot with 3-point moving average remaining close to zero throughout with fluctuations contained within $\pm 1\sigma$ bands. These analyses confirm prediction errors carry no structured information linked to sample ordering. The residuals satisfy standard assumptions for reliable regression confirming absence of systematic bias.

These four validation approaches collectively establish that despite the limited sample size, the HTC model achieves statistically robust predictions with

well-calibrated uncertainty estimates. The simplified architecture and validation justify deployment for compositional screening.

## Supplementary References


1. Subramanian, PR, Mendiratta, MG & Dimiduk, DM. The development of Nb-based advanced intermetallic alloys for structural applications. *JOM* **48**, 33-38 (1996).
2. Bewlay, BP, Jackson, MR & Subramanian, PR. Processing high-temperature refractory-metal silicide in-situ composites. *JOM* **51**, 32-36 (1999).
3. Tyson, WR & Miller, WA. Surface free energies of solid metals: Estimation from liquid surface tension measurements. *Surf. Sci.* **62**, 267-276 (1977).
4. Liebermann, HH. *Rapidly solidified alloys : processes, structures, properties, applications*. New York : M. Dekker (1993).
