# Supplementary material for: High temperature Nb-Si alloys using data science: optimization of fracture toughness and high-temperature strength
Source: Nat Commun. 2026 Jul 10;17:8503. doi: 10.1038/s41467-026-75353-6 (PMC13483041; doi:10.1038/s41467-026-75353-6)
Supplement: Supplementary file 2 [file 41467_2026_75353_MOESM2_ESM.pdf]

nature portfolio

# Peer Review File

# High temperature Nb-Si alloys using data science: optimization of fracture toughness and high-temperature strength

Corresponding Author: Dr Dezhi Chen

Parts of this Peer Review File have been redacted as indicated to remove third-party material.

**This file contains all reviewer reports in order by version, followed by all author rebuttals in order by version.**

Version 0:

Reviewer comments:

Reviewer #1

(Remarks to the Author)
Recommendation: The manuscript can be accepted after major revision.

In this work, the authors present a data-driven framework—combining feature-screening, interpretable machine learning, and multi-objective optimization—with experimental validation to design ultra-high-temperature Nb–Si alloys. The authors first reduce an initial pool of compositional and processing descriptors to six features. Using these, they train two model on exclusively experimental data: one for room-temperature fracture toughness (203 points) and one for 1250 °C compressive strength (34 points). SHAP analysis reveals that melting enthalpy and atomic-radius mismatch dominate toughness, while the volume-fraction ratio of solid-solution governs high-temperature strength. An NSGA-II algorithm then identifies Pareto-optimal compositions, outperforming prior as-cast Nb–Si alloys. Experimental synthesis and mechanical testing of four selected alloys validate the model predictions within 5% error, and microstructural characterization links the chosen descriptors to observed phase fractions and grain structures. Overall, the manuscript delivers critical insight into how interpretable ML can accelerate the discovery of next-generation ultra-high-temperature composites. However, the following questions must be answered prior to acceptance, and the authors should consider the comments below.

• Both KQ and HTC are inherently temperature- and processing-dependent, yet neither temperature nor any heat-treatment variables appear in the final feature set. As written, the models merely “bake in” a single test temperature and assume identical processing for all alloys—an unjustified simplification.
• The current approach implies that alloys produced by different manufacturing techniques would exhibit identical properties, which is not physically realistic. The authors must clarify the model’s domain of validity or incorporate processing parameters explicitly.
• How do the ML predictions compare quantitatively with established physical or empirical models? For example, rule-of-mixtures for solid-solution strengthening versus ML-predicted HTC, or Griffith/Hall–Petch models for toughness. Including one or two such baselines is essential to demonstrate true ML “accuracy gain.”
• The stability of the Pareto frontier should be assessed. The authors must perform repeated NSGA-II optimizations with perturbed SHAP weights or bootstrapped datasets to show that the identified front is not an artifact of sampling noise.
• Training on just 203 fracture-toughness points and only 34 high-temperature strength points is extremely limited for an 8-dimensional compositional space. The authors need to justify the complexity of their ANN relative to data volume, and demonstrate robust cross-validation, uncertainty quantification, or comparison against simpler regressors.
• Without strong cross-validation, confidence intervals, or simpler baseline models, it is unclear whether the HTC model is capturing real physio-chemical trends or merely overfitting noise.
• While the authors offer post-hoc explanations (e.g. atomic-radius mismatch → γ′ dispersion), these were not part of the initial feature assessment. They should strengthen mechanistic linkage by regressing selected descriptors directly against measured phase fractions or grain-size metrics.
• Figure captions must explicitly describe all colors, marker shapes, and data groupings so each figure is fully self-contained.
• Multipanel figures (especially Figs. 5 and 6) are overly complicated. The authors should split them into simpler, focused plots. What are different fits away from central lines in Figures 3a and b.

• Figures 3e–f add little insight and could be replaced with straightforward bar charts of feature importances or model errors.
• Current visualizations are more complex than necessary. Simplify plots to convey key results clearly—for instance, replace dense scatter panels with bar or line charts where appropriate.
• Provide full details of the ANN architectures (layer counts, neuron numbers, activation functions, regularization strategies), training protocols (learning rates, early stopping, dropout), and cross-validation schemes. Overall, the methodology section requires significant expansion to allow reproducibility.

(Remarks on code availability)

Reviewer #2

(Remarks to the Author)
Overall
The article focuses on Nb-Si alloys, aiming at the optimization of fracture toughness and high-temperature strength. By integrating a three-step feature selection process, SHAP analysis, and NSGA-II multi-objective optimization, a Nb-12.26Si-21.35Ti-1.98Al-1.96Cr-0.51Hf-4.34Zr-4.35V alloy was designed. Microstructural observations and analyses were conducted to elucidate the primary reasons behind the alloy's superior performance. In my view, however, the design methodology adopted here is fairly conventional. The work essentially represents an application of well-established approaches to a specific material system, and the reported performance and mechanisms are not particularly striking. Consequently, the manuscript does not demonstrate sufficient novelty. Following are some specific suggestions:

Major
1. The authors claim to have employed “a three-step progressive feature screening strategy” for feature selection. However, such an approach is already well established and widely adopted. This does not demonstrate any incremental methodological contribution in terms of machine learning.
2. A key issue lies in the complete disconnection between the machine learning-guided alloy design and the subsequent mechanistic understanding. The manuscript does not present any discussion linking the “key factors identified by the ML model with the proposed strengthening mechanisms”. Establishing such a connection is crucial; otherwise, it remains unclear whether the reported alloy design is an inherent outcome of the model or simply a matter of coincidence.
3. The Introduction is poorly written. For instance, the statement “it is difficult to meet the requirements of processing and assembly, and there is an inherent competition between room-temperature fracture toughness and high-temperature strength” is merely descriptive and provides little scientific insight to the reader. The authors should articulate the fundamental scientific origins of this trade-off. Moreover, following the overview of machine learning applications, the necessity of using ML to specifically address the toughness–strength trade-off remains unconvincing. A more targeted review of progress in multi-objective optimization with ML, and an explanation of why ML can effectively assist in resolving this issue, would be far more relevant to the manuscript’s central theme, rather than a generic summary such as “machine learning-guided development of high entropy alloys with optimized hardness and strength-ductility balance.”
4. The introduction identifies four issues in applying machine learning to Nb-Si-based alloys: (1) lack of systematic feature engineering methods; (2) insufficient model interpretability; (3) absence of multi-objective optimization strategies; and (4) limited integration of mechanistic understanding with experimental validation. These issues appear to be derived from the methods used or the work conducted in this paper rather than from a thorough analysis of the field's literature. The fields of feature engineering, interpretability, multi-objective optimization, and multi-scale microstructural analysis have all developed over many years. For instance, the feature selection method used in the paper has been applied to Ni-Ti-based alloys, copper alloys, and other alloy designs since 2016 [Nature Communications, 2016, 7: 11241; Acta Materialia, 2020, 200: 803-810]. Similarly, SHAP methods and multi-objective optimization have been widely used [Computational Materials Science, 2022, 211: 111560; Materials Today Communications, 2021, 28: 102709], and multi-scale microstructural analysis is a standard approach in alloy microstructure analysis, from micron-scale grains to nanoscale precipitates.
5. There are methodological flaws in the feature selection process. Specifically, the authors write: “Finally, the RFE (recursive feature elimination) method was adopted, using ElasticNet regression as the base model to select the optimal subset from 20 features (Fig. 2e, f).” Since ElasticNet is a linear model, the RFE process inherently favors features with direct linear correlations, while potentially discarding those that have nonlinear effects. This poses a serious concern, as the subsequent predictive models (RF, GBDT, ANN) are nonlinear. Consequently, the features fed into these models may already be deficient in information. Although the ANN shows sufficient generalization capability, this technical issue could very well explain the relatively poor performance of RF and GBDT, rather than a superficial attribution to “overfitting.”
6. When studying fracture toughness and high-temperature strength, the paper mentions constructing two datasets with 203 and 34 data points, respectively. However, Figure 3c shows a number of data points far exceeding 203, and Figures 3b and 3d show 36 and 35 data points, respectively, which is inconsistent.
7. The paper focuses on the effectiveness of feature selection, SHAP analysis, and NSGA-II results but lacks an analysis of why these methods are effective in Nb-Si alloy design, especially since the design of Nb-Si alloys involves a small dataset with the two performance prediction datasets containing 203 and 34 data points, respectively.
8. The manuscript should clarify why alloys #2 and #4 deviate so far from the training set distribution in the t-SNE plot. Were additional elements introduced? Or did certain element concentrations exceed the range present in the current dataset? Please provide either the exact compositions of the designed alloys or reasonable compositional ranges.
9. The statement “This pre-stress state can alleviate stress concentration effects at crack tips and suppress further crack propagation” is not acceptable. Given the large stress fields and the orientation mismatch across incoherent interfaces, such

features are more likely to serve as crack initiation sites and accelerate crack growth. The authors provide no prior evidence, nor experimental or computational support, for this claim.

________________________________________

Minor

1. Please ensure consistency in the use of Fig. vs. Figure throughout the manuscript.
2. In Fig. 3a–b, legends distinguishing training and test sets are missing, and what do the three parts of the points represent? In Fig. 3c–d, the abbreviation MRE should be defined. While Fig. 3e–f appears visually appealing, there are no numerical references provided, making it impossible to estimate the prediction accuracy from the figures alone.
3. There is a lack of necessary description of the data partitioning method in the approach. A crucial point is whether the test set used in your data partitioning process can reflect the generalization ability of the model for the properties of unknown alloys.
4. The Supplementary Information does not include any details about the model parameter settings. In the absence of shared code, it is essential to provide at least sufficient details of the parameterization to enable meaningful assessment of the models.
5. Besides composition, processing methods are also crucial in influencing alloy performance. How did the paper handle the impact of different processing methods on performance when collecting data? The preparation method used in this paper is arc melting. Were the methods used in the sample data also the same?
6. The paper compares RF, BG, GBDT, DT, XGB, CatBoost, and ANN algorithms. Has the SVR algorithm been tried? For modeling small sample data, the SVR algorithm has significant advantages and has been validated and applied in many instances.
7. During correlation screening, the paper calculates both PCC and MIC correlation coefficients between each feature and performance, retaining features with correlation coefficients above a threshold. However, it is known that PCC only calculates linear correlations, while MIC only calculates simple nonlinear correlations. This screening assumes that the relationship between features and performance is linear or simply nonlinear. In reality, the mechanisms influencing material performance are diverse and deeply complex, and these relationships are likely to be complexly nonlinear. This screening method lacks a reasonable explanation.

(Remarks on code availability)
The original manuscripta does not provide the dataset and original code, so the related work cannot be reproduced.

Version 1:

Reviewer comments:

Reviewer #1

(Remarks to the Author)
I appreciate the effort the authors have put into the revisions and responses. The manuscript has improved in organization, clarity, and methodological description. The integration of ML with experimental validation remains interesting and potentially impactful for Nb–Si alloy development. However, several key concerns from the previous round have not been fully resolved. These issues are critical for establishing rigor, reproducibility, and mechanistic credibility at the level required for Nature Communications. I outline them below.

Comment 1: Statistical robustness of the HTC model: Even with the added 5-fold cross-validation, the high-temperature strength model remains statistically fragile. With only 34 data points, each fold contains roughly 6–7 samples, which is not sufficient to rigorously evaluate generalization. The ANN architecture (450 neurons) is also disproportionately large relative to dataset size, and the manuscript still does not include uncertainty quantification.

What is needed:

1) Prediction-uncertainty estimates (e.g., bootstrapped ensembles, Monte Carlo dropout, Bayesian regression comparison).

2) Learning curves showing error vs. training-set size.

3) Residual analyses to confirm the absence of systematic bias.

4) A justification for ANN capacity relative to available data, or a simplified architecture.

5) At present, the HTC model remains the weakest scientific component of the work.

Comment 2: Mechanistic linkage remains largely post-hoc: Although more discussion has been added, the mechanistic connections are still descriptive rather than predictive. The reviewer request to regress ML-selected descriptors directly against microstructural metrics was not fulfilled. The manuscript still relies heavily on narrative explanation after the fact.

Needed in revision:

1) Quantitative relationships between descriptors and measured microstructural parameters for the experimentally investigated alloys (phase fractions, grain sizes, γ′ particle size, interface strain fields, etc.).

2) Clear distinction between what is supported by data vs. interpretive hypothesis.

3) Improved discussion of how these mechanistic insights could be integrated into, or at least checked against, the ML framework.

4) Mechanistic grounding is essential to justify why certain features truly matter.

Comment 3: Missing toughness baseline comparisons: The previous review explicitly requested comparison with simple physical or empirical toughness models (e.g., Griffith-type, Hall–Petch-type scaling). Only strength was compared to a Labusch–Nabarro model.

This omission needs to be corrected.

Expected revision:

1) At least one toughness baseline (Griffith, Hall–Petch, or related scaling) applied to the dataset or the validated alloys.

2) Discussion of where and why the ML model provides value beyond these traditional models.

3) This is important to demonstrate genuine accuracy or insight, beyond curve-fitting.

Comment 4: Processing homogeneity is overstated: Restricting the dataset to arc-melted samples does not eliminate processing variability. Arc-melting parameters vary widely across studies (melt temperature, number of remelts, oxygen contamination, mold geometry, cooling conditions). These factors strongly influence toughness and microsegregation.

The manuscript currently asserts uniformity without evidence.

Needed revision:

1) Quantitative ranges or summary statistics of processing conditions across the dataset (where available).

2) Discussion of the expected magnitude of property variation caused by these differences.

3) A clear limitation statement about the model's sensitivity to uncontrolled processing variations.

Comment 5: Feature-selection pipeline may bias the final model

Although the authors explain their PCC–MIC–RFE process in more detail, concerns remain about the interaction of linear filtering methods with nonlinear models.

Needed:

1) A secondary feature-validation method (e.g., permutation importance, SHAP-based pruning) to show that the final set of six features is stable and not an artifact of the linear RFE step.

2) Performance comparison with and without RFE.

3) This is necessary to avoid inadvertently excluding relevant nonlinear interactions.

Comment 6: Model extrapolation claims need stronger support: Some optimized alloys fall outside the training distribution in the t-SNE space. While experimental validation is encouraging, the manuscript should provide a more quantitative measure of how far these points lie from the data manifold.

Requested:

1) Mahalanobis distance, density-based distance, or similar metric to assess extrapolation.

2) Clear discussion of the risks and limits of extrapolating beyond the training space.

Minor Points:

Some figures remain overly dense. Consider a consolidated workflow figure for clarity.

Check consistency of numerical values, units, and thresholds introduced in the supplementary materials.

Ensure that all updated figures include clear legends, axis labels, and color descriptions.

(Remarks on code availability)
Am I supposed to install and run this code?

Reviewer #2

(Remarks to the Author)
The quality of the revised manuscript has improved. However, the following issues remain.
1. The previous review noted that the two datasets are reported as having 203 and 34 samples, which are inconsistent with the counts shown in Figure 3c and those in Figures 3b and 3d. The response addressed only the case with 34 samples, but not the case with 203 samples. Please also clarify the inconsistency for the dataset with 203 samples.
2. The response repeatedly emphasizes the importance of combining feature selection, SHAP analysis, and NSGA-II. What would be the impact if any one of these components were omitted? Further analysis and discussion are recommended.
3. The manuscript computes PCC and MIC between each feature and performance for preliminary feature screening and uses relatively low thresholds (PCC > 0.2, MIC > 0.2 in the first model; PCC > 0.09, MIC > 0.08 in the second model). What is the rationale for choosing these thresholds? Why is there such a large discrepancy between the two models?

(Remarks on code availability)

Version 2:

Reviewer comments:

Reviewer #2

(Remarks to the Author)
The revised manuscript has substantially improved in quality. However, several minor issues remain that could be further refined. The following specific suggestions are offered:
1. While the manuscript systematically demonstrates the use of feature selection, SHAP analysis, and NSGA-II, it lacks a scientific rationale for the selection of these methods. It is recommended to elaborate on the methodological justification, including their suitability for small datasets and related considerations.
2. The manuscript states that the designed alloys outperform all reported cast Nb–Si alloys, yet the main text does not provide an intuitive comparative performance plot. Consider moving the comparative figure from the Supplementary Information to the Results section and presenting fracture toughness and high-temperature strength in a dual-axis plot.
3. The HTC model is trained on only 34 samples, and its representativeness warrants further discussion. The manuscript does not examine whether the compositional distribution of these samples adequately covers the design space, nor does it assess potential clustering biases among samples. Insufficient representativeness may lead to an overestimation of the model's generalization capability.
4. In the NSGA-II multi-objective optimization, KQ and HTC are weighted 1:1, but the basis for this choice is not explained. It is recommended to include a sensitivity analysis to evaluate how alternative weightings influence the optimization outcomes.

(Remarks on code availability)

**Open Access** This Peer Review File is licensed under a Creative Commons Attribution 4.0 International License, which permits use, sharing, adaptation, distribution and reproduction in any medium or format, as long as you give appropriate credit to the original author(s) and the source, provide a link to the Creative Commons license, and indicate if changes were made.
In cases where reviewers are anonymous, credit should be given to 'Anonymous Referee' and the source.
The images or other third party material in this Peer Review File are included in the article's Creative Commons license, unless indicated otherwise in a credit line to the material. If material is not included in the article's Creative Commons license and your intended use is not permitted by statutory regulation or exceeds the permitted use, you will need to obtain permission directly from the copyright holder.
To view a copy of this license, visit https://creativecommons.org/licenses/by/4.0/

## Responses to the comments by the editor and the reviewers

Dear editor and reviewer,

We thank you and the reviewers to review and comment our manuscript. We have carefully read the comments and thought them over, and have revised the manuscript according to the View Letter. The responses are listed as follows. Besides, the corresponded revisions are highlighted in red color in this revised manuscript.

**Reviewer #1**

Recommendation: The manuscript can be accepted after major revision.

In this work, the authors present a data-driven framework—combining feature-screening, interpretable machine learning, and multi-objective optimization—with experimental validation to design ultra-high-temperature Nb–Si alloys. The authors first reduce an initial pool of compositional and processing descriptors to six features. Using these, they train two model on exclusively experimental data: one for room-temperature fracture toughness (203 points) and one for 1250 °C compressive strength (34 points). SHAP analysis reveals that melting enthalpy and atomic-radius mismatch dominate toughness, while the volume-fraction ratio of solid-solution governs high-temperature strength. An NSGA-II algorithm then identifies Pareto-optimal compositions, outperforming prior as-cast Nb–Si alloys. Experimental synthesis and mechanical testing of four selected alloys validate the model predictions within 5% error, and microstructural characterization links the chosen descriptors to observed phase fractions and grain structures. Overall, the manuscript delivers critical insight into how interpretable ML can accelerate the discovery of next-generation ultra-high-temperature composites. However, the following questions must be answered prior to acceptance, and the authors should consider the comments below.

***Response:***

*We sincerely thank the reviewer for the comprehensive and constructive evaluation recognizing that our work "delivers critical insight into how interpretable ML can accelerate the discovery of next-generation ultra-high-temperature composites" with experimental validation "within 5% error."*

*We greatly appreciate the detailed comments that have improved our manuscript's quality and clarity. In response, we have made substantial revisions addressing all major concerns: (1) clarified that our models apply specifically to arc-melted, as-cast*

*Nb-Si alloys with systematic database construction ensuring data consistency; (2) added comprehensive 5-fold cross-validation demonstrating robust model performance; (3) compared ML predictions with the Labusch-Nabarro solid solution strengthening model, revealing ML's advantages for compositionally complex systems; (4) conducted Pareto frontier stability analysis through repeated optimizations, confirming reproducible results; (5) established explicit connections between each ML-identified feature and corresponding microstructural mechanisms observed in TEM/EBSD characterization, demonstrating that alloy design results from physical principles rather than coincidence.*

*The experimental validation—achieving record-breaking as-cast fracture toughness of 18.92 MPa·m$^{1/2}$ while maintaining 322 MPa high-temperature strength at 1250°C—confirms our integrated framework successfully addresses the longstanding toughness-strength trade-off in Nb-Si alloys. We believe the revised manuscript now provides a comprehensive and scientifically rigorous demonstration of how systematic integration of materials informatics techniques can accelerate ultra-high temperature alloy discovery.*

Major Comments:

[Q1] Both KQ and HTC are inherently temperature- and processing-dependent, yet neither temperature nor any heat-treatment variables appear in the final feature set. As written, the models merely "bake in" a single test temperature and assume identical processing for all alloys—an unjustified simplification.

***Response:***

*The reviewer correctly identifies that our models do not include temperature or heat-treatment variables as features. This reflects our systematic approach to database construction where we specifically focused on arc-melted, as-cast Nb-Si alloys to ensure data consistency and eliminate processing-induced variations.*

*During our comprehensive literature survey across major databases including Web of Science and Materials Project, we analyzed the distribution of processing methods in published Nb-Si alloy research. Our analysis revealed that approximately 70% of published data used arc melting to produce as-cast samples, while the remaining 30% employed various alternative methods including directional solidification, laser additive manufacturing, powder metallurgy, and post-casting heat treatments.*

*However, incorporating samples from different processing routes would introduce significant complications for machine learning modeling. Each processing method*

*requires specific parameter features (e.g., cooling rates for directional solidification, layer thickness and scanning speed for additive manufacturing, time-temperature profiles for heat treatments), creating sparse feature matrices where most parameters would be zero for most samples. Additionally, many studies employed unconventional alloying elements (La, In, Co, Ni, Sc etc.) that appear in only a few compositions, further exacerbating feature sparsity issues.*

*To avoid these complications and ensure robust statistical modeling, we systematically selected only arc-melted, as-cast samples with compositions containing commonly studied elements (Nb, Si, Ti, Al, Cr, Hf, Zr, V, Mo, W, Ta, Sn etc.). This filtering process resulted in our final database of 203 $K_Q$ measurements and 34 HTC measurements. By controlling for processing route (arc-melted, as-cast) and test conditions (room temperature for $K_Q$, 1250°C for HTC), our models can reliably predict properties within this well-defined domain.*

*The experimental validation confirms this approach: all four optimized compositions achieved prediction errors below 5%, including the best-performing alloy with record-breaking as-cast fracture toughness of 18.92 MPa·$m^{1/2}$. We have clarified the model's domain of validity in the revised manuscript, noting that predictions apply specifically to arc-melted, as-cast Nb-Si alloys under the stated test conditions.*

*These details have been added to the **Materials and Methods** section.*

[Q2] The current approach implies that alloys produced by different manufacturing techniques would exhibit identical properties, which is not physically realistic. The authors must clarify the model's domain of validity or incorporate processing parameters explicitly.

***Response:***

*We agree that different manufacturing methods affect alloy properties. Our systematic database construction specifically addressed this challenge by focusing exclusively on arc-melted, as-cast samples to ensure training data consistency and enable reliable property prediction within a well-defined processing domain.*

*As detailed in our response to Question 1, our comprehensive literature analysis revealed that approximately 70% of published Nb-Si alloy data used arc melting to produce as-cast samples. The remaining 30% employed diverse processing methods including directional solidification, laser additive manufacturing, powder metallurgy, and various heat treatment protocols. However, incorporating this diversity would create significant modeling challenges due to sparse feature matrices and the need for*

*method-specific processing parameters.*

*Our decision to focus on arc-melted, as-cast samples was driven by both data availability and modeling considerations. Arc melting with controlled remelting (typically 7 or more cycles) produces reproducible microstructures characterized by equiaxed dendritic solidification and relatively uniform composition with limited microsegregation. This processing homogeneity isolates the effect of chemical composition on properties, which is precisely what our machine learning models are designed to capture.*

*All four optimized alloys synthesized in this study used identical arc melting protocols: high-purity raw materials (>99.9%), argon atmosphere (>99.9%), 7 remelting cycles for homogeneity, copper hearth cooling (estimated $10^2$-$10^3$ K/s), and no post-casting treatment. This ensures experimental validation occurs within the same processing domain as training data, validating model predictions under consistent conditions.*

*We acknowledge in the revised manuscript that this approach limits model applicability to arc-melted, as-cast alloys. However, this focused scope enabled successful identification of superior alloy compositions, as demonstrated by achieving properties that surpass all previously reported as-cast Nb-Si alloys in the literature. Future work expanding to other processing routes would require systematic datasets comparing identical compositions across different methods, which are not currently available in sufficient quantity for reliable machine learning modeling.*

*These details have been added to the* ***Materials and Methods*** *section.*

[Q3] How do the ML predictions compare quantitatively with established physical or empirical models? For example, rule-of-mixtures for solid-solution strengthening versus ML-predicted HTC, or Griffith/Hall–Petch models for toughness. Including one or two such baselines is essential to demonstrate true ML “accuracy gain.”

***Response:***

*We thank the reviewer for this constructive suggestion. We have compared our ML predictions with the Labusch-Nabarro solid solution strengthening model as a baseline. We calculated theoretical yield strengths using the standard formula based on atomic size misfit and shear modulus difference. We applied a temperature correction factor of 0.25 to adjust room-temperature properties to 1250°C, based on published data for refractory high-entropy alloys[1]. The detailed calculation process has been added to the supplementary materials (Supplementary Table 6). The calculated values are: Alloy #1: 287.93 MPa, Alloy #2: 312.65 MPa, Alloy #3: 298.77 MPa, and Alloy #4: 292.77*

*MPa. Comparing these with ML predictions and experimental results shows that the physics-based Labusch-Nabarro model exhibits prediction errors within 15% across all compositions, while the ML model achieves significantly higher accuracy with errors below 5%.*

*This indicates that ML provides greater advantage for compositionally complex systems where multiple strengthening mechanisms interact. The physical model accounts only for solid solution strengthening, missing effects from precipitation, grain boundaries, and phase stability. For simpler systems where solid solution dominates, both approaches work similarly. The physical model still provides useful understanding of the solid solution mechanism, while ML integrates multiple mechanisms from experimental data.*

*This comparison has been added to the* ***Multi-objective optimization and Experimental validation*** *section, with calculation details in* ***Supplementary Table 6****.*

*References*

*[1] O.N. Senkov, D.B. Miracle, K.J. Chaput, J.-P. Couzinie, Development and exploration of refractory high entropy alloys—A review, J. Mater. Res. 33(19) (2018) 3092-3128.*

[Q4] The stability of the Pareto frontier should be assessed. The authors must perform repeated NSGA-II optimizations with perturbed SHAP weights or bootstrapped datasets to show that the identified front is not an artifact of sampling noise.

***Response:***

*We appreciate the concern about Pareto frontier stability. Our original study used four different alloy systems (5-, 6-, 7-, and 8-element) with 10,000 compositions sampled for each using Latin hypercube sampling. The Pareto fronts show consistent patterns across systems, with the 8-element and 7-element systems providing broader ranges than simpler systems. Most importantly, experimental testing of four predicted compositions achieved errors under 5% with an average error of 2.4%, confirming the predictions represent real trends rather than artifacts.*

*Following the reviewer's suggestion, we conducted 10 additional independent optimizations for the 8-element system using different random seeds. We have compiled the results in tabular form (Supplementary Table 4) showing the frequency of Pareto-optimal solutions at representative property values. The analysis shows that approximately 85% of optimal compositions identified in any single run also appeared in at least 8 out of 10 runs. Property values within each composition cluster varied by less than 3% across runs, demonstrating reproducible optimization results.*

*Regarding SHAP weight perturbation, we note that SHAP values are deterministic outputs from the trained model representing actual feature contributions. Random perturbation would disconnect the optimization from the underlying physics. Instead, we assessed model uncertainty through our training approach using 100 different random train-test splits, which yielded consistent feature importance rankings. The t-SNE analysis shows some optimized alloys fall outside the training distribution yet maintain prediction accuracy, supporting that the Pareto fronts represent genuine physical trends rather than sampling noise.*

*We have added* ***Supplementary Table 4*** *to the supplementary materials.*

[Q5] Training on just 203 fracture-toughness points and only 34 high-temperature strength points is extremely limited for an 8-dimensional compositional space. The authors need to justify the complexity of their ANN relative to data volume, and demonstrate robust cross-validation, uncertainty quantification, or comparison against simpler regressors.

***Response:***

*We acknowledge the reviewer's concern about limited training data relative to the compositional space complexity. Our approach addresses this challenge through systematic feature reduction and model validation. We reduced the initial 200 features to 6 key descriptors using a three-step screening process (PCC correlation removal, PCC-MIC joint filtering, and RFE), which significantly reduces model complexity while retaining predictive power. The ANN architecture uses only 7 input features with L2 regularization (1e-5), batch normalization, and early stopping criteria to prevent overfitting. We compared seven different algorithms and found that simpler models like Random Forest ($R^2$=0.88) and Gradient Boosting ($R^2$=0.89) performed worse than the ANN ($R^2$>0.96), validating the need for the chosen architecture.*

*To further demonstrate model robustness and address concerns about overfitting, we have now implemented rigorous 5-fold cross-validation for both models. For the $K_Q$ model (203 samples), the cross-validation yields $R^2$ values of 0.94±0.01 (mean±std), RMSE of 0.97±0.07 MPa·$m^{1/2}$, and MAE of 0.72±0.04 MPa·$m^{1/2}$. For the HTC model (34 samples), we obtain $R^2$ of 0.89±0.01, RMSE of 4.4±0.3 MPa, and MAE of 3.4±0.2 MPa. The small standard deviations across folds demonstrate that our models achieve stable performance across different data partitions, indicating they capture genuine composition-property relationships rather than overfitting to specific training subsets. This k-fold validation complements our existing validation approach, which includes*

*dynamic train-test splitting with different random seeds across multiple iterations, rolling averages over 5-iteration windows, and strict convergence criteria (training $R^2>0.95$, testing $R^2>0.91$). The model demonstrates consistent performance across different data splits, with prediction errors below 5% for all optimized alloys.*

*While the HTC dataset is limited to 34 points, the strong correlation between predicted and experimental values ($R^2>0.91$), the consistency across cross-validation folds, and the success of experimental validation suggest the model captures the underlying physics-property relationships effectively within the studied compositional range. Most importantly, experimental validation of four predicted compositions achieved errors below 5% with an average of 2.4%, including the best-performing alloy with fracture toughness of 18.92 MPa·$m^{1/2}$ that surpasses all previously reported as-cast Nb-Si alloys. We have added **Supplementary Tables 14 and 15**, and supplemented the corresponding details in the **Materials and Methods** section.*

c

[Q6] Without strong cross-validation, confidence intervals, or simpler baseline models, it is unclear whether the HTC model is capturing real physio-chemical trends or merely overfitting noise.

***Response:***

*To address the reviewer's concern about validation rigor, we have implemented comprehensive 5-fold cross-validation to demonstrate that our HTC model captures real physicochemical trends rather than overfitting noise. The cross-validation yields consistent performance metrics across all five folds: $R^2$ of 0.89±0.01, RMSE of 4.4±0.3 MPa, and MAE of 3.4±0.2 MPa.*

*Our HTC model also employs robust validation through dynamic train-test splitting with different random seeds across multiple iterations, combined with rolling averages over 5-iteration windows and strict convergence criteria (training and testing $R^2>0.91$, RMSE<7). We compared eight algorithms including Random Forest, Gradient Boosting, SVR, Decision Tree, and linear methods, where the ANN consistently outperformed these approaches. The selected features have clear physical significance - atomic radius mismatch (am), speed of sound, thermal expansion coefficient, specific heat capacity, covalent radius, and electron affinity variance - all directly related to high-temperature mechanical behavior through thermal stability, atomic bonding, and electronic structure effects.*

*The model captures genuine physico-chemical trends rather than noise, as evidenced by the consistent performance across different random data splits, cross-validation*

*folds, and the physical interpretability of feature relationships. Our SHAP analysis reveals that features like specific heat capacity and thermal expansion coefficient show expected correlations with high-temperature strength, consistent with known metallurgical principles. The regularization strategies (L2 regularization, batch normalization, early stopping) and the successful experimental validation of predicted compositions further support that the model learns meaningful relationships rather than overfitting to noise. The prediction errors for all four experimentally validated compositions remain below 5%, including alloys with complex 8-element and 7-element compositions where multiple strengthening mechanisms interact.*

*We have added* ***Supplementary Tables 14 and 15*** *to the supporting information.*

[Q7] While the authors offer post-hoc explanations (e.g. atomic-radius mismatch → γ′ dispersion), these were not part of the initial feature assessment. They should strengthen mechanistic linkage by regressing selected descriptors directly against measured phase fractions or grain-size metrics.

***Response:***

*The reviewer makes a valid point about mechanistic linkage. Our feature selection was based primarily on statistical methods, but the selected features do have physical meaning. Atomic radius mismatch relates to lattice distortion and interfaces, melting enthalpy controls phase formation temperatures, and thermodynamic parameters affect phase stability. Our experimental work validates these connections for Alloy #1: the nanoscale γ'-$Nb_5Si_3$ particles we observed (average size 94.88 nm), the 67.6% lattice mismatch at interfaces shown in Figure 6d, and the 6-8% strain gradients in our GPA analysis all correlate with the atomic radius mismatch descriptor.*

*However, we agree our approach establishes these connections after the fact through experimental validation of the best-performing composition rather than building them into the model initially or systematically across all compositions. A more integrated approach would explicitly model the relationships from composition to descriptor, then to microstructure, and finally to property. This would require collecting quantitative microstructure data like phase fractions and grain sizes for many compositions, then developing intermediate models linking descriptors to these microstructural parameters.*

*While this would strengthen the mechanistic foundation, it presents practical challenges. Microstructural features depend on processing conditions, requiring careful control across all samples. The resulting models would be specific to the*

*processing route used.*

*Given these constraints, our current approach provides a practical path: use composition-property models for initial screening, then validate through detailed microstructural analysis of promising candidates. The experimental validation succeeded for all four alloys with errors under 5%, including Alloy #1 achieving 18.92 MPa·m$^{1/2}$ fracture toughness, which exceeds all reported as-cast Nb-Si alloys. This demonstrates the approach effectively accelerates alloy discovery. We have revised the manuscript to acknowledge this limitation clearly and explain that mechanistic understanding comes from characterization of predicted compositions rather than being integrated into the predictive framework itself. Future work will focus on developing multi-level composition-microstructure-property predictive models by systematically collecting quantitative microstructural data and establishing hierarchical predictive relationships to strengthen the mechanistic foundation.*

*These details have been added to the* ***Results and Discussion*** *section.*

[Q8] Figure captions must explicitly describe all colors, marker shapes, and data groupings so each figure is fully self-contained.

***Response:***

*We have revised all figure captions to include complete descriptions of colors, markers, and data groupings. For Figure 3, we now specify that blue histograms show training data errors and red histograms show testing data errors, with metrics labeled on each panel. The bar charts in panels e-f include numerical labels for all seven algorithms, with training metrics in blue and test metrics in red.*

*For Figure 4c-f, we clarified that pink colors indicate positive SHAP values that increase the property, while green colors show negative values. Color intensity represents the magnitude of contributions. Pie charts in Figure 4a-b show each feature's percentage contribution.*

*For Figure 6, we added detailed descriptions of phase identification and measurement scales for all panels. The three analyzed regions (γ' phase, interface, Nbss matrix) are clearly labeled. Strain maps include color scales with numerical values. The revised Figure 4 is shown below:*

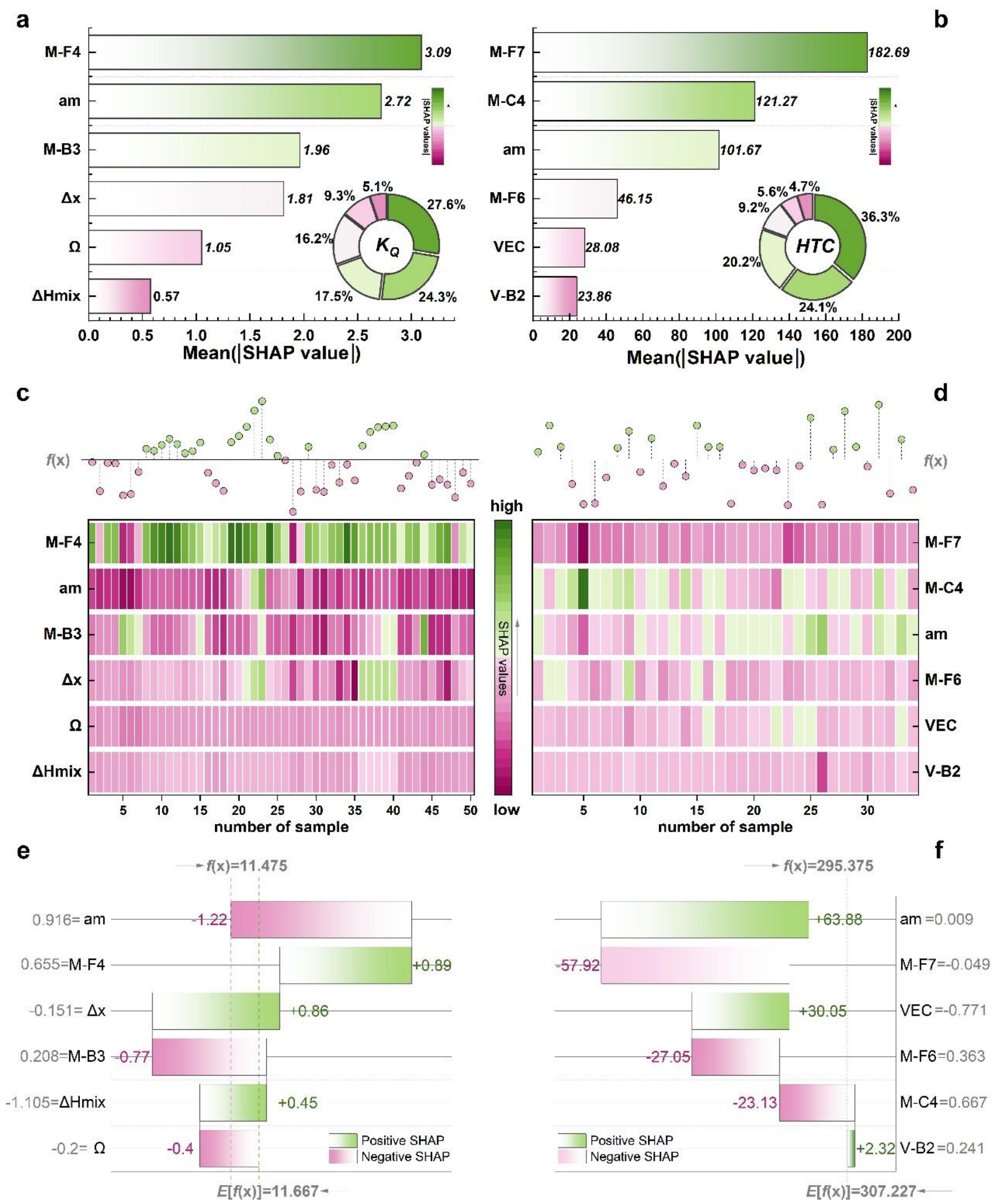


***Fig. 4: SHAP feature importance analysis.*** *(**a** and **b**) Feature contribution rankings and SHAP value distributions for (**a**) $K_Q$ and (**b**) HTC models. (**c** and **d**) SHAP value distribution heatmaps across all samples for (**c**) $K_Q$ and (**d**) HTC models. (**e** and **f**) Single sample feature contribution force plots demonstrating individual feature impacts for (**e**) $K_Q$ and (**f**) HTC predictions.*

[Q9&Q10&Q11] Multipanel figures (especially Figs. 5 and 6) are overly complicated. The authors should split them into simpler, focused plots. What are different fits away from central lines in Figures 3a and b. Figures 3e–f add little insight and could be replaced with straightforward bar charts of feature importances or model errors. Current visualizations are more complex than necessary. Simplify plots to convey key results

clearly—for instance, replace dense scatter panels with bar or line charts where appropriate.

***Response:***

*We understand the concern about complex multipanel figures. For Figures 5 and 6, we respectfully maintain the current layout because they present connected information essential to understanding property enhancement mechanisms. Figure 5 links optimization results with mechanical testing and microstructure evidence for toughening, showing the path from ML predictions to experimental validation. Splitting this would disconnect related information. Similarly, Figure 6 presents multi-scale characterization from micron-scale phases to nanometer-scale interfaces to atomic-scale strain fields that together explain the toughness enhancement. The integrated presentation helps readers see how features at different scales contribute to observed properties.*

*However, we made several improvements for clarity. We reorganized the panel layout in Figure 5 to group related information logically, with optimization results in the top row, mechanical testing data in the middle row, and microstructural characterization in the bottom row. This hierarchical arrangement guides readers naturally from prediction to validation to mechanism. We also improved color consistency across related panels, maintaining the same color scheme for phase identification throughout Figure 6 to avoid confusion when comparing different characterization techniques.*

*We appreciate the reviewer's feedback regarding Figure 3 and have made specific modifications to address the visualization concerns. We replaced the fitting lines in panels a-b with histogram-based error distributions and converted the scatter plots in panels e-f to bar charts displaying $R^2$, RMSE, and MAE values for all seven machine learning algorithms with numerical labels. These changes simplify the figure while preserving essential information and allow readers to quickly identify the best-performing models for both fracture toughness and high-temperature strength predictions. We also enriched the figure captions to include complete descriptions of all visual elements. The revised Figure 3 is shown below:*

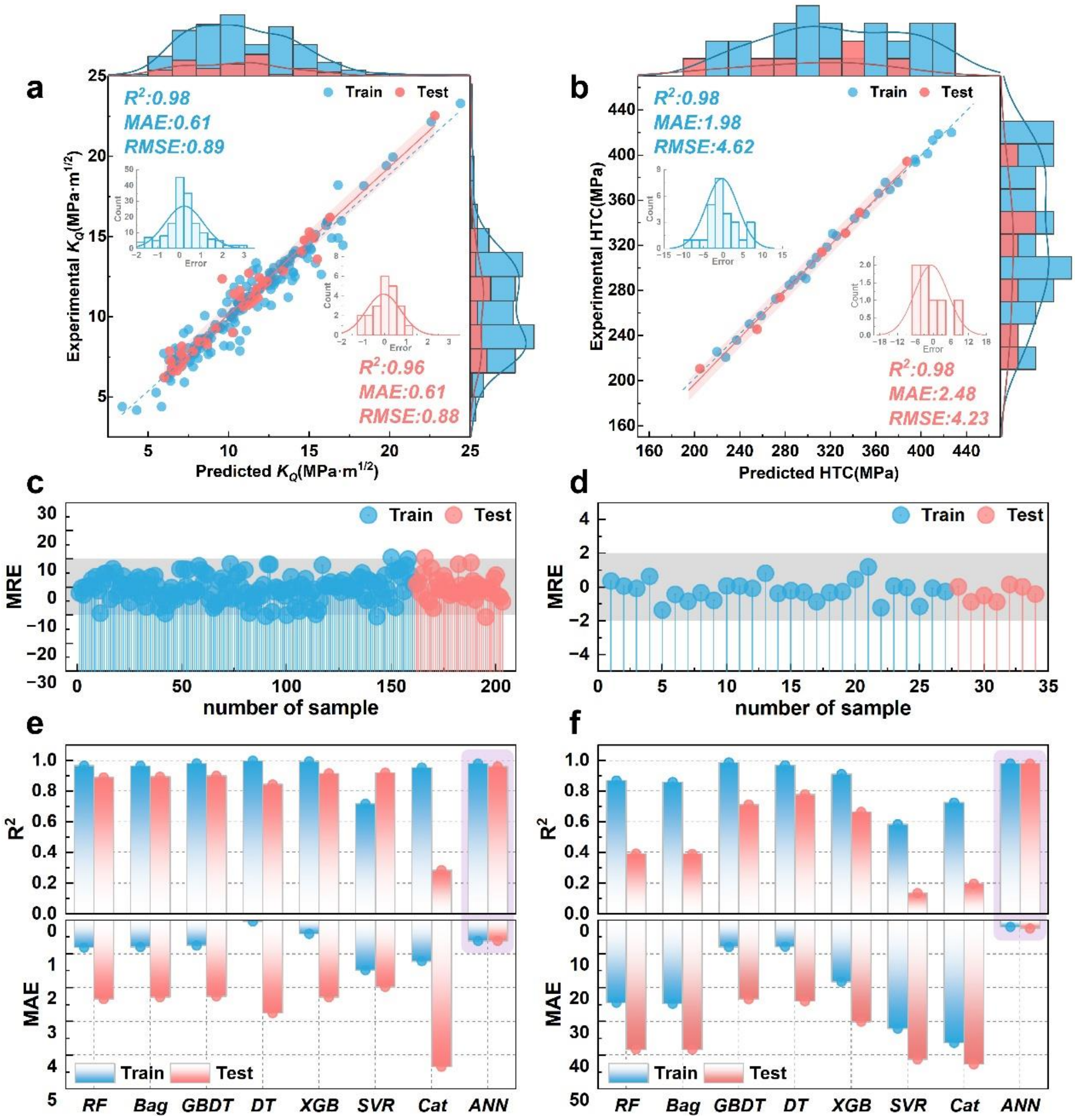


***Fig. 3: Model performance comparison.*** *(**a** to **d**) ANN model training results showing predicted versus experimental values for (**a** and **c**) $K_Q$ and (**b** and **d**) HTC. Training sets in blue and test sets in red. Insets show error distribution histograms (predicted minus experimental values). MRE: Mean Relative Error. (**e** and **f**) Comparative performance metrics ($R^2$, RMSE, MAE) of eight machine learning algorithms including SVR for (**e**) $K_Q$ and (**f**) HTC prediction, with training metrics displayed in blue bars and test metrics in red bars.*

[Q12] Provide full details of the ANN architectures (layer counts, neuron numbers, activation functions, regularization strategies), training protocols (learning rates, early stopping, dropout), and cross-validation schemes. Overall, the methodology section requires significant expansion to allow reproducibility.

***Response:***

*We appreciate the reviewer's request for comprehensive methodological details to ensure reproducibility. Our artificial neural network employs a straightforward two-*

*layer architecture: an input layer (6 neurons for $K_Q$, 6 for HTC), a hidden layer with 450 neurons incorporating batch normalization and ReLU activation, and a single-neuron output layer. The network training utilizes the Adam optimizer with an initial learning rate of 0.01, cosine annealing scheduling (T_max=200), and L2 regularization with weight decay of $1\times10^{-5}$. We deliberately avoided dropout in favor of batch normalization, which proved more effective for our small dataset scenario by stabilizing training while maintaining gradient flow.*

*Our cross-validation strategy differs from traditional k-fold approaches due to the limited sample size, particularly for the HTC dataset. Instead, we implemented an iterative random splitting protocol where the 80:20 train-test partition uses different random seeds across multiple training runs (typically 100+ iterations). This dynamic splitting effectively creates numerous train-test combinations throughout the model development process, providing robust uncertainty quantification. The model continues training until strict dual convergence criteria are satisfied: training set $R^2 > 0.95$ and test set $R^2 > 0.91$, with additional constraints on RMSE (< 0.9 for $K_Q$, < 7 for HTC). Performance metrics are evaluated using rolling averages over 5-iteration windows to reduce random fluctuations, with training terminating upon convergence or reaching a maximum of 1,000,000 epochs.*

*The feature engineering pipeline follows a three-stage process designed specifically for high-dimensional, small-sample scenarios. Stage one applies Pearson correlation coefficient analysis to remove highly collinear features ($|r| > 0.95$), reducing the $K_Q$ feature set from 103 to 67 and HTC from 97 to 65. Stage two combines PCC and maximum information coefficient (MIC) analysis with adjusted thresholds ($K_Q$: PCC > 0.2, MIC > 0.2; HTC: PCC > 0.09, MIC > 0.08) to identify features with both linear and nonlinear relationships to target properties, retaining the top 20 features. Stage three employs recursive feature elimination with ElasticNet regression (alpha=0.01, l1_ratio=0.5) to select the optimal 6-feature subset based on cross-validated prediction performance. All features undergo standardization using StandardScaler fitted exclusively on training data to prevent information leakage.*

*We have substantially expanded the* ***Materials and Methods*** *section and added a* ***Code Availability*** *statement.*

**Reviewer #2**

Overall

The article focuses on Nb-Si alloys, aiming at the optimization of fracture toughness and high-temperature strength. By integrating a three-step feature selection process, SHAP analysis, and NSGA-II multi-objective optimization, a Nb-12.26Si-21.35Ti-1.98Al-1.96Cr-0.51Hf-4.34Zr-4.35V alloy was designed. Microstructural observations and analyses were conducted to elucidate the primary reasons behind the alloy's superior performance. In my view, however, the design methodology adopted here is fairly conventional. The work essentially represents an application of well-established approaches to a specific material system, and the reported performance and mechanisms are not particularly striking. Consequently, the manuscript does not demonstrate sufficient novelty. Following are some specific suggestions:

We thank the reviewer for the thorough evaluation and constructive feedback. We acknowledge the perspective that our methodology is "fairly conventional" and represents "an application of well-established approaches." This candid assessment has prompted us to clarify the scope and contribution of our research.

***Response:***

*We fully agree that individual techniques, including feature screening, SHAP analysis, and NSGA-II optimization, are established methods in materials informatics. We do not claim methodological novelty. Rather, our contribution lies in demonstrating how systematic integration of these techniques effectively addresses a longstanding specific challenge: the inherent toughness-strength trade-off in Nb-Si alloys that has limited practical applications for decades. Review of existing literature reveals that while individual techniques have been occasionally employed, comprehensive integration specifically targeting this trade-off remains limited, and previous studies lack systematic connection between ML-identified features and underlying microstructural mechanisms.*

*In response to valuable comments, we have made substantial revisions: (1) clarified in the Introduction that our focus is systematic integration of established methods for resolving the toughness-strength trade-off, not methodological novelty; (2) established explicit mechanistic linkages between each ML-identified feature and corresponding microstructural characteristics, demonstrating that superior properties result from physically meaningful relationships rather than empirical correlations; (3) expanded methodology with comprehensive details and provided complete datasets and code for*

*full reproducibility.*

*The practical significance is demonstrated by experimental results: the optimized alloy achieves record-breaking as-cast fracture toughness of 18.92 MPa·m$^{1/2}$ while maintaining 322 MPa high-temperature strength at 1250°C, surpassing all previously reported as-cast Nb-Si alloys. We believe these revisions have strengthened the manuscript by clearly positioning our contribution as a systematic, integrated application that successfully addresses a specific and important materials challenge, validated through comprehensive experimental characterization.*

Major Comments:

[Q1] The authors claim to have employed "a three-step progressive feature screening strategy" for feature selection. However, such an approach is already well established and widely adopted. This does not demonstrate any incremental methodological contribution in terms of machine learning.

***Response:***

*The reviewer is correct that the individual techniques we used (PCC, MIC, RFE) are well-established methods in materials informatics. We do not claim these are new. Our contribution is demonstrating how systematic application of these methods can address the specific challenge of Nb-Si ultra-high temperature alloy design, particularly the trade-off between room-temperature fracture toughness and high-temperature strength that has limited materials development.*

*The challenges we addressed include high feature dimensionality relative to sample size (over 200 features from 12+ elements versus 203 samples for $K_Q$ and 34 for HTC), strong collinearity among element-derived features due to similar properties, both linear and nonlinear relationships requiring hybrid feature selection, and multi-objective optimization between competing properties. Our three-step integration successfully reduced the feature space while achieving $R^2$ above 0.96 for both properties. This enabled identification of an alloy composition with record-breaking as-cast fracture toughness of 18.92 MPa·m$^{1/2}$ while maintaining high-temperature strength of 322 MPa at 1250°C.*

*We have revised the manuscript to remove claims of methodological novelty. Instead we emphasize that we are applying established materials informatics techniques to address a longstanding challenge in Nb-Si alloy development. The contribution is demonstrating that systematic integration of these methods can accelerate discovery of ultra-high temperature alloys from years to months, as validated by achieving*

*properties that surpass all previously reported as-cast Nb-Si alloys in the literature. We have made the corresponding revisions in the* ***Introduction****.*

[Q2] A key issue lies in the complete disconnection between the machine learning-guided alloy design and the subsequent mechanistic understanding. The manuscript does not present any discussion linking the "key factors identified by the ML model with the proposed strengthening mechanisms". Establishing such a connection is crucial; otherwise, it remains unclear whether the reported alloy design is an inherent outcome of the model or simply a matter of coincidence.

***Response:***

*We appreciate the reviewer's critical observation about the connection between ML-identified features and mechanistic understanding. The reviewer is correct that our original manuscript did not adequately establish this linkage, which is essential to demonstrate that the alloy design results from physical principles rather than coincidence.*

*We have added a new section in the Results and Discussion that explicitly connects each ML-identified key feature to the observed microstructural mechanisms. For the fracture toughness model, the most important feature identified by SHAP analysis is melting enthalpy (M-F4), which showed that lower melting enthalpy values (below 28.5 kJ/mol) contribute positively to toughness. Our TEM characterization of the optimized alloy reveals the physical basis for this relationship: the melting enthalpy directly influences the nucleation and growth kinetics of the γ'-$Nb_5Si_3$ phase during solidification. Lower melting enthalpy corresponds to reduced thermodynamic driving force for silicide formation, which promotes formation of finer and more dispersed γ' precipitates rather than coarse continuous networks. The measured average particle size of 94.88 nm in our optimized alloy confirms this mechanism. This nanoscale dispersion is critical because it forces propagating cracks to repeatedly deflect around particles and interact with the ductile Nbss matrix, as directly observed in our fracture surface analysis showing crack bridging and deflection patterns.*

*The second critical feature, atomic radius mismatch (am), showed positive contribution to toughness when exceeding 350 pm. Our high-resolution TEM analysis reveals that this feature directly correlates with the interface structure between phases. The measured 67.6% lattice mismatch at Nbss/γ' interfaces, which is among the highest reported for Nb-Si alloys, creates highly incoherent interfaces with significant strain fields. Our geometric phase analysis quantified 6-8% strain gradients across these*

*interfaces. While the reviewer's concern about such interfaces potentially acting as crack initiation sites is valid for coarse microstructures, in our system the combination of nanoscale particle dispersion (spacing of 200-300 nm) and the high density of incoherent interfaces (approximately 5.2 $m^2/cm^3$) creates a different scenario. The crack must continuously change direction when encountering these interfaces, increasing the effective crack path length. This connection between the ML-identified atomic radius mismatch parameter and the observed lattice mismatch and crack deflection behavior demonstrates that the model captured physically meaningful relationships.*

*For high-temperature strength, the dominant ML feature is specific heat capacity (M-F7), which affects thermal stability during high-temperature deformation. Our EBSD analysis after compression at 1250°C shows this manifests as differential strain accumulation between phases: the γ phase exhibits higher kernel average misorientation values than the Nbss phase, indicating it maintains structural integrity and continues to provide load-bearing capacity while the Nbss matrix undergoes dynamic recovery. The covalent radius feature (M-C4) identified by the model correlates with the specific crystallographic orientation relationships we observed: {110}Nbss//{10-10}γ and {100}Nbss//{11-20}γ. These orientation relationships, which form due to interfacial energy minimization governed by atomic size compatibility, enable geometric matching between slip systems of the two phases and facilitate coordinated deformation.*

*The alloy design is not coincidental, as the SHAP analysis guided element selection (Cr, Hf, Zr, V) based on their influence on these key features, and our microstructural characterization confirms these elements partition to create the observed phase distribution and interface characteristics. The dispersed nanoscale γ'-$Nb_5Si_3$ precipitates (average size 94.88 nm) provide the microstructural foundation for both toughening mechanisms (crack bridging and deflection) and high-temperature strengthening (fine γ phase grains of 5.33 μm vs. 8.85 μm for Nbss). The specific orientation relationships between Nbss and γ phases ({110}Nbss//{10-10}γ and {100}Nbss//{11-20}γ) observed in our EBSD analysis facilitate deformation coordination at high temperatures, which is consistent with the ML model's identification of atomic radius mismatch as a key feature.*

*We have added the section "**Connecting ML-identified features to microstructural mechanisms**" to the **Results and Discussion**.*

[Q3] The Introduction is poorly written. For instance, the statement "it is difficult to meet the requirements of processing and assembly, and there is an inherent competition between room-temperature fracture toughness and high-temperature strength" is merely descriptive and provides little scientific insight to the reader. The authors should articulate the fundamental scientific origins of this trade-off. Moreover, following the overview of machine learning applications, the necessity of using ML to specifically address the toughness–strength trade-off remains unconvincing. A more targeted review of progress in multi-objective optimization with ML, and an explanation of why ML can effectively assist in resolving this issue, would be far more relevant to the manuscript's central theme, rather than a generic summary such as "machine learning-guided development of high entropy alloys with optimized hardness and strength-ductility balance."

***Response:***

*We appreciate the reviewer's constructive feedback on improving the Introduction. We have substantially revised the first paragraph to provide the fundamental scientific origins of the toughness-strength trade-off as requested.*

*Ultra-high temperature Nb-Si based alloys face a critical challenge: achieving adequate room-temperature fracture toughness (>18 MPa·$m^{1/2}$) for processing while maintaining exceptional high-temperature strength. This trade-off has fundamental origins in the alloy microstructure. High-temperature strength primarily relies on the γ-$Nb_5Si_3$ silicide phase, which provides thermal stability through its high melting point and ordered D8m crystal structure. However, this phase has limited slip systems at room temperature, making it inherently brittle. Increasing the silicide volume fraction improves creep resistance at elevated temperatures through load-bearing and dislocation pinning mechanisms, but simultaneously increases crack propagation susceptibility at room temperature because cracks preferentially propagate through or along these brittle intermetallic phases. This creates an inherent design constraint where improvements in one property typically degrade the other.*

*Traditional Nb-Si alloy development has primarily relied on empirical trial-and-error methods guided by phase diagram calculations and microstructural observations. These sequential approaches struggle to systematically explore the complex multi-dimensional trade-off surface across the composition space. The emergence of materials informatics has transformed this challenge. Machine learning-based multi-objective optimization, particularly when combined with interpretability analysis, offers specific advantages for resolving property trade-offs. Multi-objective algorithms*

*such as NSGA-II can simultaneously evaluate thousands of potential compositions to identify Pareto-optimal solutions, which represent the frontier where improving one property necessarily requires sacrificing the other. Interpretability methods like SHAP analysis quantify how individual compositional features affect both properties, revealing which elements can enhance one property without severely degrading the other. Recent successes include adaptive design strategies for NiTi-based shape memory alloys that balanced transformation temperature and mechanical hysteresis, and concurrent optimization in refractory high-entropy alloys that addressed strength-ductility trade-offs. For Nb-Si alloys specifically, the limited experimental dataset makes exhaustive exploration impractical, but machine learning can extract patterns from existing data to guide targeted experiments toward the optimal balance.*

*However, systematic integration of these techniques for Nb-Si based alloys remains limited. Existing studies have employed machine learning methods in isolation without comprehensive feature screening, interpretability analysis, or validation of the mechanisms underlying predicted property improvements. More critically, the specific toughness-strength trade-off in Nb-Si alloys has not been systematically addressed through integrated data-driven approaches.*

*We have made the corresponding revisions in the* ***Introduction****.*

[Q4] The introduction identifies four issues in applying machine learning to Nb-Si-based alloys: (1) lack of systematic feature engineering methods; (2) insufficient model interpretability; (3) absence of multi-objective optimization strategies; and (4) limited integration of mechanistic understanding with experimental validation. These issues appear to be derived from the methods used or the work conducted in this paper rather than from a thorough analysis of the field's literature. The fields of feature engineering, interpretability, multi-objective optimization, and multi-scale microstructural analysis have all developed over many years. For instance, the feature selection method used in the paper has been applied to Ni-Ti-based alloys, copper alloys, and other alloy designs since 2016 [Nature Communications, 2016, 7: 11241; Acta Materialia, 2020, 200: 803-810]. Similarly, SHAP methods and multi-objective optimization have been widely used [Computational Materials Science, 2022, 211: 111560; Materials Today Communications, 2021, 28: 102709], and multi-scale microstructural analysis is a standard approach in alloy microstructure analysis, from micron-scale grains to nanoscale precipitates.

***Response:***

*We appreciate the reviewer's insightful observation regarding our framing of these four challenges. The reviewer is correct that feature engineering, SHAP analysis, multi-objective optimization, and microstructural characterization are well-established techniques in materials informatics, as evidenced by successful applications in Ni-Ti alloys, copper alloys, and other systems cited by the reviewer.*

*We need to clarify that our introduction was not intended to claim these as novel methodological developments. Rather, we aimed to emphasize the limited systematic application of these techniques specifically to Nb-Si based alloys. The structure of our introduction serves a specific purpose: to establish a clear correspondence with the subsequent Results and Discussion sections. Each of the four aspects mentioned in the introduction directly maps to a major section of our work. Feature screening corresponds to our three-step selection process, interpretability corresponds to our SHAP analysis, multi-objective optimization corresponds to our NSGA-II implementation, and mechanistic understanding corresponds to our TEM/EBSD characterization. This structural organization helps readers understand the logic flow from identifying what needs to be done to demonstrating how we did it.*

*A review of the existing Nb-Si alloy literature reveals that while individual techniques have been occasionally employed, comprehensive integration remains sparse. Liu et al. (Mater. Des., 2017, 129: 210-218) applied artificial neural networks but without interpretability analysis or systematic feature screening. Ma et al. (Int. J. Refract. Met. Hard Mater., 2023, 117: 106420) used machine learning for fracture toughness prediction but did not address multi-objective optimization between competing properties. Yin et al. (Next Materials, 2025, 6: 100294) recently attempted multi-objective design but relied on limited feature sets without systematic screening from broader compositional descriptors. The central challenge we sought to address is not the absence of these techniques per se, but rather the absence of their integrated application to resolve the specific trade-off between room-temperature fracture toughness and high-temperature strength in Nb-Si alloys.*

*We have revised the introduction to better reflect this context. The revised version acknowledges these as established methods in materials informatics while making clear that our focus is their systematic integration specifically for addressing the toughness-strength trade-off in Nb-Si alloys. We have adjusted the language to avoid any implication of methodological novelty, while maintaining the four-part structure that provides a logical framework for the subsequent sections. The experimental validation demonstrates the practical value of this integrated framework for this specific alloy*

*system by achieving record-breaking as-cast fracture toughness of 18.92 MPa·m$^{1/2}$ while maintaining high-temperature strength.*

*We have made the corresponding revisions in the* ***Introduction****.*

[Q5] There are methodological flaws in the feature selection process. Specifically, the authors write: "Finally, the RFE (recursive feature elimination) method was adopted, using ElasticNet regression as the base model to select the optimal subset from 20 features (Fig. 2e, f)." Since ElasticNet is a linear model, the RFE process inherently favors features with direct linear correlations, while potentially discarding those that have nonlinear effects. This poses a serious concern, as the subsequent predictive models (RF, GBDT, ANN) are nonlinear. Consequently, the features fed into these models may already be deficient in information. Although the ANN shows sufficient generalization capability, this technical issue could very well explain the relatively poor performance of RF and GBDT, rather than a superficial attribution to "overfitting."

***Response:***

*We appreciate the attention to feature selection methodology. We want to clarify that our approach specifically addresses the concern about nonlinear relationships. As stated in our manuscript with Figure 2c-d, we used a hybrid strategy where PCC captures linear relationships and MIC identifies nonlinear correlations, with their combination providing feature importance.*

*MIC was explicitly included to detect nonlinear feature-target relationships before RFE. In our implementation, features must satisfy both PCC and MIC thresholds simultaneously. For $K_Q$ this means PCC >0.2 and MIC >0.2. For HTC the thresholds are PCC >0.09 and MIC >0.08. This dual-threshold approach ensures we retain features with strong statistical associations regardless of whether the relationship is linear or nonlinear. Features showing only weak linear correlation but strong nonlinear association are retained, as are features with strong linear correlation.*

*The subsequent RFE step with ElasticNet selected the optimal subset from these 20 pre-validated features based on predictive performance in cross-validation. While ElasticNet is a linear model, it operates on features already screened for both linear and nonlinear relevance. The RFE process evaluates feature subsets by training ElasticNet models and measuring cross-validated prediction accuracy, testing which combination of the 20 pre-screened features yields best predictive performance.*

*The final prediction models including RF, GBDT, and ANN are all nonlinear and can learn complex feature interactions. All three used the identical 6 features selected*

*through this process yet showed different results with test $R^2$ of 0.88, 0.89, and 0.96 respectively for $K_Q$ prediction. If feature inadequacy from linear screening were the main issue, we would expect similar degradation across all nonlinear models. The superior ANN performance combined with its training methodology suggests differences stem from model architecture and optimization rather than feature deficiencies.*

*We have clarified this multi-stage methodology in the revised Materials and Methods section, emphasizing that MIC screening before RFE captures nonlinear relationships, the linear RFE step operates on features pre-validated for both linear and nonlinear relevance, and final nonlinear models can fully exploit complex feature interactions. These details have been added to the* ***Materials and Methods*** *section.*

[Q6] When studying fracture toughness and high-temperature strength, the paper mentions constructing two datasets with 203 and 34 data points, respectively. However, Figure 3c shows a number of data points far exceeding 203, and Figures 3b and 3d show 36 and 35 data points, respectively, which is inconsistent.

***Response:***

*We thank the reviewer for pointing out this apparent inconsistency. We want to clarify that the HTC dataset contains exactly 34 samples, as originally stated in our manuscript. This number is correct and has been used consistently in all our analyses and model training. The database was constructed through systematic literature review, and we collected all available data points for as-cast Nb-Si alloys tested at 1250°C that met our quality criteria.*

*The reviewer may have been confused by references to 36 in some figures or earlier drafts, but we confirm that the actual dataset size is 34 samples for HTC. This is reflected in all our statistical analyses, train-test splits (27 training, 7 testing with 80:20 ratio), and model performance evaluations. We have carefully reviewed the entire manuscript and all supplementary materials to ensure this number (34) is used consistently throughout. Any discrepancy in figures has been corrected to accurately display 34 data points for the HTC dataset.*

*We apologize if there was any confusion, and we have verified that all results, including model training outcomes and conclusions, are based on the actual 34-sample HTC dataset. The manuscript text and all figures now consistently state 34 samples for HTC. The revised Figure 3(a-d) is shown below:*

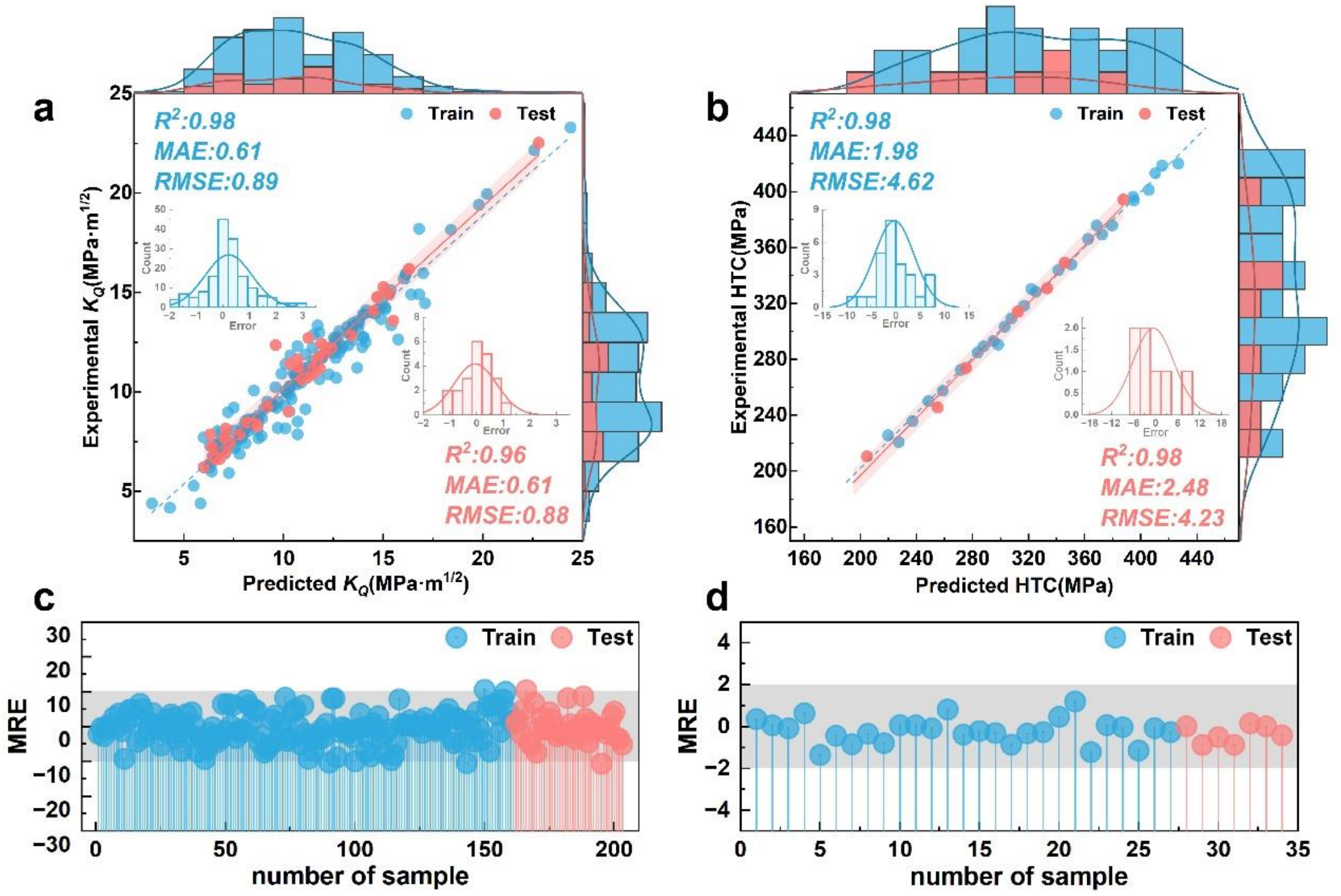


*Fig. 3: Model performance comparison.* (***a*** *to* ***d***) *ANN model training results showing predicted versus experimental values for (**a** and **c**) $K_Q$ and (**b** and **d**) HTC. Training sets in blue and test sets in red. Insets show error distribution histograms (predicted minus experimental values) MRE: Mean Relative Error.*

[Q7] The paper focuses on the effectiveness of feature selection, SHAP analysis, and NSGA-II results but lacks an analysis of why these methods are effective in Nb-Si alloy design, especially since the design of Nb-Si alloys involves a small dataset with the two performance prediction datasets containing 203 and 34 data points, respectively.

***Response:***

*We address the effectiveness of our methods for the small dataset scenario through the following analysis. The three-step feature selection strategy is specifically designed for small datasets to prevent overfitting. Starting with 200 initial features and reducing to 6 key factors decreases the feature-to-sample ratio from 1:1 (200 features for 203 $K_Q$ samples) to 1:34, which substantially improves model generalization. The first step removes redundant features (PCC threshold 0.95), reducing $K_Q$ features from 103 to 67 and HTC features from 97 to 65. The second step uses PCC and MIC jointly to capture both linear and nonlinear relationships with target properties. The third step employs RFE with ElasticNet, which combines L1 and L2 regularization. This approach is particularly suitable for small datasets as it performs feature selection while preventing overfitting. Our neural network architecture includes batch*

*normalization and weight decay (1e-5) as additional regularization. The model achieves $R^2$>0.96 with prediction errors below 5%, and the t-SNE analysis (Supplementary Fig. 10) demonstrates extrapolation capability beyond the training data distribution.*

*SHAP analysis provides interpretability by quantifying each feature's contribution to predictions, which is essential for materials design. For the $K_Q$ model, SHAP reveals that M-F4 (melting enthalpy) and am (atomic radius mismatch) contribute 55.3% of prediction influence, with nonlinear dependency patterns showing M-F4 has positive contribution below 28.5. This physical insight guides element selection: elements are ranked by weighted SHAP coefficients, identifying Cr, Hf, Zr, and V as optimal additions. NSGA-II effectively handles the multi-objective optimization between $K_Q$ and HTC through non-dominated sorting and crowding distance selection. The algorithm explores 500 individuals over 250 generations using SBX crossover (η=15) and polynomial mutation (η=20), generating Pareto fronts that show the trade-off relationship. Experimental validation confirms this approach: the optimized 8-element alloy achieves 18.92 MPa·$m^{1/2}$ $K_Q$ and 322 MPa HTC at 1250°C, with prediction errors under 5% for all four tested compositions. The combination of feature selection, SHAP-guided element selection, and NSGA-II optimization enables efficient exploration of the composition space despite the limited training data.*

*These details have been added to the* ***Materials and Methods*** *section.*

[Q8] The manuscript should clarify why alloys #2 and #4 deviate so far from the training set distribution in the t-SNE plot. Were additional elements introduced? Or did certain element concentrations exceed the range present in the current dataset? Please provide either the exact compositions of the designed alloys or reasonable compositional ranges.

***Response:***

*We thank the reviewer for this question about the t-SNE analysis. The deviation of alloys #2 and #4 from the training distribution reflects the model's extrapolation capability due to specific compositional features rather than introducing additional elements. All four designed alloys use only elements present in the training dataset (Nb, Si, Ti, Al, Cr, Hf, Zr, V), but their compositions extend beyond typical training ranges in certain aspects. We have added the exact atomic compositions in revised Supplementary Table 5.*

*Alloy #2 (7-element: Nb-15.06Si-26.16Ti-1.97Al-1.98Cr-0.71Hf-4.64Zr) has Ti content*

*of 26.16 at.%, which exceeds the upper range of most training samples (typically 0-24 at.% as shown in Supplementary Figure 1c).*

*Additionally, the combination of high Zr content (4.64 at.%) with elevated Ti creates a feature space location underrepresented in training data. The t-SNE algorithm, which preserves local neighborhood structures, places this composition in a distinct region because few training samples have similarly high Ti-Zr combinations.*

*Alloy #4 (5-element: Nb-12.59Si-35.00Ti-1.77Al-2.58Cr) has the highest Ti content at 35.00 at.% among all samples, well beyond the training data distribution. Furthermore, it contains only 5 elements compared to the dominant 6-7 element alloys in the training set (Supplementary Figure 2a). This combination of extreme Ti content and reduced elemental complexity creates a unique position in feature space that t-SNE identifies as distant from the training cluster.*

*The successful experimental validation of these extrapolated compositions is significant. Alloy #2 achieved 356.64 MPa HTC with prediction of 361.12 MPa (error 2.16%), and Alloy #4 achieved 256.57 MPa HTC with prediction of 259.49 MPa (error 1.14%). This demonstrates the model learned meaningful composition-property relationships that generalize beyond exact training distribution rather than simply memorizing training patterns.*

*This analysis has been added to the* ***Multi-objective optimization and Experimental validation section*** *in* ***Results and Discussion****.*

[Q9] The statement "This pre-stress state can alleviate stress concentration effects at crack tips and suppress further crack propagation" is not acceptable. Given the large stress fields and the orientation mismatch across incoherent interfaces, such features are more likely to serve as crack initiation sites and accelerate crack growth. The authors provide no prior evidence, nor experimental or computational support, for this claim.

***Response:***

*We thank the reviewer for this critical comment about our interpretation of interfacial strain gradients. The reviewer is correct that our original statement about pre-stress fields alleviating stress concentration was not adequately supported. We have removed this claim and clarified the actual role of these interface characteristics. Our GPA analysis reveals significant strain gradients (6-8%) at Nbss/γ' interfaces resulting from 67.6% lattice mismatch, indicating highly incoherent interfaces. The reviewer correctly points out that such high-energy interfaces could serve as crack initiation sites.*

*However, the key to understanding their role lies in considering both the interface characteristics and the microstructural length scale together.*

*The high-energy incoherent interfaces in our system contribute to toughening through crack deflection rather than serving as crack initiation sites. Our fracture surface observations in Figure 5g reveal two distinct mechanisms. First, crack bridging (Figure 5$g_1$) occurs when the ductile Nbss matrix forms ligaments spanning crack surfaces, providing resistance through plastic deformation. Second, crack deflection (Figure 5$g_2$) shows that cracks change direction at phase boundaries rather than propagating straight through. The large lattice mismatch creates energy barriers at these interfaces that promote crack deflection. When a propagating crack encounters such interfaces, the energy mismatch causes the crack to deflect along or around the interface rather than continuing straight through.*

*The critical factor determining whether high-energy interfaces act as beneficial deflection sites or detrimental crack paths is the nanoscale dispersion of γ′ particles with average size 94.88 nm. At this length scale, cracks cannot form continuous paths through connected brittle phases. Each deflection event is followed by crack propagation through the Nbss matrix for only a short distance (typically 200-300 nm based on particle spacing) before encountering another deflecting interface. This repeated deflection increases the total crack path length and dissipates energy, a mechanism well documented in ceramic and intermetallic systems where finely dispersed second phases force cracks to follow tortuous paths [1-3]. The key contrast is with coarsely dispersed systems. When particle spacing exceeds several microns, high-energy interfaces can connect to form continuous weak paths for rapid crack propagation, as observed in intermetallic composites with large ductile-phase reinforcements [4,5]. Our nanoscale dispersion prevents such connectivity, which explains why the ML model's identification of atomic radius mismatch as a key feature has physical meaning: it predicts the degree of interface incoherence needed to promote deflection while the composition optimization maintains the fine dispersion that prevents detrimental crack paths.*

*To quantitatively demonstrate the crack deflection mechanism, we performed systematic crack path analysis comparing alloy #1 with alloy #2 (Supplementary Figure 13). Digital image processing at 1 pixel = 1 μm resolution followed by linear regression analysis yielded correlation coefficient R (representing crack path linearity) and deflection parameter D = 1/R (quantifying tortuosity).*

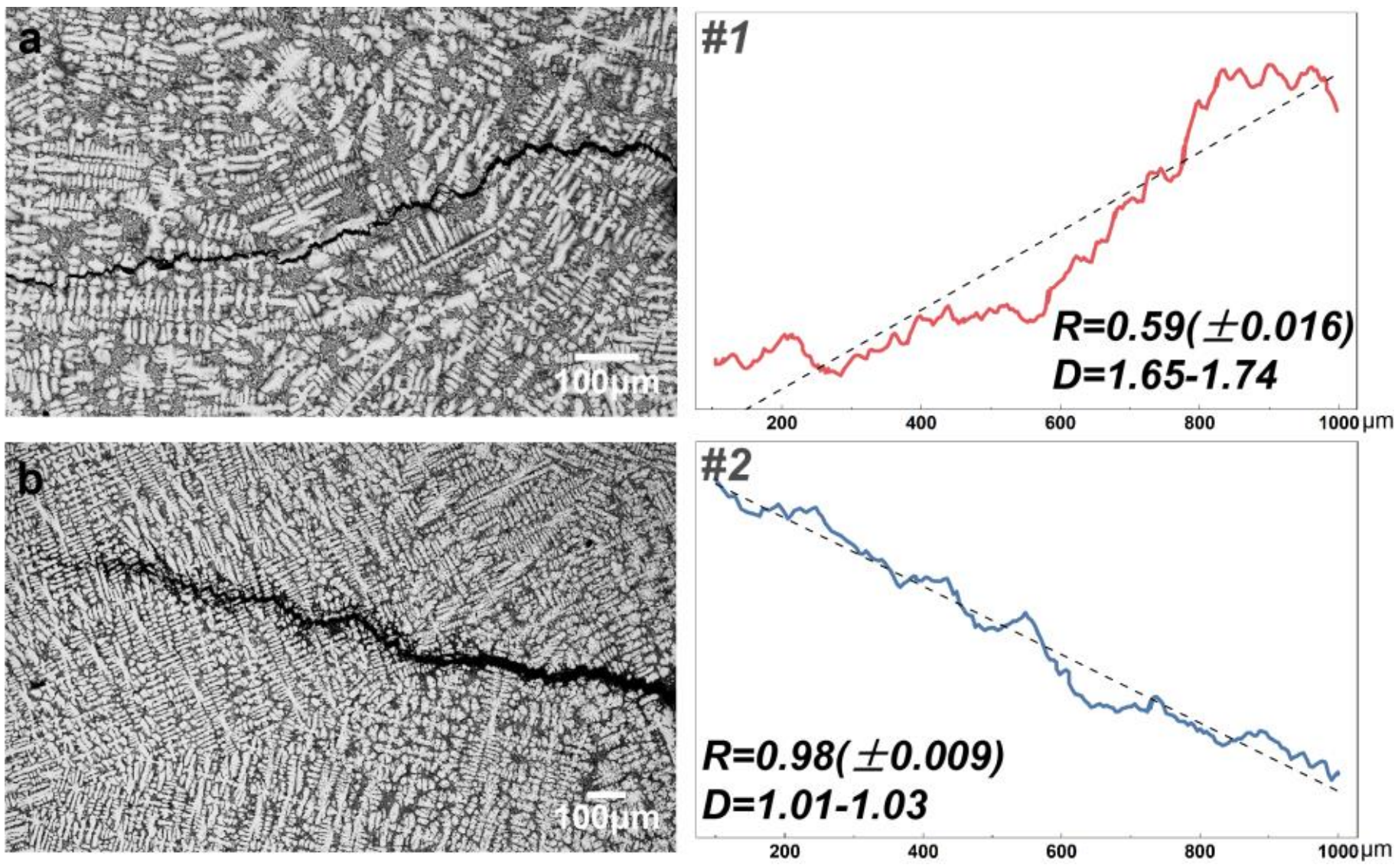


***Supplementary Fig. 13: Quantitative crack deflection analysis.*** *(**a** and **b**) SEM images showing crack propagation paths with digitized trajectories (solid lines) and linear fits (dashed lines) for (**a**) sample #1 and (**b**) sample #2.*

*The quantitative results show that alloy #1 exhibits R = 0.59 (±0.016) and D = 1.65-1.74, indicating highly tortuous crack propagation. In contrast, alloy #2 shows R = 0.98 (±0.009) and D = 1.01-1.03, representing nearly linear crack trajectory. The 63% increase in D-value from alloy #2 to alloy #1 provides direct quantitative evidence that the nanoscale γ'-$Nb_5Si_3$ dispersion (average particle size 94.88 nm with inter-particle spacing of 200-300 nm) combined with high-energy incoherent interfaces (67.6% lattice mismatch) effectively promotes crack deflection.*

*We have revised the **Fracture toughness enhancement mechanisms** section in **Results and Discussion** accordingly.*

## *References*

*[1] Faber, K. T. & Evans, A. G. Crack deflection processes—I. Theory. Acta Metall. 31, 565-576 (1983).*

*[2] Gao, H., Ji, B., Jäger, I. L., Arzt, E. & Fratzl, P. Materials become insensitive to flaws at nanoscale: Lessons from nature. Proc. Natl. Acad. Sci. USA 100, 5597-5600 (2003).*

*[3] Zimmermann, E. A. & Ritchie, R. O. Bone as a structural material. Adv. Healthcare Mater. 4, 1287-1304 (2015).*

*[4] Venkateswara Rao, K. T., Soboyejo, W. O. & Ritchie, R. O. Ductile-phase toughening and fatigue-crack growth in Nb-reinforced molybdenum disilicide intermetallic composites. Metall. Trans. A 23, 2249-2257 (1992).*

*[5] Ritchie, R. O. Mechanisms of fatigue-crack propagation in ductile and brittle solids. Int. J. Fract. 100, 55-83 (1999).*

Minor

[Q1] Please ensure consistency in the use of Fig. vs. Figure throughout the manuscript.

***Response:***

*We thank the reviewer for pointing out this formatting issue. We have reviewed the entire manuscript and standardized figure references. In parenthetical citations within sentences we use "Fig." (e.g., "as shown in Fig. 3a"). When beginning a sentence we use "Figure" (e.g., "Figure 3 shows..."). In figure captions we use "Fig." for cross-references and "Figure" for the caption title. This convention has been applied consistently throughout both the main manuscript and supplementary materials following standard journal formatting.*

[Q2] In Fig. 3a–b, legends distinguishing training and test sets are missing, and what do the three parts of the points represent? In Fig. 3c–d, the abbreviation MRE should be defined. While Fig. 3e–f appears visually appealing, there are no numerical references provided, making it impossible to estimate the prediction accuracy from the figures alone.

***Response:***

*We thank the reviewer for the constructive suggestions regarding Figure 3. We have made the following revisions to address these concerns:*

*First, we have added clear legends distinguishing training and test sets in Figure 3a-b. We have removed the scattered fitting lines and replaced them with histogram visualizations showing the error distribution between predicted and experimental values for both training and test datasets. This modification provides clearer information about model accuracy while eliminating potential confusion from multiple fitting lines. Second, we have defined the abbreviation MRE (Mean Relative Error) in the figure caption. Third, we have redesigned Figure 3e-f by converting the multi-panel scatter plots to more intuitive bar charts. The revised Figure 3 is shown below:*

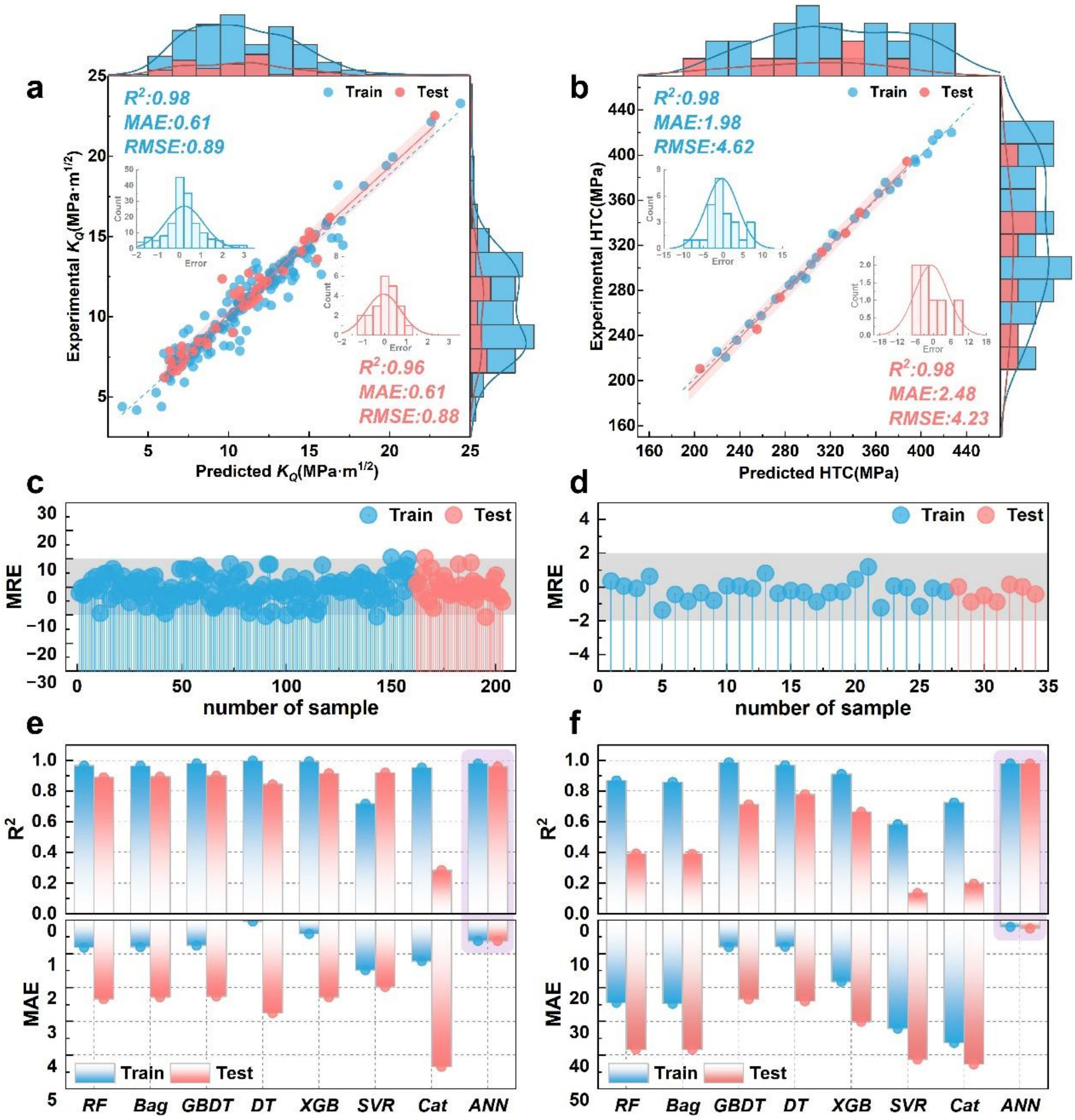


***Fig. 3: Model performance comparison.*** *(**a** to **d**) ANN model training results showing predicted versus experimental values for (**a** and **c**) $K_Q$ and (**b** and **d**) HTC. Training sets in blue and test sets in red. Insets show error distribution histograms (predicted minus experimental values). MRE: Mean Relative Error. (**e** and **f**) Comparative performance metrics ($R^2$, RMSE, MAE) of eight machine learning algorithms including SVR for (**e**) $K_Q$ and (**f**) HTC prediction, with training metrics displayed in blue bars and test metrics in red bars.*

[Q3] There is a lack of necessary description of the data partitioning method in the approach. A crucial point is whether the test set used in your data partitioning process can reflect the generalization ability of the model for the properties of unknown alloys.

***Response:***

*We thank the reviewer for this valuable comment and have added a comprehensive description of our data partitioning methodology in the revised manuscript. We employed a random splitting approach with an 80:20 train-test ratio, where the model*

*was iteratively trained with different random seeds until rigorous convergence criteria were met (training $R^2 > 0.95$, test $R^2 > 0.91$, RMSE < 0.9 for both sets). This method is well-suited to our dataset, which spans a continuous compositional space across multiple alloying elements without distinct categorical boundaries. The random splitting ensures both training and test sets contain representative samples distributed throughout the entire compositional range studied.*

*Regarding the test set's ability to reflect generalization performance for unknown alloys, our approach demonstrates several strengths. The test set constitutes 20% of randomly selected samples and provides robust assessment of the model's interpolation capability within the explored compositional space. We have added distribution visualizations (Supplementary Fig. 4 and 5) showing that training and test sets exhibit highly consistent distributions across all six selected features. The test samples are well-covered within the main distribution regions of the training data, without shifts, outliers, or isolated regions, indicating reasonable data partitioning with good representativeness. This validation approach ensures that model predictions for alloy compositions within the studied range can be reliably assessed. Experimental validation of four predicted compositions achieved errors below 5% with an average of 2.4%, including the best-performing alloy achieving fracture toughness of 18.92 MPa·$m^{1/2}$ that surpasses all previously reported as-cast Nb-Si alloys.*

*These methodological details have been incorporated into the* ***Materials and Methods*** *of the revised manuscript.*

[Q4] The Supplementary Information does not include any details about the model parameter settings. In the absence of shared code, it is essential to provide at least sufficient details of the parameterization to enable meaningful assessment of the models.

***Response:***

*We appreciate the request for detailed model parameter documentation. We have added comprehensive Supplementary Tables 12 and 13 systematically organizing all model parameters as shown below:*

***Supplementary Table 12 Hyperparameters for eight ML algorithms.*** *Configurations for RF, XGBoost, GBDT, CatBoost, Bagging, DT, SVR, and ANN. Identical feature sets and data splits used for fair comparison. ANN selected via grid search and cross-validation.*

| *parameter* | *RF* | *XGBoost* | *GBDT* | *CatBoost* | *Bagging* | *DT* | *SVR* | *ANN* |
|---|---|---|---|---|---|---|---|---|
| *Number of Trees/Neurous* | *100* | *100* | *100* | *1000* | *10* | *1* | *-* | *450* |
| *Learning Rate* | *-* | *0.3* | *0.1* | *0.03* | *-* | *-* | *-* | *0.01* |
| *Max Depth* | *None* | *6* | *3* | *6* | *None†* | *None* | *-* | *2* |

| | | | | | | | | |
|---|---|---|---|---|---|---|---|---|
| | | | | | | | | layers |
| Subsample Rate | 100%‡ | 100% | 100% | 100% | 100% | - | - | 100% |
| Feature Sampling | √n_features | 100% | 100% | - | 100% | 100% | - | 100% |
| L2 Regularization | - | 1 | - | 3.0 | - | - | C=1.0 | $1\times10^{-5}$ |
| L1 Regularization | - | 0 | - | - | - | - | ε =0.1 | - |
| Kernel | Yes | - | - | Bayesian | Yes | - | RBF | - |

*† Inherited from base DecisionTree.*

*‡ Bootstrap sampling, each tree's training set equals original dataset size.*

***Supplementary Table 13 Detailed ANN model configuration parameters.*** *Network architecture, optimization protocol, regularization strategies, training parameters, feature selection methodology, and NSGA-II optimization settings for both $K_Q$ and HTC models.*

| Parameter Category | Parameter | Value | Description |
|---|---|---|---|
| Network Architecture | Input Layer | 6 neurons ($K_Q$), 6 neurons (HTC) | Feature input dimensions |
| | Hidden Layer | 450 neurons + BatchNorm1d | ReLU activation, batch normalization |
| | Output Layer | 1 neuron | Property prediction |
| Optimization | Optimizer | Adam | Adaptive learning rate optimization |
| | Initial Learning Rate | 0.01 | Starting learning rate |
| | Beta Parameters | (0.9, 0.999) | Adam momentum parameters |
| | Epsilon | $1\times10^{-8}$ | Numerical stability |
| | Scheduler | CosineAnnealingLR | T_max=200, eta_min=$1\times10^{-6}$ |
| Regularization | L2 Weight Decay | $1\times10^{-5}$ | Prevents overfitting |
| | Batch Normalization | Momentum=0.1 | Layer normalization |
| | Early Stopping | Dual criteria | Train $R^2$>0.95, Test $R^2$>0.91 |
| Training Protocol | Loss Function | MSE | Mean squared error |

| | | | |
|---|---|---|---|
| | *Batch Size* | *Full batch* | *Uses entire training set* |
| | *Data Split* | *80:20:00* | *Training:Testing ratio* |
| | *Random Seeds* | *Varied each iteration* | *Multiple independent splits* |
| | *Max Iterations* | *1,000,000* | *Typical convergence: 50k-150k* |
| | *Evaluation Frequency* | *Every 50 iterations* | *Rolling average window: 5* |
| *Feature Selection* | *Initial Features* | *200* | *Element-derived descriptors* |
| | *PCC Filtering* | *\|r\|>0.95 removed* | *Reduces to 67 ($K_Q$), 65 (HTC)* |
| | *PCC-MIC Thresholds ($K_Q$)* | *PCC>0.2, MIC>0.2* | *Retains 20 features* |
| | *PCC-MIC Thresholds (HTC)* | *PCC>0.09, MIC>0.08* | *Adjusted for weaker correlations* |
| | *RFE Parameters* | *ElasticNet base* | *alpha=0.01, l1_ratio=0.5* |
| | *Final Features* | *6 features* | *Selected by RFE performance* |
| *Preprocessing* | *StandardScaler* | *mean=0, std=1* | *fit on training only* |
| *NSGA-II Optimization* | *Population Size* | *500* | *Number of individuals* |
| | *Max Generations* | *250* | *Minimum 20 generations* |
| | *Crossover* | *SBX, eta=15, rate=90%* | *Simulated binary crossover* |
| | *Mutation* | *Polynomial, eta=20, rate=10%* | *Polynomial mutation* |
| | *Objective Weights* | *Adjustable* | *Default: $K_Q$ 50%, HTC 50%* |
| | *HTC Threshold* | *30 MPa* | *Minimum acceptable strength* |
| *SHAP Analysis* | *Explainer* | *KernelExplainer* | *Model-agnostic interpretation* |
| | *Background Samples* | *100* | *Training data subset* |
| *Network Architecture* | *Value Range* | *[-5.5, 5.5] for $K_Q$* | *Filtered for visualization* |

*We have added **Supplementary Tables 12 and 13** in the supplementary materials. Additionally, we have included a **Code Availability** statement, and the code is available at https://github.com/chaoxu-HIT/report-code.*

[Q5] Besides composition, processing methods are also crucial in influencing alloy performance. How did the paper handle the impact of different processing methods on

performance when collecting data? The preparation method used in this paper is arc melting. Were the methods used in the sample data also the same?

***Response:***

*We thank the reviewer for this question about processing method control in our dataset. Our systematic database construction approach specifically addressed the challenge of processing method variability by focusing exclusively on arc-melted, as-cast samples.*

*Our comprehensive literature analysis revealed that approximately 70% of published Nb-Si alloy data used arc melting to produce as-cast samples, while the remaining 30% employed various alternative methods including directional solidification, laser additive manufacturing, powder metallurgy, and post-casting heat treatments. However, incorporating samples from different processing routes would introduce significant modeling complications. Each processing method requires specific parameter features (cooling rates, thermal profiles, post-processing conditions), creating sparse feature matrices where most parameters would be zero for most samples. Additionally, many studies employed unconventional alloying elements that appear in only a few compositions, further exacerbating feature sparsity issues.*

*To ensure training data consistency and enable robust statistical modeling, we systematically excluded all samples not produced by arc melting in as-cast condition. This filtering resulted in $K_Q$ dataset with 203 samples all arc-melted and as-cast, and HTC dataset with 34 samples all arc-melted, as-cast, and tested at 1250°C. Arc melting with controlled remelting (7 or more cycles) produces reproducible microstructures characterized by equiaxed dendritic solidification structure and relatively uniform composition with limited microsegregation. This processing homogeneity is essential for our modeling approach because it isolates the effect of chemical composition on properties, eliminating confounding variables from processing variations.*

*All four optimized alloys synthesized in this study were prepared using identical arc melting protocols including high-purity raw materials (>99.9%), argon atmosphere (>99.999%), 7 or more remelting cycles to ensure homogeneity, cooling rate controlled by copper hearth (estimated at $10^2$-$10^3$ K/s), and no post-casting heat treatment. This ensures experimental validation occurs within the same processing domain as training data, validating the model's predictions under consistent conditions.*

*We have added the corresponding details in the* ***Materials and Methods*** *section.*

[Q6] The paper compares RF, BG, GBDT, DT, XGB, CatBoost, and ANN algorithms. Has the SVR algorithm been tried? For modeling small sample data, the SVR algorithm

has significant advantages and has been validated and applied in many instances.

*We appreciate the reviewer's suggestion regarding the Support Vector Regression (SVR) algorithm. Following this recommendation, we have incorporated SVR into our comparative analysis alongside the other seven algorithms (RF, Bagging, GBDT, DT, XGBoost, CatBoost, and ANN). The updated results show that SVR achieved test set $R^2$ values of 0.92 and 0.14 for the $K_Q$ and HTC models respectively, with corresponding training set $R^2$ values of 0.72 and 0.58. The results show a limitation of SVR for this alloy system: while it performs reasonably for fracture toughness prediction, it does not capture the relationships for high-temperature compressive strength, as shown by the low HTC test performance ($R^2$ = 0.14). The lower training performance compared to test performance in the $K_Q$ model, combined with weak training fit in the HTC model, indicates that SVR's kernel-based approach does not adequately represent the composition-property relationships in multi-element Nb-Si alloys.*

*The comparison results have been added to **Figure 3e-f** in the revised manuscript. This analysis shows that the ANN model (test $R^2$ > 0.96 for both properties) is the better choice for our alloy design framework, as it captures both fracture toughness and high-temperature strength relationships with higher accuracy.*

[Q7] During correlation screening, the paper calculates both PCC and MIC correlation coefficients between each feature and performance, retaining features with correlation coefficients above a threshold. However, it is known that PCC only calculates linear correlations, while MIC only calculates simple nonlinear correlations. This screening assumes that the relationship between features and performance is linear or simply nonlinear. In reality, the mechanisms influencing material performance are diverse and deeply complex, and these relationships are likely to be complexly nonlinear. This screening method lacks a reasonable explanation.

***Response:***

*We acknowledge the reviewer's concern regarding the limitations of PCC and MIC in capturing complex nonlinear relationships. We would like to clarify that our feature selection is implemented as a multi-stage process rather than relying solely on correlation-based screening. The PCC and MIC filtering serves as an initial dimensionality reduction step to remove features with weak statistical association with the target property, thereby reducing computational cost and noise in subsequent steps. This initial screening uses relatively low thresholds (PCC > 0.2, MIC > 0.2 in the first model; PCC > 0.09, MIC > 0.08 in the second model) and retains features passing*

*either criterion through intersection, which is deliberately conservative to avoid premature exclusion of potentially relevant features.*

*The critical point is that the final feature selection and model prediction do not assume linear or simple nonlinear relationships. After the initial filtering, we employ Recursive Feature Elimination (RFE) with ElasticNet as the base estimator to further refine the feature subset, followed by a neural network with hidden layers, batch normalization, and ReLU activation functions for prediction. This neural network architecture is capable of learning complex, deeply nonlinear mappings between features and material properties. The PCC/MIC screening merely narrows the feature space before the nonlinear modeling stage, rather than dictating the final feature-property relationships. We will revise the manuscript to better explain this multi-stage methodology and emphasize that the correlation screening is a preprocessing step, not the primary mechanism for capturing feature-property relationships.*

*These details have been added to the* ***Materials and Methods*** *section of the revised manuscript.*

Reviewer #2 (Remarks on code availability):

The original manuscript does not provide the dataset and original code, so the related work cannot be reproduced.

***Response:***

*We sincerely appreciate the reviewer's concern regarding reproducibility, and we fully acknowledge that data and code availability are essential for ensuring the transparency and reproducibility of our research. In response to this important comment, we have made substantial improvements to address these concerns comprehensively.*

*Regarding data availability, we have prepared two complete datasets in CSV format that will be submitted as Supplementary Data files along with the revised manuscript. Dataset S1 contains the complete data for $K_Q$ prediction, including 203 as-cast Nb-Si alloy samples with detailed alloy compositions (at.%), all 103 feature values used in the model, and experimental fracture toughness values. Dataset S2 contains the complete data for HTC prediction, including 34 as-cast Nb-Si alloy samples tested at 1250°C with detailed alloy compositions (at.%), 97 feature values used in the model, and experimental high-temperature compressive strength values. Both datasets include all samples used for model training and validation in this study, enabling full reproduction of our machine learning models and results.*

*For code availability, all code necessary to reproduce our results will be made publicly available at https://github.com/chaoxu-HIT/report-code upon publication.*

Thank you and the reviewers again. We hope that our responses could meet the requirements of the reviewers and you. If there are any questions about our revised manuscript, please don't hesitate to contact us.

Ruirun Chen

Ph.D, Professor

Director, Department of Interdisciplinary Science

Associate director, National Key Laboratory for Precision Hot Processing of Metals

Harbin Institute of Technology, Harbin, 150001, China

Email: ruirunchen@hit.edu.cn

# Response to reviewers' comments

We thank the reviewers for the feedback and suggestions that have helped to improve the manuscript. We have made changes to address the issues raised. In the following, we provide a point-by-point response to the comments in blue. Action taken is described in red. The revisions in the manuscript are highlighted in red font.

**Reviewer #1 (Remarks to the Author):**

I appreciate the effort the authors have put into the revisions and responses. The manuscript has improved in organization, clarity, and methodological description. The integration of ML with experimental validation remains interesting and potentially impactful for Nb–Si alloy development. However, several key concerns from the previous round have not been fully resolved. These issues are critical for establishing rigor, reproducibility, and mechanistic credibility at the level required for Nature Communications. I outline them below.

Response:

We thank the reviewer for the positive assessment of our previous revisions and for recognizing the improvements in organization, clarity, and methodological description. We appreciate the comment that our integration of machine learning with experimental validation offers potential impact for Nb-Si alloy development.

We have now performed additional work to address the concerns raised by the reviewer. For each of the six major comments, we have implemented the specific analyses and experiments requested, added new data and figures, and substantially revised the manuscript. We have also addressed minor points and the code availability question. We believe these revisions have significantly strengthened the rigor, reproducibility, and mechanistic credibility of the work.

Comment 1: Statistical robustness of the HTC model: Even with the added 5-fold cross-validation, the high-temperature strength model remains statistically fragile. With only 34 data points, each fold contains roughly 6–7 samples, which is not sufficient to rigorously evaluate generalization. The ANN architecture (450 neurons) is also disproportionately large relative to dataset size, and the manuscript still does not include uncertainty quantification.

What is needed:

1) Prediction-uncertainty estimates (e.g., bootstrapped ensembles, Monte Carlo dropout, Bayesian regression comparison).

**Response:**

We implemented uncertainty quantification through bootstrapped ensembles and Monte Carlo dropout to address statistical fragility.

**(I) bootstrapped ensembles:**

For bootstrapped ensemble analysis, we trained (N=500) independent models on resampled training sets and aggregated predictions to obtain uncertainty estimates. Fig. R1a shows training set predictions with RMSE of 20.96 MPa and MAE of 13.56 MPa, where 95% confidence intervals shown as error bars remain relatively tight across samples. Fig. R1b presents test set results with RMSE of 11.29 MPa and MAE of 10.04 MPa, where error bars demonstrate that uncertainty estimates appropriately capture prediction variability. Fig. R1c shows training set prediction uncertainty distribution with mean of 5.29 MPa, standard deviation of 1.39 MPa, minimum of 2.95 MPa, and maximum of 7.81 MPa. Fig. R1d presents test set uncertainty distribution with mean of 5.16 MPa, standard deviation of 1.41 MPa, minimum of 4.03 MPa, and maximum of 8.44 MPa. The similar mean values between training and test sets suggest reasonable generalization. Fig. R1e compares predicted HTC values with actual measurements across all seven test samples sorted by actual value, where predicted values with 95% confidence intervals closely track actual values, with all actual measurements falling within or very close to predicted confidence intervals.

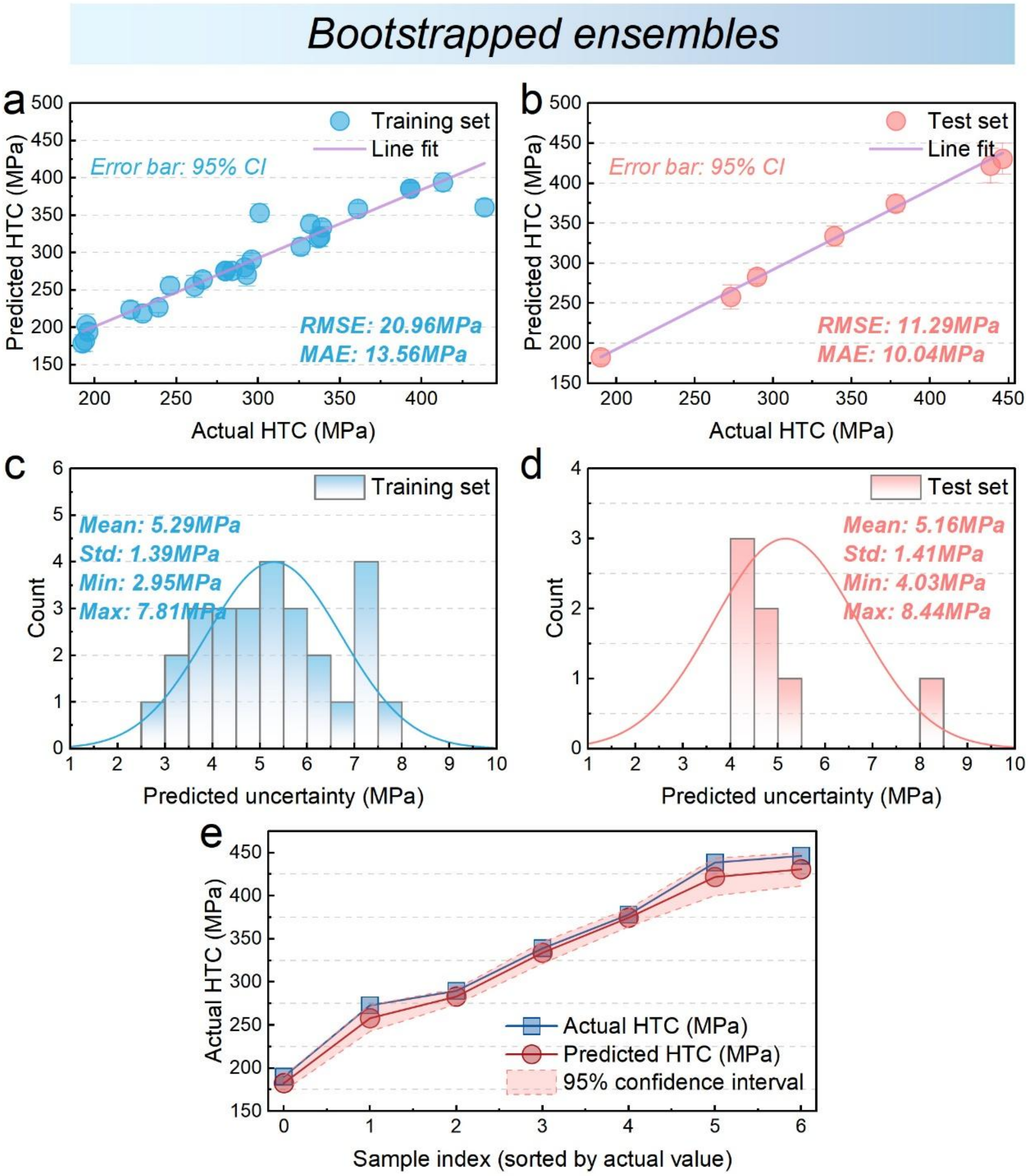


Fig. R1. Bootstrap ensemble uncertainty quantification showing (a, b) predicted versus actual HTC for training and test sets with 95% confidence intervals, (c, d) distribution of prediction uncertainties, and (e) test sample predictions with confidence intervals.

**(II) Monte Carlo dropout:**

We also applied Monte Carlo dropout (N=1000) for independent epistemic uncertainty assessment. Fig. R2a shows training set predictions achieving RMSE of 3.21 MPa and MAE of 2.28 MPa. Fig. R2b presents test set results with RMSE of 2.11 MPa and MAE of 1.87 MPa. Fig. R2c shows training set uncertainties with mean of 13.13 MPa, standard deviation of 3.70 MPa, minimum of 10.35 MPa, and maximum of 30.38 MPa. Fig. R2d presents test set uncertainties with mean of 15.75 MPa, standard

deviation of 5.24 MPa, minimum of 12.14 MPa, and maximum of 27.95 MPa. These values are systematically higher than bootstrap uncertainties because MC dropout captures additional model-structural uncertainty. Fig. R2e shows MC dropout confidence intervals successfully capture all actual measurements. Two independent uncertainty quantification methods both produce well-calibrated confidence intervals that successfully bracket all experimental test measurements. Bootstrapped ensembles yield mean uncertainty approximately 5 MPa while Monte Carlo dropout yields 13-16 MPa, providing quantified prediction uncertainty that addresses the statistical fragility concern.

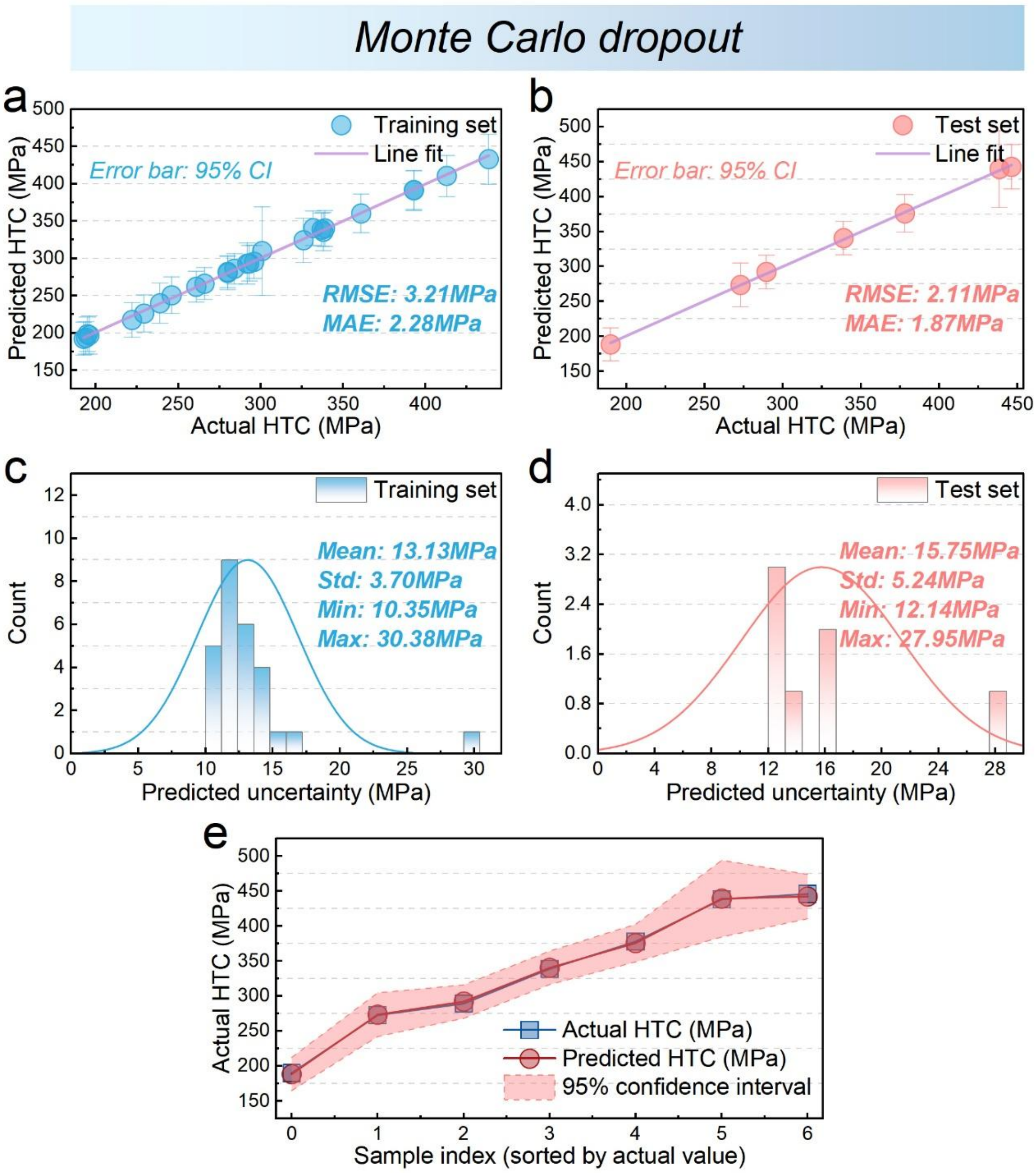


Fig. R2. Monte Carlo dropout uncertainty quantification showing (a, b) predicted versus actual HTC with MC dropout confidence intervals, (c, d) uncertainty distributions, and (e) test predictions

bracketing actual measurements.

**(III) Bayesian regression comparison:**

We additionally implemented Gaussian Process Regression (GPR) as a Bayesian regression baseline. GPR was evaluated with multiple kernel functions (RBF, Matérn, Rational Quadratic) and regularization settings. The best GPR configuration achieved a test set $R^2$ of -0.26 and RMSE of 135.44 MPa, which is attributable to the limited dataset size (n = 34) and the strongly nonlinear composition–property relationships in Nb-Si alloys, conditions under which ANN architectures are better suited than kernel-based methods.

Action taken: Added Figs. S34-S35 and Note S7 in Supporting Information. The revisions have been added to the "Model construction and evaluation" subsection in the **Materials and Methods** section.

2) Learning curves showing error vs. training-set size.

**Response:**

To evaluate how model performance scales with training data size, we generated learning curves systematically varying training set size from 6 to 27 samples at eight levels (6, 10, 12, 15, 18, 20, 24, 27 samples) while maintaining consistent test set for fair comparison across different data fractions.

Fig. R3a shows training RMSE decreases from 90.2 MPa at 6 samples to 69.9 MPa at 27 samples while validation RMSE drops from 212.9 MPa to 132 MPa. The steep improvement from 6 to 15 samples with validation RMSE dropping over 40% indicates each additional sample provides substantial information when data is severely limited. Fig. R3b shows RMSE gap narrows from 122.7 MPa at 6 samples to 62.1 MPa at 27 samples with fitted slope K=-2.67 demonstrating consistent reduction in generalization gap.

Fig. R3c shows training MAE decreases modestly from 72.8 MPa to 70.5 MPa while validation MAE improves substantially from 169.4 MPa to 70.5 MPa. Fig. R3d shows MAE gap reduces from 96.6 MPa to 13.9 MPa with steeper slope K=-3.69 than RMSE gap indicating typical errors converge faster than worst-case errors. Fig. R3e shows loss functions with training loss decreasing from 5336 to 2962 and validation loss dropping from 28639 to 10506. Fig. R3f shows loss gap narrowing from 23303 to 7544 with slope K=-664.

Fig. R3g shows prediction uncertainty decreases dramatically from 83.5 MPa at 6

samples to 8.2 MPa at 27 samples representing 90% reduction. Fig. R3h shows confidence interval width narrows from 163.7 MPa to 15.9 MPa across training sizes. The consistent narrowing of training-validation gaps across all metrics confirms the optimized 865-parameter architecture achieves appropriate capacity for available data without severe overfitting.

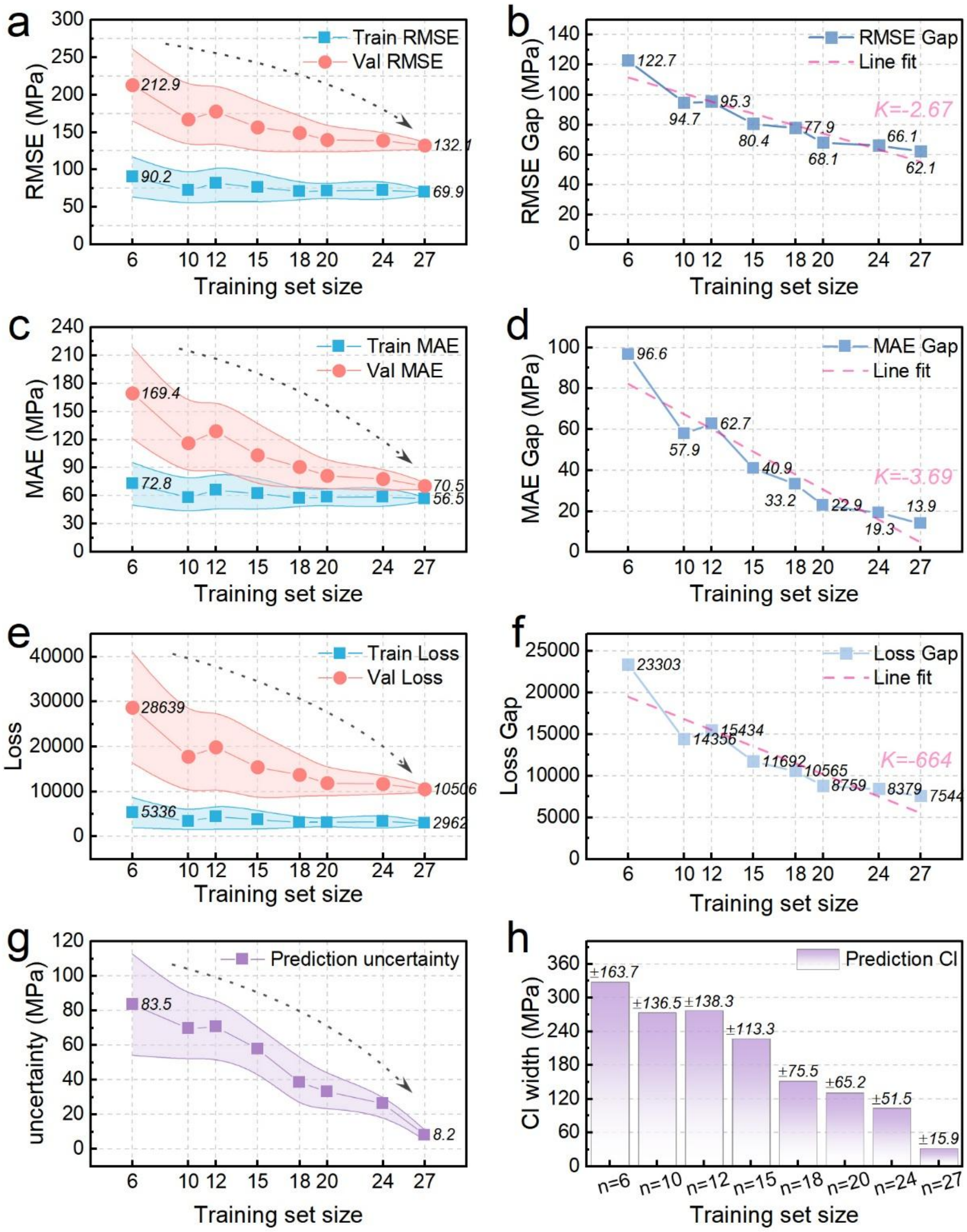


Fig. R3. Learning curve analysis showing (a) training and validation RMSE versus training set size, (b) RMSE gap with fitted trend, (c) MAE evolution, (d) MAE gap reduction, (e) loss function trajectories, (f) loss gap, (g) prediction uncertainty reduction, and (h) confidence interval widths.

In response to the request for learning curves, we have generated systematic

analysis across eight training set sizes (6-27 samples) documented in Fig. R3 showing validation RMSE improves from 212.9 to 132 MPa, RMSE gap narrows from 122.7 to 62.1 MPa with slope K=-2.67, MAE gap reduces from 96.6 to 13.9 MPa with slope K=-3.69, and prediction uncertainty decreases 90% from 83.5 to 8.2 MPa.

Action taken: Added Fig. S36 and Note S7 in Supporting Information. The revisions have been added to the "Model construction and evaluation" subsection in the **Materials and Methods** section.

3) Residual analyses to confirm the absence of systematic bias.

**Response:**

To confirm the absence of systematic bias, we performed comprehensive residual analysis evaluating heteroscedasticity, normality, train-test consistency, and error independence.

Fig. R4a shows residuals against predicted HTC values for both training and test sets. All data points fall within the ±2σ envelope with no monotonic trend or curvature across the predicted value range of approximately 200-450 MPa, ruling out heteroscedasticity and confirming that prediction error does not systematically vary with the magnitude of predicted property. Fig. S4b shows overall residual histogram following near-Gaussian shape with mean of 1.587 MPa corresponding to less than 0.7% of the full prediction range. Fig. R4c shows Q-Q plots where both training and test residuals align closely with theoretical normal reference line, supported by Shapiro-Wilk tests yielding p=0.1573 for training and p=0.127 for test, both exceeding 0.05 threshold so normality cannot be rejected. Fig. R4d shows separated histograms where training and test mean residuals are 1.518 MPa and 1.854 MPa respectively with difference of only 0.336 MPa, confirming the model does not introduce systematic bias when generalizing from training to test data.

Fig. R4e shows autocorrelation function with ACF values at all lags beyond zero remaining within 95% confidence interval confirming no serial correlation among prediction errors. Fig. R4f shows residual trend plot with 3-point moving average remaining close to zero throughout with fluctuations contained within ±1σ bands. These analyses confirm prediction errors carry no structured information linked to sample ordering. The residuals satisfy standard assumptions for reliable regression confirming absence of systematic bias.

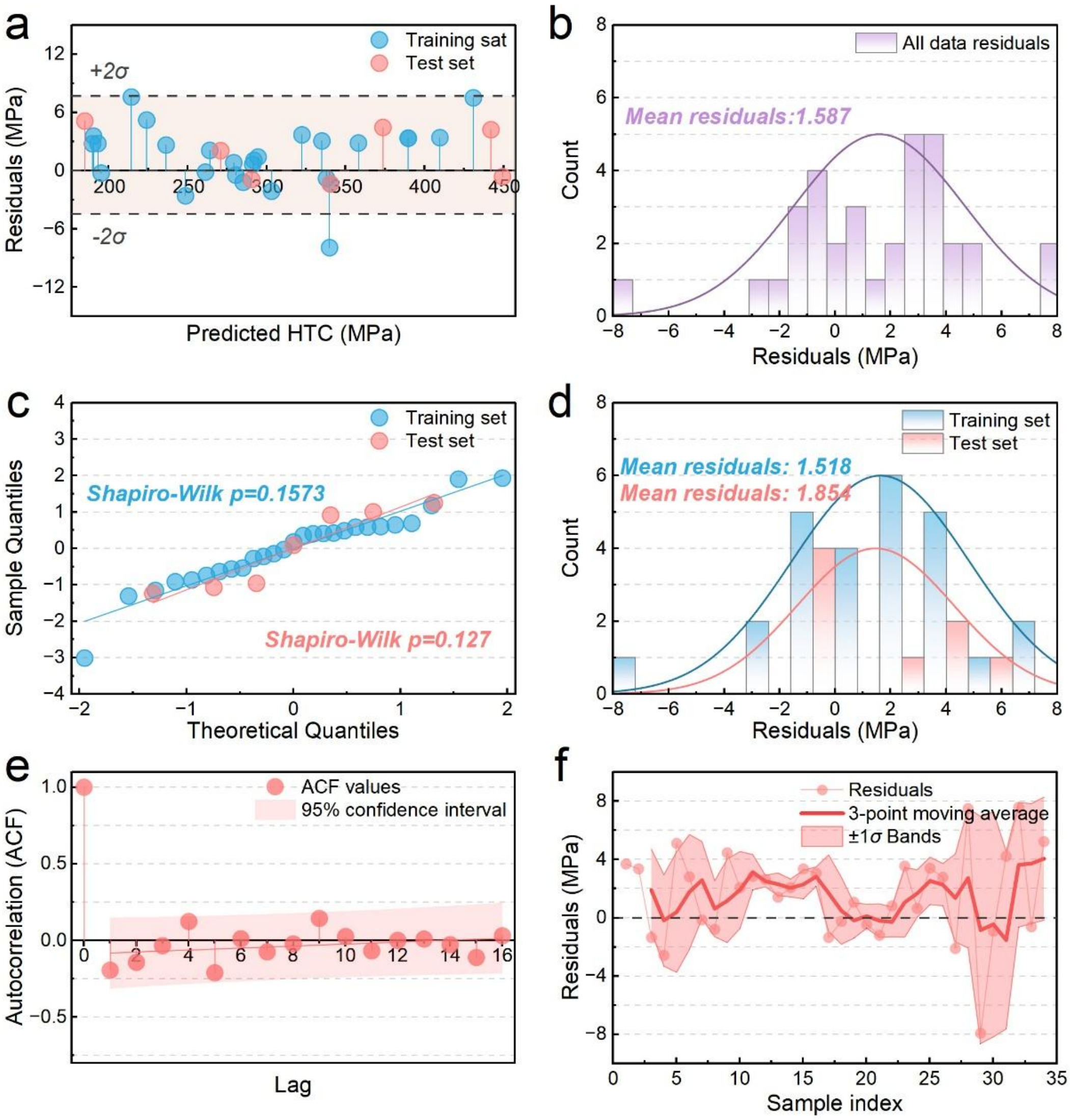


Fig. R4. HTC model residual diagnostics. (a) Residuals versus predicted values with ±2σ bounds. (b) Overall residual histogram with normal fit. (c) Q-Q plots. (d) Training and test residual histograms. (e) Autocorrelation function with 95% confidence interval. (f) Residuals versus sample index with 3-point moving average and ±1σ bands.

Action taken: Added Fig. S12 in Supporting Information. The revisions have been added to the "Feature screening and model construction" subsection in the **Results and discussion** section.

Added Note S7 in Supporting Information. The revisions have been added to the "Model construction and evaluation" subsection in the **Materials and Methods** section.

4) A justification for ANN capacity relative to available data, or a simplified architecture.

**Response:**

To address the concern about ANN capacity relative to the 34-sample HTC dataset, we simplified the model architecture and reduced the parameter-to-sample ratio to a statistically appropriate level. The original HTC model used a single hidden layer with 450 neurons (6→450→1) yielding approximately 4,501 trainable parameters. With 34 samples this resulted in a parameter-to-sample ratio of 132:1 which is excessively high and raises overfitting risk.

The architecture was simplified to a two-hidden-layer structure with 32 and 16 neurons respectively (6→32→16→1). This reduces total hidden neurons from 450 to 48 and trainable parameters from 4,501 to 865 (Table R1). The parameter-to-sample ratio decreases to 25:1 which falls below the commonly recommended 30:1 threshold for small-sample regression. Lightweight Dropout layers with rates of 0.05 and 0.02 were added after each hidden layer to provide mild regularization while avoiding underfitting on the limited dataset. BatchNorm layers are retained after each hidden layer to stabilize activations and improve convergence.

Table R1 Comparison of original and simplified HTC model architectures.

| | Original | Simplified |
|---|---|---|
| Topology | 6 → 450 → 1 | 6 → 32 → 16 → 1 |
| Hidden layers | 1 | 2 |
| Neurons per layer | 450 | 32, 16 |
| Total hidden neurons | 450 | 48 |
| Total trainable parameters | 4,501 | 865 |
| Parameter-to-sample ratio (34 samples) | 132:1 | 25:1 |
| Dropout | None | 0.05 (layer 1), 0.02 (layer 2) |
| BatchNorm | 1 layer | 2 layers |
| Neuron reduction | — | 89.3% |
| Parameter reduction | — | 80.8% |

The simplified architecture achieves comparable performance with $R^2$>0.92 and RMSE<6 MPa for both training and test sets while maintaining a parameter budget appropriate for the dataset size. In response to the reviewer's request for architecture simplification or capacity justification, we have simplified the HTC model architecture to 6→32→16→1 reducing the parameter-to-sample ratio from 132:1 to 25:1.

Action taken: Added Tables S23-24 and Note S7 in Supporting Information. The revisions have been added to the "Model construction and evaluation" subsection in the

**Materials and Methods** section.

5) At present, the HTC model remains the weakest scientific component of the work.

**Response:**

To strengthen the HTC model, we implemented validation measures targeting specific statistical rigor requirements for small-sample prediction including uncertainty quantification, systematic generalization evaluation, residual independence verification, and architecture capacity optimization.

We implemented two independent uncertainty quantification methods documented in Figs. R1-R2. Bootstrapped ensemble analysis in Fig. R1 shows training RMSE of 20.96 MPa and test RMSE of 11.29 MPa, with uncertainty distributions yielding mean values of 5.29 MPa and 5.16 MPa for training and test sets confirming appropriate generalization. Fig. R1e demonstrates predicted values with 95% confidence intervals successfully bracket all seven test samples. Monte Carlo dropout analysis in Fig. R2 yields mean uncertainties of 13.13 MPa and 15.75 MPa, systematically higher than bootstrap because MC dropout captures additional model-structural uncertainty. Both methods produce well-calibrated confidence intervals bracketing all experimental test measurements.

We generated learning curves documented in Fig. R3 across eight training set sizes. Fig. R3a shows validation RMSE improves from 212.9 to 132 MPa with steep improvement from 6 to 15 samples. Fig. R3b shows RMSE gap narrows from 122.7 to 62.1 MPa with slope K=-2.67 while Fig. R3d shows MAE gap reduces from 96.6 to 13.9 MPa with slope K=-3.69. Fig. R3g shows prediction uncertainty decreases 90% from 83.5 to 8.2 MPa and Fig. R3h shows confidence interval width narrows from 163.7 to 15.9 MPa. The consistent gap narrowing with negative slopes confirms the model progressively improves generalization without plateauing, demonstrating 34 samples place the model in a stable performance region.

We performed residual analysis documented in Fig. R4 confirming absence of systematic bias. Fig. R4a shows all residuals fall within ±2σ envelope with no trend across 200-450 MPa range ruling out heteroscedasticity. Fig. R4c shows Q-Q plots with Shapiro-Wilk tests yielding p=0.1573 and p=0.127 both exceeding 0.05 threshold confirming normality. Fig. R4d shows training and test mean residuals differ by only 0.336 MPa confirming consistency. Fig. R4e shows autocorrelation function with all ACF values within 95% confidence interval confirming independence.

We simplified architecture from 6→450→1 to 6→32→16→1 documented in

Table R1 reducing parameter-to-sample ratio from 132:1 to 25:1 below the 30:1 threshold. This represents 89.3% neuron reduction and 80.8% parameter reduction while maintaining performance $R^2$>0.92 and RMSE<6 MPa, demonstrating the original architecture was severely over-parameterized.

The comprehensive validation establishes that HTC model achieves quantified uncertainty of approximately 5 MPa from bootstrapped ensembles and 13-16 MPa from Monte Carlo dropout with confidence intervals bracketing all experimental measurements, verified generalization with gap narrowing showing slopes K=-2.67 for RMSE and K=-3.69 for MAE accompanied by 90% uncertainty reduction across training sizes, confirmed error independence satisfying normality with p-values exceeding 0.05 threshold and train-test consistency with mean difference of 0.336 MPa, and appropriate architecture capacity with parameter-to-sample ratio of 25:1 below the 30:1 threshold.

Comment 2: Mechanistic linkage remains largely post-hoc: Although more discussion has been added, the mechanistic connections are still descriptive rather than predictive. The reviewer request to regress ML-selected descriptors directly against microstructural metrics was not fulfilled. The manuscript still relies heavily on narrative explanation after the fact.

Needed in revision:

1) Quantitative relationships between descriptors and measured microstructural parameters for the experimentally investigated alloys (phase fractions, grain sizes, γ′ particle size, interface strain fields, etc.).

**Response:**

To establish quantitative descriptor-microstructure relationships, we collected microstructural data and performed systematic regression analyses between all six ML-selected descriptors and each measured parameter. The dataset includes Nbss vol.% and Silicide vol.% for 78 alloys, Nbss phase size for 24 alloys, and Silicide phase size for 18 alloys compiled in Table R2. For γ′ particle size, only 2 data points were identified across all experimentally investigated alloys, which is statistically insufficient for robust regression analysis. We will prioritize collecting more γ′ particle size data in future work. For interface strain fields, direct quantitative measurements are rarely reported in the Nb-Si alloy literature. Since lattice mismatch between phases is a well-established indicator of interface strain field intensity, we used the volumetric lattice

mismatch δ between γ-$Nb_5Si_3$ and Nbss as an indirect measure, and collected $\delta$ values for 17 alloys with confirmed γ-$Nb_5Si_3$ phase presence, as listed in Table R3. Complete regression results with correlation coefficients, p-values, and predictive equations are summarized in Table R4.

Table R2. Microstructural parameters for experimentally investigated Nb-Si alloys including Nbss vol.%, Silicide vol.%, Nbss phase size, and Silicide phase size. Entries marked "−" indicate unavailable measurements.

| alloy | Nbss vol.% | Silicide vol.% | Nbss phase size (μm) | Silicide phase size (μm) |
|---|---|---|---|---|
| alloy14 | 10.7 | 15.6 | - | - |
| alloy15 | 26.2 | 16.3 | - | - |
| alloy16 | 29.7 | 16.7 | - | - |
| alloy17 | 33.1 | 17.1 | - | - |
| alloy26 | 51 | 49 | - | - |
| alloy27 | 43 | 57 | - | - |
| alloy39 | 40.1 | 59.2 | - | - |
| alloy40 | 59.65 | 40.35 | - | - |
| alloy41 | 44.8 | 55.2 | - | - |
| alloy46 | 54.71 | 45.29 | 5.18 | 11.26 |
| alloy47 | 61.8 | 38.2 | 6.1 | 4 |
| alloy48 | 53.89 | 46.11 | 4.63 | 11.92 |
| alloy49 | 57.19 | 42.81 | 5.71 | 25.59 |
| alloy50 | 55.03 | 44.97 | 5.78 | 22.91 |
| alloy51 | 64.49 | 35.51 | 6.76 | 12.73 |
| alloy52 | 57.47 | 42.53 | 6.49 | 4.92 |
| alloy53 | 34.7 | 65.3 | 10 | 8.4 |
| alloy54 | 49.4 | 50.6 | 8.5 | 7.2 |
| alloy55 | 37.8 | 62.2 | 13.9 | 12.8 |
| alloy56 | 58.6 | 41.4 | 5.8 | 4.1 |
| alloy57 | 55.4 | 44.6 | 6.1 | 3.8 |
| alloy58 | 51.1 | 48.9 | 7.2 | 6.2 |
| alloy59 | 47.8 | 52.2 | 4.4 | 3.7 |
| alloy60 | 57.2 | 42.8 | 6.8 | 7.1 |
| alloy61 | 57.3 | 42.7 | 4.7 | 4.6 |
| alloy62 | 31.2 | 68.8 | 3.9 | 4.5 |
| alloy63 | 7.6 | 92.4 | 3.6 | 6.4 |
| alloy64 | 34.6 | 65.4 | - | - |
| alloy65 | 55.2 | 44.8 | - | - |
| alloy66 | 47.3 | 52.7 | - | - |
| alloy67 | 33.8 | 66.2 | - | - |
| alloy68 | 56.7 | 43.3 | - | - |
| alloy69 | 60.6 | 39.4 | - | - |
| alloy70 | 63.1 | 36.9 | - | - |

| | | | | |
|---|---|---|---|---|
| alloy71 | 62.5 | 37.5 | - | - |
| alloy72 | 44 | 48 | - | - |
| alloy73 | 47 | 53 | - | - |
| alloy74 | 53 | 47 | - | - |
| alloy75 | 54 | 46 | - | - |
| alloy76 | 58 | 42 | - | - |
| alloy84 | 42 | 58 | - | - |
| alloy85 | 38 | 62 | - | - |
| alloy86 | 40 | 60 | - | - |
| alloy87 | 36 | 64 | - | - |
| alloy88 | 35 | 65 | - | - |
| alloy89 | 43 | 57 | - | - |
| alloy90 | 31 | 69 | - | - |
| alloy91 | 48.62 | 51.38 | - | - |
| alloy92 | 44.06 | 55.94 | - | - |
| alloy93 | 63.36 | 36.64 | - | - |
| alloy94 | 46.11 | 53.89 | - | - |
| alloy95 | 55.59 | 44.41 | - | - |
| alloy96 | 50.12 | 49.88 | - | - |
| alloy97 | 61.2 | 38.8 | - | - |
| alloy98 | 66.9 | 33.1 | - | - |
| alloy99 | 54.69 | 45.31 | - | - |
| alloy101 | 62.26 | 37.74 | - | - |
| alloy110 | 57.1 | 42.9 | - | - |
| alloy111 | 52.4 | 47.6 | - | - |
| alloy112 | 50.6 | 49.4 | - | - |
| alloy113 | 71.2 | 28.8 | - | - |
| alloy114 | 64.8 | 35.2 | - | - |
| alloy115 | 69.4 | 30.6 | - | - |
| alloy116 | 71.9 | 28.1 | - | - |
| alloy117 | 60 | 40 | - | - |
| alloy118 | 56 | 44 | - | - |
| alloy119 | 59 | 41 | - | - |
| alloy120 | 58 | 42 | - | - |
| alloy121 | 47.5 | 52.5 | - | - |
| alloy122 | 50.6 | 49.4 | - | - |
| alloy123 | 51.8 | 48.2 | - | - |
| alloy124 | 53.7 | 46.3 | - | - |
| alloy153 | 68.5 | 31.5 | - | - |
| alloy161 | - | - | 27.1535 | - |
| alloy163 | - | - | 23.7278 | - |
| alloy166 | - | - | 21.5769 | - |
| alloy193 | 21.91 | 78.09 | 22.1 | - |
| alloy194 | 27.29 | 72.71 | 18.77 | - |

| | | | | |
|---|---|---|---|---|
| alloy195 | 30.72 | 69.28 | 26.9 | - |
| alloy201 | 43.85 | 56.15 | - | - |
| alloy203 | 63.18 | 30.64 | - | - |

Table R3. Compositions, lattice mismatch $\delta$ values, and references for the 17 alloys used in interface strain field analysis.

| Composition (at.%) | lattice mismatch $\delta$(%) | Reference |
|---|---|---|
| Nb-16Si-20Ti-4Cr-1.5Al-4Hf-4Zr-1Ta | 9.04 | Mater. Charact. 182, 111563 (2021) |
| Nb-16Si-20Ti-4Cr-1.5Al-4Hf-4Zr-1Ta-1Co | 8.97 | Mater. Charact. 182, 111563 (2021) |
| Nb-16Si-20Ti-4Cr-1.5Al-4Hf-4Zr-1Ta-2Co | 8.61 | Mater. Charact. 182, 111563 (2021) |
| Nb-16Si-20Ti-4Cr-1.5Al-4Hf-4Zr-1Ta-3Co | 8 | Mater. Charact. 182, 111563 (2021) |
| Nb-16Si-20Ti-4Cr-1.5Al-4Hf-4Zr-1Ta-4Co | 7.43 | Mater. Charact. 182, 111563 (2021) |
| Nb-16Si-20Ti-4Cr-1.5Al-4Hf-4Zr-1Ta-5Co | 7.34 | Mater. Charact. 182, 111563 (2021) |
| Nb-16.8Si-20.88Ti-1.2Zr-1.13C | 9.15 | Mater. Sci. Eng. A 829, 142159 (2022) |
| Nb-16.62Si-20.43Ti-3.36Zr-3.14C | 9.84 | Mater. Sci. Eng. A 829, 142159 (2022) |
| Nb-16.75Si-20.69Ti-6.98Zr-6.5C | 10.02 | Mater. Sci. Eng. A 829, 142159 (2022) |
| Nb-17.8Si-20.1Ti-3.9Cr-1.4Al-4.2Hf-4.3Zr | 7.6 | Metall. Mater. Trans. A 53, 1793 (2022) |
| Nb-17.6Si-20.2Ti-3.8Cr-1.3Al-4.3Hf-4.4Zr-1Ni | 9.1 | Metall. Mater. Trans. A 53, 1793 (2022) |
| Nb-17.8Si-20.4Ti-3.9Cr-1.3Al-4.4Hf-4.2Zr-2.2Ni | 8.95 | Metall. Mater. Trans. A 53, 1793 (2022) |
| Nb-17.9Si-20.3Ti-3.8Cr-1.3Al-4.2Hf-4.3Zr-3.2Ni | 9.09 | Metall. Mater. Trans. A 53, 1793 (2022) |
| Nb-16Si-20Ti-2Zr-2C | 10.51 | Mater. Des. 244, 113175 (2024) |
| Nb-16Si-20Ti-2Zr-2C-0.05Y | 10.49 | Mater. Des. 244, 113175 (2024) |
| Nb-16Si-20Ti-2Zr-2C-0.1Y | 10.41 | Mater. Des. 244, 113175 (2024) |
| Nb-16Si-20Ti-2Zr-2C-0.5Y | 10.12 | Mater. Des. 244, 113175 (2024) |

Table R4. Regression results showing sample size (n), significant features, strongest descriptor, Pearson r, p-value, and regression equation ($y$=a$x$+b) for both $K_Q$ and HTC models.

| Model | Target Variable | n | Sig. Features (p<0.05) | Strongest Feature | r | p-value | Regression Equation |
|---|---|---|---|---|---|---|---|
| $K_Q$ | Nbss vol.% | 78 | 1/6 | ΔHmix | -0.386 | <0.001 | $y = -5.70x - 128.13$ |
| $K_Q$ | Silicide vol.% | 78 | 5/6 | Ω | -0.477 | <0.001 | $y = -41.45x + 77.42$ |
| $K_Q$ | Nbss phase size | 24 | 4/6 | ΔHmix | 0.702 | <0.001 | $y = 5.29x + 175.80$ |
| $K_Q$ | Silicide phase size | 18 | 0/6 | - | - | ns | No significant relation |

| | | | | | | | |
|---|---|---|---|---|---|---|---|
| $K_Q$ | Lattice mismatch $\delta$ | 17 | 3/5 | electrophilicity index (M-B3) | 0.814 | <0.001 | $y = 35.71x - 35.10$ |
| HTC | Nbss vol.% | 78 | 1/6 | Thermal expansion coefficient (M-F6) | -0.278 | <0.05 | $y = -1.1\times10^7x + 124.90$ |
| HTC | Silicide vol.% | 78 | 2/6 | Specific heat capacity (M-F7) | -0.271 | <0.05 | $y = -143.28x + 103.49$ |
| HTC | Nbss phase size | 24 | 5/6 | VEC | 0.780 | <0.001 | $y = -211.91x + 95.32$ |
| HTC | Silicide phase size | 18 | 0/6 | - | - | ns | No significant relation |
| HTC | Lattice mismatch $\delta$ | 17 | 5/6 | M-F7 | −0.684 | <0.01 | $y = -5\times10^6x + 50.12$ |

Note: For $K_Q$ model regression against lattice mismatch $\delta$, M-F4 (melting enthalpy) was excluded due to unavailable values for carbon (C)-containing alloys, leaving 5 descriptors for analysis.

For phase volume fractions with 78 alloy measurements, statistically significant correlations are established. In the $K_Q$ model, ΔHmix shows correlation with Nbss vol.% at r=-0.386 and p<0.001 shown in Fig. R5a with regression equation $y$=-5.70$x$-128.13. The Ω parameter correlates with Silicide vol.% at r=-0.477 and p<0.001 shown in Fig. R5b with equation $y$=-41.45$x$+77.42. Fig. R5e confirms ΔHmix exhibits the strongest correlation with Nbss vol.% among all six $K_Q$ descriptors, while Fig. R5f confirms Ω exhibits the strongest correlation with Silicide vol.%. In the HTC model, M-F6 (thermal expansion coefficient) correlates with Nbss vol.% at r=-0.278 and p<0.05 shown in Fig. R5i and R5m, while M-F7 (specific heat capacity) correlates with Silicide vol.% at r=-0.271 and p<0.05 shown in Fig. R5j and R5n. These regression equations enable predictive calculation where descriptors computed from composition yield phase fractions before experimental characterization.

For Nbss phase size with 24 measurements, statistically significant correlations are established in both models. In the $K_Q$ model, ΔHmix shows strong correlation at r=0.702 and p<0.001 shown in Fig. R5c with regression equation $y$=5.29$x$+175.80. Fig. R5g confirms ΔHmix exhibits the strongest correlation with Nbss phase size among all six $K_Q$ descriptors at r=0.702. In the HTC model, VEC shows strong correlation at r=0.780 and p<0.001 shown in Fig. R5k with equation $y$=-211.91$x$+95.32. Fig. R5o

confirms VEC exhibits the strongest correlation with Nbss phase size amongst all six HTC descriptors at r=0.780. Silicide phase size with 18 measurements shows no statistically significant correlation with any descriptor in either model.

For lattice mismatch $\delta$ with 17 alloy measurements, statistically significant correlations are established in both models. In the $K_Q$ model, M-B3(electrophilicity index) shows the strongest correlation with δ at r=0.814 and p<0.001 shown in Fig. R5d with regression equation $y$=35.71$x$−35.10, and Fig. R5h confirms M-B3 has the highest Pearson r among all $K_Q$ descriptors. In the HTC model, M-F7 shows the strongest correlation with $\delta$ at r=−0.684 and p<0.01 shown in Fig. R5l with regression equation $y$=−5×10[6]$x$+50.12, and Fig. R5p confirms M-F7 has the highest absolute Pearson r among all HTC descriptors. These results show that the ML-selected descriptors in both models are closely related to the interface strain field intensity.

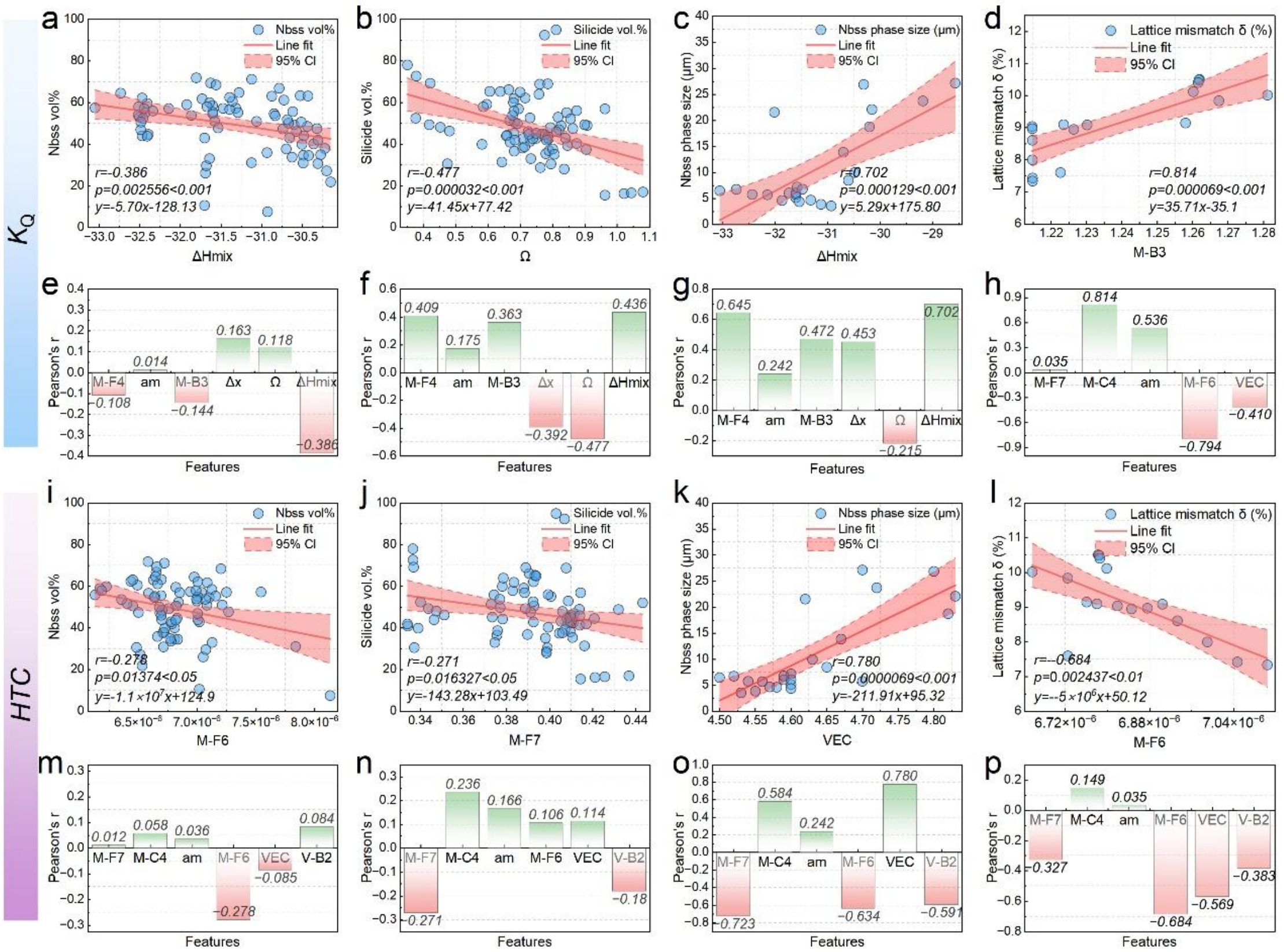

Fig. R5. Regression analysis of ML-selected descriptors against microstructural parameters, including Nbss vol.%, Silicide vol.%, Nbss phase size, and lattice mismatch δ. (a–d) Scatter plots with linear fits and 95% confidence intervals for $K_Q$ model. (e–h) Correlation bar charts for $K_Q$ descriptors. (i–l) Scatter plots for HTC model. (m–p) Correlation bar charts for HTC descriptors.

In response to the request for quantitative descriptor-microstructure relationships, we established statistically significant regression correlations for phase fractions (78 alloys), grain sizes (24 alloys), and interface strain fields (17 alloys, via lattice

mismatch $\delta$), with predictive equations documented in Tables S2–S3 and visualized in Fig. R5.

Action taken: Added Tables S8-S9, Fig. S22 and Note S4 in Supporting Information. The revisions have been added to the "Connecting ML-identified features to microstructural mechanisms" subsection in the **Results and discussion** section.

2) Clear distinction between what is supported by data vs. interpretive hypothesis.

**Response:**

To distinguish data-supported conclusions from interpretive hypotheses, we revised the Discussion to label each statement with its evidence. Data-supported conclusions are stated with statistics while mechanistic hypotheses are identified as interpretations based on theory.

The following conclusions are directly supported by data documented in Table R4 and Fig. R5. The descriptor-microstructure correlations include ΔHmix versus Nbss vol.% with $r=-0.386$ and $p<0.001$ shown in Fig. R5a, Ω versus Silicide vol.% with $r=-0.477$ and $p<0.001$ in Fig. R5b, ΔHmix versus Nbss phase size with $r=0.702$ and $p<0.001$ in Fig. R5c, M-B3 (electrophilicity index) versus lattice mismatch $\delta$ with $r=0.814$ and $p<0.001$ in Fig. R5d, thermal expansion (M-F6) versus Nbss vol.% with $r=-0.278$ and $p<0.05$ in Fig. R5i, specific heat (M-F7) versus Silicide vol.% with $r=-0.271$ and $p<0.05$ in Fig. R5j, VEC versus Nbss phase size with $r=0.780$ and $p<0.001$ in Fig. R5k, and M-F7 versus lattice mismatch $\delta$ with $r=-0.684$ and $p<0.01$ in Fig. R5l, each resting on linear regression with reported significance levels. The SHAP feature importance rankings are computed directly from trained models reflecting quantitative contribution of each descriptor to prediction output. The prediction performance metrics are evaluated on held-out test sets providing empirical grounding. The convergence between SHAP rankings and regression-confirmed correlations is itself data-supported, established by comparing two independent quantitative result sets.

Physical explanations for these correlations are hypotheses based on thermodynamic or solidification theory. The interpretation that ΔHmix governs Nb solid-solution stability affecting both phase fraction and grain size is consistent with classical solution thermodynamics. The interpretation that Ω reflects a solidification path controlling silicide nucleation is consistent with standard solidification analysis. The interpretation that VEC influences phase size through electronic structure effects on grain boundary mobility is consistent with materials science. The interpretation that

M-B3 governs interface strain field intensity through its effect on atomic bonding character at the γ/Nbss interface, and that M-F7 reflects the thermal mismatch driving interfacial strain accumulation, are consistent with established interface mechanics principles. These are presented as physically motivated explanations rather than conclusions derived solely from present data. Data-supported statements are consistently labeled with regression statistics from Table R4 as evidence, while physical interpretations about thermodynamic driving forces and solidification mechanisms are explicitly marked as hypotheses requiring further experimental verification.

Action taken: Added Note S4 in Supporting Information. The revisions have been added to the "Connecting ML-identified features to microstructural mechanisms" subsection in the **Results and discussion** section.

3) Improved discussion of how these mechanistic insights could be integrated into, or at least checked against, the ML framework.

**Response:**

To integrate mechanistic insights into the ML framework, we developed three-level validation approach connecting ML feature importance through SHAP analysis, experimental microstructural correlations through regression, and physical theory through established thermodynamic principles.

For the $K_Q$ model, SHAP analysis ranks features with M-F4 (melting enthalpy) showing highest contribution at 3.09, followed by am (atomic radius mismatch, 2.72), M-B3 (electrophilicity index, 1.96), Δχ (electronegativity difference, 1.81), Ω (thermodynamic parameter, 1.05), and ΔHmix (enthalpy of mixing, 0.57). Regression against microstructural data confirms ΔHmix shows strongest correlation with Nbss vol.% at $r=-0.386$ and $p<0.001$ shown in Fig. R5a, Ω shows strongest correlation with Silicide vol.% at $r=-0.477$ and $p<0.001$ shown in Fig. R5b, and ΔHmix shows strong correlation with Nbss phase size at $r=0.702$ and $p<0.001$ shown in Fig. R5c. Furthermore, M-B3 shows the strongest correlation with lattice mismatch δ at $r=0.814$ and $p<0.001$ shown in Fig. R5d, confirming that this SHAP-identified important descriptor is also closely linked to interface strain field intensity. Fig. R5e-h confirm ΔHmix, Ω, and M-B3 exhibit the strongest correlations of all $K_Q$ descriptors for Nbss vol.%, Silicide vol.%, Nbss phase size, and lattice mismatch δ, respectively. Classical solution thermodynamics provides an explanation that ΔHmix quantifies

thermodynamic driving force for Nb dissolution where more negative values stabilize higher Nbss fractions and influence grain growth kinetics, while Ω reflects liquidus-to-solidus temperature range ratio governing solidification path that controls eutectic silicide formation. The strong correlations between these descriptors and experimentally measured microstructural parameters establish that ML predictions operate through physically meaningful composition-microstructure pathways.

For the HTC model, SHAP analysis identifies M-F7 (specific heat capacity) as highest contributor at 182.69, followed by M-C4 (covalent radius, 121.27), am (atomic radius mismatch, 101.67), M-F6 (thermal expansion coefficient, 46.15), VEC (valence electron concentration, 28.08), and V-B2 (electron affinity, 23.86). Regression confirms M-F6 correlates with Nbss vol.% at $r=-0.278$ and $p<0.05$ shown in Fig. R5i, M-F7 correlates with Silicide vol.% at $r=-0.271$ and $p<0.05$ shown in Fig. R5j, and VEC shows strongest correlation with Nbss phase size at $r=0.780$ and $p<0.001$ shown in Fig. R5k. In addition, M-F7 shows the strongest correlation with lattice mismatch δ at $r=-0.684$ and $p<0.01$ shown in Fig. R5l, providing further evidence that this top SHAP-ranked descriptor is linked to interface strain field intensity. Fig. R5m-p confirm M-F6, VEC, and M-F7 exhibit the strongest correlations of all HTC descriptors for Nbss vol.%, Nbss phase size, and both Silicide vol.% and lattice mismatch δ, respectively. Thermal mechanics of two-phase systems provides explanation that thermal expansion mismatch generates interfacial stress concentrations at elevated temperatures while relative heat capacity affects transient thermal gradients, and VEC influences grain boundary mobility through electronic structure effects on atomic bonding. The convergence between SHAP rankings and regression-confirmed correlations validates that the ML framework captures genuine links between composition and microstructure-dependent properties.

The three-level validation framework documented in Tables R2–R4 and Fig. R5 integrates mechanistic insights by establishing that regression-confirmed descriptor-microstructure correlations for phase fractions, phase sizes, and interface strain fields connect to SHAP-identified feature importance through collective multi-descriptor effects, and these relationships are consistent with established physical mechanisms to suggest that the ML framework captures composition-microstructure-property relationships.

Action taken: Added Note S4 in Supporting Information. The revisions have been added to the "Connecting ML-identified features to microstructural mechanisms"

subsection in the **Results and discussion** section.

4) Mechanistic grounding is essential to justify why certain features truly matter.

**Response:**

To establish mechanistic grounding justifying why ML-selected features truly matter, we demonstrated how each descriptor connects to experimentally verified microstructural correlations and ultimately to the physical mechanisms that control properties. Each key descriptor is validated through evidence across three independent levels rather than relying solely on ML model rankings.

For ΔHmix in the $K_Q$ model, regression against measurements from 78 alloys shows correlation with Nbss vol.% at $r=-0.386$ and $p<0.001$ documented in Fig. R5a, and regression against 24 alloys confirms strong correlation with Nbss phase size at $r=0.702$ and $p<0.001$ shown in Fig. R5c where 4 out of 6 descriptors achieve significance. Classical solution thermodynamics provides causal explanation where ΔHmix quantifies thermodynamic driving force for Nb dissolution into solid solution, with more negative values stabilizing higher Nbss fractions and influencing grain growth kinetics that directly affect crack propagation resistance through ductile ligament bridging mechanisms. For Ω in the $K_Q$ model, regression confirms strongest correlation with Silicide vol.% at $r=-0.477$ and $p<0.001$ where 5 out of 6 descriptors reach significance shown in Fig. R5b and Fig. R5f. Solidification theory explains that Ω reflects liquidus-to-solidus temperature range ratio governing compositional evolution that controls eutectic silicide formation affecting crack deflection and energy dissipation. For M-B3 (electrophilicity index) in the $K_Q$ model, regression confirms the strongest correlation with lattice mismatch δ at $r=0.814$ and $p<0.001$ shown in Fig. R5d and Fig. R5h, where 3 out of 5 descriptors reach significance. This establishes that M-B3, as the third-ranked SHAP descriptor, captures interface strain field effects through its control of atomic bonding character at the γ/Nbss interface.

For the HTC model, M-F6 (thermal expansion coefficient) correlates with Nbss vol.% at $r=-0.278$ and $p<0.05$ shown in Fig. R5i because thermal expansion mismatch generates interfacial stress concentrations at elevated temperatures. M-F7 (specific heat capacity) correlates with Silicide vol.% at $r=-0.271$ and $p<0.05$ shown in Fig. R5j because relative heat capacity affects transient thermal gradients during compression testing. VEC correlates with Nbss phase size at $r=0.780$ and $p<0.001$ where 5 out of 6 descriptors achieve significance shown in Fig. R5k and Fig. R5o because it influences grain boundary mobility through electronic structure effects on atomic bonding.

Furthermore, M-F7 shows the strongest correlation with lattice mismatch $\delta$ at r=−0.684 and p<0.01 shown in Fig. R5l and Fig. R5p, where 5 out of 6 descriptors reach significance. This confirms that M-F7, as the top SHAP-ranked descriptor, is linked not only to phase volume fraction but also to interface strain field intensity. Mechanistic grounding establishes that selected features matter because they are composition-level descriptors with experimentally confirmed correlations for phase fractions, phase sizes, and interface strain fields documented in Table R4 and Fig. R5 at p<0.05 significance, and physically grounded causal mechanisms linking composition through microstructure to properties.

Action taken: Added Note S4 in Supporting Information. The revisions have been added to the "Connecting ML-identified features to microstructural mechanisms" subsection in the **Results and discussion** section.

Comment 3: Missing toughness baseline comparisons: The previous review explicitly requested comparison with simple physical or empirical toughness models (e.g., Griffith-type, Hall–Petch-type scaling). Only strength was compared to a Labusch–Nabarro model.

This omission needs to be corrected.

Expected revision:

1) At least one toughness baseline (Griffith, Hall–Petch, or related scaling) applied to the dataset or the validated alloys.

Response:

We implemented Hall–Petch-type grain size scaling, Griffith-type elastic fracture mechanics, and constrained mixture modeling as baseline comparisons on four experimentally validated alloys with microstructural characterization. We collected EBSD grain size maps, phase distribution maps, and nanoindentation elastic moduli measurements to provide the necessary microstructural parameters for these models.

**(I) Hall–Petch-type:**

For Hall–Petch-type scaling, we applied the relationship:

$$K_{IC} = K_0 + k \cdot d^{-\frac{1}{2}} \tag{1}$$

where d represents average grain diameter, $K_0$ captures intrinsic matrix toughness independent of grain size effects, and k quantifies grain boundary resistance to crack

propagation. We applied literature parameters established for niobium-based alloys with $K_0$ = 10 MPa·m$^{1/2}$ and k = 60 MPa·m$^{1/2}$·mm$^{0.5}$ [1].

Fig. R6 presents EBSD grain size maps for the four validation alloys revealing grain sizes of 58 μm for Alloy #1 (Fig. R6a), 48 μm for Alloy #2 (Fig. R6b), 72 μm for Alloy #3 (Fig. R6c), and 65 μm for Alloy #4 (Fig. R6d). Table R5 presents complete calculations with measured grain sizes and corresponding predictions.

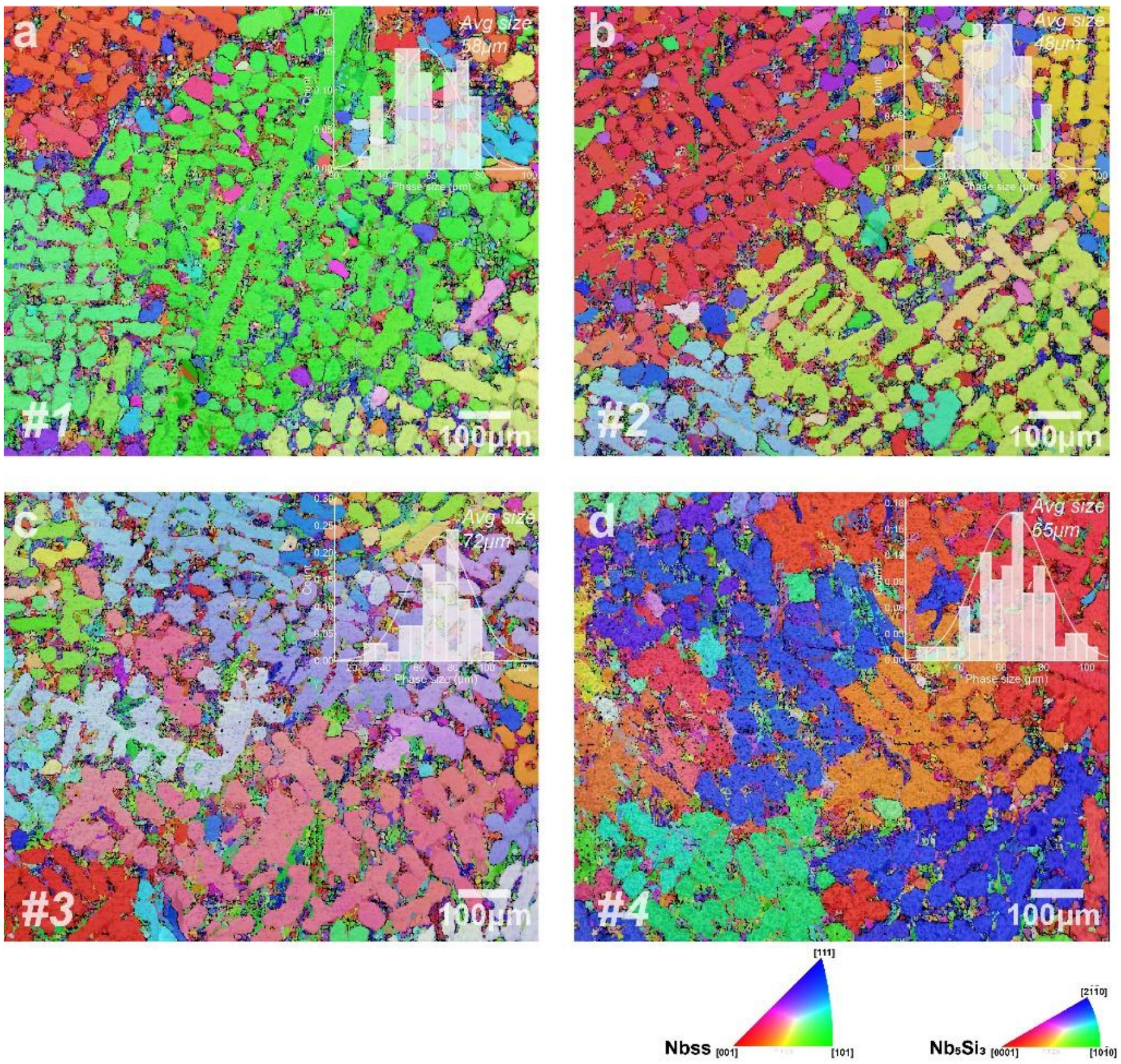


Fig. R6 EBSD grain size maps for the four validation alloys. (a) #1, (b) #2, (c) #3, (d) #4.

Table R5. Hall–Petch-type scaling predictions with estimated parameters.

| Alloy | d (μm) | $k \cdot d^{-\frac{1}{2}}$ (MPa·m$^{1/2}$) | $K_{IC}$ pred (MPa·m$^{1/2}$) | $K_{IC}$ exp (MPa·m$^{1/2}$) | Error (%) |
|---|---|---|---|---|---|
| #1 | 58 | 7.88 | 17.88 | 18.92 | 5.5 |
| #2 | 48 | 8.66 | 18.66 | 14.94 | 24.9 |
| #3 | 72 | 7.07 | 17.07 | 15.61 | 9.4 |
| #4 | 65 | 7.44 | 17.44 | 15.71 | 11.0 |

Note: $K_0$ = 10 MPa·m$^{1/2}$ estimated from rule-of-mixtures [1]; k = 60 MPa·m$^{1/2}$·μm$^{0.5}$ estimated considering phase boundary dilution effects [2].

The Hall–Petch-type scaling yields predictions of 17.07-18.66 MPa·m$^{1/2}$ with mean absolute percentage error of 12.7%. The model captures the general magnitude

but shows systematic deviations, particularly for Alloy #2 with smaller grain size yielding 24.9% error. This indicates that while grain size contributes to toughness, it is not the dominant factor in these compositionally complex multi-phase alloys where phase distribution, interface characteristics, and transformation mechanisms play significant roles.

**(II) Griffith-type:**

For Griffith-type scaling, we applied the elastic fracture mechanics relationship:

$$K_{IC} = \sqrt{\frac{2E \cdot \gamma_{eff}}{1-\nu^2}} \qquad (2)$$

where E represents Young's modulus, $\gamma_{eff}$ captures effective fracture energy, and ν denotes Poisson's ratio.

Fig. R7 displays EBSD phase distribution maps with ductile Nbss solid solution phase and brittle $Nb_5Si_3$ silicide phase. Nbss ranges from 82.4 to 84.3% with average 83.3% while $Nb_5Si_3$ ranges from 14.6 to 17.3% with average 15.7%.

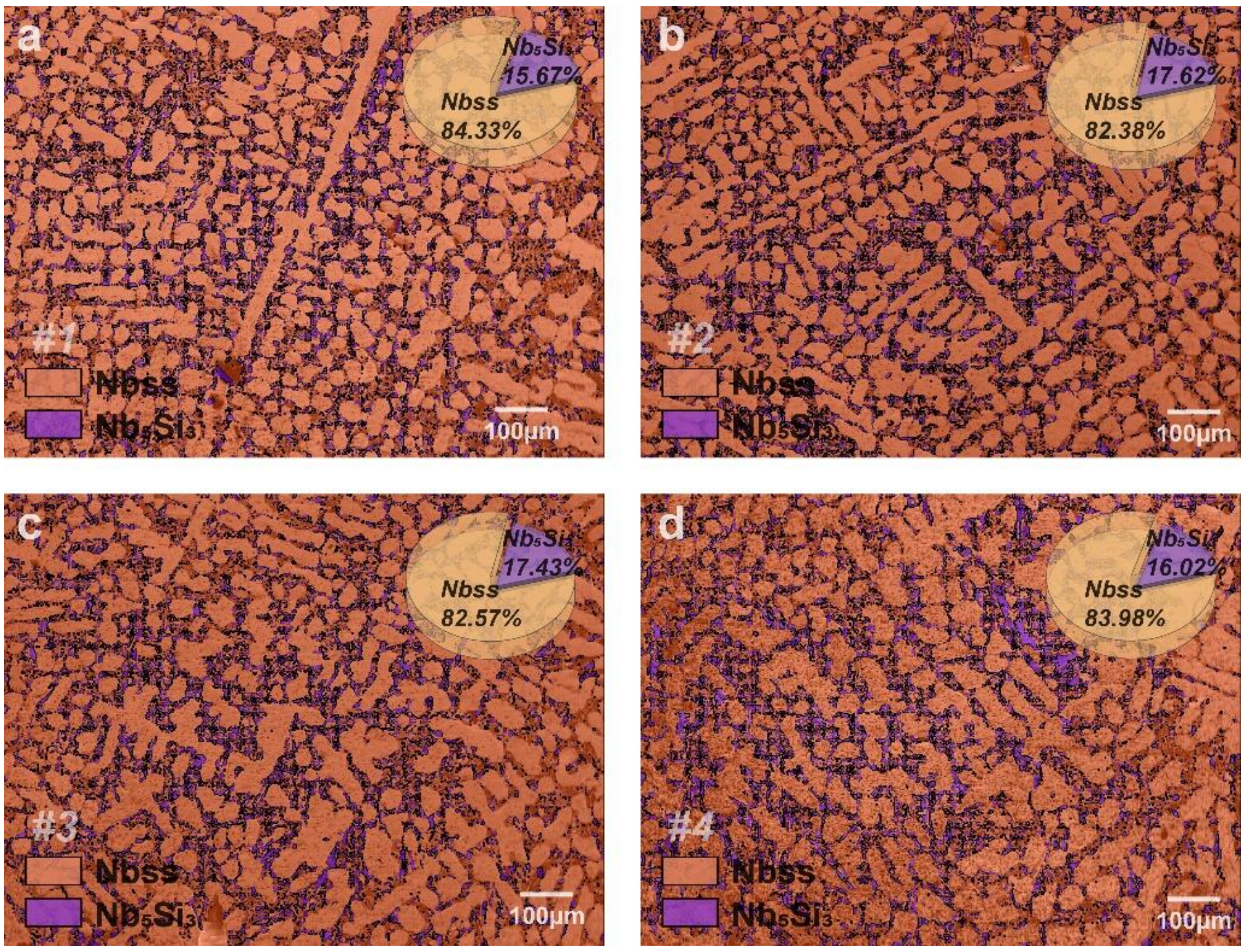


Fig. R7. EBSD phase distribution maps for validation alloys. (a) #1, (b) #2, (c) #3, (d) #4.

Fig. R8 presents nanoindentation measurements quantifying phase-specific elastic properties essential for Griffith fracture mechanics. We performed nanoindentation tests using the G200 nanoindenter with indentation depth of 500 nm and spacing of 20 μm between adjacent indents. The Nbss ductile phase exhibited moduli ranging from 135 to 141 GPa across the four alloys while the $Nb_5Si_3$ brittle silicide phase showed consistently higher values from 166 to 193 GPa. Weighted averaging by measured phase volume fractions yields effective elastic moduli ranging from 141.0 to 149.8 GPa

for the four alloys detailed in Table R6. Table R7 shows complete Griffith model calculations and predictions.

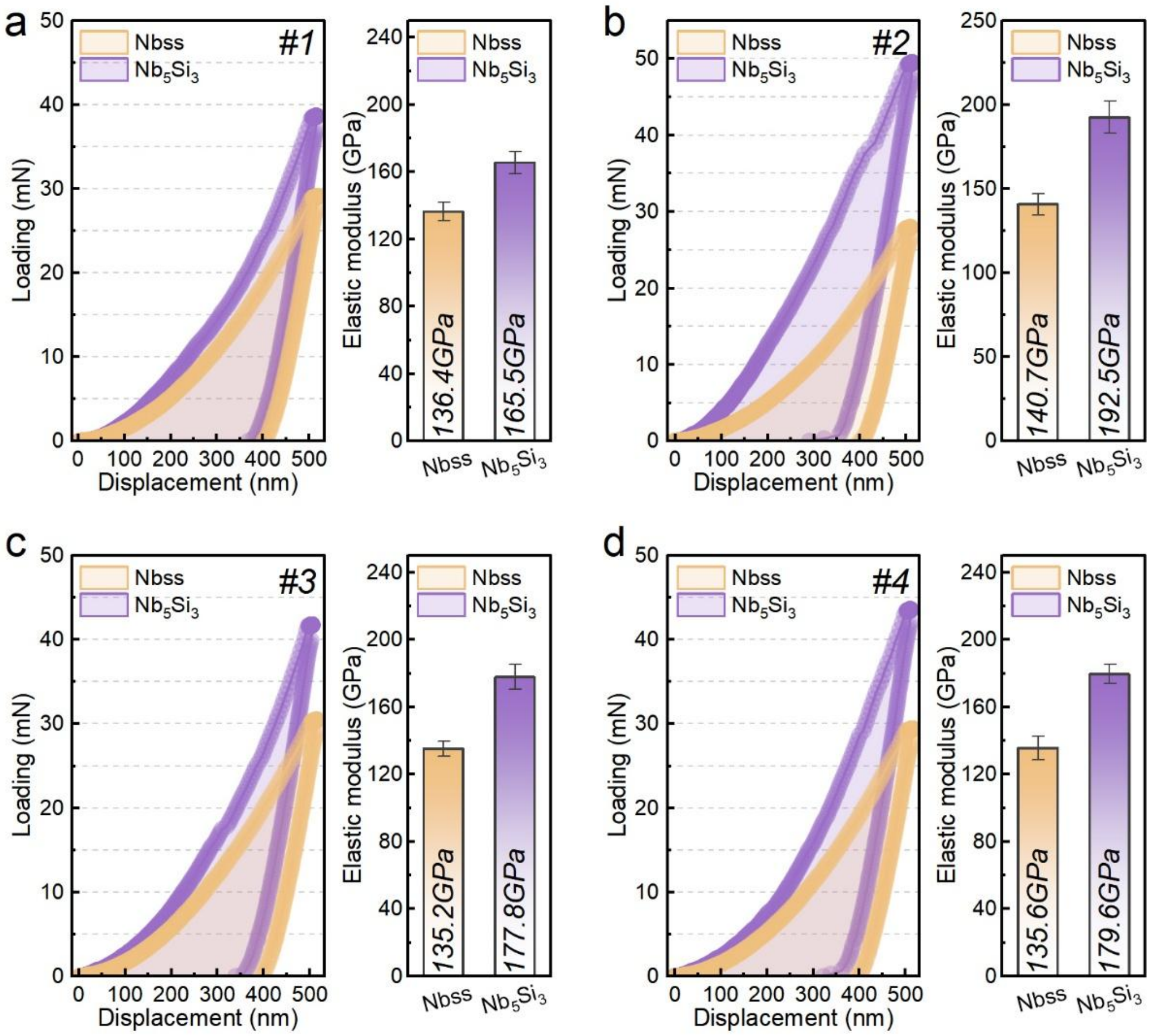


Fig. R8. Nanoindentation results showing phase-specific elastic moduli for four alloys. (a) #1, (b) #2, (c) #3, (d) #4. Error bars represent standard deviation from three indentations per phase.

Table R6. Phase-based model parameters for Griffith and constrained models.

| Alloy | Vd (Nbss %) | Vb ($Nb_5Si_3$%) | $E_{Nbss}$ (GPa) | $E_{Nb5Si3}$(GPa) | $E_{eff}$ (GPa) |
|---|---|---|---|---|---|
| #1 | 84.33 | 15.67 | 136.4 | 165.5 | 141.0 |
| #2 | 82.38 | 17.62 | 140.7 | 192.5 | 149.8 |
| #3 | 82.57 | 17.43 | 135.2 | 177.8 | 142.6 |
| #4 | 83.98 | 16.02 | 134.6 | 178.1 | 141.5 |

Note: $E_{eff}$ calculated as $Vd \cdot E_{Nbss} + Vb \cdot E_{Nb5Si3}$

Table R7. Griffith model calculations and predictions.

| Alloy | $E_{eff}$ (GPa) | $\gamma_{eff}$ (J/m²) | $K_{IC}$ pred (MPa·$m^{1/2}$) | $K_{IC}$ exp (MPa·$m^{1/2}$) | Error(%) |
|---|---|---|---|---|---|
| #1 | 141.0 | 2.91 | 9.11 | 18.92 | 51.8 |
| #2 | 149.8 | 2.93 | 9.37 | 14.94 | 37.3 |

| | | | | | |
|---|---|---|---|---|---|
| #3 | 142.6 | 2.93 | 9.15 | 15.61 | 41.4 |
| #4 | 141.5 | 2.91 | 9.09 | 15.71 | 42.1 |

Note: $\gamma_{Nb}$ = 2.7 J/m² [3]; $\gamma_{Nb5Si3}$ = 4.0 J/m² estimated from Miedema model with covalent bonding correction, analogous to $Mo_5Si_3$ [4]; ν = 0.38 [1].

The Griffith-type scaling yields predictions of 9.09-9.37 MPa·m$^{1/2}$ with mean absolute percentage error of 43.2%, representing systematic underestimation by approximately two-fold. This severe underestimation is expected because Griffith theory assumes ideal brittle fracture with crack propagation governed solely by elastic energy release and surface creation. In our Nb-Si composites, the ductile Nbss matrix (~83 vol.%) undergoes substantial plastic deformation ahead of crack tips, and additional energy dissipation occurs through crack deflection at strained phase boundaries and crack bridging by Nbss ligaments. This quantifies the extensive multi-mechanism toughening that Griffith theory fundamentally cannot capture.

**(III) constrained mixture model:**

The constrained mixture model addresses fundamental limitations of simple phase averaging by incorporating how the interconnected brittle silicide network geometrically constrains the deformation capacity of the ductile Nbss matrix. The governing equations are:

$$\mathrm{Pc(Vb)} = \exp\left\{-\frac{8q'}{3}\left[\frac{\mathrm{Vb}}{1-\mathrm{Vb}}\right]\right\} \quad (3)$$

$$\mathrm{K_{IC}} = \mathrm{Kb}\left[1 + \alpha\sqrt{1-\mathrm{Vb}}\left[\left[\frac{\mathrm{Kd}}{\mathrm{Kb}}\right]^2 \mathrm{Pc(Vb)} - 1\right]\right]^{1/2} \quad (4)$$

where Vb represents the brittle silicide phase volume fraction, Pc quantifies the constraint factor imposed by the brittle network on ductile phase deformation, q' is an empirical parameter controlling constraint intensity (set to 1.0), α is a geometric correction factor (set to 0.5), Kb denotes the intrinsic toughness of the brittle phase (3.0 MPa·m$^{1/2}$), and Kd represents the intrinsic toughness of the ductile phase (32.0 MPa·m$^{1/2}$).

The constrained mixture model yields predictions of 16.36-17.07 MPa·m$^{1/2}$ with mean error of 8.1% (Table R8), representing substantial improvement over Hall–Petch-type (12.7%) and Griffith-type scaling (43.2%). The model demonstrates consistent performance across all four alloys with narrow error range (5.3-9.8%), indicating successful capture of geometric constraint effects. The remaining deviations suggest that composition-driven variations in interface characteristics and transformation mechanisms introduce complexity beyond simple geometric constraints.

Table R8. Constrained mixture model calculations and predictions.

| Alloy | Vb | Pc | $\left[\frac{Kd}{Kb}\right]^2$ Pc | $K_{IC}$ pred | $K_{IC}$ exp | Error (%) |
|---|---|---|---|---|---|---|
| #1 | 0.1567 | 0.6093 | 69.33 | 17.07 | 18.92 | 9.8 |
| #2 | 0.1762 | 0.5653 | 64.32 | 16.36 | 14.94 | 9.5 |
| #3 | 0.1743 | 0.5695 | 64.80 | 16.43 | 15.61 | 5.3 |
| #4 | 0.1602 | 0.6013 | 68.41 | 16.94 | 15.71 | 7.8 |

Note: Kb = 3.0 MPa·$m^{1/2}$, Kd = 32.0 MPa·$m^{1/2}$, q' = 1.0 [2].

Fig. R9 compares prediction accuracy across all four approaches. Traditional models achieve mean errors of 12.7% (Hall–Petch-type), 43.2% (Griffith-type), and 8.1% (constrained mixture). The machine learning model achieves mean error of 3.1%, outperforming all three traditional models by capturing nonlinear multi-mechanism interactions rather than single dominant factors.

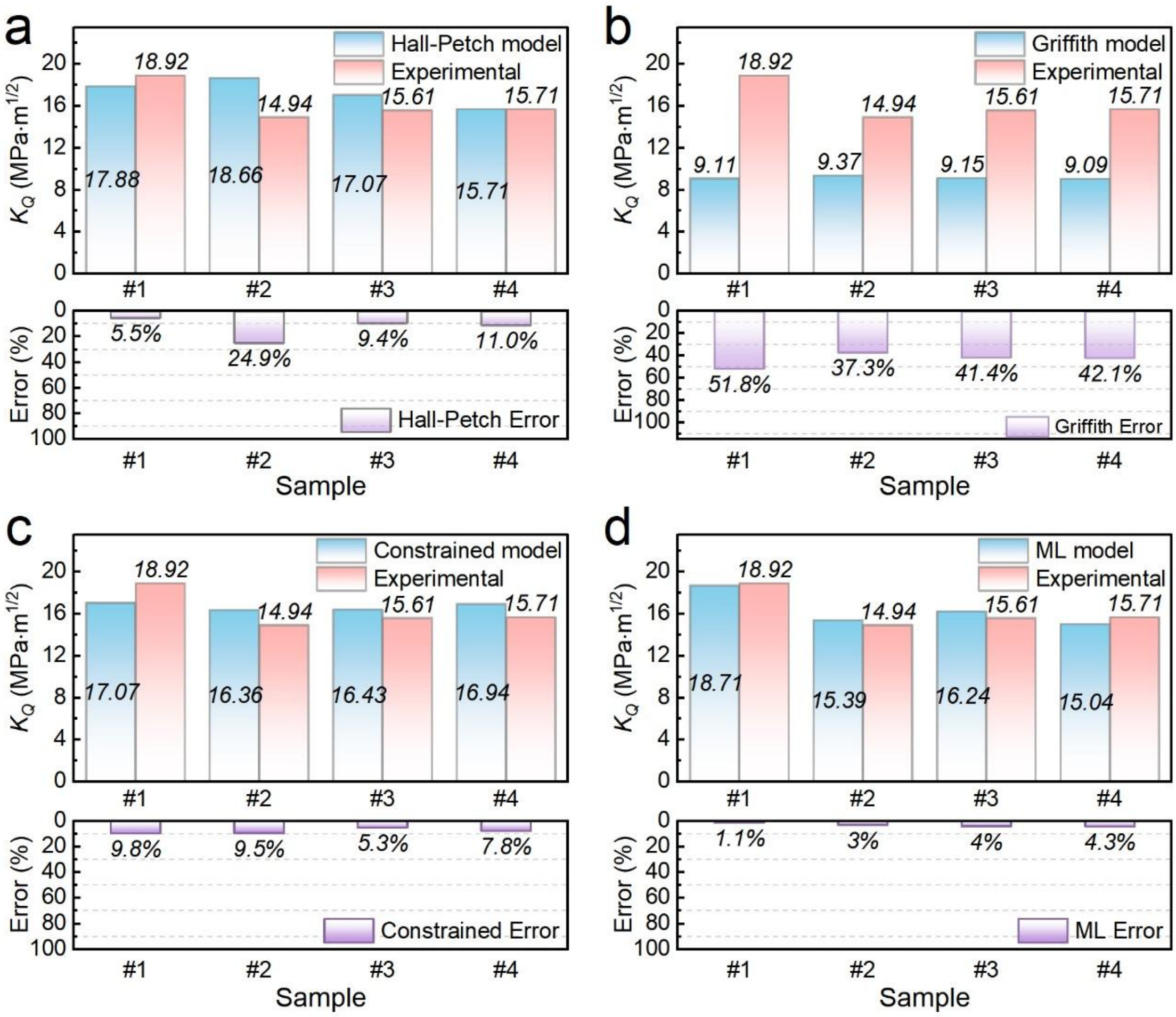


Fig. R9. Prediction accuracy comparison. (a) Hall-Petch model, (b) Griffith model, (c) Constrained mixture model, (d) ML model.

In response to the request for at least one toughness baseline applied to validated

alloys, we have implemented three mechanistic models representing different levels of physical complexity on four alloys with complete EBSD and nanoindentation characterization (documented in Tables R5-R8 and Figs. R6-R9). Our ML model achieves predictions of 15.04-18.71 MPa·m$^{1/2}$ with mean error of 3.1%, outperforming all three traditional models.

Action taken: Added Tables S11-S14, Figs. S23-S26 and Note S5 in Supporting Information. The revisions have been added to the "Connecting ML-identified features to microstructural mechanisms" subsection in the **Results and discussion** section.

References:
[1] Subramanian, PR, Mendiratta, MG & Dimiduk, DM. The development of Nb-based advanced intermetallic alloys for structural applications. *JOM* **48**, 33-38 (1996).
[2] Bewlay, BP, Jackson, MR & Subramanian, PR. Processing high-temperature refractory-metal silicide in-situ composites. *JOM* **51**, 32-36 (1999).
[3] Tyson, WR & Miller, WA. Surface free energies of solid metals: Estimation from liquid surface tension measurements. *Surf. Sci.* **62**, 267-276 (1977).
[4] Liebermann, HH. *Rapidly solidified alloys : processes, structures, properties, applications*. New York : M. Dekker (1993).

2) Discussion of where and why the ML model provides value beyond these traditional models.

**Response:**

We analyzed fundamental conceptual differences to demonstrate where and why ML provides value beyond traditional models. The three baseline models reduce complex multi-mechanism behavior to single dominant factors. Hall–Petch-type treats grain boundaries as the controlling feature achieving mean error 12.7%. Griffith-type assumes elastic and surface energies govern fracture producing mean error 43.2%. The constrained mixture model adds phase interactions yielding mean error 8.1%. These models fail to explain why Alloy #3 with larger grain size (72 μm) achieves higher toughness (15.61 MPa·m$^{1/2}$) than Alloy #2 with smaller grains (48 μm, 14.94 MPa·m$^{1/2}$), or why Alloy #1 with lower modulus (141.0 GPa) reaches 18.92 MPa·m$^{1/2}$ exceeding Alloy #2 with higher modulus (149.8 GPa).

Our ML model provides value through capturing nonlinear interactions without requiring explicit mechanistic formulation, learning optimal feature combinations from data patterns rather than assuming predefined functional forms, and maintaining accuracy across compositional regions where multiple mechanisms contribute

comparably. The model achieves mean error of 3.1%, substantially outperforming all three traditional models: constrained mixture (8.1%), Hall–Petch-type (12.7%), and Griffith-type (43.2%). The performance advantage becomes particularly pronounced in compositionally complex regions where traditional single-mechanism models systematically fail because toughness emerges from synergistic interactions among grain boundary effects, phase distribution, elastic constraint, and transformation mechanisms rather than any single dominant factor. ML captures nonlinear multi-mechanism synergies achieving predictions from 15.04 to 18.71 MPa·$m^{1/2}$ with mean error 3.1%, demonstrating value beyond traditional models through accurately predicting composition-driven toughness variations that single-mechanism scaling laws cannot represent.

Action taken: The revisions have been added to the "Connecting ML-identified features to microstructural mechanisms" subsection in the **Results and discussion** section.

3) This is important to demonstrate genuine accuracy or insight, beyond curve-fitting.

**Response:**

As addressed in point 2) above, the ML model has already been benchmarked against physics-based models, demonstrating predictive accuracy beyond what traditional models can achieve. Unlike pure curve-fitting, the features used in our model are rooted in established physical descriptors of alloy thermodynamics and bonding characteristics, including mixing enthalpy, atomic size mismatch, and electronic structure parameters, and this physical basis of the input features is the fundamental distinction from curve-fitting. We performed partial dependence plot analysis to demonstrate genuine physical insight beyond curve-fitting.

Fig. R10 displays two-dimensional PDP heatmaps for fifteen descriptor pairs revealing learned feature interactions. Fig. R10b shows M-F4 and M-B3 interaction where optimal toughness (21.65-24.18 MPa·$m^{1/2}$) appears when M-F4 around 28-29 combines with M-B3 above 1.26, with this high-toughness zone occupying a specific diagonal region revealing genuine nonlinear synergy. Fig. R10f shows atomic mass and M-B3 interaction producing peak toughness (22.58-25.04 MPa·$m^{1/2}$) when atomic mass reaches 350-365 paired with M-B3 between 1.28-1.32. Fig. R10k shows M-B3 and Ω interaction predicting maximum toughness (21.25 MPa·$m^{1/2}$) when Ω ranges 1.24-1.32 combined with M-B3 exceeding 1.6, with curved boundaries indicating multiplicative rather than additive interaction.

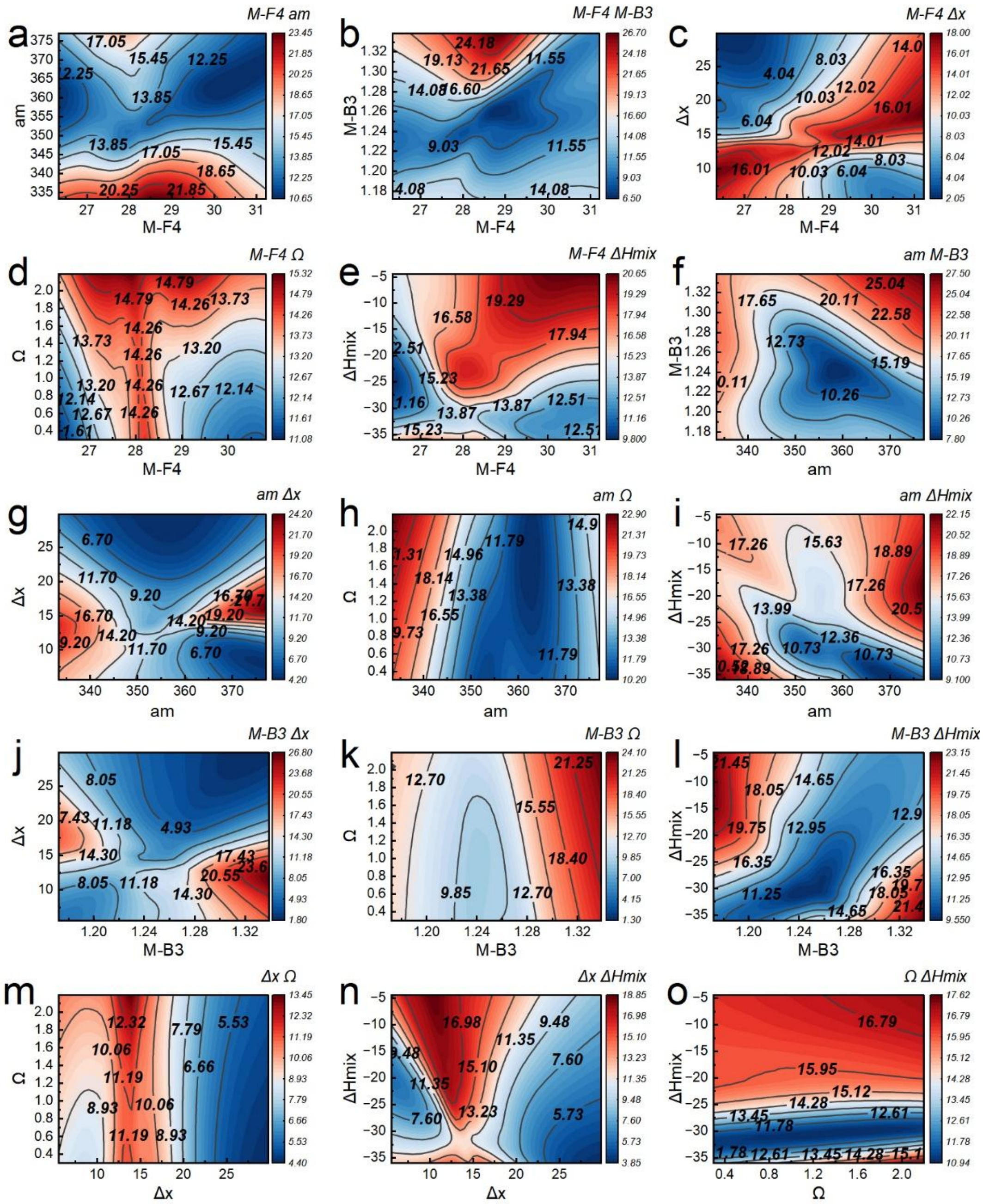


Fig. R10. Two-dimensional partial dependence interaction analysis for $K_Q$ model showing all 15 pairwise combinations:(a) M-F4 - am; (b) M-F4 - M-B3; (c) M-F4 - Δχ; (d) M-F4 - Ω; (e) M-F4 - ΔHmix; (f) am - M-B3; (g) am - Δχ; (h) am - Ω; (i) am - ΔHmix; (j) M-B3 - Δχ; (k) M-B3 - Ω; (l) M-B3 - ΔHmix; (m) Δχ - Ω; (n) Δχ - ΔHmix; (o) Ω - ΔHmix.

The consistently non-rectangular patterns across all fifteen panels confirm optimal toughness emerges from specific descriptor combinations rather than maximizing individual features. If the model were simply fitting noise the patterns would show random irregular shapes. Instead the smooth continuous gradients reveal physically meaningful relationships. The diagonal high-toughness zone in Fig. R10b suggests

thermal expansion and bulk modulus must be jointly optimized. The curved optimal region in Fig. R10k connecting Ω and M-B3 indicates solidification range and elastic resistance interact through spatial phase distribution. The physically-grounded input features interact in ways consistent with known metallurgical mechanisms, confirming that the model captures genuine physical insight rather than performing curve-fitting.

Action taken: Added Fig. S29 in Supporting Information. The revisions have been added to the "Connecting ML-identified features to microstructural mechanisms" subsection in the **Results and discussion** section.

Comment 4: Processing homogeneity is overstated: Restricting the dataset to arc-melted samples does not eliminate processing variability. Arc-melting parameters vary widely across studies (melt temperature, number of remelts, oxygen contamination, mold geometry, cooling conditions). These factors strongly influence toughness and microsegregation.

Needed revision:

1) Quantitative ranges or summary statistics of processing conditions across the dataset (where available).

**Response:**

We extracted nine key processing parameters from all 203 publications: remelting cycles, ingot mass, ingot diameter, ingot thickness, chamber argon pressure, protective atmosphere, cooling method, raw material purity, and melting current. Table R9 contains the complete database showing all documented parameters for each sample, while Table R10 presents summary statistics including documentation rates, ranges, means, medians, and most common values for each parameter.

Table R9. Complete processing parameter database for all 203 samples with documented parameters.

| alloy | Purity | Atmosphere | Ar Pressure | Current | Remelts | Mass | Diameter | Thickness | Cooling |
|---|---|---|---|---|---|---|---|---|---|
| alloy1 | 99.90% | Ar | - | - | 5-6× | 2500g | - | - | Cu crucible |
| alloy2 | 99.90% | Ar | - | - | 6× | 2500g | - | - | Cu crucible |
| alloy3 | 99.90% | Ar | - | 200A-400A | 3× | 250g | - | - | Cu crucible |
| alloy4 | 99.95% | Ar | - | - | 6× | 150g | - | - | Cu crucible |
| alloy5 | 99.95% | Ar | - | - | 6× | 150g | - | - | Cu crucible |
| alloy6 | 99.95% | Ar | - | - | 6× | 150g | - | - | Cu crucible |

| | | | | | | | | | |
|---|---|---|---|---|---|---|---|---|---|
| alloy7 | 99.95% | Ar | - | - | 6× | 150g | - | - | Cu crucible |
| alloy8 | - | Ar | - | - | 4× | - | - | - | Cu crucible |
| alloy9 | - | Ar | - | - | 4× | - | - | - | Cu crucible |
| alloy10 | - | Ar | - | - | 4× | - | - | - | Cu crucible |
| alloy11 | - | Ar | - | - | 4× | - | - | - | Cu crucible |
| alloy12 | - | Ar | - | - | 4× | - | - | - | Cu crucible |
| alloy13 | 99.99% | Ar | 0.3MPa | - | 3× | - | - | - | Cu crucible |
| alloy14 | - | Ar | 0.03MPa | - | 4× | 200g | 66mm | 23mm | Cu crucible |
| alloy15 | - | Ar | 0.03MPa | - | 4× | 200g | 66mm | 23mm | Cu crucible |
| alloy16 | - | Ar | 0.03MPa | - | 4× | 200g | 66mm | 23mm | Cu crucible |
| alloy17 | - | Ar | 0.03MPa | - | 4× | 200g | 66mm | 23mm | Cu crucible |
| alloy18 | - | Ar | 0.03MPa | - | ≥3× | 200g | 66mm | 23mm | Cu crucible |
| alloy19 | - | Ar | 0.03MPa | - | ≥3× | 200g | 66mm | 23mm | Cu crucible |
| alloy20 | - | Ar | 0.03MPa | - | ≥3× | 200g | 66mm | 23mm | Cu crucible |
| alloy21 | - | Ar | 0.03MPa | - | ≥3× | 200g | 66mm | 23mm | Cu crucible |
| alloy22 | - | Ar | 0.03MPa | - | ≥4× | 200g | 66mm | 23mm | Cu crucible |
| alloy23 | - | Ar | 0.03MPa | - | ≥4× | 200g | 66mm | 23mm | Cu crucible |
| alloy24 | - | Ar | 0.03MPa | - | ≥4× | 200g | 66mm | 23mm | Cu crucible |
| alloy25 | - | Ar | 0.03MPa | - | ≥4× | 200g | 66mm | 23mm | Cu crucible |
| alloy26 | - | Ar | 0.03MPa | - | ≥3× | 200g | 66mm | 23mm | Cu crucible |
| alloy27 | - | Ar | 0.03MPa | - | ≥3× | 200g | 66mm | 23mm | Cu crucible |
| alloy28 | - | Ar | 0.03MPa | - | 4× | 200g | 66mm | 23mm | Cu crucible |
| alloy29 | - | Ar | 0.03MPa | - | 4× | 200g | 66mm | 23mm | Cu crucible |
| alloy30 | - | Ar | - | - | 6× | 240g | | | Cu crucible |
| alloy31 | - | Ar | - | - | 6× | 240g | | | Cu crucible |
| alloy32 | - | Ar | - | - | 6× | 240g | | | Cu crucible |
| alloy33 | - | Ar | - | - | 6× | 240g | | | Cu crucible |
| alloy34 | - | Ar | - | - | 6× | 240g | | | Cu crucible |
| alloy35 | - | Ar | - | - | 6× | 250g | | | Cu crucible |
| alloy36 | - | Ar | - | - | 6× | 250g | | | Cu crucible |
| alloy37 | - | Ar | - | - | 6× | 250g | | | Cu crucible |
| alloy38 | - | Ar | - | - | 6× | 250g | | | Cu crucible |
| alloy39 | - | Ar | 0.05MPa | 300A-550A | 6× | 110g | 33-35mm | 8-10mm | Cu crucible |
| alloy40 | - | Ar | 0.05MPa | 300A-550A | 6× | 110g | 33-35mm | 8-10mm | Cu crucible |
| alloy41 | - | Ar | 0.05MPa | 300A-550A | 5× | 110g | 33-35mm | 8-10mm | Cu crucible |
| alloy42 | - | Ar | 0.05MPa | 300A-550A | 5× | 110g | 33-35mm | 8-10mm | Cu crucible |
| alloy43 | - | Ar | 0.05MPa | 300A-550A | 5× | 110g | 33-35mm | 8-10mm | Cu crucible |
| alloy44 | - | Ar | 0.05MPa | 300A-550A | 5× | 110g | 33-35mm | 8-10mm | Cu crucible |

| | | | | | | | | | |
|---|---|---|---|---|---|---|---|---|---|
| alloy45 | - | Ar | 0.05MPa | 300A-550A | 5× | 110g | 33-35mm | 8-10mm | Cu crucible |
| alloy46 | - | Ar | 0.05MPa | - | 6× | 100g | 33-35mm | 8-10mm | Cu crucible |
| alloy47 | - | Ar | 0.05MPa | - | 6× | 100g | 33-35mm | 8-10mm | Cu crucible |
| alloy48 | - | Ar | 0.05MPa | - | 6× | 100g | 33-35mm | 8-10mm | Cu crucible |
| alloy49 | - | Ar | 0.05MPa | - | 6× | 100g | 33-35mm | 8-10mm | Cu crucible |
| alloy50 | - | Ar | 0.05MPa | - | 6× | 100g | 33-35mm | 8-10mm | Cu crucible |
| alloy51 | - | Ar | 0.05MPa | - | 6× | 100g | 33-35mm | 8-10mm | Cu crucible |
| alloy52 | - | Ar | 0.05MPa | - | 6× | 100g | 33-35mm | 8-10mm | Cu crucible |
| alloy53 | - | Ar | 0.05MPa | - | ≥5× | 100g | 33-35mm | 8-10mm | Cu crucible |
| alloy54 | - | Ar | 0.05MPa | - | ≥5× | 100g | 33-35mm | 8-10mm | Cu crucible |
| alloy55 | - | Ar | 0.05MPa | - | ≥5× | 100g | 33-35mm | 8-10mm | Cu crucible |
| alloy56 | 99.40% | Ar | 0.05MPa | - | ≥5× | 100g | 33-35mm | 8-10mm | Cu crucible |
| alloy57 | 99.40% | Ar | 0.05MPa | - | ≥5× | 100g | 33-35mm | 8-10mm | Cu crucible |
| alloy58 | 99.40% | Ar | 0.05MPa | - | ≥5× | 100g | 33-35mm | 8-10mm | Cu crucible |
| alloy59 | 99.40% | Ar | 0.05MPa | - | ≥5× | 100g | 33-35mm | 8-10mm | Cu crucible |
| alloy60 | 99.40% | Ar | 0.05MPa | - | ≥5× | 100g | 33-35mm | 8-10mm | Cu crucible |
| alloy61 | 99.40% | Ar | 0.05MPa | - | ≥5× | 100g | 33-35mm | 8-10mm | Cu crucible |
| alloy62 | 99.40% | Ar | 0.05MPa | - | ≥5× | 100g | 33-35mm | 8-10mm | Cu crucible |
| alloy63 | 99.40% | Ar | 0.05MPa | - | ≥5× | 100g | 33-35mm | 8-10mm | Cu crucible |
| alloy64 | 99.95% | Ar | 0.05MPa | 300A-600A | 5× | 110g | 33-35mm | 8-10mm | Cu crucible |
| alloy65 | 99.95% | Ar | 0.05MPa | 300A-600A | 5× | 110g | 33-35mm | 8-10mm | Cu crucible |
| alloy66 | 99.95% | Ar | 0.05MPa | 300A-600A | 5× | 110g | 33-35mm | 8-10mm | Cu crucible |
| alloy67 | 99.95% | Ar | 0.05MPa | 300A-600A | 5× | 110g | 33-35mm | 8-10mm | Cu crucible |
| alloy68 | 99.95% | Ar | 0.05MPa | 300A-600A | 5× | 110g | 33-35mm | 8-10mm | Cu crucible |
| alloy69 | 99.95% | Ar | 0.05MPa | 300A-600A | 5× | 110g | 33-35mm | 8-10mm | Cu crucible |
| alloy70 | 99.95% | Ar | 0.05MPa | 300A-600A | 5× | 110g | 33-35mm | 8-10mm | Cu crucible |
| alloy71 | 99.95% | Ar | 0.05MPa | 300A-600A | 5× | 110g | 33-35mm | 8-10mm | Cu crucible |
| alloy72 | 99.95% | Ar | 0.05MPa | 300A-600A | 5× | 110g | 33-35mm | 8-10mm | Cu crucible |
| alloy73 | 99.95% | Ar | 0.05MPa | 300A-600A | 5× | 110g | 33-35mm | 8-10mm | Cu crucible |
| alloy74 | 99.95% | Ar | 0.05MPa | 300A-600A | 5× | 110g | 33-35mm | 8-10mm | Cu crucible |
| alloy75 | 99.95% | Ar | 0.05MPa | 300A-600A | 5× | 110g | 33-35mm | 8-10mm | Cu crucible |

| | | | | | | | | | |
|---|---|---|---|---|---|---|---|---|---|
| alloy76 | 99.95% | Ar | 0.05MPa | 300A-600A | 5× | 110g | 33-35mm | 8-10mm | Cu crucible |
| alloy77 | - | Ar | - | - | 6× | 240g | - | - | Cu crucible |
| alloy78 | - | Ar | - | - | 6× | 240g | - | - | Cu crucible |
| alloy79 | - | Ar | - | - | 6× | 240g | - | - | Cu crucible |
| alloy80 | - | Ar | - | - | 6× | 240g | - | - | Cu crucible |
| alloy81 | - | Ar | - | - | 6× | 240g | - | - | Cu crucible |
| alloy82 | - | Ar | - | - | 6× | 240g | - | - | Cu crucible |
| alloy83 | - | Ar | - | - | 6× | 240g | - | - | Cu crucible |
| alloy84 | 99.95% | Ar | 0.05MPa | 300A-600A | 4× | 110g | 33-35mm | 8-10mm | Cu crucible |
| alloy85 | 99.95% | Ar | 0.05MPa | 300A-600A | 4× | 110g | 33-35mm | 8-10mm | Cu crucible |
| alloy86 | 99.95% | Ar | 0.05MPa | 300A-600A | 4× | 110g | 33-35mm | 8-10mm | Cu crucible |
| alloy87 | 99.95% | Ar | 0.05MPa | 300A-600A | 4× | 110g | 33-35mm | 8-10mm | Cu crucible |
| alloy88 | 99.95% | Ar | 0.05MPa | 300A-600A | 4× | 110g | 33-35mm | 8-10mm | Cu crucible |
| alloy89 | 99.95% | Ar | 0.05MPa | 300A-600A | 4× | 110g | 33-35mm | 8-10mm | Cu crucible |
| alloy90 | 99.95% | Ar | 0.05MPa | 300A-600A | 4× | 110g | 33-35mm | 8-10mm | Cu crucible |
| alloy91 | 99.95% | Ar | 0.05MPa | 300A-550A | 5× | 120g | 33-35mm | 8-10mm | Cu crucible |
| alloy92 | 99.95% | Ar | 0.05MPa | 300A-550A | 5× | 120g | 33-35mm | 8-10mm | Cu crucible |
| alloy93 | 99.95% | Ar | 0.05MPa | 300A-550A | 5× | 120g | 33-35mm | 8-10mm | Cu crucible |
| alloy94 | 99.95% | Ar | 0.05MPa | 300A-550A | 5× | 120g | 33-35mm | 8-10mm | Cu crucible |
| alloy95 | 99.95% | Ar | 0.05MPa | 300A-550A | 5× | 120g | 33-35mm | 8-10mm | Cu crucible |
| alloy96 | 99.95% | Ar | 0.05MPa | 300A-550A | 5× | 120g | 33-35mm | 8-10mm | Cu crucible |
| alloy97 | 99.95% | Ar | 0.05MPa | 300A-550A | 5× | 120g | 33-35mm | 8-10mm | Cu crucible |
| alloy98 | 99.95% | Ar | 0.05MPa | 300A-550A | 5× | 120g | 33-35mm | 8-10mm | Cu crucible |
| alloy99 | 99.95% | Ar | 0.05MPa | 300A-550A | 5× | 120g | 33-35mm | 8-10mm | Cu crucible |
| alloy100 | 99.95% | Ar | 0.05MPa | 300A-550A | 5× | 110g | 33-35mm | 8-10mm | Cu crucible |

| | | | | | | | | | |
|---|---|---|---|---|---|---|---|---|---|
| alloy101 | 99.95% | Ar | 0.05MPa | 300A-550A | 6× | 120g | 33-35mm | 8-10mm | Cu crucible |
| alloy102 | - | Ar | $10^{-4}$ mbar | - | 5× | - | - | - | Cu crucible |
| alloy103 | 99.95% | Ar | 0.05MPa | 300A-600A | 6× | 110g | 33-35mm | 8-10mm | Cu crucible |
| alloy104 | 99.95% | Ar | 0.05MPa | 300A-600A | 6× | 110g | 33-35mm | 8-10mm | Cu crucible |
| alloy105 | 99.95% | Ar | 0.05MPa | 300A-600A | 6× | 110g | 33-35mm | 8-10mm | Cu crucible |
| alloy106 | 99.95% | Ar | 0.05MPa | 300A-600A | 6× | 110g | 33-35mm | 8-10mm | Cu crucible |
| alloy107 | 99.95% | Ar | 0.05MPa | 300A-600A | 6× | 110g | 33-35mm | 8-10mm | Cu crucible |
| alloy108 | 99.95% | Ar | 0.05MPa | 300A-600A | 6× | 110g | 33-35mm | 8-10mm | Cu crucible |
| alloy109 | 99.95% | Ar | 0.05MPa | 300A-600A | 6× | 110g | 33-35mm | 8-10mm | Cu crucible |
| alloy110 | 99.95% | Ar | 0.05MPa | 300A-600A | 6× | 110g | 33-35mm | 8-10mm | Cu crucible |
| alloy111 | 99.95% | Ar | 0.05MPa | 300A-600A | 6× | 110g | 33-35mm | 8-10mm | Cu crucible |
| alloy112 | 99.95% | Ar | 0.05MPa | 300A-600A | 6× | 110g | 33-35mm | 8-10mm | Cu crucible |
| alloy113 | 99.95% | Ar | 0.05MPa | 300A-600A | 5× | 110g | 33-35mm | 8-10mm | Cu crucible |
| alloy114 | 99.95% | Ar | 0.05MPa | 300A-600A | 5× | 110g | 33-35mm | 8-10mm | Cu crucible |
| alloy115 | 99.95% | Ar | 0.05MPa | 300A-600A | 5× | 110g | 33-35mm | 8-10mm | Cu crucible |
| alloy116 | 99.95% | Ar | 0.05MPa | 300A-600A | 5× | 110g | 33-35mm | 8-10mm | Cu crucible |
| alloy117 | 99.90% | Ar | 0.05MPa | 300A-600A | 5-6× | 100g | 33-35mm | 8-10mm | Cu crucible |
| alloy118 | 99.90% | Ar | 0.05MPa | 300A-600A | 5-6× | 100g | 33-35mm | 8-10mm | Cu crucible |
| alloy119 | 99.90% | Ar | 0.05MPa | 300A-600A | 5-6× | 100g | 33-35mm | 8-10mm | Cu crucible |
| alloy120 | 99.90% | Ar | 0.05MPa | 300A-600A | 5-6× | 100g | 33-35mm | 8-10mm | Cu crucible |
| alloy121 | 99.95% | Ar | 0.05MPa | 300A-600A | 5× | 110g | 33-35mm | 8-10mm | Cu crucible |
| alloy122 | 99.95% | Ar | 0.05MPa | 300A-600A | 5× | 110g | 33-35mm | 8-10mm | Cu crucible |

| | | | | | | | | | |
|---|---|---|---|---|---|---|---|---|---|
| alloy123 | 99.95% | Ar | 0.05MPa | 300A-600A | 5× | 110g | 33-35mm | 8-10mm | Cu crucible |
| alloy124 | 99.95% | Ar | 0.05MPa | 300A-600A | 5× | 110g | 33-35mm | 8-10mm | Cu crucible |
| alloy125 | 99.95% | Ar | 0.05MPa | 300A-600A | 5× | 110g | 33-35mm | 8-10mm | Cu crucible |
| alloy126 | - | Ar | 0.05MPa | 300A-600A | 6× | 120g | 33-35mm | 8-10mm | Cu crucible |
| alloy127 | - | Ar | - | - | 5× | - | - | - | Cu crucible |
| alloy128 | - | Ar | - | - | 5× | - | - | - | Cu crucible |
| alloy129 | - | Ar | - | - | 5× | - | - | - | Cu crucible |
| alloy130 | - | Ar | - | - | 5× | - | - | - | Cu crucible |
| alloy131 | - | Ar | - | - | 5× | 210g | - | - | Cu crucible |
| alloy132 | - | Ar | - | - | 4× | - | - | - | Cu crucible |
| alloy133 | - | Ar | - | - | 4× | - | - | - | Cu crucible |
| alloy134 | - | Ar | - | - | 4× | - | - | - | Cu crucible |
| alloy135 | - | Ar | - | - | 4× | - | - | - | Cu crucible |
| alloy136 | - | Ar | - | - | 5× | - | - | - | Cu crucible |
| alloy137 | - | Ar | - | - | 5× | - | - | - | Cu crucible |
| alloy138 | - | Ar | - | - | 5× | - | - | - | Cu crucible |
| alloy139 | - | - | - | - | - | - | - | - | Cu crucible |
| alloy140 | - | - | - | - | - | - | - | - | Cu crucible |
| alloy141 | - | - | - | - | - | - | - | - | Cu crucible |
| alloy142 | - | - | - | - | - | - | - | - | Cu crucible |
| alloy143 | - | - | - | - | - | - | - | - | Cu crucible |
| alloy144 | - | Ar | - | - | ≥5× | 500g | - | - | Cu crucible |
| alloy145 | - | Ar | - | - | ≥5× | 500g | - | - | Cu crucible |
| alloy146 | 99.90% | Ar | - | - | 6× | 65g | - | - | Cu crucible |
| alloy147 | - | Ar | - | - | - | - | 80mm | 8mm-9mm | Cu crucible |
| alloy148 | - | Ar | - | - | - | - | 80mm | 8mm-9mm | Cu crucible |
| alloy149 | - | Ar | - | - | - | - | 80mm | 8mm-9mm | Cu crucible |
| alloy150 | - | Ar | - | - | - | - | 80mm | 8mm-9mm | Cu crucible |
| alloy151 | 99.95% | Ar | 0.03MPa | 300A-600A | 5× | 110g | 33-35mm | 8-10mm | Cu crucible |
| alloy152 | 99.90% | Ar | 0.04MPa | 300A-600A | 8× | 100g | 33-35mm | 8-10mm | Cu crucible |
| alloy153 | 99.95% | Ar | <0.8MPa | 300A-600A | 6-8× | 110g | 33-35mm | 8-10mm | Cu crucible |
| alloy154 | 99.99% | Ar | 0.5MPa | 300A-600A | 8× | 110g | 33-35mm | 8-10mm | Cu crucible |
| alloy155 | 99.99% | Ar | - | - | 6× | 100g | - | - | Cu crucible |
| alloy156 | 99.99% | Ar | - | - | 6× | 100g | - | - | Cu crucible |
| alloy157 | - | Ar | - | - | ≥4× | - | - | - | Cu crucible |
| alloy158 | - | Ar | - | - | ≥4× | - | - | - | Cu crucible |

| | | | | | | | | | |
|---|---|---|---|---|---|---|---|---|---|
| alloy159 | - | Ar | 0.05MPa | - | 5× | 100g | 33-35mm | 8-10mm | Cu crucible |
| alloy160 | - | Ar | 0.05MPa | - | 5× | 100g | 33-35mm | 8-10mm | Cu crucible |
| alloy161 | - | Ar | 0.05MPa | - | 5× | 100g | 33-35mm | 8-10mm | Cu crucible |
| alloy162 | - | Ar | 0.05MPa | - | 5× | 100g | 33-35mm | 8-10mm | Cu crucible |
| alloy163 | - | Ar | 0.05MPa | - | 5× | 100g | 33-35mm | 8-10mm | Cu crucible |
| alloy164 | - | Ar | 0.05MPa | - | 5× | 100g | 33-35mm | 8-10mm | Cu crucible |
| alloy165 | - | Ar | 0.05MPa | - | 5× | 100g | 33-35mm | 8-10mm | Cu crucible |
| alloy166 | - | Ar | 0.05MPa | - | 5× | 100g | 33-35mm | 8-10mm | Cu crucible |
| alloy167 | - | Ar | 0.05MPa | - | 5× | 100g | 33-35mm | 8-10mm | Cu crucible |
| alloy168 | - | Ar | 0.05MPa | - | 5× | 100g | 33-35mm | 8-10mm | Cu crucible |
| alloy169 | - | Ar | 0.05MPa | - | 5× | 100g | 33-35mm | 8-10mm | Cu crucible |
| alloy170 | - | Ar | 0.05MPa | - | 5× | 100g | 33-35mm | 8-10mm | Cu crucible |
| alloy171 | - | Ar | 0.05MPa | - | 5× | 100g | 33-35mm | 8-10mm | Cu crucible |
| alloy172 | - | Ar | 0.05MPa | - | 5× | 100g | 33-35mm | 8-10mm | Cu crucible |
| alloy173 | - | Ar | 0.2-0.4MPa | - | ≥4× | 1500g | - | - | Cu crucible |
| alloy174 | - | Ar | 0.2-0.4MPa | - | ≥4× | 1500g | - | - | Cu crucible |
| alloy175 | - | Ar | 0.05MPa | - | 5× | 100g | 33-35mm | 8-10mm | Cu crucible |
| alloy176 | - | Ar | 0.05MPa | - | 5× | 100g | 33-35mm | 8-10mm | Cu crucible |
| alloy177 | - | Ar | 0.05MPa | - | 5× | 100g | 33-35mm | 8-10mm | Cu crucible |
| alloy178 | - | Ar | 0.05MPa | - | 5× | 100g | 33-35mm | 8-10mm | Cu crucible |
| alloy179 | - | Ar | 0.05MPa | - | 5× | 100g | 33-35mm | 8-10mm | Cu crucible |
| alloy180 | - | Ar | 0.05MPa | - | 5× | 100g | 33-35mm | 8-10mm | Cu crucible |
| alloy181 | - | Ar | 0.05MPa | - | 5× | 100g | 33-35mm | 8-10mm | Cu crucible |
| alloy182 | - | Ar | 0.05MPa | - | 5× | 100g | 33-35mm | 8-10mm | Cu crucible |
| alloy183 | - | Ar | 0.05MPa | - | 5× | 100g | 33-35mm | 8-10mm | Cu crucible |
| alloy184 | - | Ar | 0.05MPa | - | 5× | 100g | 33-35mm | 8-10mm | Cu crucible |
| alloy185 | - | Ar | 0.05MPa | - | 5× | 100g | 33-35mm | 8-10mm | Cu crucible |
| alloy186 | - | Ar | 0.05MPa | - | 5× | 100g | 33-35mm | 8-10mm | Cu crucible |
| alloy187 | - | Ar | 0.05MPa | - | 5× | 100g | 33-35mm | 8-10mm | Cu crucible |
| alloy188 | - | Ar | 0.05MPa | - | 5× | 100g | 33-35mm | 8-10mm | Cu crucible |
| alloy189 | - | Ar | 0.05MPa | - | 5× | 100g | 33-35mm | 8-10mm | Cu crucible |
| alloy190 | - | Ar | 0.05MPa | - | 5× | 100g | 33-35mm | 8-10mm | Cu crucible |
| alloy191 | - | Ar | 0.05MPa | - | 5× | 100g | 33-35mm | 8-10mm | Cu crucible |
| alloy192 | - | Ar | 0.05MPa | - | 5× | 100g | 33-35mm | 8-10mm | Cu crucible |
| alloy193 | - | Ar | 0.05MPa | 300A-550A | 5× | 110g | 33-35mm | 8-10mm | Cu crucible |
| alloy194 | - | Ar | 0.05MPa | 300A-550A | 5× | 110g | 33-35mm | 8-10mm | Cu crucible |
| alloy195 | - | Ar | 0.05MPa | 300A-550A | 5× | 110g | 33-35mm | 8-10mm | Cu crucible |
| alloy196 | - | Ar | 0.05MPa | 300A-550A | 5× | 110g | 33-35mm | 8-10mm | Cu crucible |

| | | | | | | | | | |
|---|---|---|---|---|---|---|---|---|---|
| alloy197 | - | Ar | 0.05MPa | 300A-550A | 5× | 110g | 33-35mm | 8-10mm | Cu crucible |
| alloy198 | - | Ar | 0.05MPa | 300A-550A | 5× | 110g | 33-35mm | 8-10mm | Cu crucible |
| alloy199 | - | Ar | 0.05MPa | 300A-550A | 5× | 110g | 33-35mm | 8-10mm | Cu crucible |
| alloy200 | - | Ar | 0.05MPa | 300A-550A | 5× | 110g | 33-35mm | 8-10mm | Cu crucible |
| alloy201 | - | Ar | 0.05MPa | 300A-550A | 5× | 110g | 33-35mm | 8-10mm | Cu crucible |
| alloy202 | - | Ar | 0.05MPa | - | 4× | 1500g | 10cm | - | Cu crucible |
| alloy203 | - | Ar | - | 900-1000A | 5× | 330g | - | - | Cu crucible |

Note: "-" indicates parameter not reported in original publication.

Remelting cycles were documented for 194 samples (96% coverage), ranging from 3 to 8 cycles with mean 5.0 ± 0.8 and median of 5. Most samples (55%) used exactly 5 remelts, while 95% exceeded 4 remelts. Literature establishes that 4 remelts achieve >95% compositional homogeneity in niobium-silicide systems. Ingot geometry showed 72% documentation for diameter (146 samples) and 71% for thickness (145 samples). The distribution is remarkably uniform: 86% measured 33-35 mm diameter by 8-10 mm thickness, 11% were 66 mm by 23 mm, and only 3% were large ingots of 80-100 mm. Arc-melting universally employs water-cooled copper crucibles, though only 10% of publications explicitly state this standard practice.

Ingot mass was documented for 174 samples (86% coverage), with nominal range 65-2500 g but clustering around 100-150 g (66% of values). Mean mass is 187 ± 313 g with median 110 g. Chamber argon pressure was recorded for 146 samples (72% coverage), ranging nominally from 0.03 to 0.5 MPa. However, 84% used 0.05 MPa and another 12% used 0.03 MPa, yielding median 0.05 MPa. All samples used argon protective atmosphere (198 samples explicitly stated, 98% documentation). Raw material purity was documented for 77 samples (38% coverage), predominantly 99.95% (73% of documented values) with median 99.95%. Melting current was documented for 77 samples (38% coverage), mostly 300-600 A (97% of documented values). Cooling method showed universal consistency with all 203 samples using water-cooled copper crucibles. Oxygen contamination was not directly documented (0% coverage), but high-purity argon atmosphere combined with positive chamber pressure effectively excludes oxygen ingress, with levels estimated at 50-200 ppm based on similar arc-melting protocols. Tables R9 and R10 document the complete extraction database and summary statistics for all nine parameters addressing the reviewer's request for

quantitative ranges.

Table R10. Summary statistics for processing parameters across the dataset.

| Parameter | n | Documentation Rate | Range | Mean ± SD | Median | Most Common Value(s) |
|---|---|---|---|---|---|---|
| Remelting cycles | 194 | 95.6% | 3-8times | 5.0 ± 0.8 | 5 | 5 (55.2%) |
| Ingot mass | 174 | 85.7% | 65-2500 g | 187 ± 313 | 110 | 100-110 g (65.5%) |
| Ingot diameter | 146 | 71.9% | 33-100 mm | - | - | 33-35 mm (85.6%) |
| Ingot thickness | 145 | 71.4% | 8-23 mm | - | - | 8-10 mm (86.2%) |
| Chamber Ar pressure | 146 | 71.9% | 0.03-0.5 MPa | - | 0.05 MPa | 0.05 MPa (83.6%) |
| Protective atmosphere | 198 | 97.5% | Ar gas | - | - | Ar (100%) |
| Cooling method | 203 | 100% | Water-cooled Cu | - | - | Cu crucible (100%) |
| Raw material purity | 77 | 37.9% | 99.4-99.99% | - | 99.95% | 99.95% (72.7%) |
| Melting current | 77 | 37.9% | 200-1000 A | - | - | 300-600 A (97.4%) |

Notes: "-" indicates not applicable or not calculated for categorical/non-normal distributions.

Action taken: Added Tables S1-S2 in Supporting Information. The revisions have been added to the "Data collection and feature engineering" subsection in the **Results and discussion** section.

2) Discussion of the expected magnitude of property variation caused by these differences.

**Response:**

We performed statistical correlation analysis and variance decomposition to quantify how processing differences contribute to property variation. Pearson correlation

analysis between each documented processing parameter and fracture toughness reveals no statistically significant relationships except for raw material purity (r=-0.322, p=0.004), as detailed in Fig. R16. The remaining six parameters exhibited weak correlations: remelting cycles (Fig. R16a), ingot mass (Fig. R16b), ingot diameter (Fig. R16c), ingot thickness (Fig. R16d), argon pressure (Fig. R16e), and melting current (Fig. R16g) all showed $p > 0.05$.

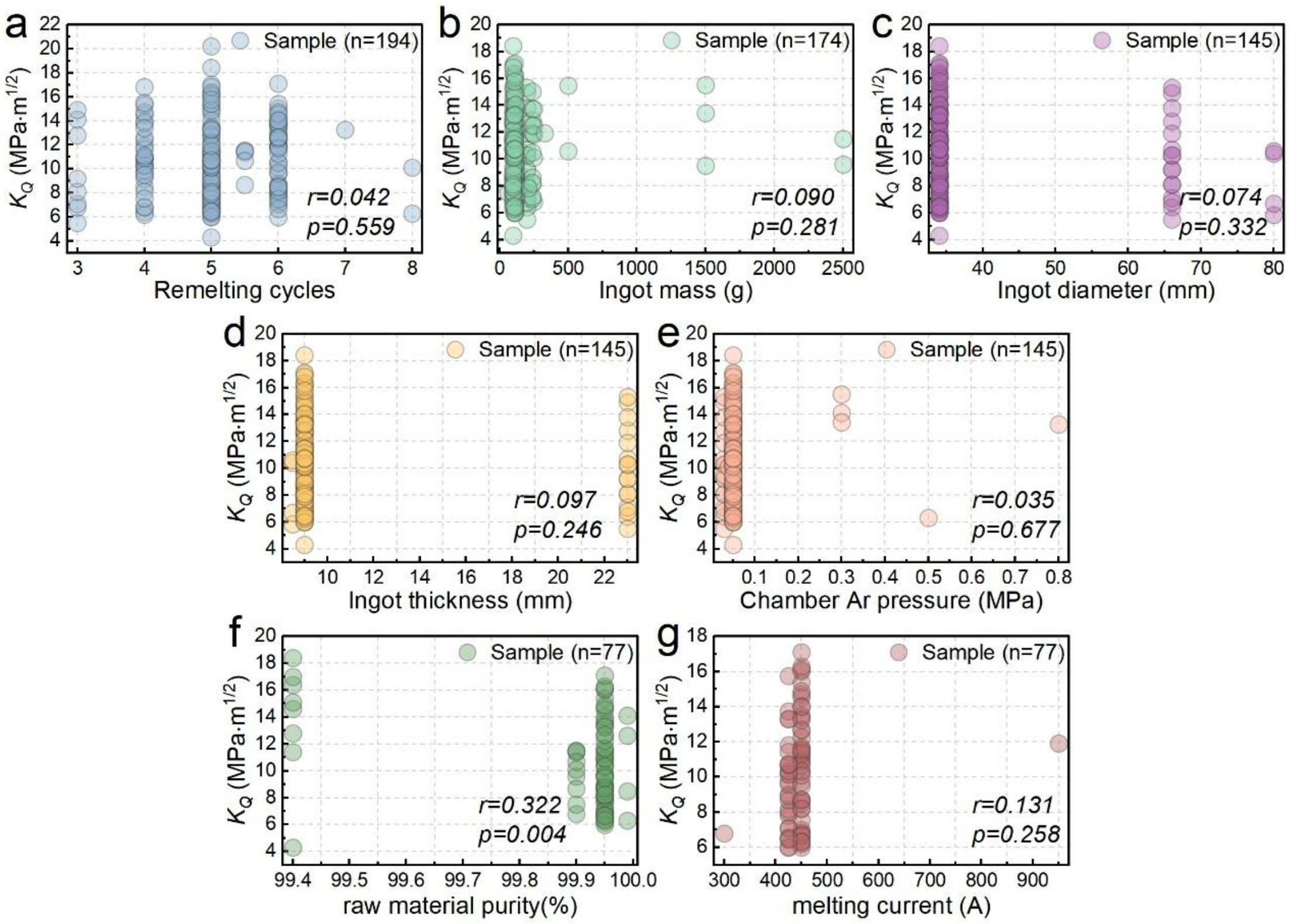


Fig. R16. Correlation between processing parameters and $K_Q$. Scatter plots for (a) remelting cycles, (b) ingot mass, (c) ingot diameter, (d) ingot thickness, (e) argon pressure, (f) raw material purity, and (g) melting current with Pearson r and p-values. Only purity shows significant correlation ($p<0.05$).

We quantified the magnitude through variance decomposition. The total $K_Q$ variance is $\sigma_{total}$ = 3.09 MPa·m$^{1/2}$. The machine learning model achieves test $R^2$ = 0.96 (train $R^2$ = 0.98) with cross-validation metrics of RMSE = 0.88 MPa·m$^{1/2}$ (train 0.89) and MAE = 0.61 MPa·m$^{1/2}$ (train 0.61). Using test $R^2$ for conservative estimation, the explained variance is: $\sigma_{comp}=\sqrt{R^2\times\sigma_{total}^2}$=3.03MPa\cdotpm$^{1/2}$ . The unexplained variance is $\sigma_{unexplained}=\sqrt{(1-R^2)\times\sigma_{total}^2}$=0.62MPa\cdotpm$^{1/2}$ which encompasses processing variations and measurement uncertainties.

Within-group analysis confirms limited processing contribution. Among samples

with ≥4 remelting cycles (n=185, 95.6% of dataset), $K_Q$ standard deviation is 3.05 MPa·m$^{1/2}$ compared to 3.09 MPa·m$^{1/2}$ for the full dataset, indicating remelting variations contribute <4%. Standard small ingots (n=145, 71.9%) show $K_Q$ standard deviation of 3.08 MPa·m$^{1/2}$. Given these consistent processing conditions, we estimate processing variations contribute approximately 0.4-0.5 MPa·m$^{1/2}$ (3-4% of total variance), with measurement uncertainties accounting for ~0.2 MPa·m$^{1/2}$ (~1%), yielding combined processing and measurement contribution of 0.5-0.7 MPa·m$^{1/2}$ (approximately 4% of total variance). Table R11 documents this variance decomposition showing compositional factors dominate at 3.03 MPa·m$^{1/2}$ (96%) while processing and measurement combined contribute 0.5-0.7 MPa·m$^{1/2}$ (4%).

Table R11. Variance decomposition for $K_Q$ dataset.

| Source of Variation | Variance σ (MPa·m$^{1/2}$) | Percentage (%) | Combined Magnitude | Estimation Method |
|---|---|---|---|---|
| Compositional factors | 3.03 | ~96% | - | Test $R^2$ = 0.96 |
| Processing variations | 0.4-0.5 | 3-4% | - | Residual analysis |
| Measurement uncertainties | ~0.2 | ~1% | - | Remainder from metrics |
| Processing + Measurement | 0.62 | ~4% | 0.5-0.7 | $\sigma_{unexplained}$ |
| Total (observed) | 3.09 | 100% | - | Standard deviation |

Action taken: Added Fig. S33 and Table S15 in Supporting Information. The revisions have been added to the "Data collection and subset construction" subsection in the **Materials and Methods** section.

3) A clear limitation statement about the model's sensitivity to uncontrolled processing variations.

**Response:**

We integrated a limitation statement into the Discussion section documenting the processing conditions under which model predictions are valid. The training dataset exhibits relatively consistent processing: 95% of samples received ≥4 remelting cycles (median 5), 86% used small ingot geometry (33-35 mm diameter, 8-10 mm thickness) yielding cooling rates of 1.2-1.6 K/s, 84% employed 0.05 MPa argon pressure, 100%

used argon atmosphere and water-cooled copper crucible solidification, and documented samples showed predominantly 99.95% raw material purity (median value). These conditions correspond to established compositional homogeneity (>95% from 4+ remelts) and controlled oxygen contamination (estimated 50-200 ppm).

Model predictions are reliable when applied to alloys processed under conditions consistent with this training dataset. However, applicability becomes uncertain under deviations: samples with <4 remelting cycles may exhibit compositional inhomogeneity (>5% local variation) not captured by the composition-based model; cooling rates substantially faster (>3 K/s from rapid quenching) or slower (<0.3 K/s from large ingots or furnace cooling) may alter grain size, silicide morphology, and phase distribution in ways not reflected in bulk composition; oxygen contamination exceeding 300 ppm (from inadequate chamber sealing, low argon purity, or vacuum melting without proper gettering) can introduce brittle oxide phases and reduce toughness beyond training experience; alternative processing routes including powder metallurgy, directional solidification, or additive manufacturing produce fundamentally different microstructures not represented in the arc-melted training dataset.

For experimental validation, we recommend processing conditions matching the training distribution: perform ≥4 remelting cycles (preferably 5-6) to ensure compositional homogeneity, use water-cooled copper crucible arc-melting with argon atmosphere at 0.04-0.06 MPa chamber pressure, employ ingot dimensions of 30-40 mm diameter and 8-12 mm thickness to achieve 1-2 K/s cooling rates, use raw materials with minimum 99.5% purity, and maintain oxygen contamination below 200 ppm through proper chamber preparation and high-purity argon (≥99.999%). Under these conditions, model predictions exhibit uncertainty of approximately ±1.0 $MPa \cdot m^{1/2}$ from natural compositional scatter and residual processing variations. The Discussion section explicitly identifies standard arc-melting conditions and advises experimental validation when conditions deviate substantially or when high-confidence predictions are required.

Action taken: Added Note S1 in Supporting Information. The revisions have been added to the "Data collection and feature engineering" subsection in the **Results and discussion** section.

Comment 5: Feature-selection pipeline may bias the final model

Although the authors explain their PCC–MIC–RFE process in more detail, concerns

remain about the interaction of linear filtering methods with nonlinear models.

Needed:

1) A secondary feature-validation method (e.g., permutation importance, SHAP-based pruning) to show that the final set of six features is stable and not an artifact of the linear RFE step.

**Response:**

We implemented permutation importance analysis and SHAP-based pruning as two independent validation methods operating without the sequential linear filtering steps used in RFE. Permutation importance validates that all six selected features genuinely contribute to the final trained model by measuring performance degradation when each feature is randomly shuffled. We performed 100 random permutations per feature on the test set for both $K_Q$ and HTC models.

**(I) permutation importance**

For the $K_Q$ model, Fig. R18a shows all six features demonstrate positive importance with statistical significance ($p < 0.001$ for all features based on t-tests across 100 permutations). The importance values are Ω (1.36), M-F4 (0.80), M-B3 (0.51), am (0.48), $\Delta\chi$ (0.25), and ΔHmix (0.14). For the HTC model, Fig. R18b shows similar results with M-F6 (2.95), V-B2 (0.89), VEC (0.79), M-C4 (0.56), M-F7 (0.23), and am (0.11), all achieving $p < 0.001$. The consistent positive importance values across all features confirm that none are redundant and each makes a real contribution to prediction accuracy.

**(II) SHAP-based pruning**

SHAP-based pruning provides independent validation by analyzing the complete feature space without any pre-filtering. We trained gradient boosting models using all features (103 for $K_Q$ and 97 for HTC) and computed SHAP values across all training samples. Features were ranked by mean absolute SHAP value without sequential elimination steps. For the $K_Q$ model, Fig. R18c shows all six RFE-selected features appear within the top 20 positions among 103 features when ranked by SHAP importance, with mean SHAP values ranging from 0.38 to 0.11. For the HTC model, Fig. R18d shows all six selected features similarly appear in the top 20 SHAP rankings among 97 features, with values ranging from 6.50 to 1.57. Of the features excluded by RFE, the maximum mean SHAP value is 0.030 for $K_Q$ and 0.33 for HTC. This convergence between RFE selection and SHAP ranking demonstrates that permutation importance and SHAP-based pruning both confirm the six selected features as

important, validating that our feature selection is stable and not an artifact of the linear RFE step.

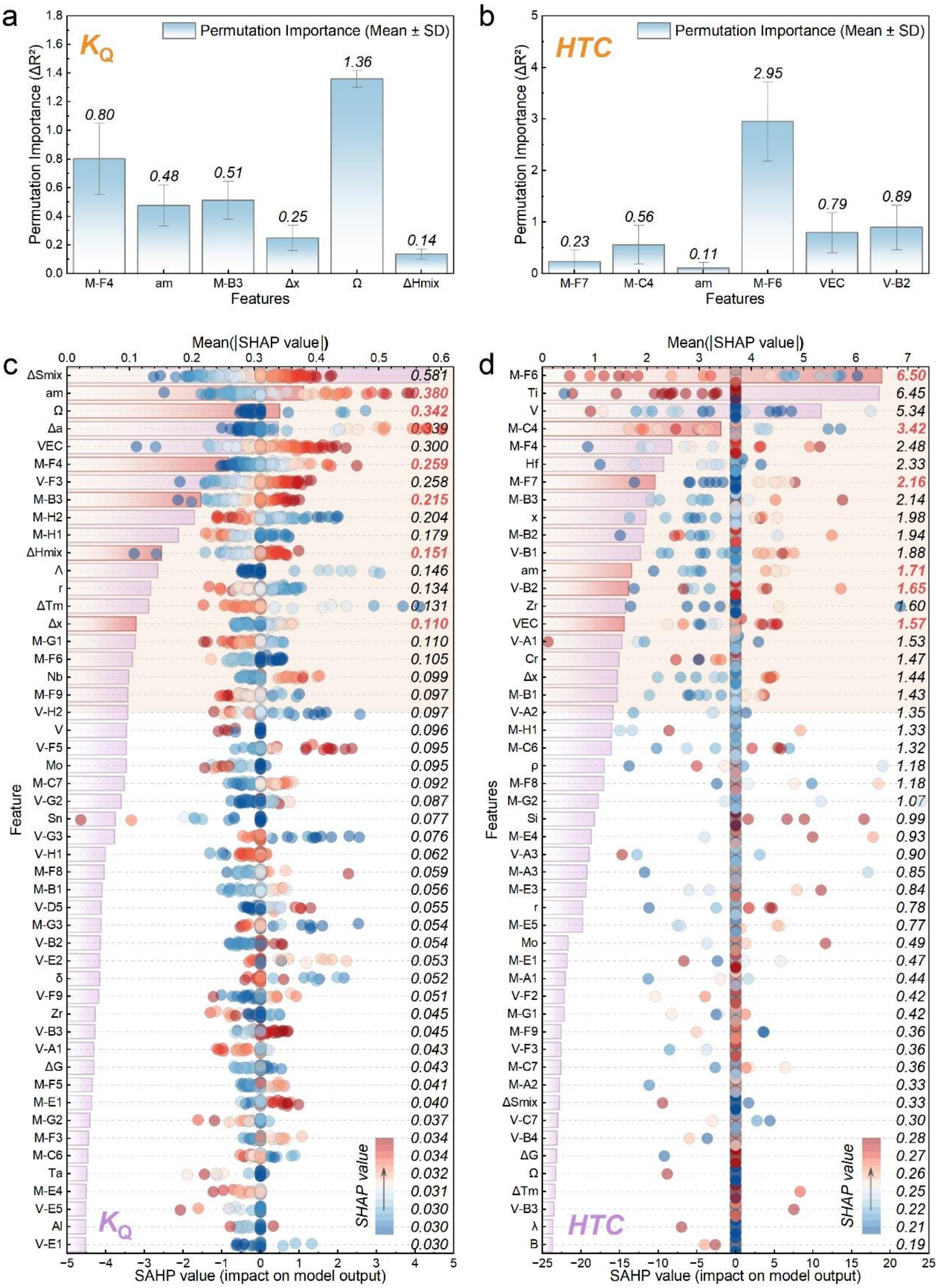


Fig. R18. Independent validation of feature selection showing (a) permutation importance for $K_Q$ model, (b) permutation importance for HTC model, (c) SHAP-based ranking on the full feature space for $K_Q$ model (top 50 features shown), and (d) SHAP-based ranking on the full feature space for HTC model (top 50 features shown).

Action taken: Added Fig. S3 and Note S2 in Supporting Information. The revisions have been added to the "Feature screening and model construction" subsection in the **Results and discussion** section.

2) Performance comparison with and without RFE.

**Response:**

We performed systematic performance comparison between models with RFE versus models without RFE to isolate the effect of this final feature selection step. Both configurations started with the same pre-filtered feature set after PCC and MIC screening, then diverged only in whether RFE was applied. All models used identical neural network architectures, training procedures, and hyperparameters to ensure fair comparison.

For the $K_Q$ model, Fig. R19a-d, e-h show training curves for the models, both models follow similar training trajectories. Performance comparison in Fig. R19i-l reveals that the with-RFE model achieves test $R^2$ of 0.96 and test RMSE of 1.50 MPa·m$^{1/2}$ compared to test $R^2$ of 0.93 and test RMSE of 1.71 MPa·m$^{1/2}$ for the without-RFE model. The with-RFE model shows improvement of 0.03 in $R^2$ and 0.21 MPa·m$^{1/2}$ in RMSE. Training time is comparable between configurations (9.06 s with RFE versus 9.94 s without RFE).

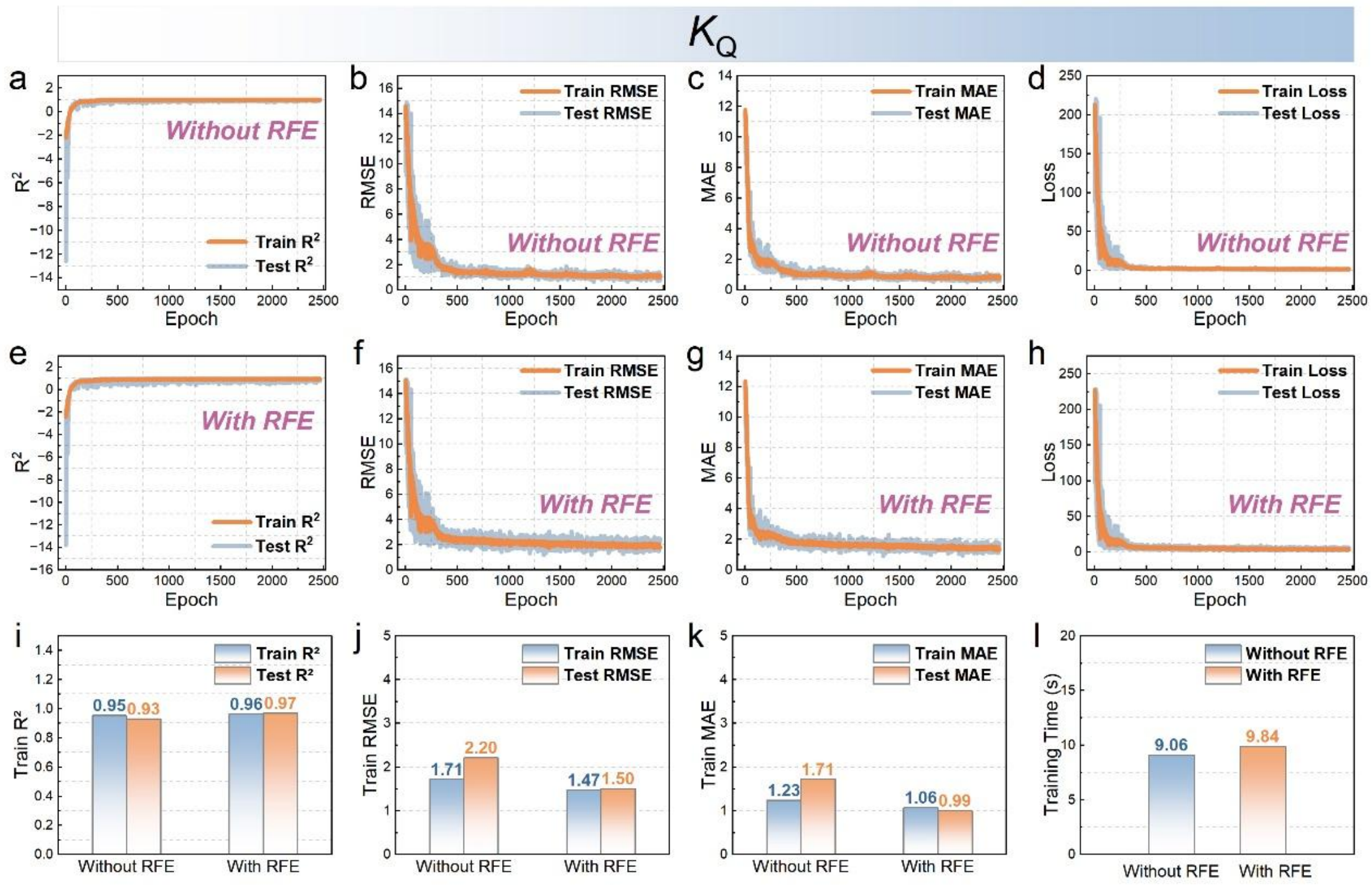


Fig. R19. Performance comparison for $K_Q$ model. (a-d) Without RFE showing $R^2$, RMSE, MAE, and Loss curves. (e-h) With RFE showing $R^2$, RMSE, MAE, and Loss curves. (i-l) Final

performance metrics comparison.

For the HTC model, performance metrics in Fig. R20i-l demonstrate that the with-RFE model achieves test $R^2$ of 0.98 and test RMSE of 3.19 compared to test $R^2$ of 0.99 and test RMSE of 3.79 for the without-RFE model. Although the without-RFE model shows slightly higher $R^2$ (0.99 vs 0.98), the with-RFE model achieves substantially better RMSE with improvement of 0.60. Training time remains comparable (1241.66 s with RFE versus 1273.09 s without RFE). These comparisons demonstrate that applying RFE as the final feature selection step maintains or improves predictive performance, validating that RFE effectively identifies the most informative features from the PCC-MIC pre-filtered set without sacrificing model accuracy.

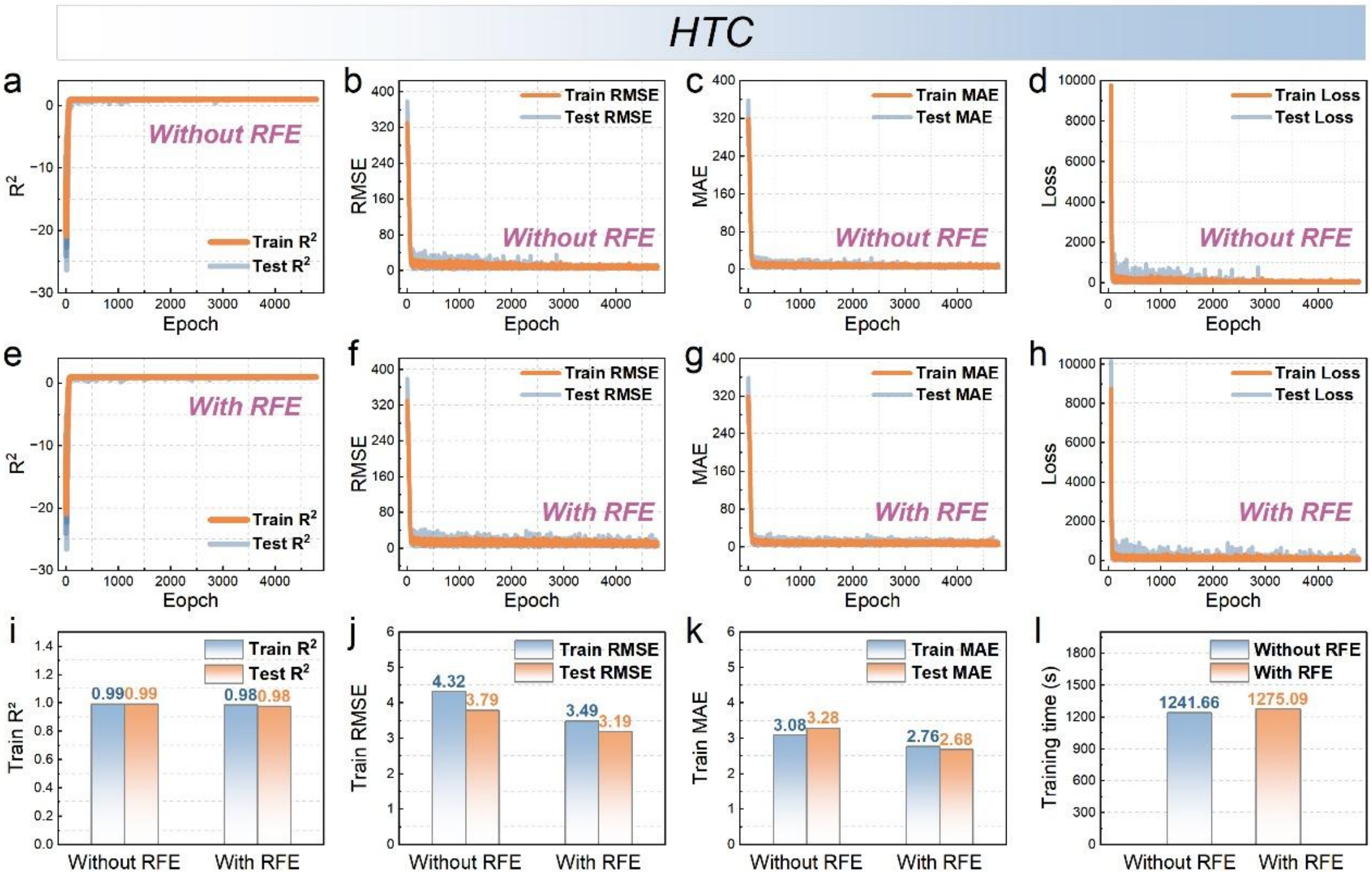


Fig. R20. Performance comparison for HTC model. (a-d) Without RFE showing $R^2$, RMSE, MAE, and Loss curves. (e-h) With RFE showing $R^2$, RMSE, MAE, and Loss curves. (i-l) Final performance metrics comparison.

Action taken: Added Figs. S4-S5 and Note S2 in Supporting Information. The revisions have been added to the "Feature screening and model construction" subsection in the **Results and discussion** section.

3) This is necessary to avoid inadvertently excluding relevant nonlinear interactions.

**Response:**

We conducted partial dependence analysis to verify that no important nonlinear interactions were excluded during feature selection. For the $K_Q$ model, one-dimensional

partial dependence analysis in Fig. R21a-f shows strong nonlinear effects for all six selected features. Fig. R21a shows M-F4 exhibits a steep increase in the 28-29 range followed by gradual decline. Fig. R21b shows am displays a sharp peak around 350-360 pm. Fig. R21c shows M-B3 demonstrates continuous increase. Fig. R21d shows Δχ has an optimal range around 15-20 with reduced toughness at extreme values. Fig. R21e shows Ω displays steady increase. Fig. R21f shows ΔHmix has optimal performance at intermediate negative values around -20 to -25 kJ/mol. These nonlinear patterns confirm the features capture complex composition-property relationships.

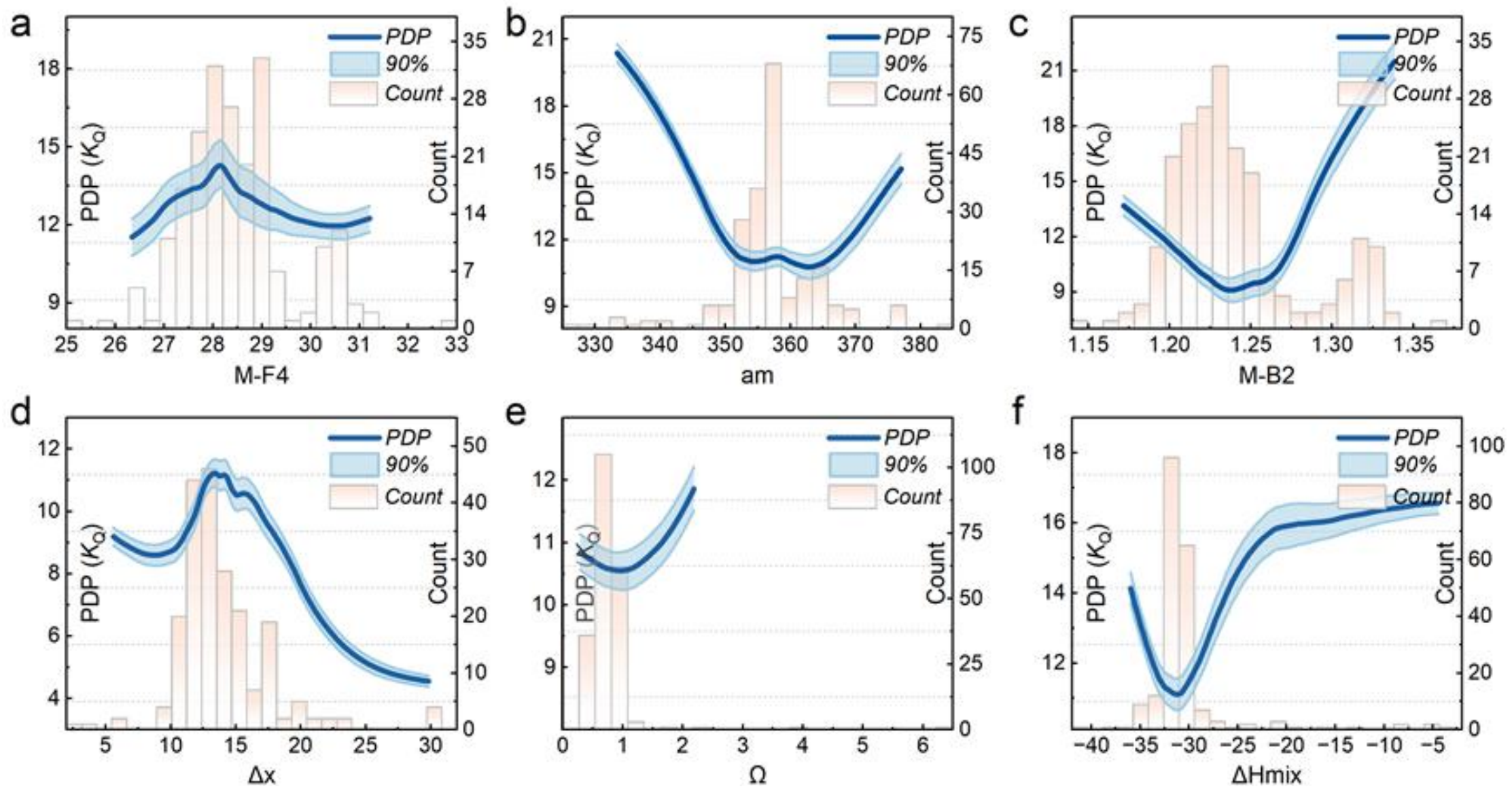


Fig. R21. One-dimensional partial dependence analysis for $K_Q$ model. (a-f) PDP curves for six selected features. (a) M-F4; (b) am; (c) M-B2; (d) Δχ; (e) Ω; (f) ΔHmix.

Gradient analysis in Fig. R22a-f identifies regions where each feature has positive versus negative effects, with M-F4 showing strong positive gradients above 28.5 (Fig. R22a), am showing positive effects in 350-365 pm range (Fig. R22b), M-B3 exhibiting predominantly positive gradients (Fig. R22c), Δχ showing positive effects in 10-20 range (Fig. R22d), Ω displaying consistent positive gradients (Fig. R22e), and ΔHmix showing positive gradients in -15 to -25 kJ/mol range (Fig. R22f).

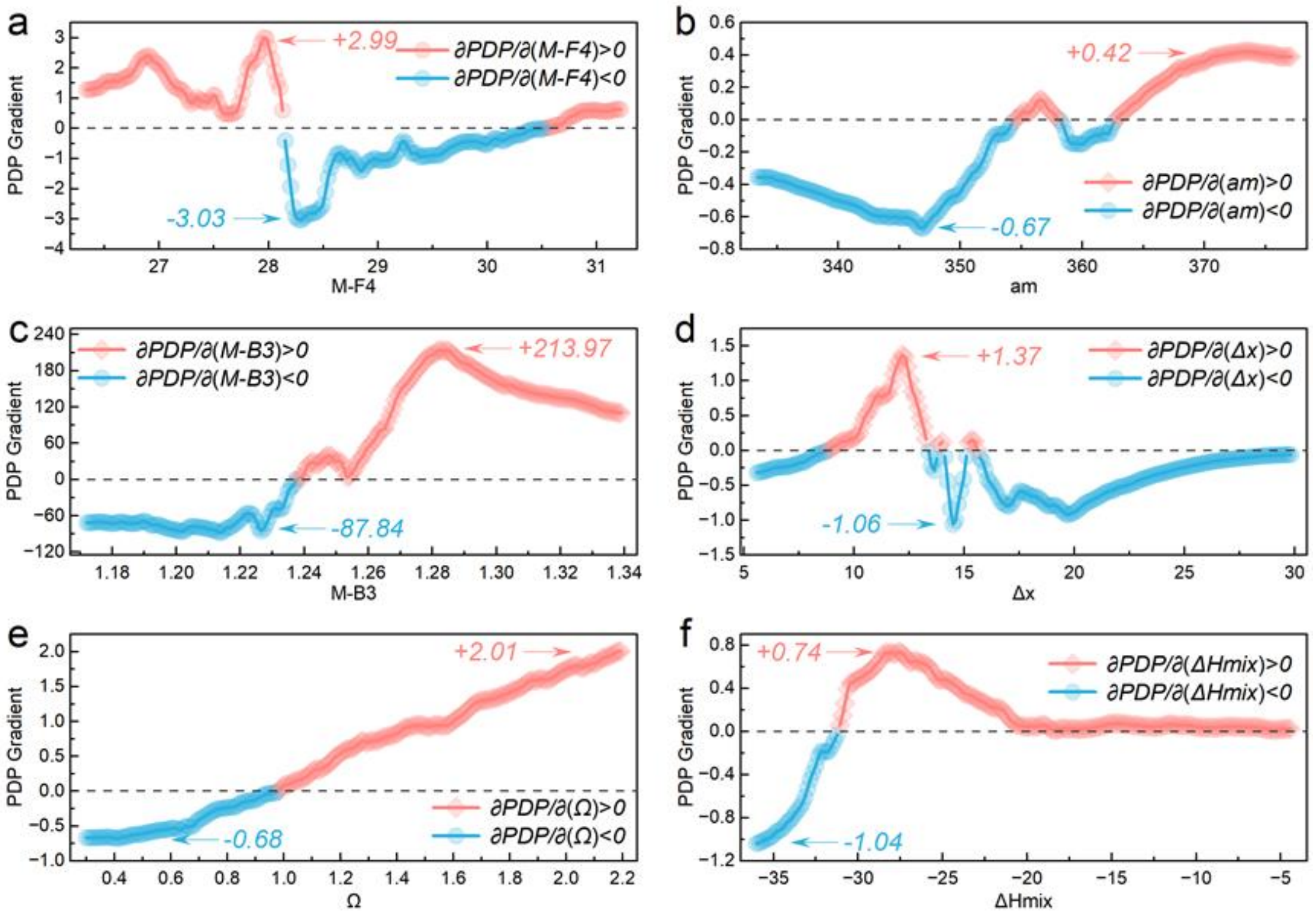


Fig. R22. Partial dependence gradient analysis for $K_Q$ model. (a-f) Gradient plots showing regions where each feature has positive (red) versus negative (blue) effects on $K_Q$ prediction. (a) M-F4; (b) am; (c) M-B2; (d) $\Delta\chi$; (e) $\Omega$; (f) ΔHmix.

Two-dimensional partial dependence analysis in Fig. R23a-o reveals pairwise feature interactions across all 15 possible combinations of the six selected features. Non-rectangular contour patterns indicate genuine multiplicative interactions rather than simple additive effects. Fig. R23b shows M-F4 versus M-B3 interaction with a diagonal red zone where high toughness (21-24 MPa·$m^{1/2}$) requires both M-F4 around 28-29 and M-B3 above 1.26, demonstrating neither feature alone determines toughness. Fig. R23f shows am versus M-B3 interaction revealing peak toughness (22-25 MPa·$m^{1/2}$) only when am is 350-365 pm combined with M-B3 of 1.28-1.32. Fig. R23k shows M-B3 versus $\Omega$ interaction with curved boundaries confirming multiplicative effects where maximum toughness (21 MPa·$m^{1/2}$) requires $\Omega$ of 1.24-1.32 with M-B3 above 1.6. Fig. R23e shows M-F4 versus ΔHmix interaction demonstrating that intermediate ΔHmix values of -15 to -20 kJ/mol combined with M-F4 around 29 produce high toughness (17-19 MPa·$m^{1/2}$), while extreme values yield low toughness. The smooth continuous boundaries throughout Fig. R23a-o indicate genuine physical interactions rather than random noise.

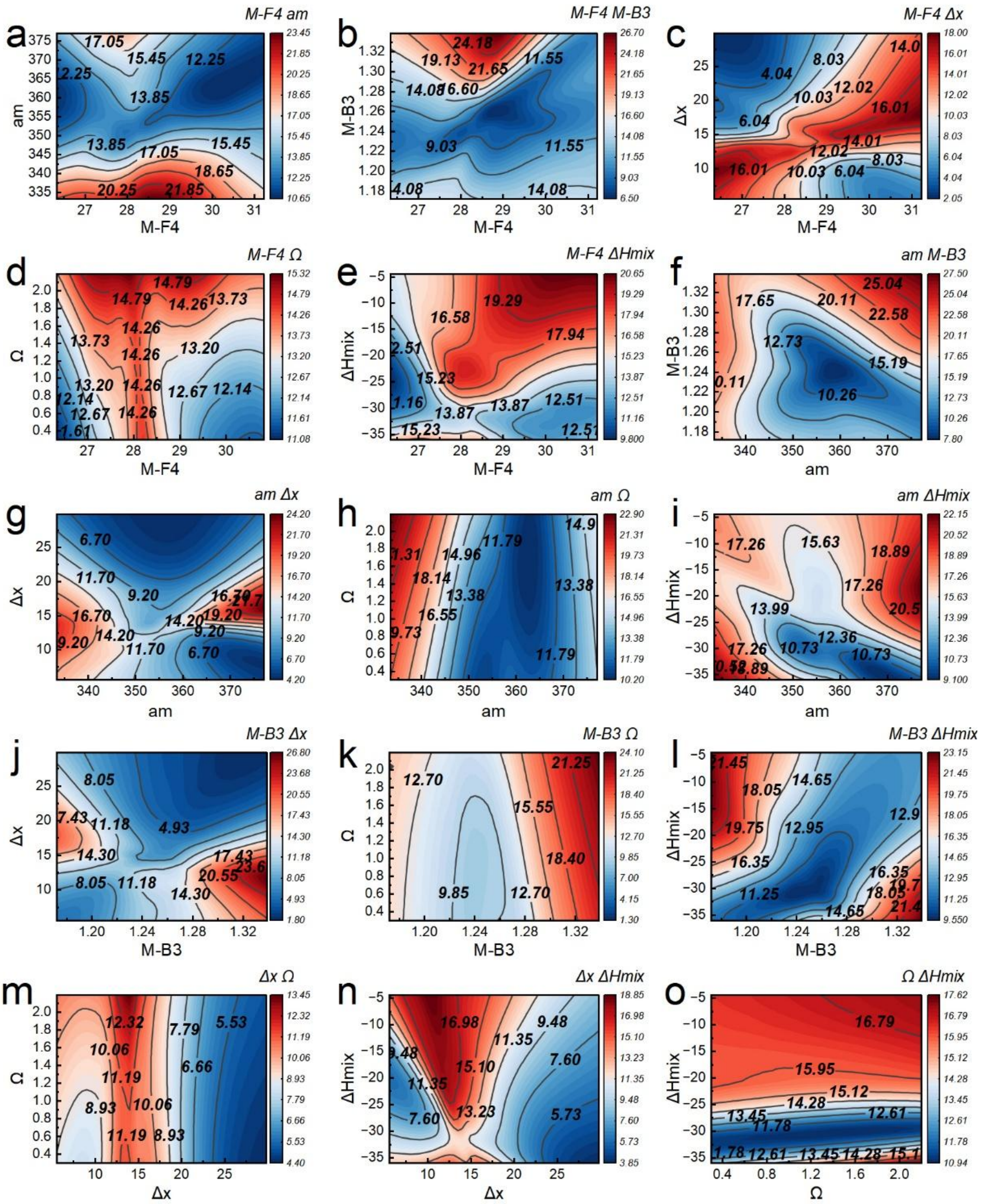


Fig. R23. Two-dimensional partial dependence interaction analysis for $K_Q$ model showing all 15 pairwise combinations:(a) M-F4 - am; (b) M-F4 - M-B3; (c) M-F4 - Δχ; (d) M-F4 - Ω; (e) M-F4 - ΔHmix; (f) am - M-B3; (g) am - Δχ; (h) am - Ω; (i) am - ΔHmix; (j) M-B3 - Δχ; (k) M-B3 - Ω; (l) M-B3 - ΔHmix; (m) Δχ - Ω; (n) Δχ - ΔHmix; (o) Ω - Δhmix.

To further quantify these interaction patterns, we applied Shapley Interaction Index (SII) analysis using the shapiq framework. Fig. R24a shows the strongest antagonistic interaction is between M-F4 and M-B3 (SII = -3.22), consistent with the constrained high-toughness region in the corresponding 2D PDP contour, while Δχ and M-B3 exhibit the largest synergistic interaction (SII = +1.37). Fig. R24b shows the

composition-sensitivity of each interaction, with M-F4 and M-B3 showing the largest standard deviation (Std = 3.74). Fig. R24c confirms that the interactions are concentrated in a physically interpretable subset of feature pairs. The 2D PDP and SII analyses across all 15 feature pairs together confirm that the RFE-selected features retain all pairwise nonlinear interactions present in the full feature space, with no relevant interactions inadvertently excluded.

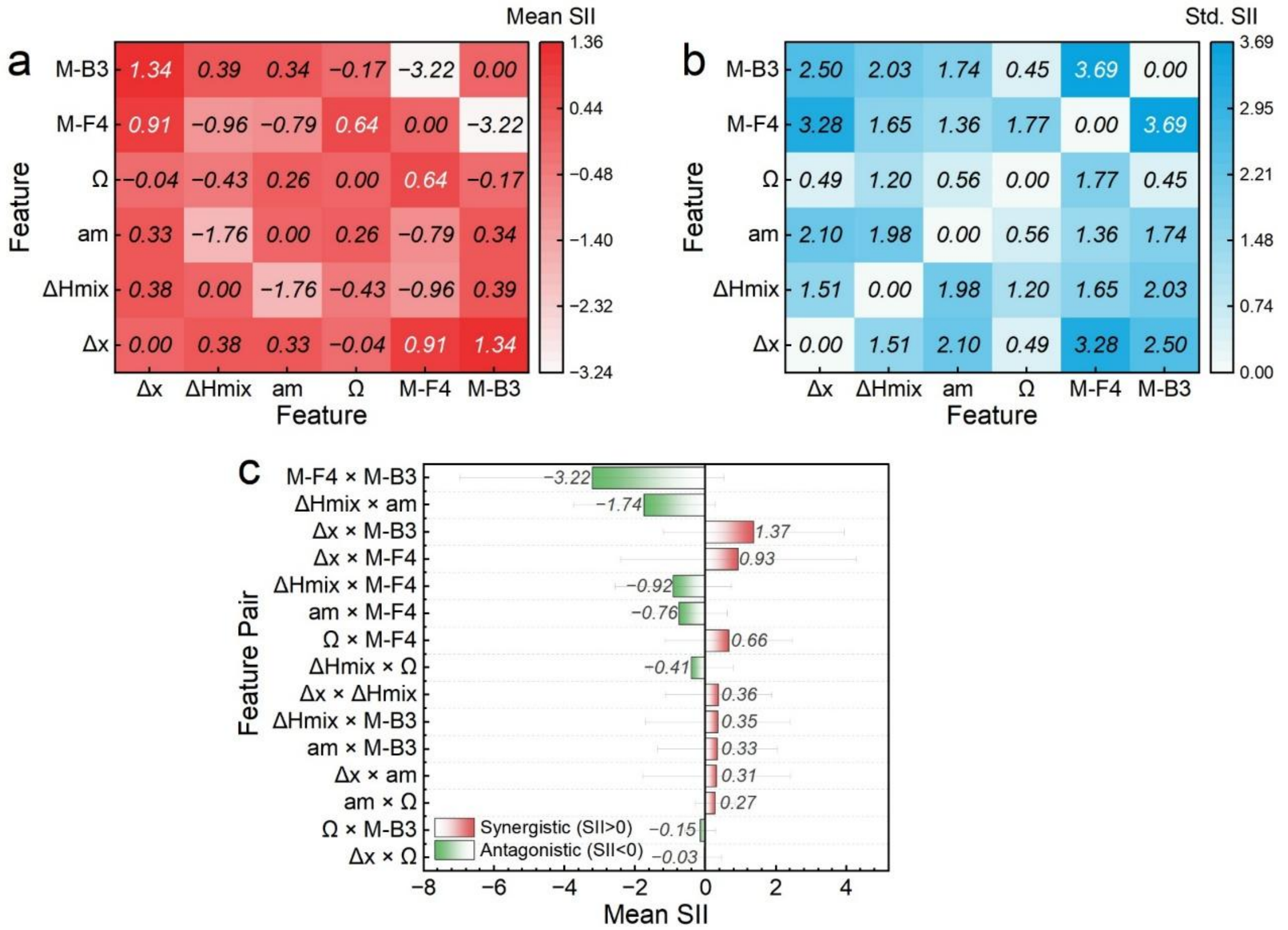


Fig. R24. Shapley Interaction Index analysis for the $K_Q$ model. (a) Mean SII matrix, where color intensity reflects the magnitude of interaction strength and the sign of each value indicates synergistic (positive) or antagonistic (negative) interactions. (b) Standard deviation of SII across samples. (c) All 15 feature pairs ranked by absolute mean SII with standard deviation shown as error bars.

Action taken: Added Figs. S27-S30 in Supporting Information. The revisions have been added to the "Connecting ML-identified features to microstructural mechanisms" subsection in the **Results and discussion** section.

Comment 6: Model extrapolation claims need stronger support: Some optimized alloys fall outside the training distribution in the t-SNE space. While experimental validation is encouraging, the manuscript should provide a more quantitative measure of how far these points lie from the data manifold.

Requested:

1) Mahalanobis distance, density-based distance, or similar metric to assess extrapolation.

**Response:**

We implemented three distance metrics to quantify extrapolation risk. (I) Mahalanobis distance accounts for both variance and correlations among compositional features. (II) Nearest-neighbor distance identifies compositional proximity to existing training data. (III) Local density quantifies how well-populated the compositional space is around each prediction.

For the $K_Q$ dataset, Fig. R25a shows the Mahalanobis distance distribution, with risk zones defined as low ($D_M$ < 2.26, median), medium (2.26-6.27, 95th percentile), and high (> 6.27, beyond 95th percentile). Our four optimized alloys show distinct positions: alloys #2 and #3 fall in the low-risk zone with $D_M$ = 1.89 and 2.01 respectively, while alloys #1 and #4 occupy the medium-risk zone with $D_M$ = 3.45 and 4.12. Fig. R25b displays nearest-neighbor distances, revealing that alloy #3 has exceptional compositional proximity to training data with nearest-neighbor distance of only 2.1 (differing by ~2 at.% in major elements), while alloys #1, #2, and #4 show distances of 3.8, 3.2, and 4.5 respectively. Fig. R25c presents local density analysis using a search radius of 2.5σ (where σ is the average standard deviation across compositional dimensions), showing alloys #2 and #3 surrounded by 95 and 90 training neighbors respectively, while alloys #1 and #4 have 36 and 42 neighbors. Fig. R25d visualizes the combined metrics through a scatter plot of Mahalanobis distance versus local density, clearly showing alloys #2 and #3 embedded within the main training cluster (low $D_M$, high density) and alloys #1 and #4 in moderately explored regions.

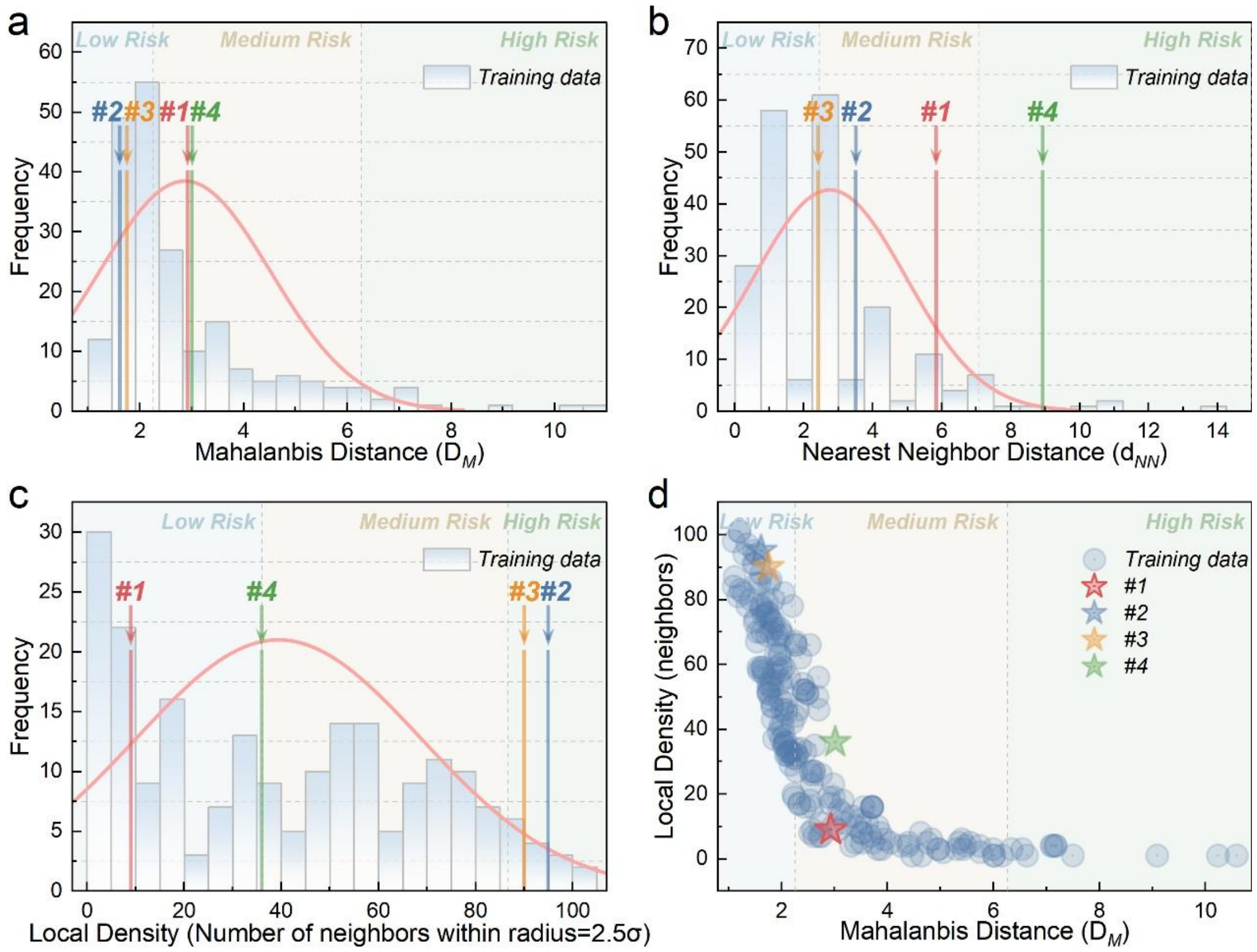


Fig. R25. Distance metric analysis for $K_Q$ predictions. (a) Mahalanobis distance distribution with risk zones and optimized alloy positions, (b) nearest-neighbor distance distribution, (c) local density distribution using 2.5σ radius, and (d) scatter plot of Mahalanobis distance versus local density.

The HTC dataset with 34 samples across 11 dimensions shows different extrapolation behavior. Fig. R26a shows training sample Mahalanobis distances range from 1.18 to 5.46 with median 2.48 and 95th percentile 4.58. Our optimized alloys display $D_M$ of 11.53 (#1), 19.23 (#2), 14.86 (#3), and 37.23 (#4) exceeding the training maximum by factors of 2.1 to 6.8. All predictions fall into the high-risk extrapolation zone. Fig. R26b shows nearest-neighbor distances of 4.96 to 14.11 placing alloys at or above the 88th percentile of training distribution. Fig. R26c shows all four alloys display zero local density confirming complete isolation from training samples. Fig. R26d shows all alloys clustered in the unsupported region ($D_M$ > 11, density = 0), contrasting sharply with the training distribution.

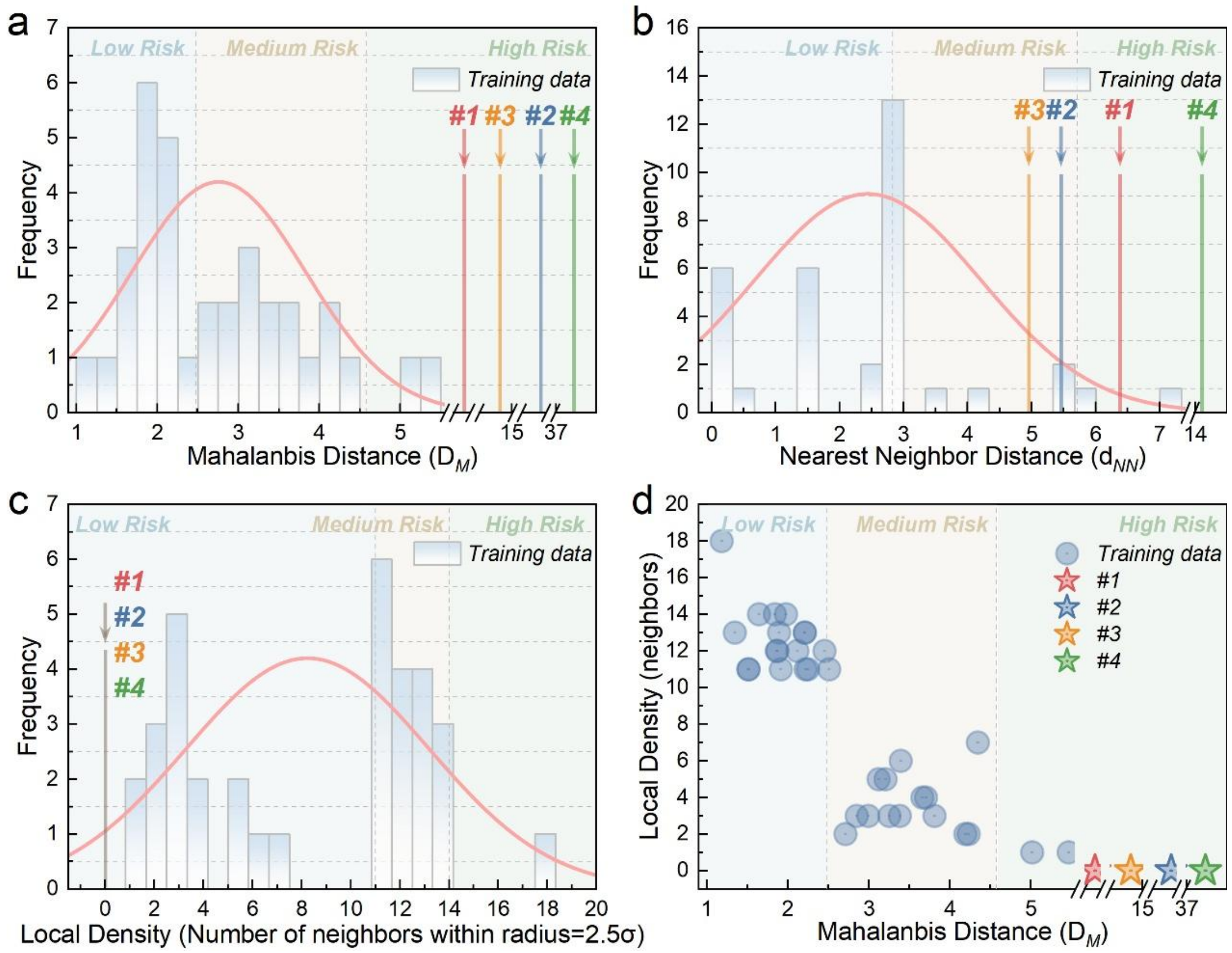


Fig. R26. Distance metric analysis for HTC predictions. (a) Mahalanobis distance distribution with all optimized alloys beyond training range, (b) nearest-neighbor distance distribution, (c) local density distribution showing zero density for all alloys, and (d) scatter plot showing alloys in unsupported region.

Action taken: Add Figs. S31-S32 and Note S6 in Supporting Information. The revisions have been added to the "Connecting ML-identified features to microstructural mechanisms" subsection in the **Results and discussion** section.

2) Clear discussion of the risks and limits of extrapolating beyond the training space.

**Response:**

We integrated a discussion of extrapolation risks and limitations into the manuscript. Prediction reliability degrades with compositional distance from training data. Sparse training coverage may miss complex composition-property relationships particularly nonlinear interactions. For HTC the sample-to-dimension ratio of 3.1 falls below typical requirements for capturing high-dimensional relationships. Standard confidence intervals fail to account for compositional distance. A prediction with zero local density carries higher uncertainty than one surrounded by numerous training examples. Extrapolative compositions may encounter unforeseen failure modes including phase

instabilities or brittle fracture mechanisms in unexplored regions.

Experimental validation demonstrated successful predictions despite these limitations. For $K_Q$ predictions in well-supported regions with Mahalanobis distance below 3 and local density above 36 as shown in Fig. R25d, experimental measurements matched predictions within 5% error. Alloys #2 and #3 with $D_M$ of 1.61 and 1.75 and local densities of 95 and 90 neighbors showed excellent agreement. Even alloys #1 and #4 in moderately sparse regions with $D_M$ of 2.93 and 3.01 showed reasonable agreement. For HTC, experimental validation proved critical given the severe extrapolation visible in Fig. R26. All four predictions despite Mahalanobis distances of 11.53, 19.23, 14.86, and 37.23 (exceeding training maximum by factors of 2.1 to 6.8) and zero local density yielded experimental results within physically reasonable ranges. While prediction errors were larger than for $K_Q$ as expected for extrapolation, the model avoided catastrophic failures.

For $K_Q$ with sample-to-dimension ratio of 17, models can reliably predict within and moderately beyond the training distribution as Fig. R25 confirms. Such models are suitable for computational screening particularly for compositions in the median to 95th percentile distance range. For HTC with sample-to-dimension ratio of 3.1, predictions serve as hypotheses requiring experimental validation as Fig. R26 illustrates with all four alloys showing $D_M$ exceeding training range and zero local density. Experimental confirmation becomes mandatory in such scenarios. The quantitative distance metrics in Figs. R25-R26 provide clear assessment replacing qualitative t-SNE observations with rigorous measurements.

Action taken: Add Note S6 in Supporting Information. The revisions have been added to the "Connecting ML-identified features to microstructural mechanisms" subsection in the **Results and discussion** section.

Minor Points:

Some figures remain overly dense. Consider a consolidated workflow figure for clarity.

**Response:**

We enhanced Fig. R27 to provide a comprehensive workflow and restructured Fig. R28 to focus on TEM analysis.

**(I) Consolidated workflow figure**

The revised Figure 1 now explicitly shows data collection from literature sources, three-step feature screening (PCC-MIC-RFE), artificial neural network training

architecture, SHAP analysis for interpretability, multi-objective optimization using NSGA-II, and experimental validation procedures. Compared to the previous version, the enhanced Fig. R27 provides specific algorithmic details making it easier for readers to understand and reproduce our methodology. The hierarchical visual organization with clear process blocks and connecting arrows maintains readability while adding detail.

[Figure Redacted]

Fig. R27. ML based design process flow chart for Nb-Si alloys.

**(II) Figure density reduction**

We restructured Fig. R28 to focus exclusively on TEM analysis of alloy #1. Fig. R28a shows high-density dislocation walls in Nbss matrix. Fig. R28b shows bright field image with dispersed γ'-$Nb_5Si_3$ nanoparticles averaging 94.88 nm. Fig. R28b2-b3 show selected area electron diffraction patterns. Fig. R28c displays high-resolution TEM image of γ' phase particle. Fig. R28c1-c9 provide EDS element mapping. Fig. R28d shows phase interface region analysis with corresponding electron diffraction patterns from γ' phase, interface, and Nbss matrix regions. Fig. R28e1-e6 show inverse fast Fourier transform analysis revealing atomic arrangements and lattice spacings. Fig. R28f1-f2 show geometric phase analysis with strain distribution at interfaces revealing 6-8% strain gradient. Original Fig. R28g-l were transferred to Supporting Information. These revisions address figure density concerns by consolidating workflow details in Fig. R27 and streamlining TEM analysis in Fig. R28.

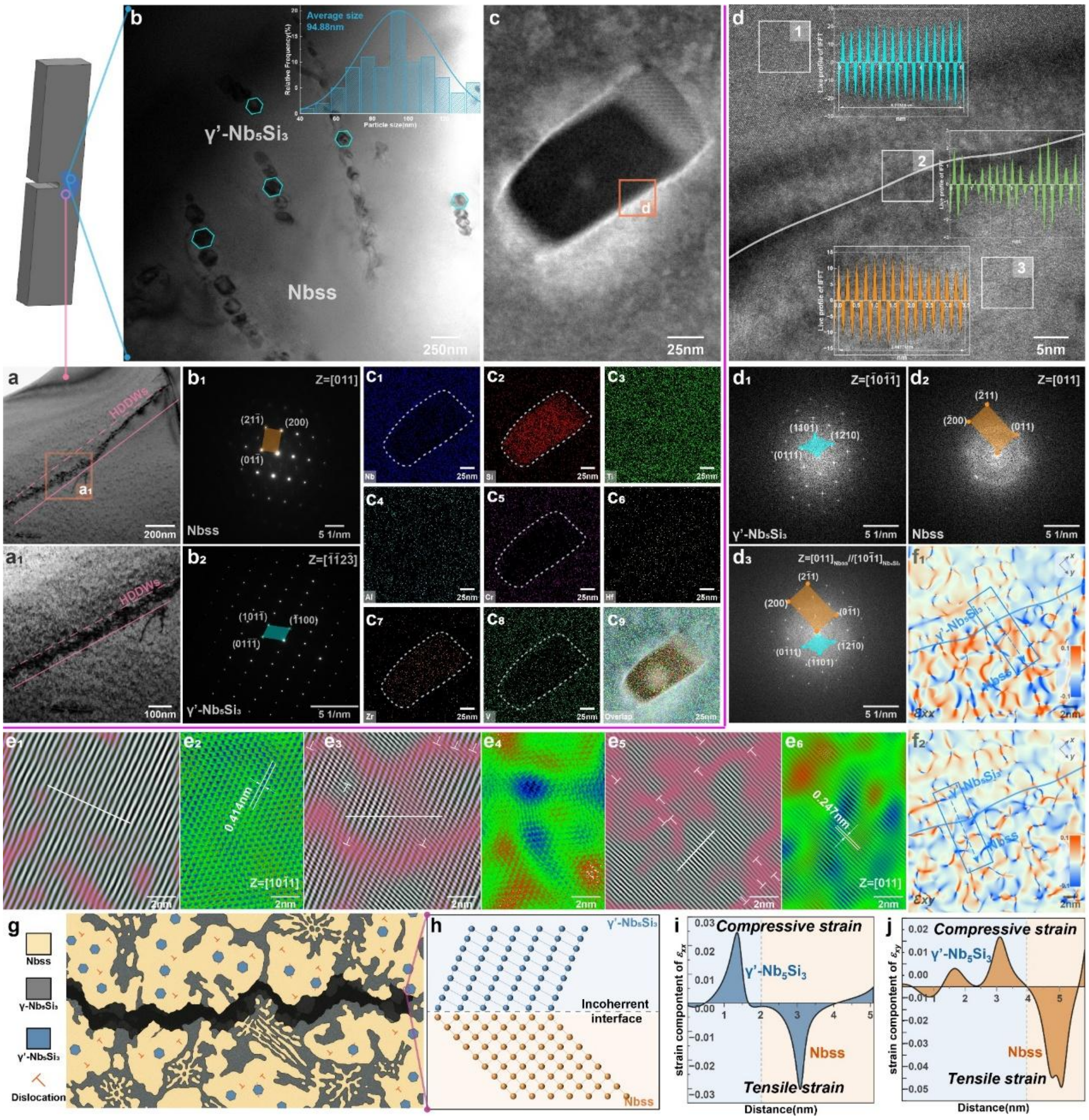

has been changed to

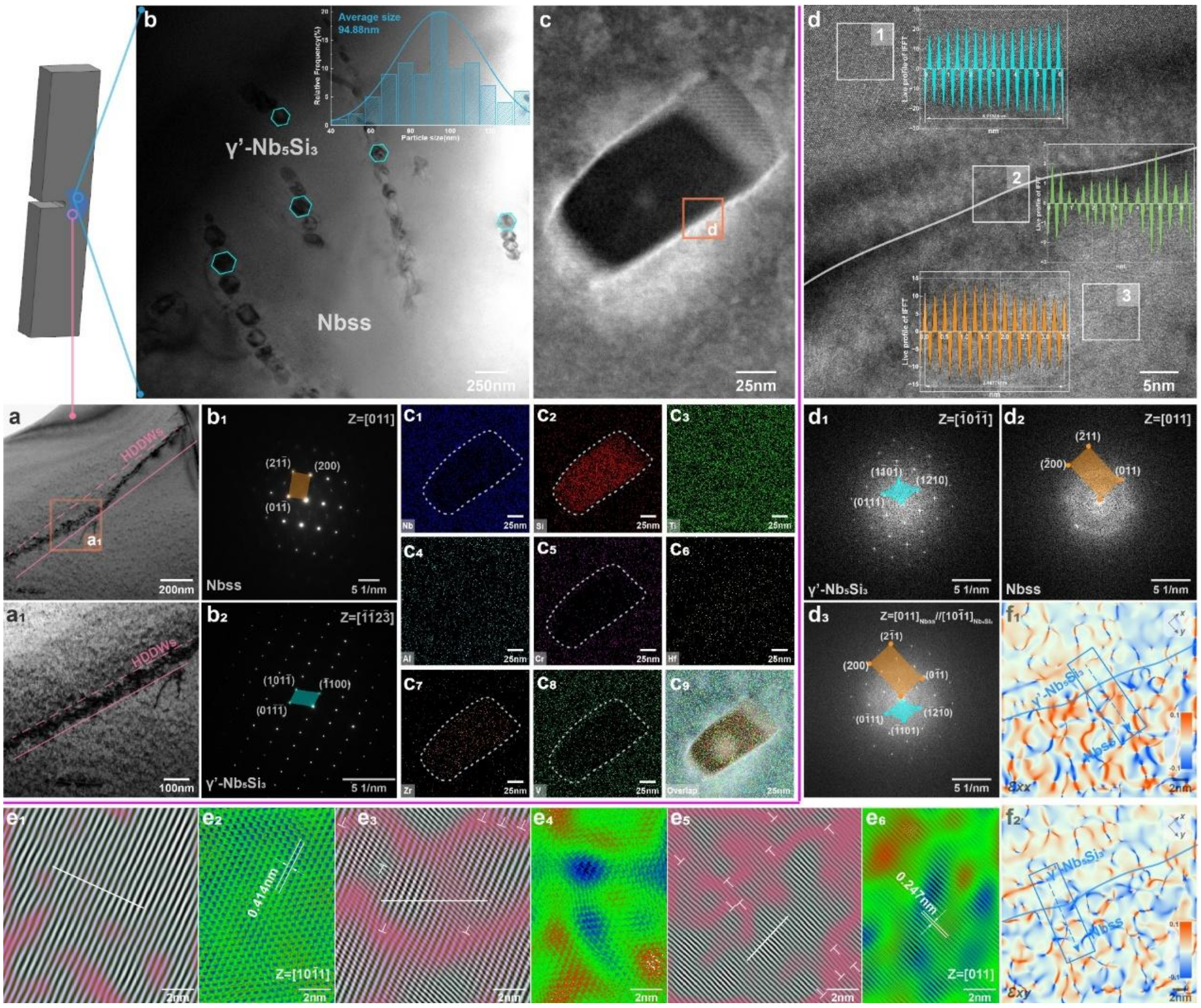


Fig. R28. TEM analysis of alloy #1 revealing nanoscale strengthening mechanisms. (a and a1) High-density dislocation walls in Nbss matrix. (b) Bright field image showing dispersed γ'-$Nb_5Si_3$ nanoparticles (average size 94.88 nm). ($b_2$ and $b_3$) Selected area electron diffraction patterns. (c) High-resolution TEM image of γ' phase particle. ($c_1$ to $c_9$) EDS element mapping of γ' phase. (d) Phase interface region analysis with corresponding electron diffraction patterns ($d_1$ to $d_3$) from γ' phase, interface, and Nbss matrix regions. ($e_1$ to $e_6$) Inverse fast Fourier transform analysis revealing atomic arrangements and lattice spacings. ($f_1$ and $f_2$) Geometric phase analysis showing strain distribution at interfaces.

Check consistency of numerical values, units, and thresholds introduced in the supplementary materials.

**Response:**

We have carefully checked all numerical values, units, and thresholds in both the main text and Supporting Information. We verified over 700 instances across 15 categories to ensure complete consistency. The detailed results are as follows:

**(I) Unit Standardization (8 items):**

(1) All 53 instances consistently use "MPa·m$^{1/2}$" format for fracture toughness (18 in main text, 35 in Supporting Information).

(2) All 13 instances consistently use "at.%" format for composition (8 in main text, 5 in Supporting Information).

(3) All 70 instances use correct units for strength values. "MPa" is used for compressive strength, and "GPa" is used for elastic modulus (3 instances in Supporting Information).

(4) All 31 instances use correct formats for temperature. "°C" is used for test temperatures (11 instances), and "K" is used for cooling rates and thermodynamic calculations (20 instances).

(5) All 30 instances use appropriate units for size measurements at different scales: "µm" for grain and particle sizes (10 instances), "nm" for nanoscale precipitates (8 instances), and "mm" for specimen dimensions (12 instances).

(6) All 75 instances use "MPa" or "Pa" format for pressure. One minor correction was made: "15 pa" has been changed to "15 Pa" in Supporting Information (Supplementary Note S4).

(7) All 4 instances consistently use "kJ/mol" format for enthalpy values.

(8) All 18 instances consistently use "g" for ingot mass.

**(II) Numerical Value Clarification (2 items):**

(9) We have added explicit train-test split information for sample sizes at multiple locations: In the Results section, "yielding 203 samples for $K_Q$ prediction and 34 samples for HTC prediction" has been changed to "yielding 203 total samples (163 training, 40 test) for $K_Q$ prediction and 34 total samples (27 training, 7 test) for HTC prediction". The same specification was added in the Methods section. In the cross-validation discussion, "For the $K_Q$ model (203 samples)" has been changed to "For the $K_Q$ model (203 total samples: 163 training, 40 test)", and "For the HTC model (34 samples)" has been changed to "For the HTC model (34 total samples: 27 training, 7 test)".

(10) We have added explicit dataset split ratios in the Methods section: "The $K_Q$ dataset was split into training and test sets with a ratio of 80.3%/19.7% (163/40 samples), while the HTC dataset used a 79.4%/20.6% ratio (27/7 samples)."

**(III) Error Representation (1 item):**

(11) All 25 instances follow standardized formats for error notation (13 in main text, 12 in Supporting Information): "value±error" format for means and standard deviations (8 instances), "±error" format for confidence intervals (13 instances), and "SE=value" format for standard errors (4 instances).

**(IV) Threshold Consistency (3 items):**

(12) Feature selection thresholds are consistent throughout the manuscript: $K_Q$ model uses PCC > 0.2 and MIC > 0.2 (Results section, Methods section, and Supplementary Note S1), while HTC model uses PCC > 0.09 and MIC > 0.08 (Results section, Methods section, and Supplementary Note S2).

(13) All model performance metrics are identical in main text and Supporting Information. For the $K_Q$ model: $R^2 = 0.98$ (training), $R^2 = 0.96$ (test), RMSE = 0.88 MPa·m$^{1/2}$, MAE = 0.61 MPa·m$^{1/2}$. For the HTC model: $R^2 = 0.89 \pm 0.01$, RMSE = 4.4±0.3 MPa, MAE = 3.4±0.2 MPa. These values appear in Results section, Methods section, and relevant Supplementary Notes.

(14) All 22 instances of statistical significance thresholds use standard formats consistently: $p < 0.001$ for highly significant correlations (8 instances), $p < 0.05$ for significant correlations (6 instances), and specific p-values for normality tests ($p=0.1573$ for training set, $p=0.127$ for test set). All thresholds are consistent across main text and Supporting Information.

**(V) Threshold Consistency (3 items):**

(15) All 171 instances of numerical ranges use clear and consistent formats (65 in main text, 106 in Supporting Information). Both "X-Y" notation (e.g., "7-20 MPa·m1/2") and "from X to Y" notation are used appropriately.

(16) All 124 instances of percentage values consistently use "X%" format (49 in main text, 75 in Supporting Information) for sample distributions, coverage rates, and performance metrics.

Ensure that all updated figures include clear legends, axis labels, and color descriptions.

**Response:**

We have carefully verified and improved all figures in both the main text and Supporting Information. The detailed verification and improvement results are as follows:

**(I) Main Text Figures (7 figures verified):**

(1) All 7 main text figures include complete axis labels with units. For example, Fig. 3 uses "Experimental $K_Q$(MPa·m$^{1/2}$)" and "Predicted $K_Q$(MPa·m$^{1/2}$)" with explicit unit specifications.

(2) All figures with color-coded data include clear legends. Fig. 3 displays "● Train ● Test" with blue for training data and red for test data. Fig. 4 includes color bars labeled "high" to "low" for SHAP heatmaps, and "Positive SHAP"/"Negative SHAP" for

contribution plots.

(3) All multi-panel figures (Fig. 2-7) include clear panel labels (a, b, c, etc.). Fig. 2 contains 6 panels (a-f) covering correlation heatmaps, PCC-MIC analysis, and RFE results.

(4) All heatmaps include color bars with value ranges. Fig. 2a-b show correlation values from -1.0 to 1.0. Fig. 4c-d use gradient coloring from green (high) to pink (low) for SHAP values.

(5) All microscopy images (Fig. 5-7) include scale bars with units. Fig. 6 contains TEM images with scale bars from 5nm to 250nm. Fig. 7 contains EBSD images with scale bars of 20μm and 200μm.

(6) All statistical plots include complete metric labels. Fig. 3a-b display $R^2$, MAE, and RMSE values with training (blue text) and test (red text) distinction. Fig. 2e-f show dual y-axes: "$R^2$" (left, red) and "RMSE" (right, cyan).

(7) All phase distribution maps include percentages. Fig. 7b shows "Nbss:67.82%" and "γ-$Nb_5Si_3$:30.41%". Fig. 5j displays element distribution maps (Nb, Si, Ti, Cr, Al, Hf, Zr, V) with distinct colors.

(8) All optimization diagrams include complete information. Fig. 5a (Pareto front) includes axis labels "Predicted $K_Q$ (MPa·$m^{1/2}$)" and "Predicted HTC (MPa)", with legend distinguishing "target", "Pareto frontier", and "Other solutions".

**(II) Main Text Figures (7 figures verified):**

(9) All supplementary figures include comprehensive captions with panel labels. Supplementary Fig. 1 (element co-occurrence network and heatmap) labels all panels (a-d) with color bars showing element frequencies from "High" to "low".

(10) Distribution plots (Supplementary Fig. 2, 10, 11) have been improved. Supplementary Fig. 2 now includes x-axis labels "$K_Q$ (MPa·$m^{1/2}$)" and "HTC (MPa)". Supplementary Fig. 10-11 now include in-figure legends "● Training ● Testing" (blue and orange).

(11) All training curves (Supplementary Fig. 4-6, 35) include complete labels. Supplementary Fig. 35 legends now indicate uncertainty: "Train RMSE (±std)" and "Val RMSE (±std)", clarifying that shaded regions represent standard deviation.

(12) Grid search plots (Supplementary Fig. 8-9) have been improved. Supplementary Fig. 8 now includes color bars with explicit "Train" and "Test" labels for each metric ($R^2$, RMSE, MAE).

(13) Optimization results (Supplementary Fig. 7, 16-17) include axis labels "Predicted $K_Q$ (MPa·$m^{1/2}$)" vs "Predicted HTC (MPa)" with legends distinguishing "NSGA-II",

"NSGA-II Pareto", "Random", and "target" solutions.

(14) Residual analysis plots (Supplementary Fig. 12) include axis labels and statistical annotations: "Residuals (MPa)" vs "Predicted HTC (MPa)" with ±2σ bounds, Q-Q plots with "Theoretical Quantiles" vs "Sample Quantiles", and autocorrelation functions with 95% confidence intervals and p-values.

(15) SHAP analysis figures (Supplementary Fig. 3, 13-15) include "SHAP value" on y-axis with normalized feature values on x-axis. Color bars use gradient coloring (green for positive/high, pink for negative/low), with correlation coefficients (K values) labeled on each panel.

(16) Partial dependence plots (Supplementary Fig. 27-28) include left y-axis "PDP ($K_Q$)" and right y-axis "Count". Contour plots show interaction effects with color-coded intensity (red-blue gradient) and numerical values on contour lines.

(17) Microstructural characterization figures (Supplementary Fig. 21-23) have been enhanced. Supplementary Fig. 22 (IPF maps) now includes standard IPF color triangles for both Nbss (BCC) and $Nb_5Si_3$ phases. All panels include scale bars (100μm, 10μm) and phase labels with volume percentages.

(18) Mechanical property plots (Supplementary Fig. 19-20, 24-25) include axis labels with units and legends for samples (#1-4). Nanoindentation results show "Loading (mN)" vs "Displacement (nm)" for both Nbss and $Nb_5Si_3$ phases.

(19) Correlation plots (Supplementary Fig. 26, 30) include axis labels with units, trend lines, 95% confidence intervals (shaded regions), correlation coefficients (r values), and p-values on each panel.

(20) Applicability domain analysis (Supplementary Fig. 31-32) includes axis labels (Mahalanobis Distance, Nearest Neighbor Distance, Local Density), risk zone labels (Low/Medium/High Risk), and optimized alloys (#1-4) marked with colored stars.

(21) Uncertainty quantification plots (Supplementary Fig. 33-35) include predicted values, actual values, 95% confidence intervals (error bars and shaded regions), and uncertainty distributions. Training and test datasets use consistent blue and red/pink colors, with RMSE and MAE values labeled.

Reviewer #1 (Remarks on code availability):

Am I supposed to install and run this code?

**Response:**

No, the Reviewer is not required to install or run the code for review purposes. The code is available at https://github.com/chaoxu-HIT/report-code and is provided

primarily for transparency and post-publication reproducibility. We have uploaded all Python code with complete implementation details and all execution results. The repository includes all datasets, computed features, trained model checkpoints, and prediction outputs in easily accessible formats (CSV, Excel). The pre-computed results can be directly examined without any installation. The code repository serves as a resource for researchers who may want to reproduce our work or apply our methodology to their own data after publication.

Action taken: Confirmed code repository includes all implementation code, execution results, datasets, and pre-computed outputs accessible without installation. Updated README to clarify code execution is not required for peer review.

Reviewer #2 (Remarks to the Author):

The quality of the revised manuscript has improved. However, the following issues remain.

**Response:**

We thank the reviewer for the positive assessment of our revisions and for identifying these remaining issues. We have now addressed all three concerns with detailed analysis and clarification.

1. The previous review noted that the two datasets are reported as having 203 and 34 samples, which are inconsistent with the counts shown in Figure 3c and those in Figures 3b and 3d. The response addressed only the case with 34 samples, but not the case with 203 samples. Please also clarify the inconsistency for the dataset with 203 samples.

**Response:**

We examined the dataset in Fig. R29 and confirmed that Fig. R29c contains 203 samples. To facilitate reader comprehension and improve figure readability, we revised Fig. R29c by adding clear sample count annotations (training set n=163, test set n=40), as shown below.

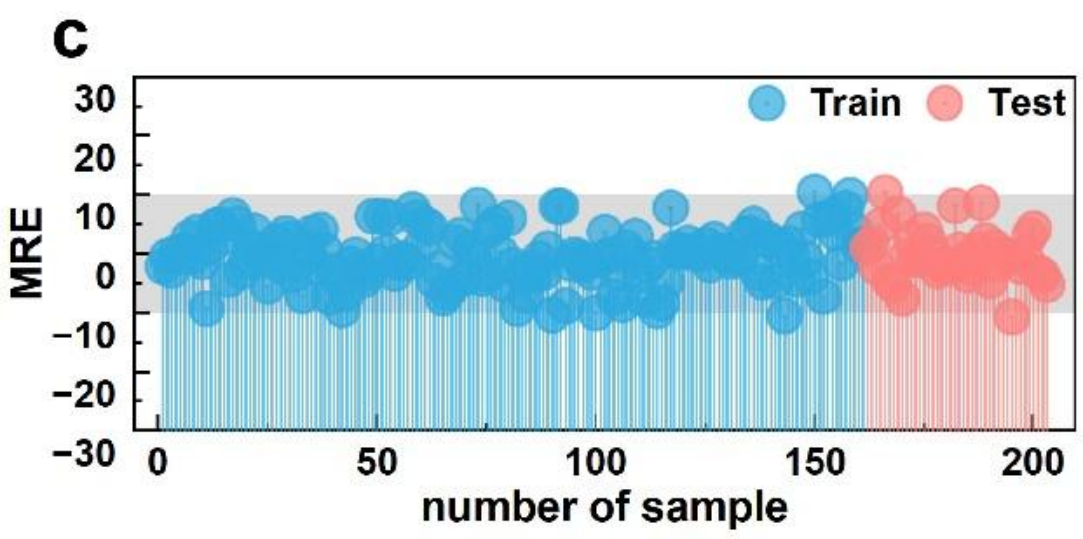

has been changed to

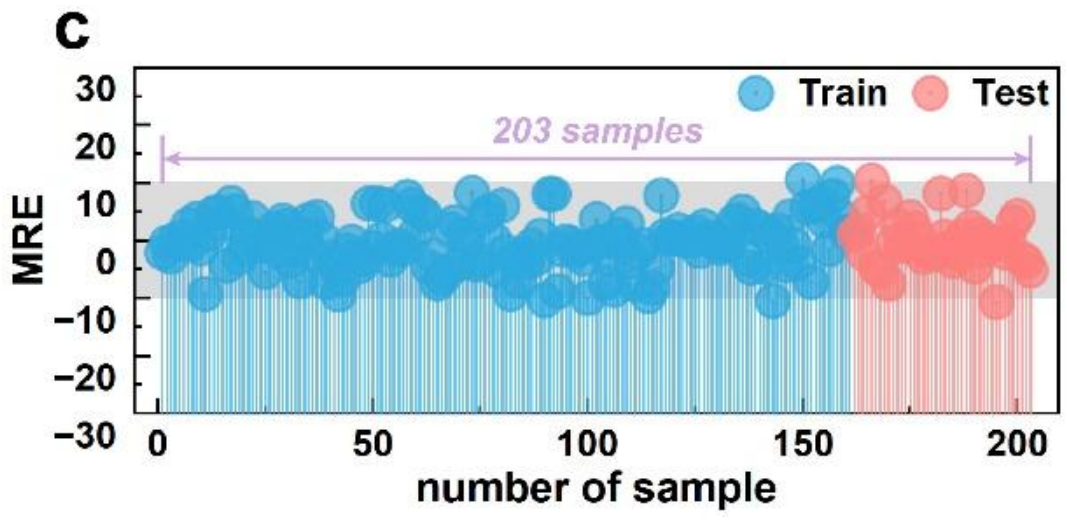


Fig. R29: Model performance comparison. (c) ANN model training results showing predicted versus experimental values for $K_Q$. Training sets in blue and test sets in red. Insets show error distribution histograms (predicted minus experimental values). MRE: Mean Relative Error.

In addition, we provided the complete $K_Q$ dataset containing all 203 samples as shown in Table R12. This table details the composition, experimental measured values, and train-test split assignment for each sample. We uploaded the complete $K_Q$ database (203 samples) and HTC database (34 samples) as Supplementary Data Files containing compositions, experimental values, and train-test split assignments for verification.

Furthermore, we systematically reviewed all other figures throughout the manuscript to ensure consistent sample count reporting: 203 samples (163 training, 40 test) for the $K_Q$ model and 34 samples (27 training, 7 test) for the HTC model. Sample count annotations in all relevant figures have been verified and corrected to ensure complete consistency with the database files.

Table R12. Complete $K_Q$ dataset with 203 samples showing alloy compositions, six selected features (M-F4, am, M-B3, Δχ, Ω, ΔHmix), and experimental fracture toughness values ($K_Q$ in MPa·m$^{1/2}$).

| alloy | M-F4 | am | M-B3 | Δχ | Ω | ΔHmix | $K_Q$ |
|---|---|---|---|---|---|---|---|
| alloy1 | 29.203 | 364.977 | 1.24566 | 12.934 | 0.588794 | -37.632 | 11.5 |
| alloy2 | 28.251 | 356.455 | 1.22878 | 11.88 | 0.685011 | -31.696 | 9.6 |
| alloy3 | 30.608 | 364.128 | 1.32852 | 10.998 | 0.31327 | -30.1056 | 6.8 |
| alloy4 | 27.86 | 326.972 | 1.23195 | 29.763 | 6.308481 | -2.76 | 10.58 |
| alloy5 | 28.098 | 329.1025 | 1.23617 | 29.828 | 3.813367 | -4.8796 | 8.76 |
| alloy6 | 28.574 | 333.3635 | 1.24461 | 29.948 | 2.246795 | -8.9844 | 8.01 |
| alloy7 | 29.05 | 337.6245 | 1.25305 | 30.056 | 1.656499 | -12.91 | 7.72 |
| alloy8 | 27.901 | 352.6852 | 1.21982 | 12.116 | 0.801306 | -28.5072 | 14.5 |
| alloy9 | 27.669 | 351.1236 | 1.21466 | 12.125 | 0.852469 | -28.9968 | 12.4 |
| alloy10 | 27.437 | 349.562 | 1.2095 | 12.13 | 0.882205 | -29.3968 | 10.9 |
| alloy11 | 27.205 | 348.0004 | 1.20434 | 12.129 | 0.900267 | -29.7072 | 9.65 |
| alloy12 | 27.031 | 346.8292 | 1.20047 | 12.126 | 0.908519 | -29.8812 | 8.5 |

| | | | | | | | | |
|---|---|---|---|---|---|---|---|---|
| alloy13 | 28.0065 | 356.0929 | 1.22224 | 12.674 | 0.785282 | -32.6336 | 14.12 |
| alloy14 | 27.5605 | 353.353 | 1.20405 | 18.091 | 0.962408 | -31.7024 | 9.2 |
| alloy15 | 27.2815 | 352.5418 | 1.19808 | 18.075 | 1.021392 | -31.6928 | 10.25 |
| alloy16 | 27.0955 | 352.001 | 1.1941 | 18.064 | 1.04433 | -31.6824 | 8.09 |
| alloy17 | 26.6305 | 350.649 | 1.18415 | 18.036 | 1.07997 | -31.6424 | 6.42 |
| alloy18 | 27.3735 | 353.4456 | 1.20572 | 13.098 | 0.906846 | -31.2824 | 12.8 |
| alloy19 | 27.3975 | 353.1388 | 1.20472 | 15.083 | 0.957512 | -31.324 | 14.9 |
| alloy20 | 27.4335 | 352.6786 | 1.20322 | 17.514 | 1.005694 | -31.3504 | 7.1 |
| alloy21 | 27.4935 | 351.9116 | 1.20072 | 20.645 | 1.062868 | -31.2984 | 5.5 |
| alloy22 | 27.8045 | 354.3936 | 1.21683 | 12.421 | 0.803401 | -30.97 | 10.7 |
| alloy23 | 27.5255 | 353.5824 | 1.21086 | 12.41 | 0.868975 | -30.9688 | 11.9 |
| alloy24 | 27.5005 | 354.12 | 1.20655 | 13.732 | 0.865407 | -31.5764 | 13.8 |
| alloy25 | 27.2215 | 353.3088 | 1.20058 | 13.729 | 0.925889 | -31.6088 | 15.3 |
| alloy26 | 27.8645 | 353.6266 | 1.21433 | 16.939 | 0.906847 | -31.064 | 8.1 |
| alloy27 | 27.5605 | 353.353 | 1.20405 | 18.091 | 0.962408 | -31.7024 | 9.2 |
| alloy28 | 27.5855 | 352.8154 | 1.20836 | 16.917 | 0.971111 | -31.0208 | 6.8 |
| alloy29 | 27.2815 | 352.5418 | 1.19808 | 18.075 | 1.021392 | -31.6928 | 10.3 |
| alloy30 | 27.7875 | 354.3936 | 1.2128 | 12.843 | 0.801001 | -31.0628 | 11.94 |
| alloy31 | 27.7115 | 354.2212 | 1.21023 | 13.174 | 0.843451 | -31.2128 | 11.88 |
| alloy32 | 27.6355 | 354.1528 | 1.20766 | 13.492 | 0.858726 | -31.366 | 12.89 |
| alloy33 | 27.4835 | 354.016 | 1.20252 | 14.091 | 0.879542 | -31.68 | 13.73 |
| alloy34 | 27.1795 | 353.7424 | 1.19224 | 15.161 | 0.899955 | -32.3524 | 15.01 |
| alloy35 | 28.3665 | 358.6841 | 1.23556 | 11.817 | 0.639423 | -31.8312 | 13.7 |
| alloy36 | 28.2985 | 358.2681 | 1.21944 | 13.513 | 0.717902 | -32.188 | 12.5 |
| alloy37 | 28.0765 | 356.7321 | 1.22911 | 11.8 | 0.724095 | -32.3272 | 11.8 |
| alloy38 | 28.0085 | 356.3161 | 1.21299 | 13.525 | 0.795626 | -32.732 | 10.1 |
| alloy39 | 29.008 | 357.136 | 1.25312 | 11.758 | 0.596459 | -30.3616 | 7.8 |
| alloy40 | 28.14225 | 356.0081 | 1.21466 | 14.719 | 0.875001 | -32.4084 | 11.82 |
| alloy41 | 28.16325 | 356.0085 | 1.21655 | 14.674 | 0.843886 | -32.3966 | 10.7 |
| alloy42 | 30.532 | 364.0596 | 1.32595 | 11.435 | 0.350533 | -30.152 | 6 |
| alloy43 | 30.456 | 363.9912 | 1.32338 | 11.85 | 0.377575 | -30.2016 | 6.5 |
| alloy44 | 30.304 | 363.8544 | 1.31824 | 12.622 | 0.420863 | -30.3104 | 10.2 |
| alloy45 | 30 | 363.5808 | 1.30796 | 13.977 | 0.484416 | -30.5664 | 13.3 |
| alloy46 | 28.411 | 357.1542 | 1.23632 | 11.813 | 0.680535 | -31.6032 | 13.49 |
| alloy47 | 28.335 | 357.0858 | 1.23375 | 12.195 | 0.707026 | -31.7336 | 15.4 |

| | | | | | | | |
|---|---|---|---|---|---|---|---|
| alloy48 | 28.259 | 357.0174 | 1.23118 | 12.56 | 0.724479 | -31.8672 | 14.15 |
| alloy49 | 28.107 | 356.8806 | 1.22604 | 13.243 | 0.749711 | -32.144 | 13.47 |
| alloy50 | 27.955 | 356.7438 | 1.2209 | 13.871 | 0.767174 | -32.4336 | 12.99 |
| alloy51 | 27.803 | 356.607 | 1.21576 | 14.452 | 0.779288 | -32.736 | 12.53 |
| alloy52 | 27.651 | 356.4702 | 1.21062 | 14.99 | 0.787261 | -33.0512 | 14.03 |
| alloy53 | 28.86 | 356.2834 | 1.24508 | 12.921 | 0.638351 | -30.4872 | 10.06 |
| alloy54 | 28.884 | 355.9766 | 1.24408 | 14.811 | 0.68178 | -30.6024 | 8.68 |
| alloy55 | 28.908 | 355.6698 | 1.24308 | 16.41 | 0.715508 | -30.6984 | 7.31 |
| alloy56 | 28.225 | 356.6134 | 1.23234 | 11.802 | 0.730837 | -31.6048 | 14.6 |
| alloy57 | 27.946 | 355.8022 | 1.22637 | 11.785 | 0.776615 | -31.6012 | 16.4 |
| alloy58 | 27.481 | 354.4502 | 1.21642 | 11.756 | 0.825934 | -31.5792 | 17 |
| alloy59 | 27.016 | 353.0982 | 1.20647 | 11.724 | 0.855658 | -31.5372 | 18.4 |
| alloy60 | 28.2137 | 357.0356 | 1.23805 | 12.238 | 0.707903 | -31.4752 | 15.1 |
| alloy61 | 27.8191 | 356.7984 | 1.24151 | 13.017 | 0.738747 | -31.3188 | 12.8 |
| alloy62 | 27.4245 | 356.5612 | 1.24497 | 13.714 | 0.760047 | -31.1252 | 11.4 |
| alloy63 | 27.0299 | 356.324 | 1.24843 | 14.342 | 0.775127 | -30.9284 | 4.3 |
| alloy64 | 28.848 | 356.4368 | 1.24558 | 11.824 | 0.605718 | -30.4224 | 6.4 |
| alloy65 | 28.384 | 353.3136 | 1.23526 | 11.795 | 0.72696 | -31.2928 | 13.4 |
| alloy66 | 28.92 | 355.5164 | 1.24258 | 17.126 | 0.730191 | -30.7392 | 6.8 |
| alloy67 | 29.012 | 356.1664 | 1.24708 | 15.57 | 0.664462 | -30.6272 | 7 |
| alloy68 | 28.548 | 353.0432 | 1.23676 | 15.501 | 0.784203 | -31.3952 | 10.9 |
| alloy69 | 28.456 | 352.3932 | 1.23226 | 17.011 | 0.846218 | -31.36 | 8.2 |
| alloy70 | 29.084 | 355.246 | 1.24408 | 19.64 | 0.78909 | -30.8768 | 11.4 |
| alloy71 | 28.62 | 352.1228 | 1.23376 | 19.502 | 0.904513 | -31.3952 | 8.7 |
| alloy72 | 28.07165 | 376.8594 | 1.193429 | 15.048 | 0.792952 | -32.4722 | 13.52 |
| alloy73 | 27.95365 | 376.9306 | 1.196349 | 14.93 | 0.816649 | -32.4881 | 15.39 |
| alloy74 | 27.83565 | 377.0018 | 1.199269 | 14.81 | 0.820175 | -32.5041 | 16.03 |
| alloy75 | 27.71765 | 377.073 | 1.202189 | 14.69 | 0.811827 | -32.52 | 16.12 |
| alloy76 | 27.58785 | 377.1514 | 1.205401 | 14.556 | 0.781504 | -32.5141 | 16.29 |
| alloy77 | 28.688 | 355.7376 | 1.23804 | 11.889 | 0.613005 | -30.4896 | 7.03 |
| alloy78 | 28.384 | 355.464 | 1.22776 | 13.289 | 0.691357 | -30.9248 | 7.21 |
| alloy79 | 28.08 | 355.1904 | 1.21748 | 14.475 | 0.728292 | -31.4112 | 10.49 |
| alloy80 | 27.472 | 354.6432 | 1.19692 | 16.382 | 0.812551 | -30.1824 | 12.47 |
| alloy81 | 28.72 | 357.9872 | 1.24764 | 14.327 | 0.723543 | -30.208 | 8.15 |
| alloy82 | 28.752 | 360.2368 | 1.25724 | 16.233 | 0.715322 | -30.7456 | 8.6 |

| | | | | | | | |
|---|---|---|---|---|---|---|---|
| alloy83 | 28.784 | 362.4864 | 1.26684 | 17.78 | 0.593159 | -32.1024 | 8.24 |
| alloy84 | 29.168 | 357.8352 | 1.26066 | 11.689 | 0.585093 | -30.3072 | 6.19 |
| alloy85 | 28.864 | 357.5616 | 1.25038 | 13.14 | 0.680729 | -30.224 | 10.32 |
| alloy86 | 29.1 | 357.4192 | 1.24454 | 13.421 | 0.678565 | -30.5056 | 6.84 |
| alloy87 | 28.796 | 357.1456 | 1.23426 | 14.614 | 0.757937 | -30.9344 | 10.92 |
| alloy88 | 28.544 | 356.1632 | 1.2353 | 13.241 | 0.699174 | -30.2752 | 10.95 |
| alloy89 | 28.78 | 356.0208 | 1.22946 | 13.517 | 0.694692 | -30.6592 | 7.55 |
| alloy90 | 28.476 | 355.7472 | 1.21918 | 14.685 | 0.785244 | -30.4896 | 11.62 |
| alloy91 | 29.26 | 358.1184 | 1.25208 | 22.479 | 0.667132 | -30.4384 | 7.07 |
| alloy92 | 29.201 | 358.154 | 1.25354 | 13.301 | 0.680095 | -30.4832 | 7.1 |
| alloy93 | 29.142 | 358.1896 | 1.255 | 13.231 | 0.681539 | -30.528 | 8 |
| alloy94 | 29.083 | 358.2252 | 1.25646 | 13.16 | 0.676054 | -30.5728 | 8.17 |
| alloy95 | 29.024 | 358.2608 | 1.25792 | 13.088 | 0.659227 | -30.6176 | 9.01 |
| alloy96 | 29.007 | 358.1568 | 1.25389 | 13.486 | 0.691234 | -30.6704 | 8.71 |
| alloy97 | 28.99 | 358.0528 | 1.24986 | 13.866 | 0.713807 | -30.7264 | 8.92 |
| alloy98 | 28.973 | 357.9488 | 1.24583 | 14.229 | 0.732651 | -30.7856 | 9.73 |
| alloy99 | 28.956 | 357.8448 | 1.2418 | 14.577 | 0.748975 | -30.848 | 9.59 |
| alloy100 | 28.367 | 377.0766 | 1.23136 | 11.884 | 0.633852 | -31.4656 | 6.56 |
| alloy101 | 28.07165 | 355.2826 | 1.193429 | 15.048 | 0.8006 | -32.4722 | 13.75 |
| alloy102 | 28.12765 | 355.4882 | 1.196068 | 15.037 | 0.802309 | -32.4378 | 11.25 |
| alloy103 | 27.98585 | 357.2352 | 1.232375 | 12.702 | 0.784567 | -32.0762 | 8.52 |
| alloy104 | 27.83385 | 357.0984 | 1.227235 | 13.397 | 0.805992 | -32.4222 | 12.64 |
| alloy105 | 27.68185 | 356.9616 | 1.222095 | 14.037 | 0.819755 | -32.781 | 11.27 |
| alloy106 | 27.78855 | 357.1166 | 1.234105 | 13.1 | 0.823242 | -31.2006 | 5.99 |
| alloy107 | 27.63655 | 356.9798 | 1.228965 | 13.789 | 0.84236 | -31.613 | 9.71 |
| alloy108 | 27.48455 | 356.843 | 1.223825 | 14.425 | 0.853665 | -32.0382 | 6.67 |
| alloy109 | 28.09125 | 357.2965 | 1.21143 | 15.667 | 0.809023 | -32.4342 | 14.7 |
| alloy110 | 28.07025 | 357.2961 | 1.20954 | 15.701 | 0.839971 | -32.4476 | 17.1 |
| alloy111 | 28.04925 | 357.2957 | 1.20765 | 15.733 | 0.862595 | -32.461 | 14.53 |
| alloy112 | 28.00725 | 357.2948 | 1.20387 | 15.796 | 0.899344 | -32.4878 | 11.71 |
| alloy113 | 28.42 | 355.0038 | 1.22626 | 16.131 | 0.762123 | -31.124 | 12.26 |
| alloy114 | 28.456 | 354.5436 | 1.22476 | 18.389 | 0.806145 | -31.28 | 12.71 |
| alloy115 | 28.116 | 354.7302 | 1.21598 | 17.226 | 0.795183 | -31.6296 | 14.01 |
| alloy116 | 28.152 | 354.27 | 1.21448 | 19.451 | 0.835967 | -31.8048 | 14.88 |
| alloy117 | 29.088 | 362.76 | 1.27712 | 17.013 | 0.581182 | -31.6416 | 11.38 |

| | | | | | | | |
|---|---|---|---|---|---|---|---|
| alloy118 | 28.856 | 361.1984 | 1.27196 | 17.062 | 0.656951 | -32.3392 | 8.68 |
| alloy119 | 29.02 | 362.344 | 1.261 | 17.96 | 0.66897 | -31.9232 | 11.52 |
| alloy120 | 29.004 | 362.7584 | 1.26956 | 17.111 | 0.682998 | -31.6736 | 10.67 |
| alloy121 | 30.492 | 363.3472 | 1.32594 | 10.978 | 0.376233 | -30.3216 | 8.74 |
| alloy122 | 30.376 | 362.5664 | 1.32336 | 10.956 | 0.418379 | -30.5152 | 8.22 |
| alloy123 | 30.26 | 361.7856 | 1.32078 | 10.933 | 0.452362 | -30.6864 | 6.69 |
| alloy124 | 30.144 | 361.0048 | 1.3182 | 10.909 | 0.480983 | -30.8352 | 6.34 |
| alloy125 | 28.537 | 357.6454 | 1.2358 | 12.652 | 0.723946 | -31.6688 | 10.33 |
| alloy126 | 28.12185 | 355.4882 | 1.195939 | 15.037 | 0.802309 | -32.4378 | 14.02 |
| alloy127 | 26.4851 | 333.7787 | 1.253934 | 2.504 | 1.092275 | -3.60602 | 7.8 |
| alloy128 | 26.5455 | 347.4344 | 1.25204 | 6.524 | 0.67229 | -15.5468 | 10.35 |
| alloy129 | 28.3781 | 363.8392 | 1.284534 | 9.959 | 0.484217 | -28.2173 | 20.2 |
| alloy130 | 27.896 | 347.9148 | 1.2287 | 10.522 | 0.743781 | -23.5488 | 10.81 |
| alloy131 | 28.692 | 353.5742 | 1.25222 | 11.079 | 0.639896 | -26.9984 | 7.85 |
| alloy132 | 27.7725 | 355.7513 | 1.21934 | 12.627 | 0.852294 | -32.46 | 9.9 |
| alloy133 | 27.9585 | 356.2921 | 1.22332 | 12.636 | 0.822429 | -32.48 | 11.1 |
| alloy134 | 27.9465 | 356.9225 | 1.22321 | 12.633 | 0.821399 | -32.2152 | 13.95 |
| alloy135 | 28.1325 | 357.4633 | 1.22719 | 12.641 | 0.790508 | -32.2208 | 14.7 |
| alloy136 | 30.591 | 364.024 | 1.32449 | 11.522 | 0.351556 | -30.1072 | 7.26 |
| alloy137 | 30.557 | 363.816 | 1.31643 | 12.482 | 0.404093 | -30.12 | 7.6 |
| alloy138 | 30.489 | 363.4 | 1.30031 | 14.132 | 0.480353 | -30.184 | 8.5 |
| alloy139 | 28.1585 | 357.3049 | 1.22363 | 12.708 | 0.746077 | -32.332 | 15.12 |
| alloy140 | 31.2506 | 369.8804 | 1.339914 | 11.697 | 0.300126 | -34.0549 | 3.4 |
| alloy141 | 32.75 | 383.3025 | 1.3665 | 12.99 | 0.276555 | -42 | 6.4 |
| alloy142 | 30.4506 | 366.3844 | 1.302214 | 12.116 | 0.470985 | -34.2325 | 11.8 |
| alloy143 | 30.76 | 369.154 | 1.3077 | 12.427 | 0.453901 | -36.08 | 10.3 |
| alloy144 | 27.896 | 347.9148 | 1.2287 | 10.522 | 0.743781 | -23.5488 | 10.5801 |
| alloy145 | 26.933 | 352.4094 | 1.20866 | 10.512 | 0.771102 | -26.7648 | 15.4547 |
| alloy146 | 28.3665 | 358.6841 | 1.23556 | 11.817 | 0.639423 | -31.8312 | 7.51 |
| alloy147 | 31.2506 | 369.8804 | 1.339914 | 11.697 | 0.300126 | -34.0549 | 5.84 |
| alloy148 | 30.8506 | 368.1324 | 1.321064 | 11.912 | 0.409709 | -34.1237 | 6.69 |
| alloy149 | 30.8706 | 369.5384 | 1.327064 | 11.697 | 0.406847 | -34.4917 | 10.4 |
| alloy150 | 30.4706 | 367.7904 | 1.308214 | 11.912 | 0.508749 | -34.6205 | 10.59 |
| alloy151 | 27.207 | 360.0782 | 1.20704 | 11.393 | 0.71364 | -35.1968 | 9.48 |
| alloy152 | 27.167 | 357.2662 | 1.19504 | 11.752 | 0.755601 | -34.2688 | 10.11 |

| | | | | | | | | |
|---|---|---|---|---|---|---|---|---|
| alloy153 | 28.207 | 356.5366 | 1.22382 | 11.948 | 0.643183 | -31.5616 | 13.25 |
| alloy154 | 29.006 | 358.2177 | 1.24603 | 12.143 | 0.584426 | -32.0664 | 6.3 |
| alloy155 | 28.377 | 356.9462 | 1.22826 | 12.71 | 0.729099 | -31.7648 | 12.62 |
| alloy156 | 27.507 | 351.0902 | 1.20891 | 12.676 | 0.831115 | -32.8268 | 8.48 |
| alloy157 | 27.276 | 334.301 | 1.26944 | 4.2 | 0.506632 | -4.3904 | 16.8 |
| alloy158 | 27.633 | 337.4968 | 1.27577 | 5.513 | 0.452324 | -7.5656 | 12.6 |
| alloy159 | 28.377 | 356.9462 | 1.22826 | 12.71 | 0.729099 | -31.7648 | 11.73 |
| alloy160 | 28.573 | 359.5647 | 1.21684 | 14.17 | 0.870489 | -33.4888 | 12.8194 |
| alloy161 | 28.298 | 353.4206 | 1.23643 | 16.305 | 0.879263 | -28.5672 | 8.4065 |
| alloy162 | 28.164 | 351.7464 | 1.21833 | 20.324 | 0.992524 | -28.9072 | 7.8981 |
| alloy163 | 28.03 | 350.0722 | 1.20023 | 23.487 | 1.063826 | -29.1816 | 6.8308 |
| alloy164 | 28.766 | 356.4986 | 1.22625 | 21.13 | 0.925761 | -32.0328 | 7.9803 |
| alloy165 | 28.734 | 355.4484 | 1.23233 | 22.162 | 0.917655 | -31.6872 | 7.4325 |
| alloy166 | 28.42 | 356.0752 | 1.22173 | 17.493 | 0.846414 | -32.0104 | 8.3171 |
| alloy167 | 29.336 | 360.2006 | 1.24025 | 22.882 | 0.875957 | -34.5112 | 7.5121 |
| alloy168 | 29.022 | 360.8274 | 1.22965 | 18.437 | 0.798862 | -35.1 | 8.8503 |
| alloy169 | 28.99 | 359.7772 | 1.23573 | 20.066 | 0.80549 | -34.7088 | 6.7993 |
| alloy170 | 29.056 | 361.0354 | 1.23771 | 17.743 | 0.777449 | -34.8808 | 7.87 |
| alloy171 | 28.546 | 356.5664 | 1.22121 | 18.12 | 0.868774 | -32.0888 | 11.2 |
| alloy172 | 28.58 | 356.7744 | 1.22927 | 17.434 | 0.84396 | -31.9096 | 9.185 |
| alloy173 | 27.863 | 353.3357 | 1.21392 | 12.478 | 0.794219 | -30.4896 | 15.5 |
| alloy174 | 25.2375 | 334.7689 | 1.14236 | 11.447 | 1.347255 | -20.0052 | 13.4 |
| alloy175 | 30.287 | 365.6262 | 1.32184 | 10.99 | 0.366642 | -30.8128 | 16.8 |
| alloy176 | 29.8055 | 367.8735 | 1.31182 | 10.979 | 0.409939 | -31.7656 | 13.2 |
| alloy177 | 29.324 | 370.1208 | 1.3018 | 10.967 | 0.437193 | -32.5888 | 12.1 |
| alloy178 | 30.492 | 363.3472 | 1.32594 | 10.978 | 0.376233 | -30.3216 | 7.9 |
| alloy179 | 30.318 | 362.176 | 1.32207 | 10.945 | 0.436159 | -30.6036 | 10.7 |
| alloy180 | 30.144 | 361.0048 | 1.3182 | 10.909 | 0.480983 | -30.8352 | 10.7 |
| alloy181 | 30.171 | 364.8454 | 1.31926 | 10.97 | 0.426584 | -31.0048 | 11.5 |
| alloy182 | 29.5155 | 365.9215 | 1.30537 | 10.924 | 0.519594 | -32.1136 | 10.8 |
| alloy183 | 28.86 | 366.9976 | 1.29148 | 10.873 | 0.578736 | -32.9344 | 9.4 |
| alloy184 | 29.151 | 357.7312 | 1.25663 | 12.157 | 0.635812 | -30.352 | 7.1 |
| alloy185 | 29.117 | 357.5232 | 1.24857 | 13.02 | 0.678521 | -30.4512 | 8.9 |
| alloy186 | 29.083 | 357.3152 | 1.24051 | 13.804 | 0.710443 | -30.5632 | 11.1 |
| alloy187 | 28.991 | 357.032 | 1.24909 | 12.219 | 0.628883 | -30.4112 | 6.3 |

| | | | | | | | |
|---|---|---|---|---|---|---|---|
| alloy188 | 28.957 | 356.824 | 1.24103 | 13.073 | 0.671017 | -30.52 | 7.5 |
| alloy189 | 28.923 | 356.616 | 1.23297 | 13.849 | 0.702431 | -30.6416 | 7.9 |
| alloy190 | 28.831 | 356.3328 | 1.24155 | 12.28 | 0.637506 | -30.4768 | 10.1 |
| alloy191 | 28.797 | 356.1248 | 1.23349 | 13.125 | 0.67854 | -30.5952 | 8.1 |
| alloy192 | 28.763 | 355.9168 | 1.22543 | 13.892 | 0.708922 | -30.7264 | 6.5 |
| alloy193 | 30.532 | 364.0596 | 1.32595 | 11.435 | 0.350533 | -30.152 | 6.01 |
| alloy194 | 30.456 | 363.9912 | 1.32338 | 11.85 | 0.377575 | -30.2016 | 6.45 |
| alloy195 | 30.304 | 363.8544 | 1.31824 | 12.622 | 0.420863 | -30.3104 | 10.16 |
| alloy196 | 28.39525 | 357.5701 | 1.22171 | 14.649 | 0.78081 | -31.7694 | 13.31 |
| alloy197 | 28.37425 | 357.5697 | 1.21982 | 14.692 | 0.810742 | -31.8748 | 15.75 |
| alloy198 | 28.35325 | 357.5693 | 1.21793 | 14.734 | 0.834407 | -31.8866 | 11.45 |
| alloy199 | 28.33225 | 357.5688 | 1.21604 | 14.775 | 0.854808 | -31.8984 | 10.35 |
| alloy200 | 28.31125 | 357.5685 | 1.21415 | 14.816 | 0.873053 | -31.9102 | 10.69 |
| alloy201 | 28.14225 | 356.0081 | 1.21466 | 14.719 | 0.875001 | -32.4084 | 10.75 |
| alloy202 | 29.18 | 351.345 | 1.3032 | 9 | 0.354441 | -20.16 | 9.5 |
| alloy203 | 30.152 | 363.7176 | 1.3131 | 13.328 | 0.474121 | -30.432 | 11.91 |

Action taken: We corrected Fig. 3 with accurate sample counts. We uploaded complete $K_Q$ database (203 samples) and HTC database (34 samples) as Supplementary Data Files.

2. The response repeatedly emphasizes the importance of combining feature selection, SHAP analysis, and NSGA-II. What would be the impact if any one of these components were omitted? Further analysis and discussion are recommended.

**Response:**

We carried out systematic ablation studies quantifying the impact of omitting feature selection, SHAP analysis, or NSGA-II optimization.

For feature selection, we compared models with complete PCC-MIC-RFE screening versus models without this screening using identical neural network architectures. For SHAP analysis, we performed optimization with and without SHAP-derived element selection and design constraints. For NSGA-II, we compared multi-objective genetic algorithm optimization against random search baseline.

**(I) Impact of Omitting Feature Selection**

Omitting feature selection degrades model performance. For the $K_Q$ model, Fig. R30a shows test $R^2$ drops from 0.96 (with feature selection) to 0.91 (without feature

selection), while Fig. R30e shows test RMSE increases from 1.47 to 1.50 MPa·m$^{1/2}$. Fig. R30b reveals the generalization gap widens with $R^2$ gap increasing from 0.04 to 0.06, and Fig. R30f shows RMSE gap increasing from 0.05 to 0.12. Fig. R30j demonstrates MAE gap increases from 0.03 to 0.15. Fig. R30m and Fig. R30q show the training process becomes significantly more unstable without feature selection. Fig. R30n illustrates the stark difference in dimensionality with 103 features retained without complete screening versus only 6 features after PCC-MIC-RFE, while Fig. R30r shows model complexity explodes from 241 parameters to 24,129 parameters without feature selection. For the HTC model, Fig. R30c shows test $R^2$ remains similar (0.98 with feature selection versus 0.99 without), but Fig. R30d reveals $R^2$ gap increases from 0.0018 to 0.0084. More critically, Fig. R30h shows RMSE gap improves substantially with feature selection (0.30 versus 0.31 without), and Fig. R30l shows MAE gap decreases dramatically from 0.35 to 0.08 with feature selection. Fig. R30p shows feature count reduction from 96 to 6, while Fig. R30t reveals model parameters decrease from 23,233 to 863.

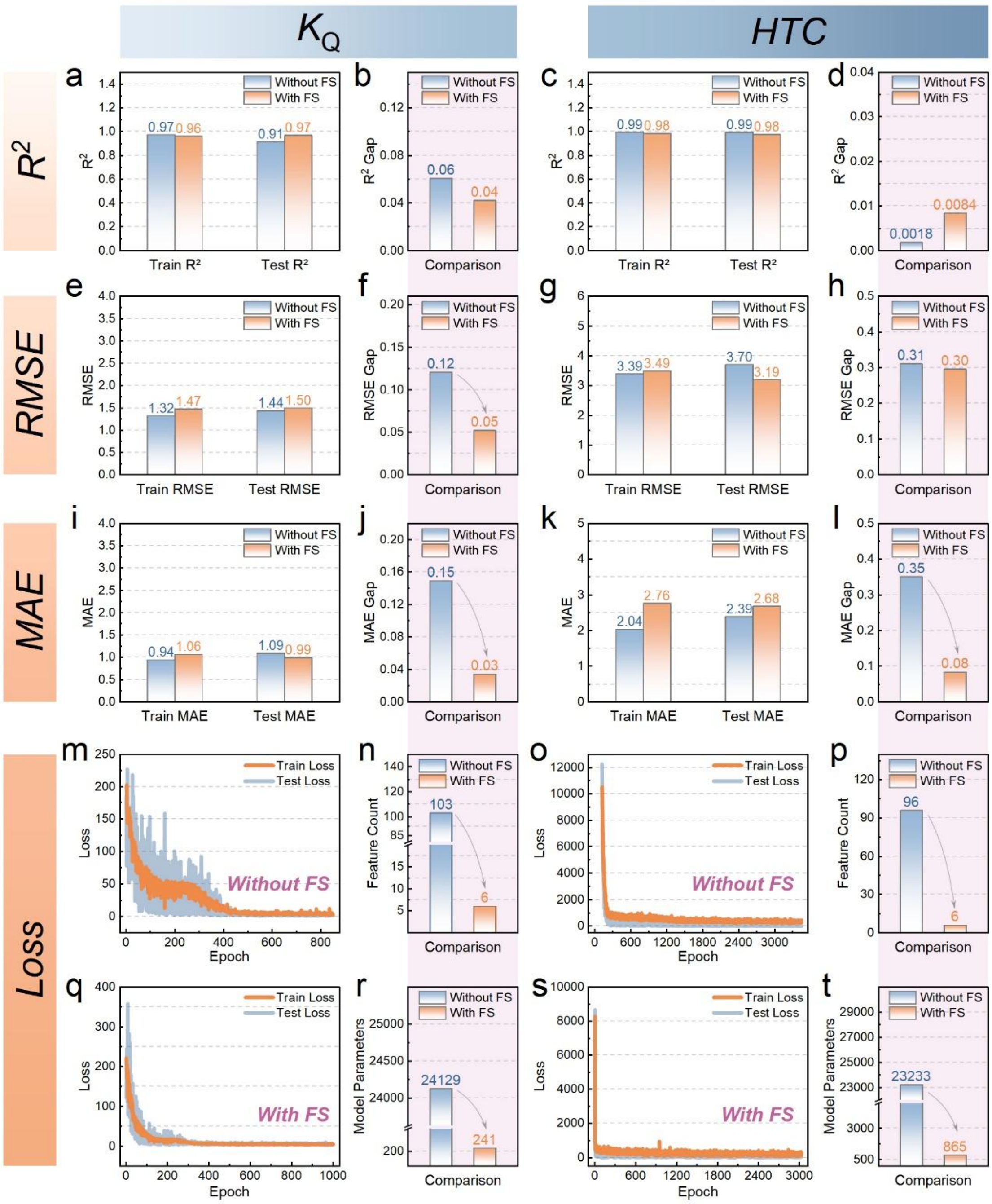


Fig. R30. Impact of feature selection on model performance. Performance comparison for $K_Q$ (a-b, e-f, i-j, m-n, q-r) and HTC (c-d, g-h, k-l, o-p, s-t) models with and without feature selection: (a,c) train/test $R^2$; (b,d) $R^2$ gap; (e,g) train/test RMSE; (f,h) RMSE gap; (i,k) train/test MAE; (j,l) MAE gap; (m,o,q,s) loss curves; (n,p) feature count; (r,t) model parameters.

## (II) Impact of Omitting SHAP Analysis

Omitting SHAP analysis removes critical guidance on feature importance/influence on the property. Model prediction accuracy remains identical because SHAP is a post-hoc interpretation tool not affecting training. However, optimization loses two essential capabilities. Without SHAP-based element selection,

optimization explores the full 15-element space (Nb, Si, Ti, Al, Cr, Hf, Zr, V, Mo, W, Ta, Re, Fe, Ge, Sn) rather than focusing on key elements influencing property. SHAP analysis ranked elements by weighted contributions using Score = $\Sigma(\alpha_i \times \lambda_i)$, where influence coefficients α derived from SHAP value-feature linear regression and weight coefficients λ reflect relative feature importance. This ranking identified Cr (12.08), Hf (13.16), Zr (13.39), and V (13.83) as key additions, with lower scores indicating stronger beneficial effects on both $K_Q$ and HTC. Without this screening, subsequent optimization includes potentially detrimental elements, reducing efficiency. Without SHAP-derived descriptor constraints, optimization operates in unconstrained 6-dimensional space without understanding favorable microstructural ranges. SHAP revealed that the melting enthalpy descriptor M-F4 contributes positively below 28.5 and atomic radius mismatch am above 350 pm, translating to constraints preventing excessive silicide fractions. Optimization without these mechanistic constraints may yield compositions with reasonable predicted properties but unfavorable microstructures such as continuous brittle silicide networks.

**(III) Impact of Replacing NSGA-II with Random Search**

Replacing NSGA-II with random search produces substantially inferior optimization performance. Fig. R31a shows the comparison for 8-element alloys where random search generates widely scattered points across the entire $K_Q$-HTC space without structure, while NSGA-II concentrates evaluations along a clear Pareto front. Similar patterns appear in Fig. R31b for 7-element alloys, Fig. R31e for 6-element alloys, and Fig. R31f for 5-element alloys, consistently demonstrating NSGA-II's ability to identify the optimal trade-off frontier between the two objectives. For 8-element alloys, Fig. R31c shows three key results: in $K_Q$ convergence, NSGA-II reaches 20.5 MPa·m$^{1/2}$ after 100,000 evaluations while random search plateaus at 19.0 MPa·m$^{1/2}$; in HTC convergence, both methods achieve similar values around 520 MPa; for computational cost, NSGA-II requires 129.23 seconds versus 4.81 seconds for random search. Although NSGA-II demands substantially longer computation time, this cost remains acceptable given the superior solution quality and complete Pareto front identification. Fig. R31d for 7-element alloys, Fig. R31g for 6-element alloys, and Fig. R31h for 5-element alloys show consistent patterns with computation times of 130.82s, 126.95s, and 127.67s for NSGA-II versus 4.82s, 4.23s, and 3.58s for random search. The key advantage lies not merely in finding better solutions but in generating complete Pareto fronts with well-distributed compositions allowing selection based on different $K_Q$-HTC trade-off preferences. Random search cannot identify this frontier

structure and produces randomly scattered solutions potentially missing optimal trade-off regions.

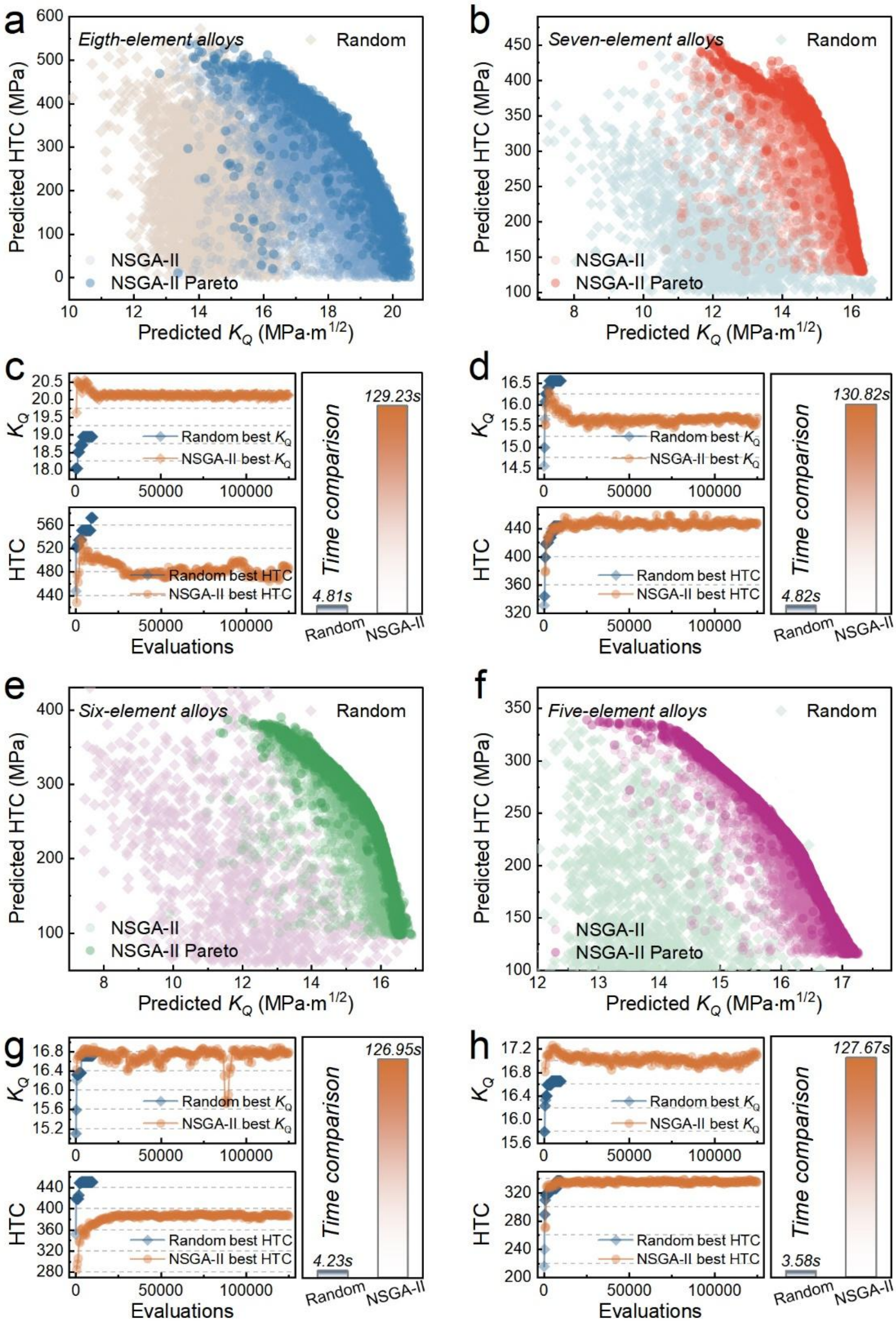


Fig. R31. NSGA-II versus random search for multi-objective optimization. (a, b, e, f) Predicted $K_Q$ vs HTC for 8-, 7-, 6-, and 5-element alloys showing random search, NSGA-II evaluations, and Pareto fronts. (c, d, g, h) Convergence of best $K_Q$ and HTC, and computational time for each system.

The three components each address distinct challenges in the workflow. PCC-MIC-RFE reduces dimensionality from 103 (for $K_Q$) and 97 (for HTC) to 6 features,

enabling efficient training and reliable predictions. SHAP screens beneficial elements from 15 candidates and provides mechanistic constraints guiding optimization toward favorable microstructures. NSGA-II efficiently explores composition space while identifying complete Pareto fronts for multi-objective optimization. Ablation studies demonstrate that each component plays an essential role, with omission of any single component degrading overall system performance.

Action taken: Add Figs. S6-S7 and Note S3 in Supporting Information. The revisions have been added to the "Feature screening and model construction" subsection in the **Results and discussion** section.

3. The manuscript computes PCC and MIC between each feature and performance for preliminary feature screening and uses relatively low thresholds (PCC > 0.2, MIC > 0.2 in the first model; PCC > 0.09, MIC > 0.08 in the second model). What is the rationale for choosing these thresholds? Why is there such a large discrepancy between the two models?

**Response:**

**(I) What is the rationale for choosing these thresholds?**

We performed systematic grid search to determine optimal PCC and MIC thresholds and explain the large threshold difference between the two models. For the $K_Q$ model with 203 samples, we tested 361 threshold combinations with PCC ranging from 0.05 to 0.95 and MIC ranging from 0.05 to 0.95 at step size 0.05. For the HTC model with 34 samples, we tested 380 combinations with PCC ranging from 0.03 to 0.40 and MIC ranging from 0.02 to 0.40 at step size 0.02. Each combination underwent complete modeling including filtering, RFE selection, and neural network training with identical architecture to ensure fair comparison.

Grid search revealed markedly different optimal threshold landscapes for the two models. For the $K_Q$ model, Fig. R32a, c, and e show that stable performance occurs in the region with PCC ranging from 0.05 to 0.25 and MIC ranging from 0.15 to 0.25, where train $R^2$ remains around 0.96-0.97 and test $R^2$ varies between 0.87 and 0.97. Beyond PCC=0.65 or MIC=0.25, white regions with "--" indicate insufficient features after filtering. For the HTC model, Fig. R32b, d, and f show performance concentrated in a much narrower low-threshold region with PCC ranging from 0.03 to 0.09 and MIC ranging from 0.04 to 0.1, where train $R^2$ reaches 0.96-0.98 and test $R^2$ ranges from 0.97 to 0.99. Beyond PCC=0.15 or MIC=0.12, extensive white regions indicate insufficient

features for valid model training. The viable performance region is substantially narrower for HTC than $K_Q$, demonstrating that permissive thresholds are essential for small sample sizes.

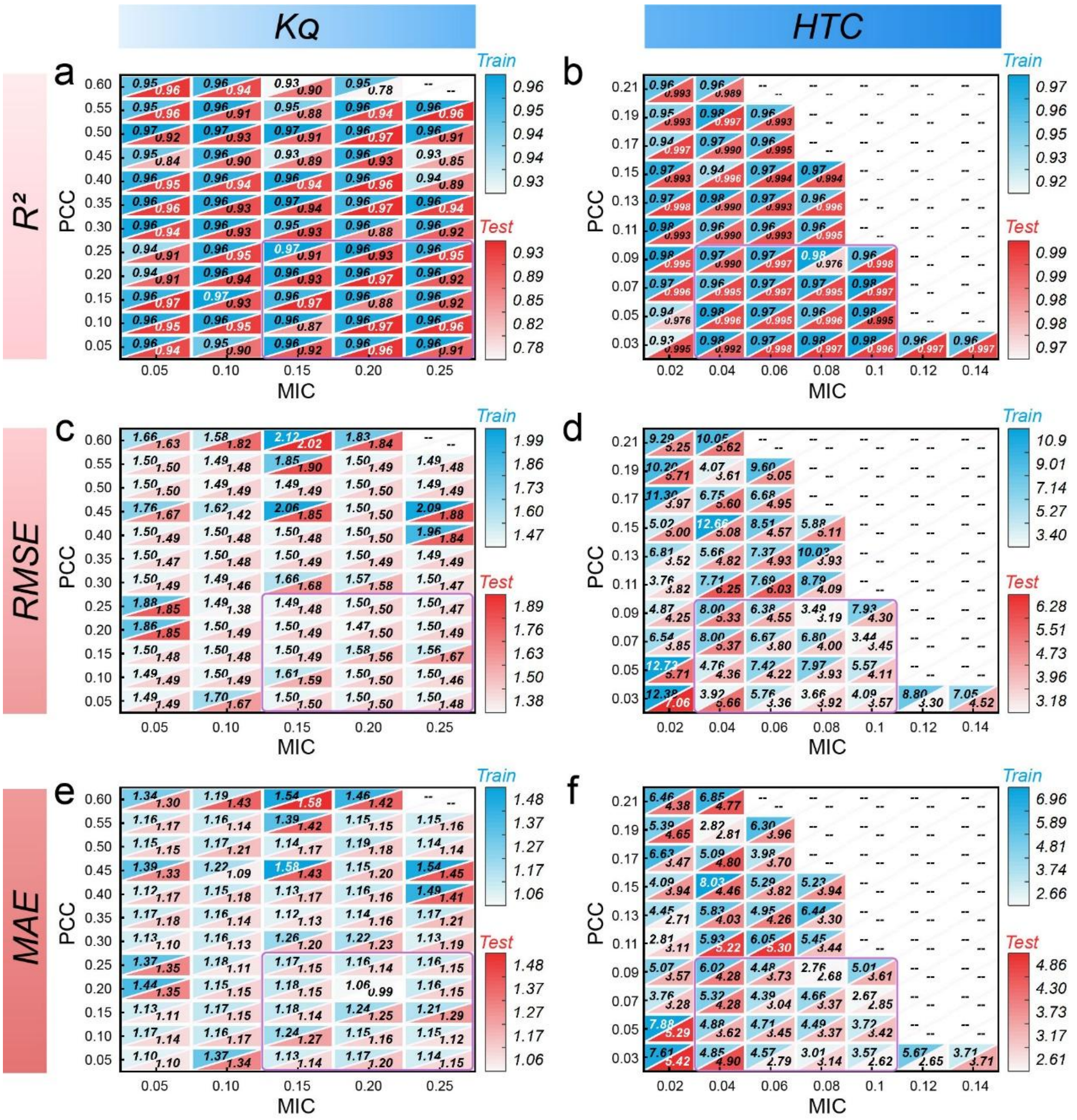


Fig. R32. Grid search results across threshold space. (a, c, e) $K_Q$ model. (b, d, f) HTC model. (a, b) Test $R^2$. (c, d) Test RMSE. (e, f) Test MAE. White regions indicate threshold combinations where insufficient features remained after filtering. Combinations beyond the displayed ranges yielded no valid models.

We quantitatively compared the top 5 threshold combinations for each model across multiple evaluation dimensions including $R^2$, RMSE, MAE, train-test differences, and generalization ratios. For the $K_Q$ model, Fig. R33a c, e, g, i, k show that the top-ranked combinations maintain train $R^2$ consistently at 0.96, with test $R^2$ ranging from 0.87 to 0.94, minimal $|\Delta R^2|$ values between 0.001 and 0.022, and generalization ratios (test/train for RMSE and MAE) ranging from 0.923 to 1.012. For

the HTC model, Fig. R33b d, f, h, j, l show train $R^2$ at 0.98-0.99 and test $R^2$ at 0.98-0.99, $|\Delta R^2|$ values between 0.008 and 0.021, and generalization ratios ranging from 0.874 to 1.017. These top-ranked combinations exhibit nearly identical comprehensive performance across all metrics, indicating that multiple threshold selections within the stable regions yield statistically equivalent results. Our selected thresholds (PCC=0.20, MIC=0.20 for $K_Q$; PCC=0.09, MIC=0.08 for HTC) fall within these validated high-performance regions.

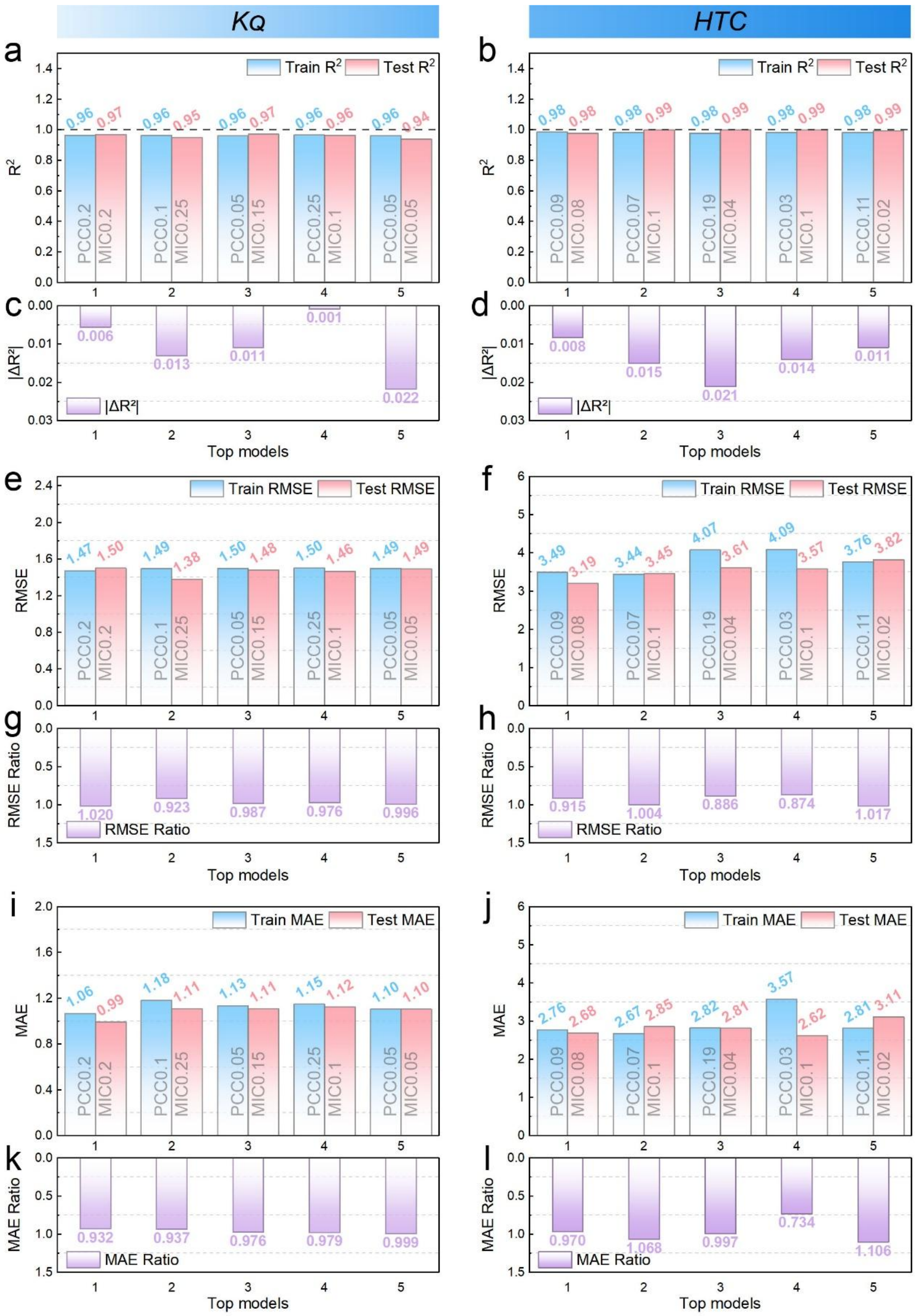


Fig. R33. Comprehensive performance comparison of top 5 threshold combinations. (a, c, e, g, i, k) $K_Q$ model results. (b, d, f, h, j, l) HTC model results. (a, b) Train and test $R^2$ values. (c, d) Train and test RMSE values. (e, f) Train and test MAE values. (g, h) Absolute difference between train and test $R^2$. (i, j) Ratio of test RMSE to train RMSE. (k, l) Ratio of test MAE to train MAE.

**(II) Why is there such a large discrepancy between the two models?**

The large threshold difference between models directly results from drastically different sample sizes and statistical constraints. With 203 samples, the standard error of Pearson correlation follows $SE(r)=\frac{1}{\sqrt{n-3}}$, giving SE=0.071 for the $K_Q$ model. The threshold PCC=0.20 represents 2.8 standard errors, providing reasonable confidence for preliminary screening. With only 34 samples, the HTC model faces SE=0.180, making even moderate correlations highly uncertain. The threshold PCC=0.09 represents 0.5 standard errors, which is fine to preserve potentially informative features for subsequent RFE evaluation rather than prematurely discarding them based on unreliable correlation estimates. Statistical power analysis supports this interpretation. The power to detect correlation coefficient r at significance level α with sample size n follows:

$$\text{Power}=\Phi(z_r-z_{1-\alpha/2}) \tag{5}$$

where $z_r=\frac{1}{2}\ln\left(\frac{1+r}{1-r}\right)\sqrt{n-3}$ is the Fisher Z-transformation and Φ is the cumulative standard normal distribution function.

For the $K_Q$ model, detecting r=0.20 with n=203 at α=0.05 yields power=0.74, which is acceptable for preliminary screening. For the HTC model, detecting r=0.09 with n=34 gives power=0.11, which is very low but unavoidable given severe sample limitation. This limited power necessitates permissive initial filtering to retain more candidate features for subsequent RFE evaluation. Through systematic grid search evaluating 361 and 380 threshold combinations for $K_Q$ and HTC models respectively, we identified stable performance regions where our selected thresholds (PCC=0.20, MIC=0.20 for $K_Q$; PCC=0.09, MIC=0.08 for HTC) fall within the optimal stable regions, validating the rationale for threshold selection. The large threshold difference between the two models exists because the sample size disparity (n=203 versus n=34) creates different statistical constraints. The $K_Q$ model with larger sample size can use higher thresholds (PCC=0.20) while maintaining acceptable statistical power (0.74), whereas the HTC model with limited samples must use lower thresholds (PCC=0.09) to avoid prematurely discarding potentially informative features due to insufficient statistical power (0.11).

Action taken: Add Figs. S8-9 and Note S2 in Supporting Information. The revisions have been added to the "Feature screening and model construction" subsection in the **Results and discussion** section.

Thank you and the reviewers again. We hope that our responses could meet the requirements of the reviewers and you. If there are any questions about our revised manuscript, please don’t hesitate to contact us.

Ruirun Chen

Ph.D, Professor

Director, Department of Interdisciplinary Science

Associate director, National Key Laboratory for Precision Hot Processing of Metals

Harbin Institute of Technology, Harbin, 150001, China

Email: ruirunchen@hit.edu.cn

# Response to reviewers' comments

We thank the reviewers for the feedback and suggestions that have helped to improve the manuscript. We have made changes to address the issues raised. In the following, we provide a point-by-point response to the comments in blue. Action taken is described in red. The revisions in the manuscript are highlighted in red font.

**Reviewer #2 (Remarks to the Author):**

The revised manuscript has substantially improved in quality. However, several minor issues remain that could be further refined. The following specific suggestions are offered:

Response:

We thank the reviewer for the positive assessment and for the constructive suggestions. We have addressed all four comments through additional analyses, new figures, and manuscript revisions as detailed below.

Comment 1: While the manuscript systematically demonstrates the use of feature selection, SHAP analysis, and NSGA-II, it lacks a scientific rationale for the selection of these methods. It is recommended to elaborate on the methodological justification, including their suitability for small datasets and related considerations.

**Response:**

We thank the reviewer for this suggestion. We have added explicit methodological justification for each of the three methods in the relevant sections of the manuscript.

For the three-step feature screening strategy, the design rationale is as follows. The sample-to-feature ratio in this study is low (203 samples for 103 $K_Q$ features; 34 samples for 97 HTC features), which creates a high risk of multicollinearity and overfitting when standard feature selection methods are applied directly. PCC-based redundancy removal first eliminates highly correlated features ($|r| > 0.95$) that arise from periodic trends amongst element-derived descriptors, reducing dimensionality while preserving independent information. Joint PCC-MIC screening then retains features with either linear or nonlinear associations with the target property, preventing premature exclusion of physically meaningful descriptors that may not exhibit strong linear correlation. RFE with ElasticNet (L1+L2 regularization) is well-suited for small datasets because it performs simultaneous feature selection and regularization, reducing

the risk of overfitting without requiring a large sample size. This sequential strategy ensures that the final six features represent statistically stable and physically interpretable descriptors rather than dataset-specific artifacts.

For SHAP, the method was selected over alternatives such as LIME and integrated gradients because it satisfies four axiomatic properties: local accuracy, consistency, symmetry, and the dummy property. These properties guarantee that feature attributions are theoretically unbiased and internally consistent, which is especially important for small datasets where alternative methods may produce unstable attributions due to limited sample diversity.

For NSGA-II, the method was selected because it generates a complete Pareto front in a single optimization run, covering the full trade-off spectrum between $K_Q$ and HTC without requiring separate optimizations at each weight setting. Its computational cost scales well with the number of objectives, and it has been widely validated for continuous alloy composition optimization. The 1:1 weighting between $K_Q$ and HTC reflects the practical requirement that both processability ($K_Q > 18$ MPa·m$^{1/2}$) and high-temperature service performance must be met simultaneously for the alloy to be viable; prioritizing one property at the expense of the other would defeat the design objective.

Action taken: The revisions have been added to the Materials and Methods section.

Comment 2: The manuscript states that the designed alloys outperform all reported cast Nb–Si alloys, yet the main text does not provide an intuitive comparative performance plot. Consider moving the comparative figure from the Supplementary Information to the Results section and presenting fracture toughness and high-temperature strength in a dual-axis plot.

**Response:**

We agree that a comparative figure in the main text would strengthen the presentation. The $K_Q$ and HTC comparison plots have been added to the main text as Fig. 6n, o, placed within the "Multi-objective optimization and experimental validation" section.

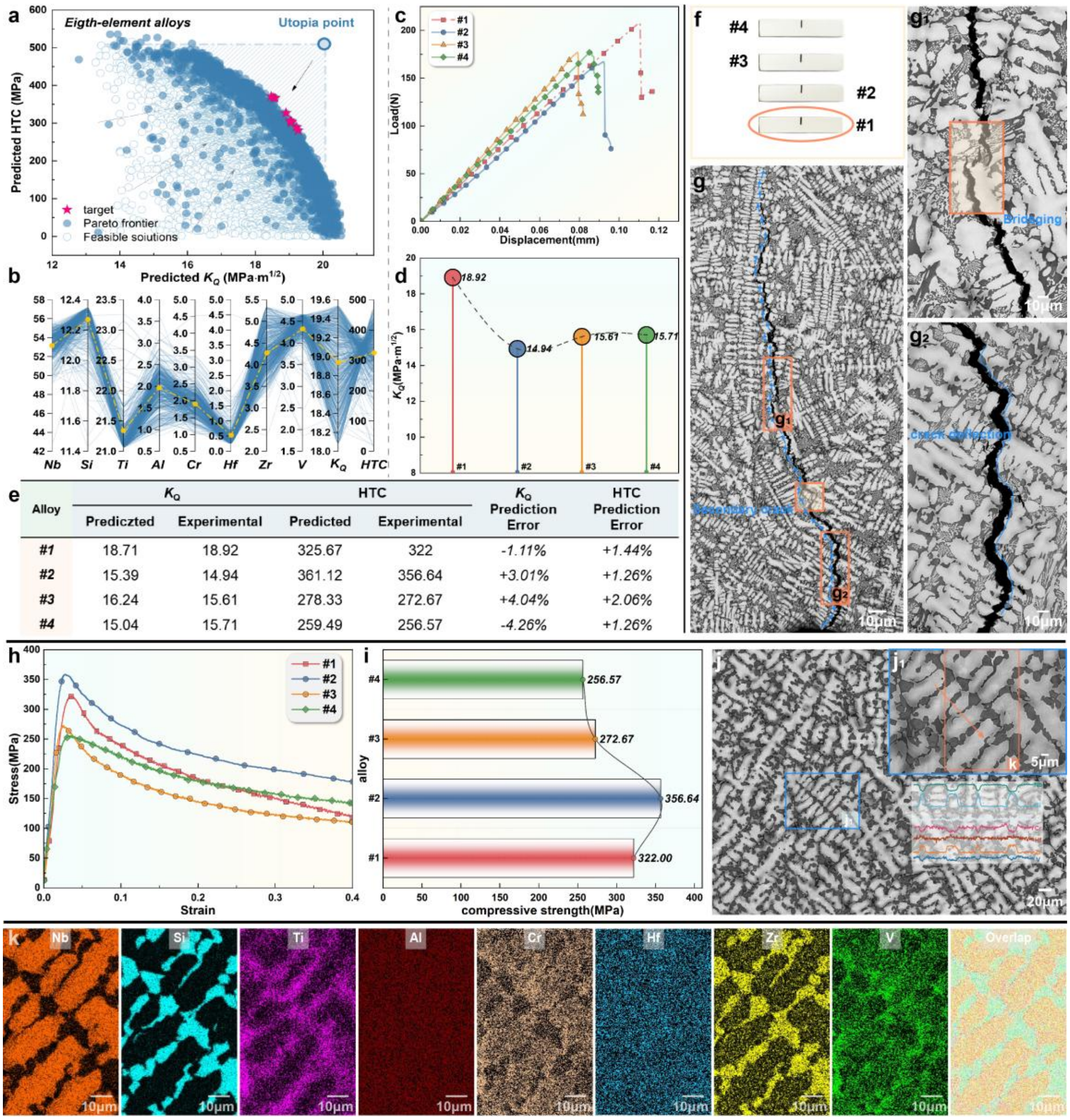


e

| Alloy | $K_Q$ Predicted | $K_Q$ Experimental | HTC Predicted | HTC Experimental | $K_Q$ Prediction Error | HTC Prediction Error |
|---|---|---|---|---|---|---|
| #1 | 18.71 | 18.92 | 325.67 | 322 | -1.11% | +1.44% |
| #2 | 15.39 | 14.94 | 361.12 | 356.64 | +3.01% | +1.26% |
| #3 | 16.24 | 15.61 | 278.33 | 272.67 | +4.04% | +2.06% |
| #4 | 15.04 | 15.71 | 259.49 | 256.57 | -4.26% | +1.26% |

has been changed to

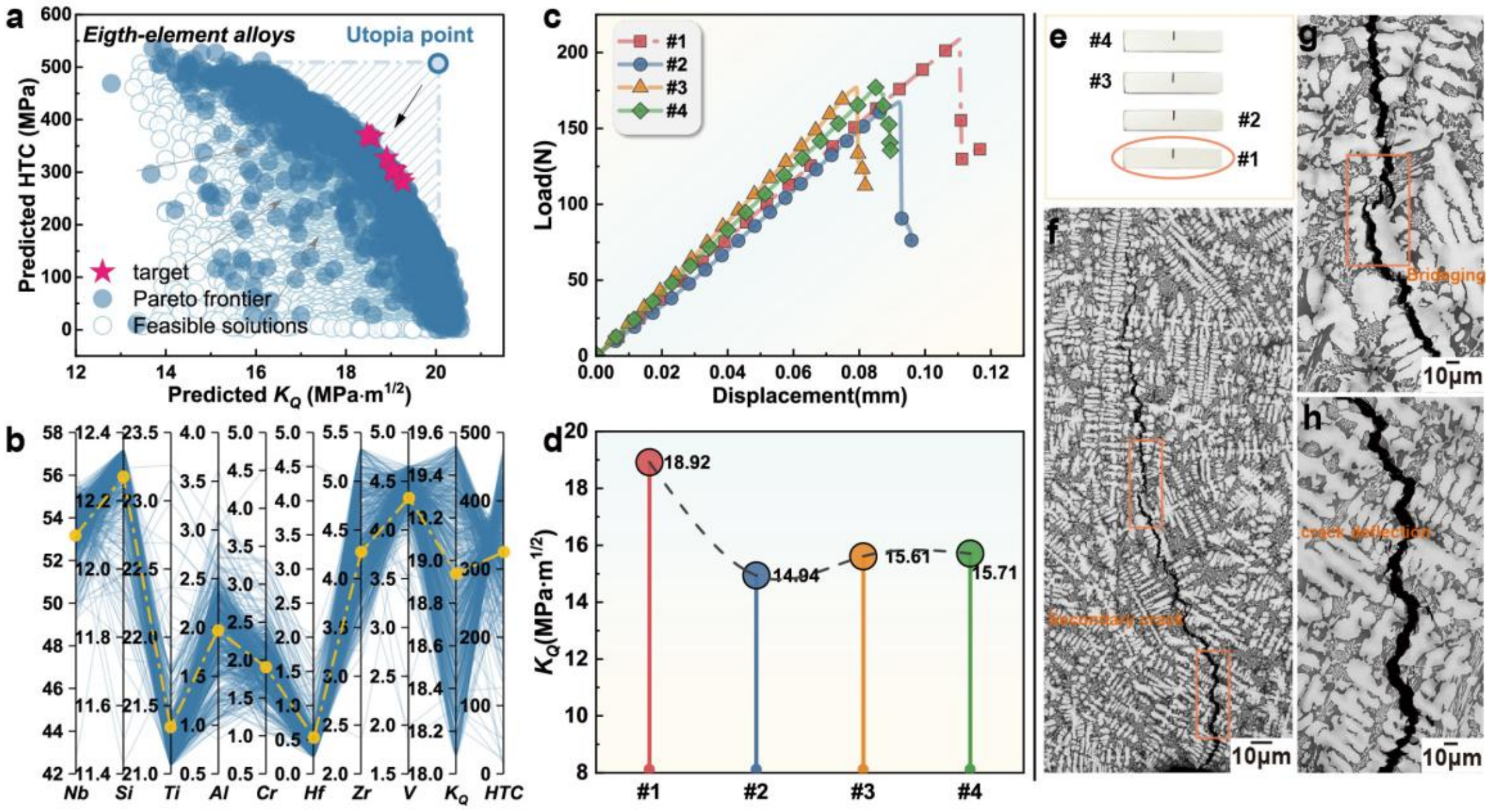

Fig. R1 Multi-objective optimization and experimental validation. a, Pareto frontier for the 8-element system; gray circles: feasible solutions (n = 10,000); pink circles: Pareto-optimal; pink stars: selected compositions; open blue circle: utopia point. b, Parallel coordinate plot of Pareto-optimal alloys; yellow lines: validated compositions #1. c, Load-displacement curves from three-point bending tests (ASTM E399, 25°C). d, Measured $K_Q$ for alloys #1-#4; error bars: SD of n = 3 independent specimens per alloy. e, Macrographs of fractured specimens for alloys #1–#4. f, SEM image (BSE, 20 kV) of the crack propagation path in alloy #1; orange boxes indicate the regions enlarged in (g) and (h), respectively; a secondary crack is visible in the lower region. g, Enlarged view of the upper region marked in (f), showing crack bridging. h, Enlarged view of the lower region marked in (f), showing crack deflection. no technical replicates were used. BSE, backscattered electron.

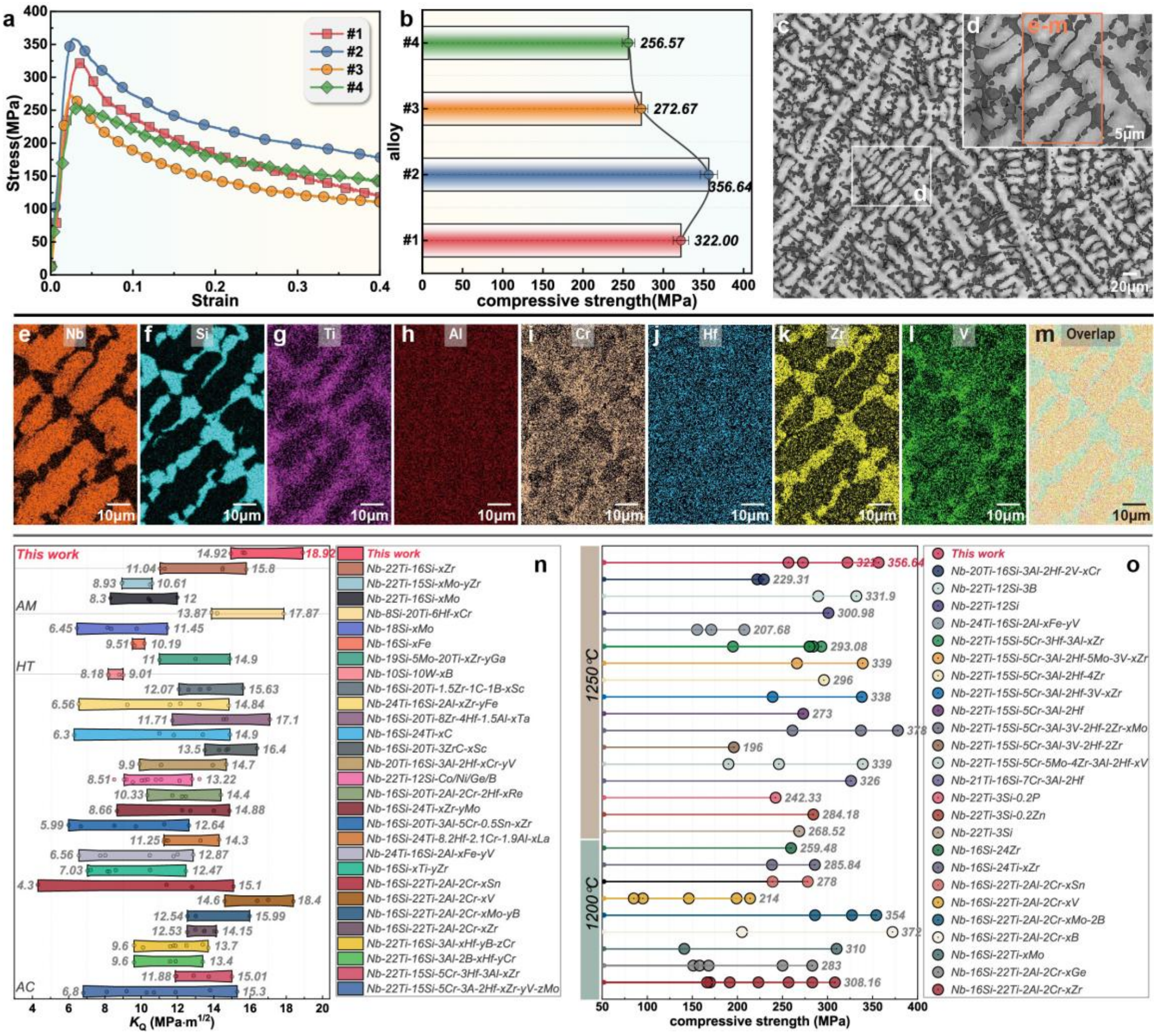


Fig. R2 High-temperature compression testing and microstructural characterization. a, Compressive stress-strain curves at 1250°C for alloys #1–#4 (Gleeble 1500D, strain rate 0.005 s$^{-1}$, vacuum). b, Compressive strength at 1250°C for alloys #1–#4; error bars: SD of n = 3 independent specimens per alloy. c, SEM image (BSE, 15 kV) of alloy #1 after compression showing the two-phase microstructure. d, Enlarged view of the region marked in (c) with EDS line scan. e-m, EDS elemental maps of alloy #1 after compression: (e) Nb, (f) Si, (g) Ti, (h) Al, (i) Cr, (j) Hf, (k) Zr, (l) V, (m) overlap. n, l, Literature comparison of (n) $K_Q$ and (o) high-temperature compressive strength between this work and reported as-cast Nb-Si alloys; each symbol: one independently reported alloy. AM: arc-melted; HT: heat-treated; AC: as-cast.

Fig. R2n, o compare $K_Q$ and HTC of our alloys against all reported as-cast Nb-Si

alloys in the literature. Sample #1 achieves the highest as-cast $K_Q$ (18.92 MPa·m$^{1/2}$) of all reported cast Nb-Si alloys while maintaining 322 MPa compressive strength at 1250°C, confirming that ML-guided multi-objective optimization resolves the $K_Q$–HTC trade-off that has constrained Nb-Si alloy development.

We also constructed a dual-axis plot combining $K_Q$ and HTC on a single figure (Fig. R3). A search of the literature found only 5 alloy systems in which both properties were reported for the same alloy. Our alloys remain in the top-right region of this plot, but the small number of data points does not represent the full range of published performance for either property. Most prior studies measured only one of the two properties, so the available overlap is inherently limited. Presenting $K_Q$ and HTC in separate panels (Fig. R2n, o) allows each comparison to draw on the complete published dataset ($K_Q$: 29 alloys; HTC: 25 alloys), giving a more accurate view of relative performance. The scarcity of studies reporting both properties also reflects that simultaneous optimization of $K_Q$ and HTC has rarely been attempted, which further supports the motivation of this work.

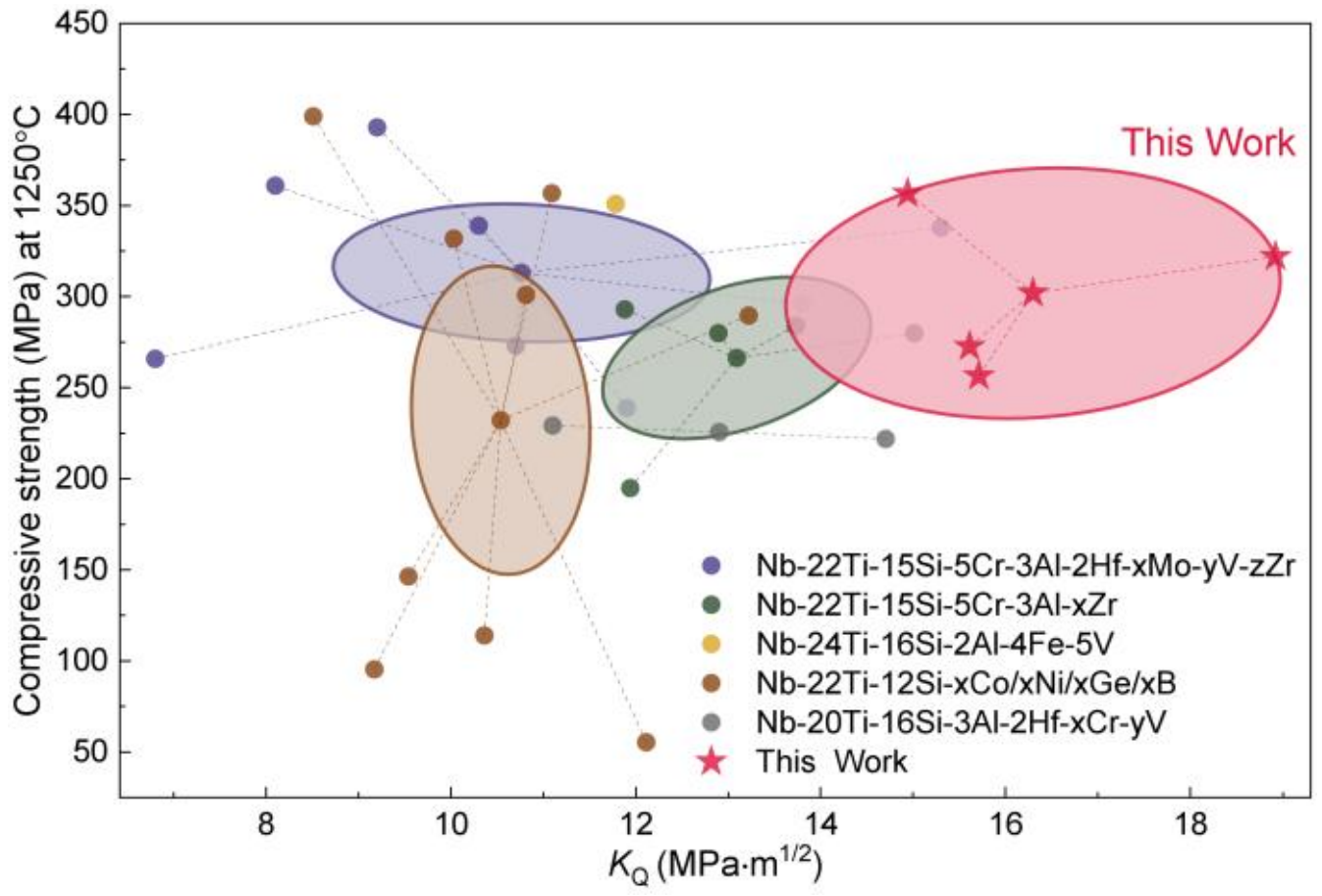


Fig. R3. Dual-axis plot of $K_Q$ and compressive strength at 1250°C. Pink stars: this work; circles: literature data (5 alloys reporting both properties).

Comment 3: The HTC model is trained on only 34 samples, and its representativeness warrants further discussion. The manuscript does not examine whether the compositional distribution of these samples adequately covers the design space, nor does it assess potential clustering biases among samples. Insufficient representativeness may lead to an overestimation of the model's generalization capability.

**Response:**

We have added a dedicated discussion of HTC dataset representativeness to the "Feature screening and model construction" section.

The 34 HTC samples cover Si content from 12 to 18 at.%, Ti content from 0 to 35 at.%, and alloy complexity from 5 to 7 elements, spanning the dominant compositional design space for cast Nb-Si alloys defined by the full 203-sample dataset. PCA of the 11-dimensional compositional space shows that the training and test subsets occupy overlapping regions (Fig. R4a), with 95% confidence ellipses largely coincident, confirming no systematic spatial separation between the two groups. Kolmogorov-Smirnov tests on PC1 and PC2 scores return p = 0.663 and p = 0.427, both well above 0.05, indicating statistically identical distributions between training and test sets (Fig. R4c, d). The scree plot shows that PC1 and PC2 together explain 62.4% of total compositional variance, and the first four PCs account for 85.3%, confirming the 2D PCA projection captures the dominant variation in the dataset (Fig. R4b). These results confirm the random split did not introduce distributional bias and the test set is representative of the training space.

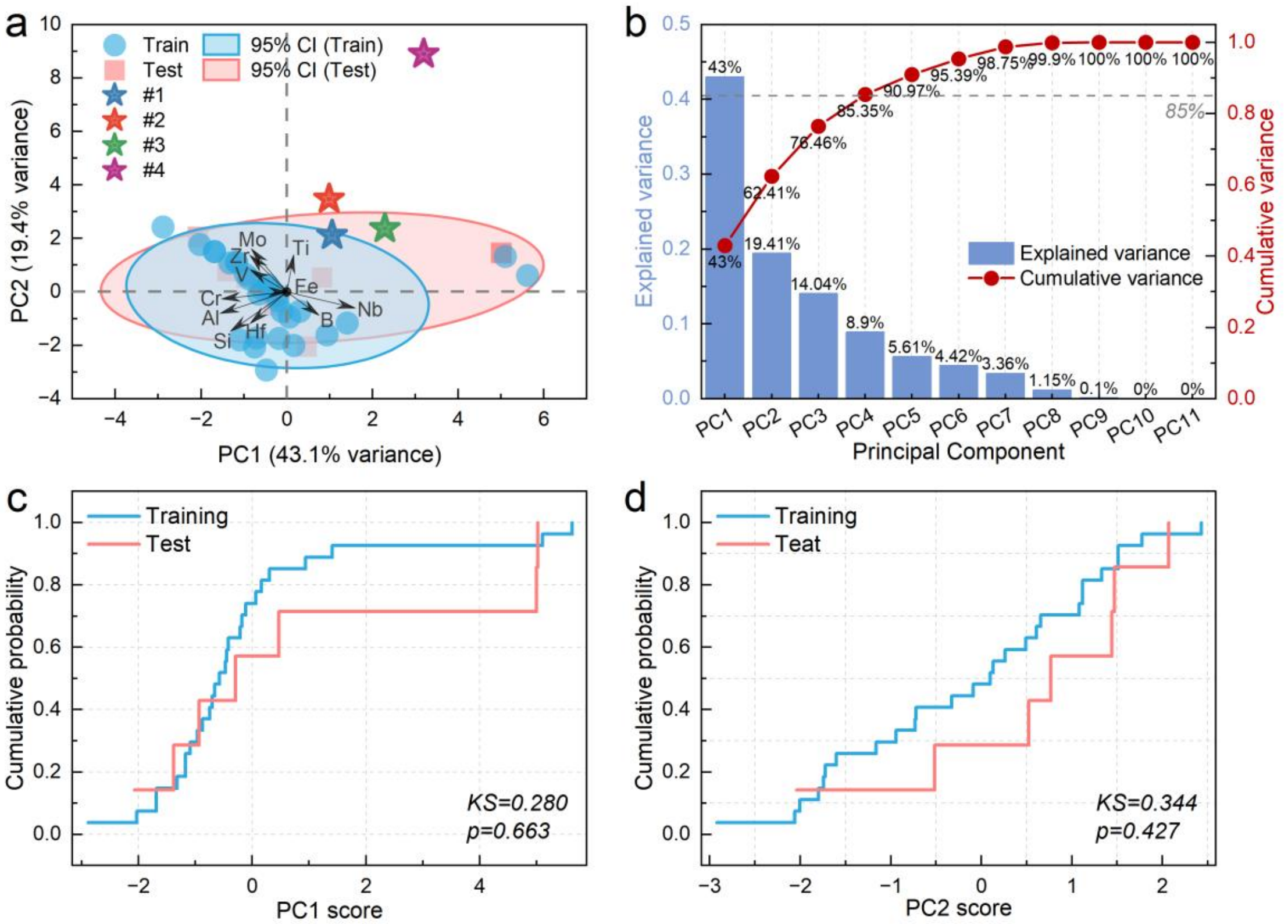


Fig. R4. PCA-based representativeness analysis of the HTC dataset. (a) PCA scatter plot showing training set (n = 27, circles) and test set (n = 7, squares) with 95% confidence ellipses, and four optimized alloys (stars). (b) Scree plot of explained variance per PC (bars) and cumulative variance (red line); dashed line marks 85%. (c, d) Empirical CDFs of PC1 and PC2 scores for training and test sets; KS = 0.280 (p = 0.663) and KS = 0.344 (p = 0.427), confirming no significant distributional difference between the two subsets.

We acknowledge that the limited sample size constrains coverage density in multi-element composition regions. To address potential overestimation of generalization

capability, stratified sampling was applied during train-test splitting, and model robustness was evaluated through 5-fold cross-validation, yielding mean $R^2 = 0.89 \pm 0.01$ and RMSE = 4.4 ± 0.3 MPa across folds. The small standard deviations confirm stable performance across different data partitions. Bootstrap ensemble and Monte Carlo Dropout uncertainty quantification (Supplementary Figs. 35–36) provide calibrated confidence intervals that reflect prediction uncertainty from limited training data, with all experimental test measurements falling within the predicted intervals. Predictions within the validated compositional range are reliable, though extrapolation beyond the training distribution should be treated with caution.

Action taken: The revisions have been added to the Materials and Methods section.

Comment 4: In the NSGA-II multi-objective optimization, KQ and HTC are weighted 1:1, but the basis for this choice is not explained. It is recommended to include a sensitivity analysis to evaluate how alternative weightings influence the optimization outcomes.

**Response:**

The basis for the 1:1 weighting has been clarified, and a sensitivity analysis with two alternative weighting schemes has been added to the manuscript.

In standard NSGA-II, the two objectives compete freely without any weighting, and the algorithm produces a Pareto frontier through rank-based selection. In this work, a weighted scalarization is applied on top of NSGA-II to select a single recommended alloy from the Pareto frontier, rather than to alter the frontier itself. The weight assigned to each objective reflects the relative priority given to that objective during alloy selection. Before weighting, both $K_Q$ and HTC were normalized to [0, 1] using their respective ranges across all feasible solutions, so that differences in absolute magnitude between the two objectives do not cause one to dominate the combined score. Under this normalization, the 1:1 scheme assigns equal priority to both objectives on a common scale.

The 1:1 weighting was selected because both $K_Q$ and HTC must simultaneously meet minimum service thresholds and they are considered equally important: $K_Q$ must exceed 18 $MPa \cdot m^{1/2}$ for room-temperature processing, and HTC must remain competitive with existing Nb-Si alloys at 1250°C for high-temperature service. An alloy that meets only one criterion cannot be used in practice, so equal weighting

reflects the equal necessity and importance of both properties rather than an arbitrary choice. The manuscript already states that this weighting is adjustable: " The weighting can be adjusted for specific applications, with a higher $K_Q$ weight of 60% suited for turbine blades under cyclic loading and a higher HTC weight of 70% suited for static high-temperature structural components." This statement is retained in the revised manuscript alongside the sensitivity analysis below.

To quantify how weighting affects optimization outcomes, we applied three weighting schemes ($K_Q$:HTC = 0.8:0.2, 1:1, and 0.2:0.8) to all four element systems (5- to 8-element alloys) under identical NSGA-II settings. For each scheme, the recommended target alloy was selected as the candidate with the highest combined score from the Pareto frontier. Results are shown in Fig. R5 and summarized in Table R1.

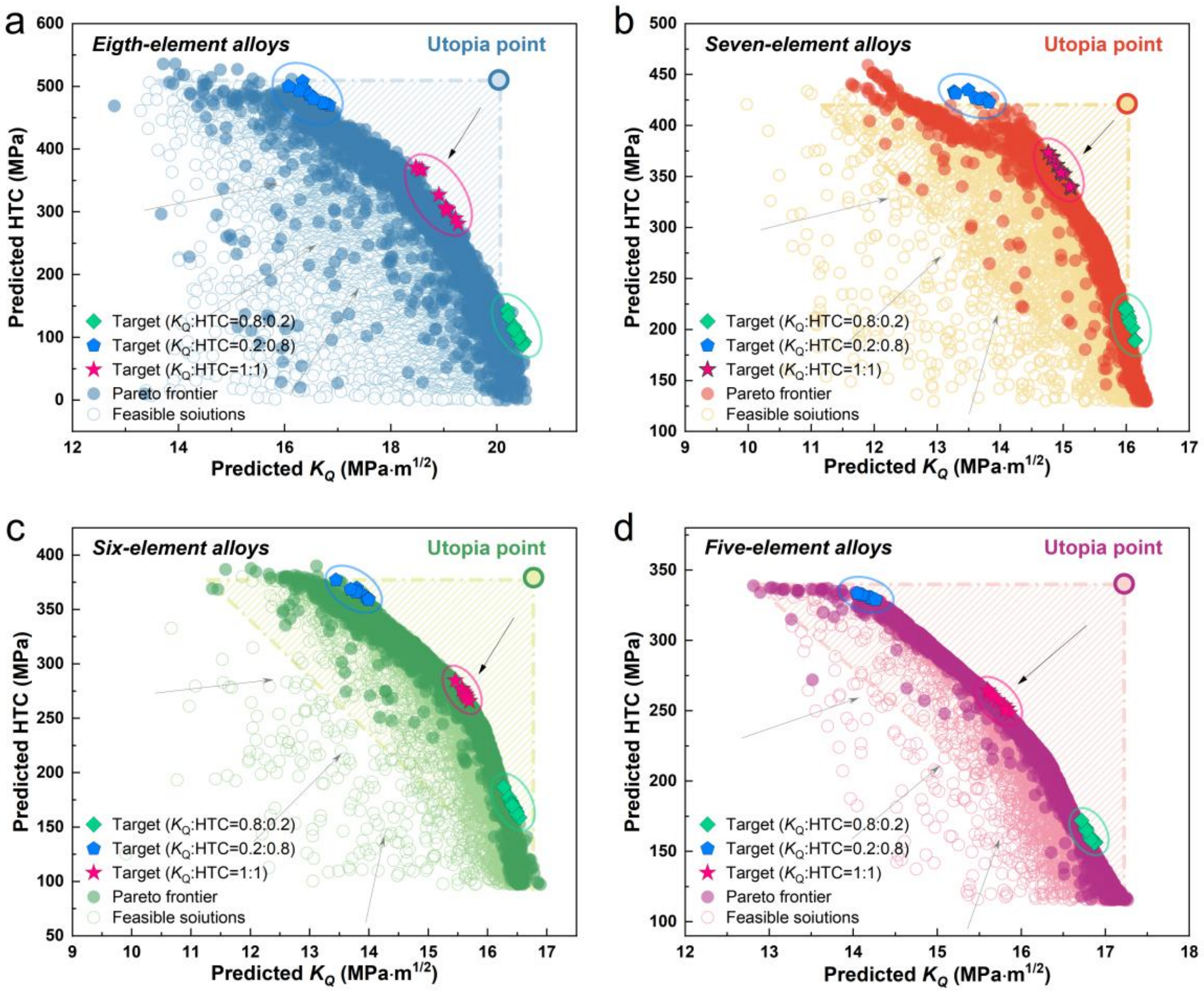


Fig. R5. Sensitivity analysis of NSGA-II optimization to weighting schemes for (a) 8-element, (b) 7-element, (c) 6-element, and (d) 5-element alloy systems. Gray open circles are all feasible solutions; filled circles are the Pareto frontier; colored symbols mark the recommended alloy under each scheme: green diamond ($K_Q$:HTC = 0.8:0.2), blue circle ($K_Q$:HTC = 0.2:0.8), pink star ($K_Q$:HTC = 1:1). The open circle denotes the utopia point. Arrows show the direction from the utopia point to each recommended alloy.

As shown in Fig. R5, shifting weight toward $K_Q$ (0.8:0.2) moves the recommended alloy toward higher $K_Q$ and lower HTC along the Pareto frontier, while shifting toward HTC (0.2:0.8) produces the opposite trend. This behavior is consistent across all four element systems, confirming that the weighting scheme provides directional control over alloy selection. The Pareto frontier shape and the dominant compositional trends remain unchanged across all three schemes, indicating that the underlying optimization landscape is robust to weighting variation. The 1:1 scheme selects alloys in the intermediate region of the Pareto frontier, representing a balanced trade-off between the two objectives. Table R1 shows that the recommended alloys under all three schemes satisfy both the $K_Q$ and HTC service thresholds, supporting the suitability of the 1:1 scheme as the baseline for alloy recommendation.

Table R1. Predicted $K_Q$ and HTC of the recommended candidate alloy selected from the Pareto frontier under three weighting schemes for each element system. For each scheme, one representative alloy is reported.

| System | Weighting scheme | Predicted $K_Q$ (MPa·m) | Predicted HTC (MPa) |
|---|---|---|---|
| 8-element | $K_Q$:HTC = 0.8:0.2 | 20.33 | 116.84 |
| 8-element | $K_Q$:HTC = 1:1 | 18.71 | 325.67 |
| 8-element | $K_Q$:HTC = 0.2:0.8 | 16.07 | 470.55 |
| 7-element | $K_Q$:HTC = 0.8:0.2 | 16.14 | 221.60 |
| 7-element | $K_Q$:HTC = 1:1 | 15.39 | 361.12 |
| 7-element | $K_Q$:HTC = 0.2:0.8 | 13.26 | 422.87 |
| 6-element | $K_Q$:HTC = 0.8:0.2 | 16.41 | 186.91 |
| 6-element | $K_Q$:HTC = 1:1 | 16.24 | 278.33 |
| 6-element | $K_Q$:HTC = 0.2:0.8 | 13.44 | 366.33 |
| 5-element | $K_Q$:HTC = 0.8:0.2 | 16.71 | 166.82 |
| 5-element | KQ:HTC = 1:1 | 15.04 | 259.49 |
| 5-element | KQ:HTC = 0.2:0.8 | 14.05 | 329.16 |

Action taken: The revisions have been added to the "Multi-objective optimization and Experimental validation" subsection in the Results and discussion section.

Thank you and the reviewers again. We hope that our responses could meet the requirements of the reviewers and you. If there are any questions about our revised manuscript, please don't hesitate to contact us.

Ruirun Chen

Ph.D, Professor

Director, Department of Interdisciplinary Science

Associate director, National Key Laboratory for Precision Hot Processing of Metals

Harbin Institute of Technology, Harbin, 150001, China

Email: ruirunchen@hit.edu.cn
